# Supplementary material for: Switchable organoplatinum metallacycles with high quantum yields and tunable fluorescence wavelengths
Source: Nat Commun. 2019 Sep 19;10:4285. doi: 10.1038/s41467-019-12204-7 (PMC6753206; doi:10.1038/s41467-019-12204-7)
Supplement: Supplementary file 1 — Supplementary Information [file 41467_2019_12204_MOESM1_ESM.pdf]

Supporting Information for

Switchable organoplatinum metallacycles with high quantum yields and tunable fluorescence wavelengths

Zhu et al.

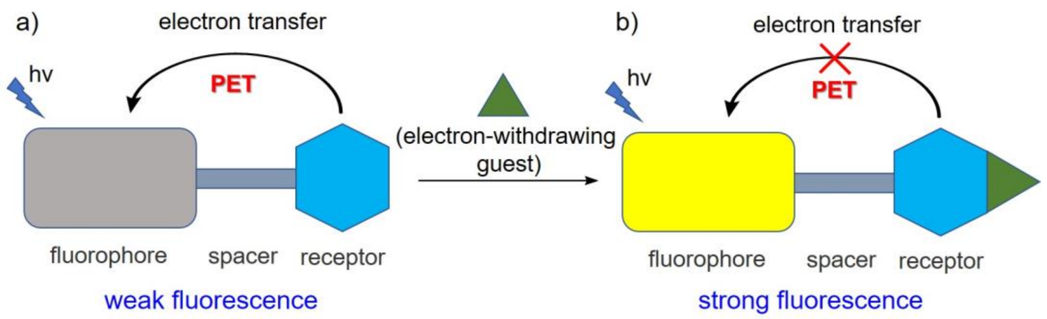

Supplementary Figure 1. Schematic representation of PET-based fluorescent system. The fluorescent system before (a) and after (b) binding the electron-withdrawing guest.

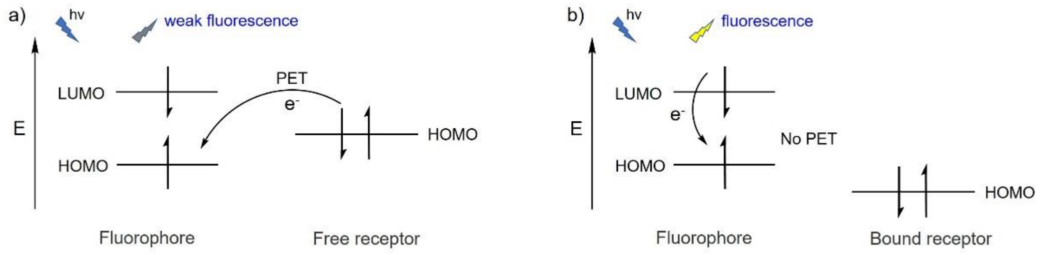

Supplementary Figure 2. Frontier orbital theory for the PET effect. The fluorescent system before (a) and after (b) binding the receptor.

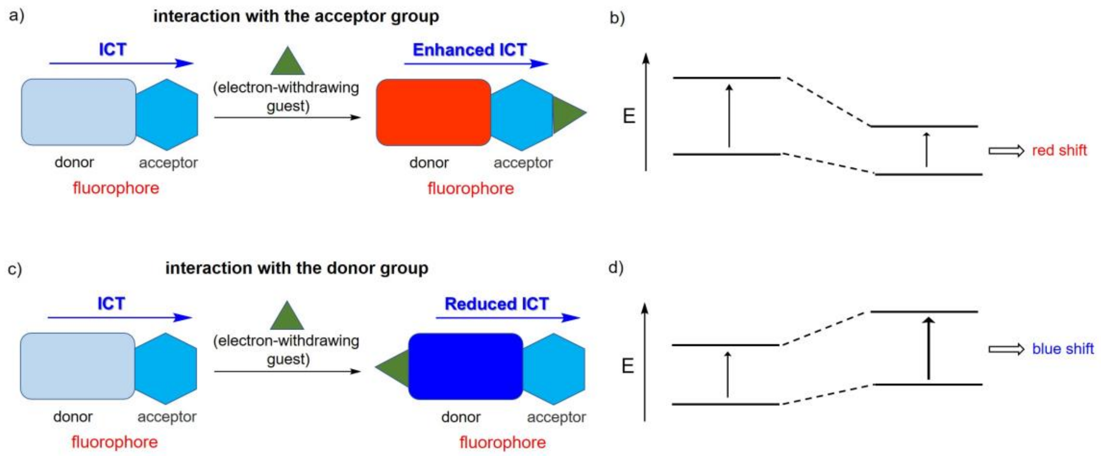

Supplementary Figure 3. Schematic representation of ICT-based fluorescent systems. Schematic representation (a) and orbital theory (b) of the electron-accepting part of ICT-based fluorescent system interacts with an electron-withdrawing guest. Schematic representation (c) and orbital theory (d) of the electron-donating part of ICT-based fluorescent system interacts with an electron-withdrawing guest.

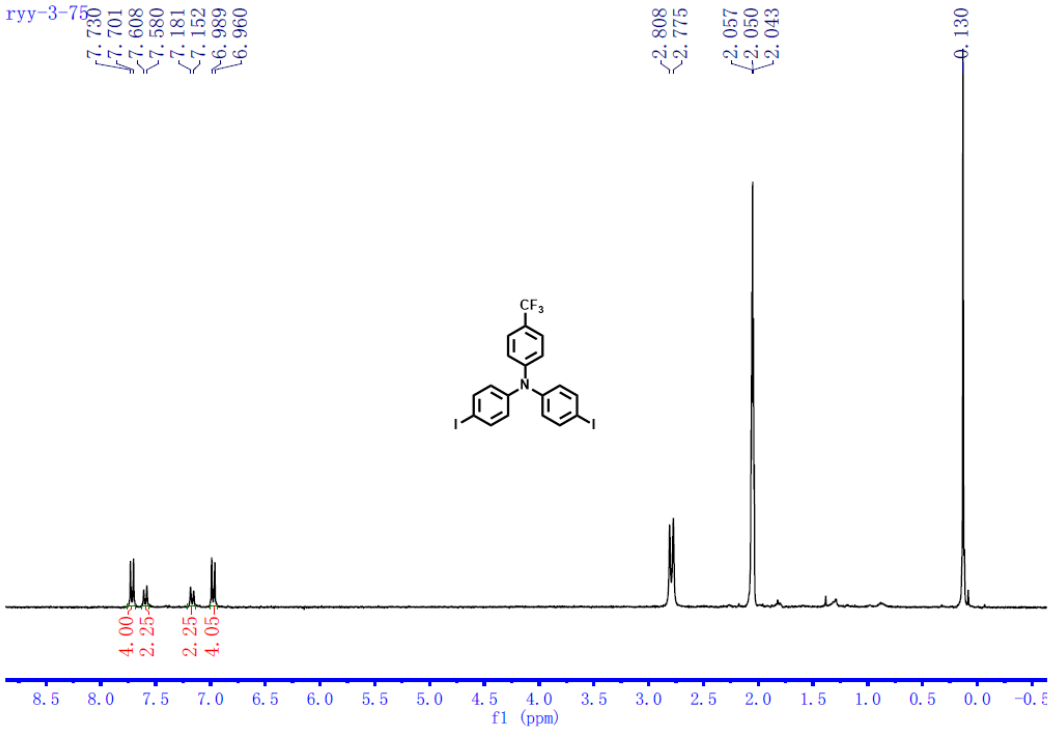

Supplementary Figure 4. ^{1}H NMR spectrum of **L1** (300 MHz, d_6-acetone).

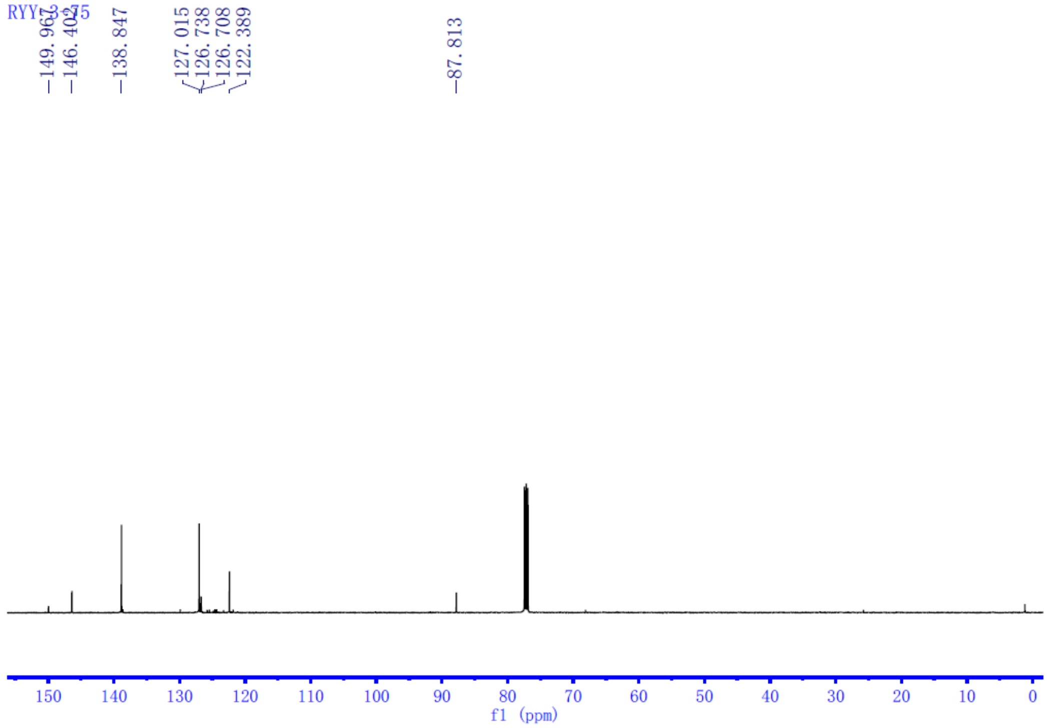

Supplementary Figure 5. ^{13}C NMR spectrum of **L1** (126 MHz, $CDCl_3$).

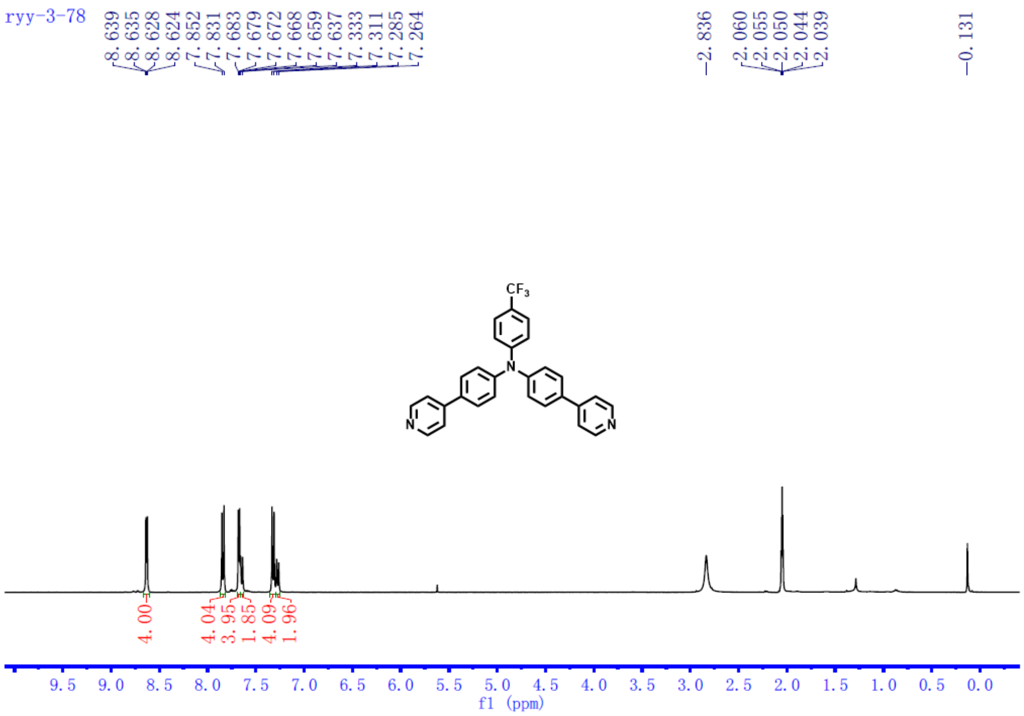

Supplementary Figure 6. ^{1}H NMR spectrum of **L1** (400 MHz, d_6-acetone).

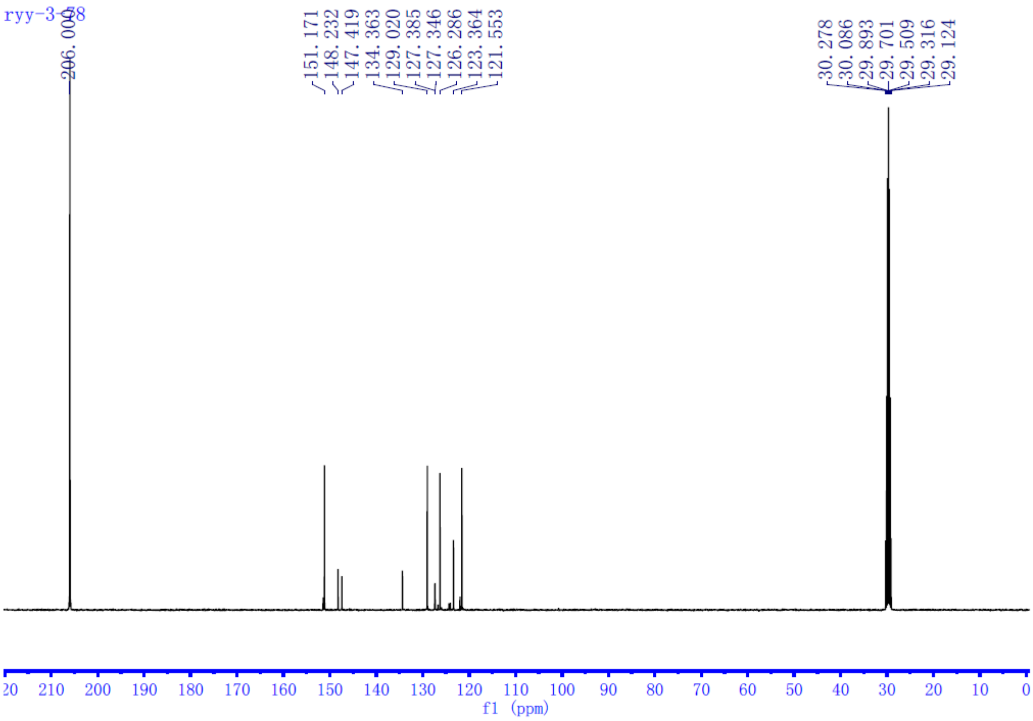

Supplementary Figure 7. ^{13}C NMR spectrum of **L1** (126 MHz, d_6-acetone).

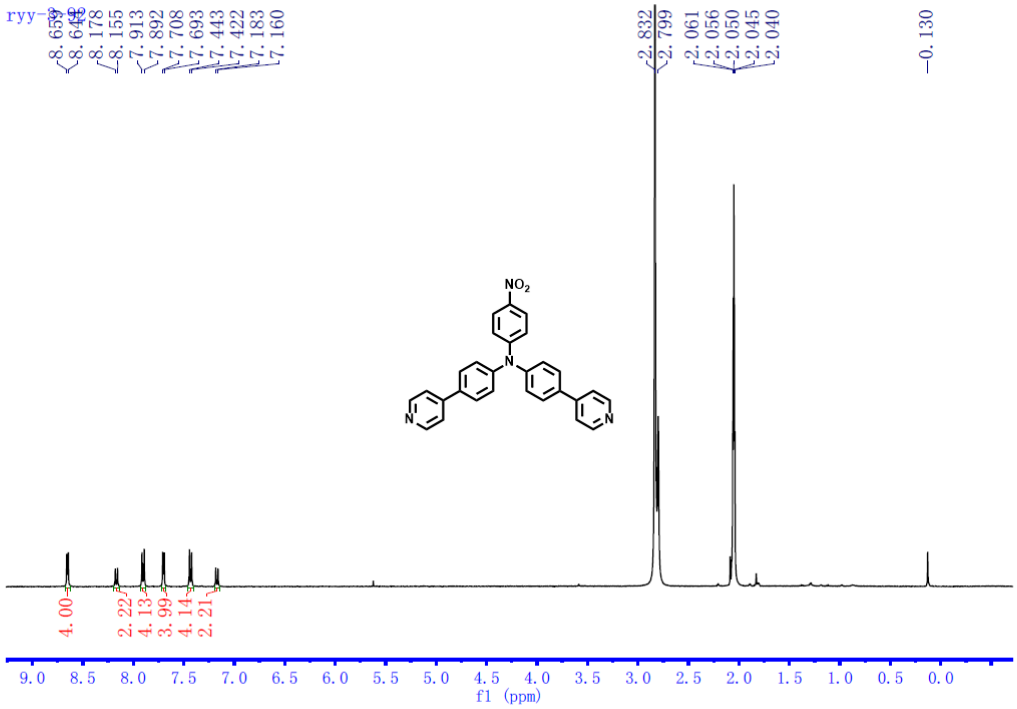

Supplementary Figure 8. ^{1}H NMR spectrum of **L2** (400 MHz, d_6-acetone).

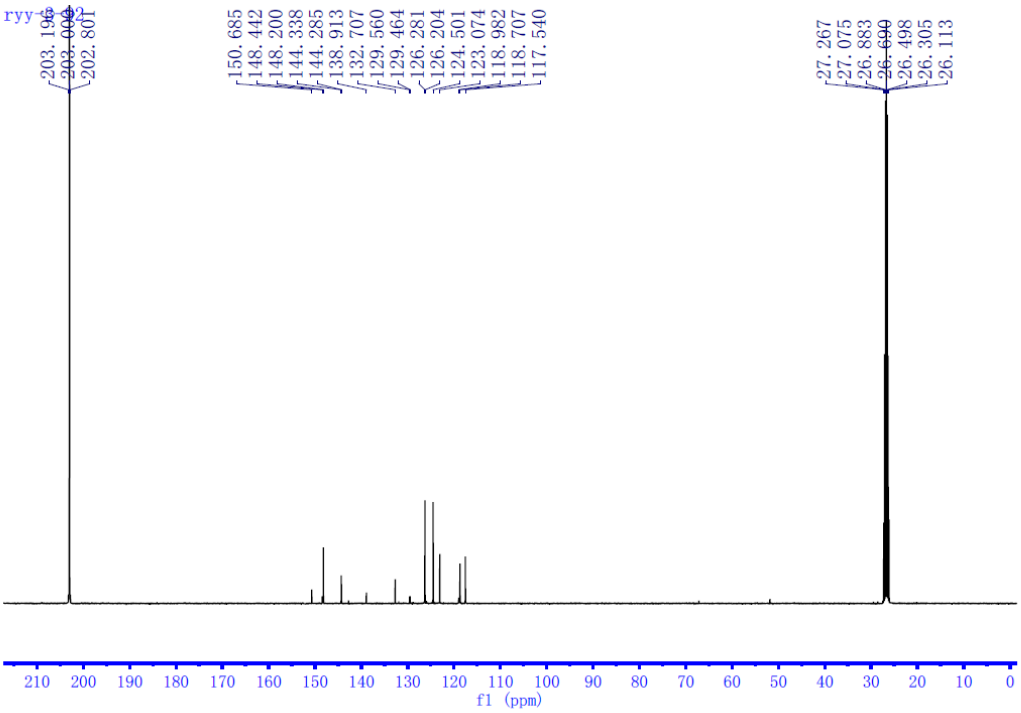

Supplementary Figure 9. ^{13}C NMR spectrum of **L2** (126 MHz, d_6-acetone).

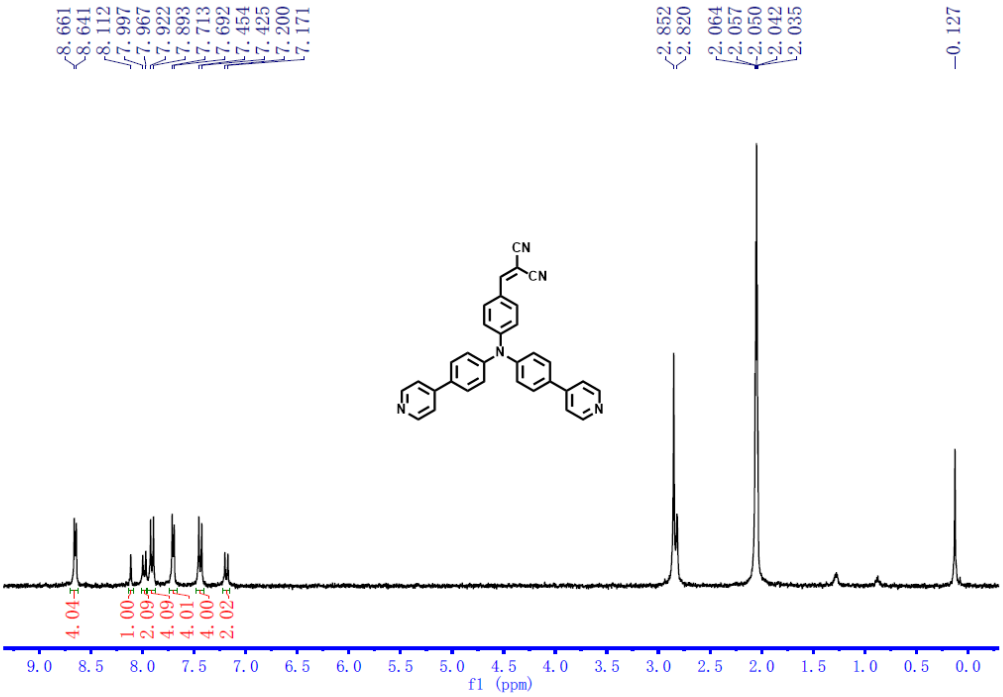

Supplementary Figure 10. ^{1}H NMR spectrum of **L3** (300 MHz, d_6-acetone).

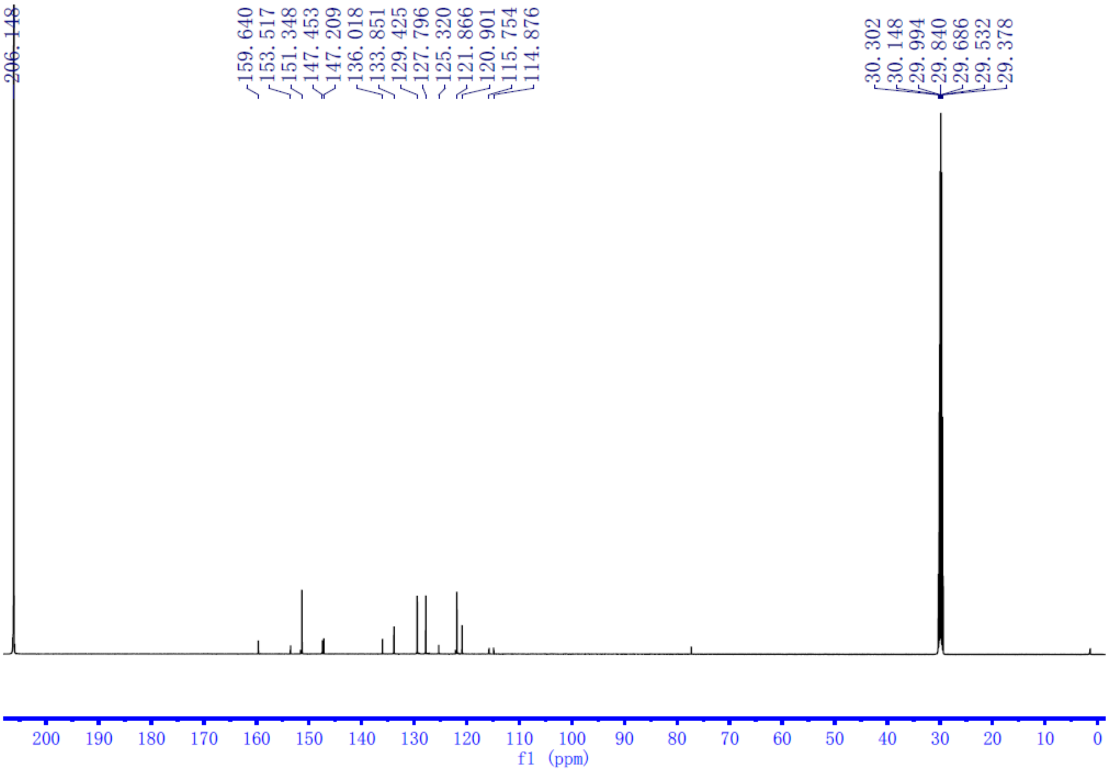

Supplementary Figure 11. ^{13}C NMR spectrum of **L3** (126 MHz, d_6-acetone).

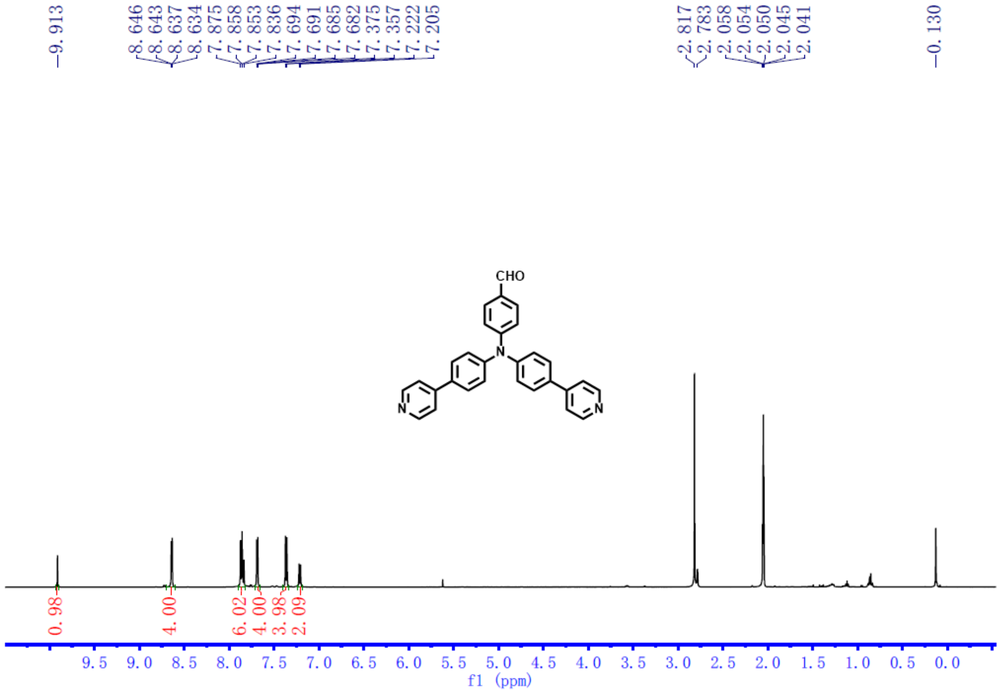

Supplementary Figure 12. ^{1}H NMR spectrum of **L4** (300 MHz, d_6-acetone).

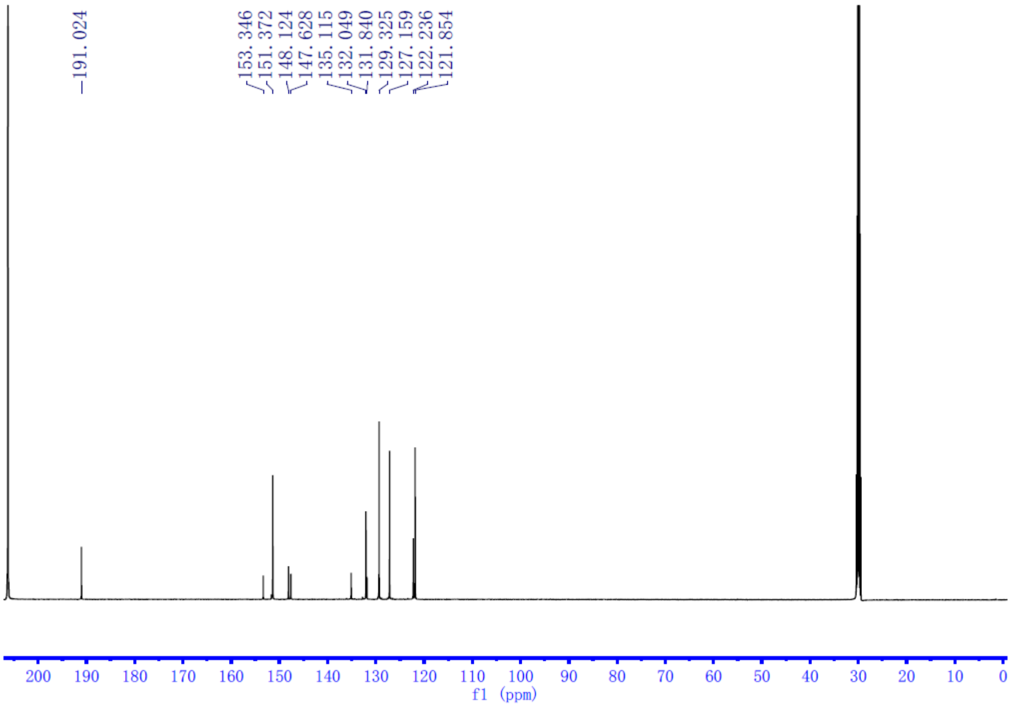

Supplementary Figure 13. ^{13}C NMR spectrum of **L4** (126 MHz, d_6-acetone).

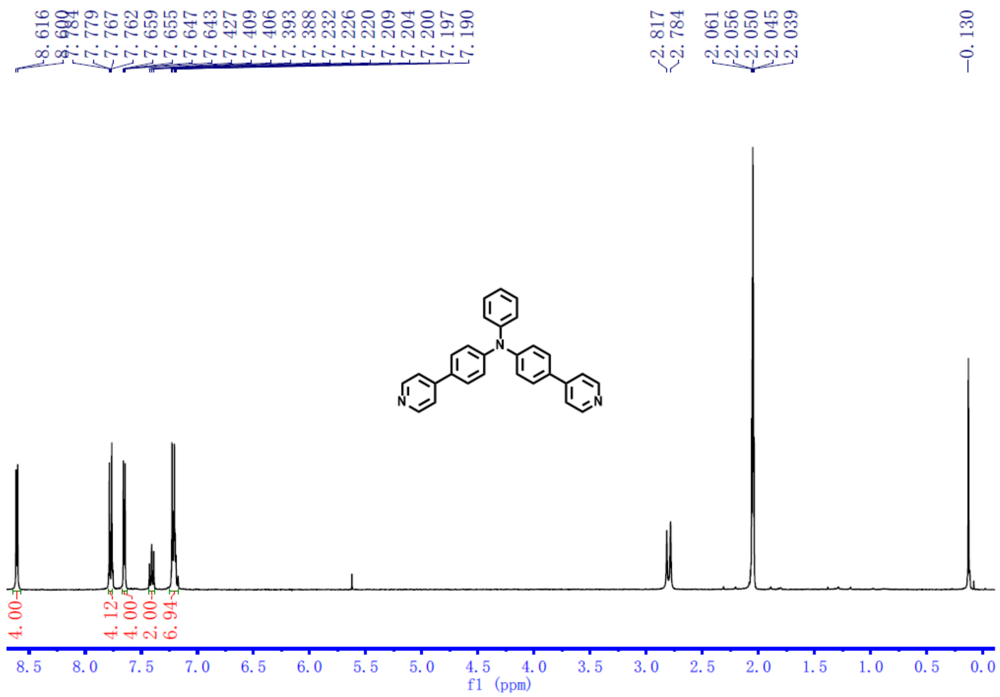

Supplementary Figure 14. ^{1}H NMR spectrum of **L5** (400 MHz, d_6-acetone).

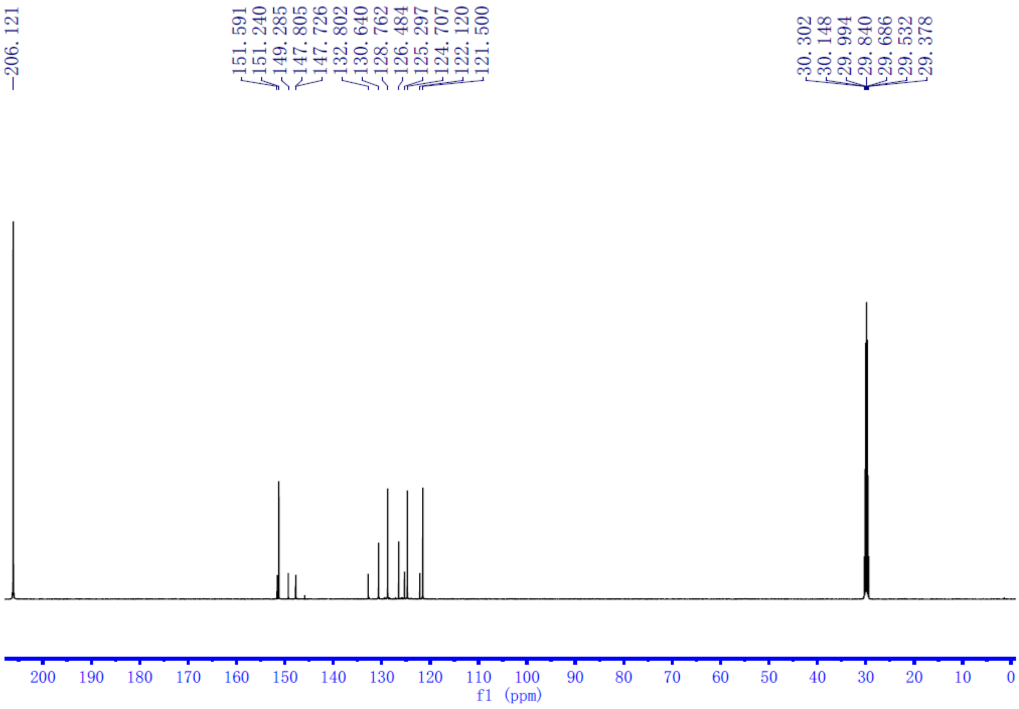

Supplementary Figure 15. ^{13}C NMR spectrum of **L5** (126 MHz, d_6-acetone).

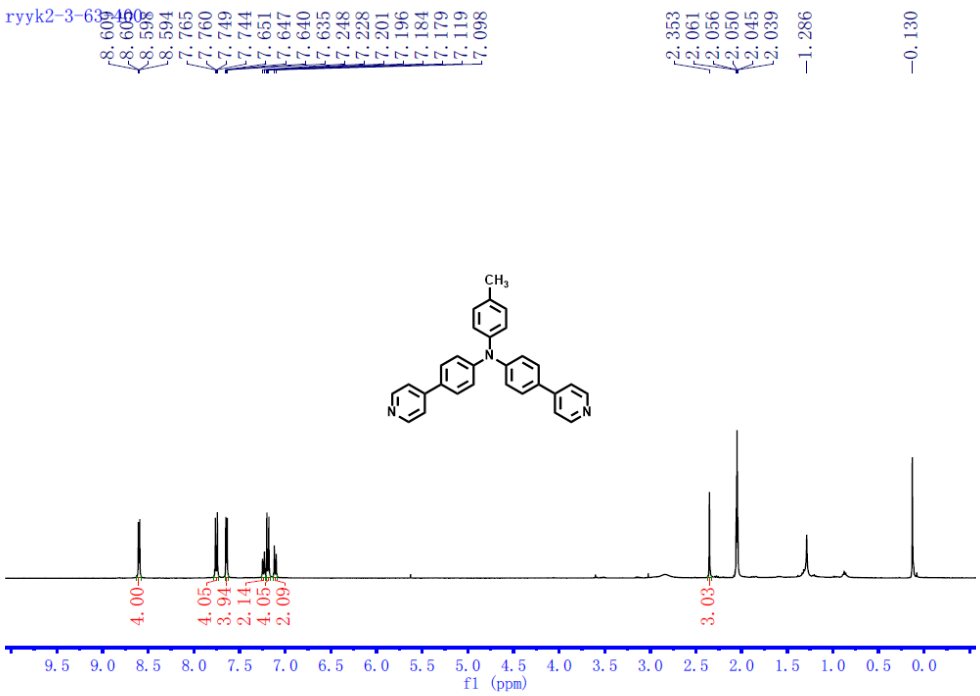

Supplementary Figure 16. ^{1}H NMR spectrum of **L6** (400 MHz, d_6-acetone).

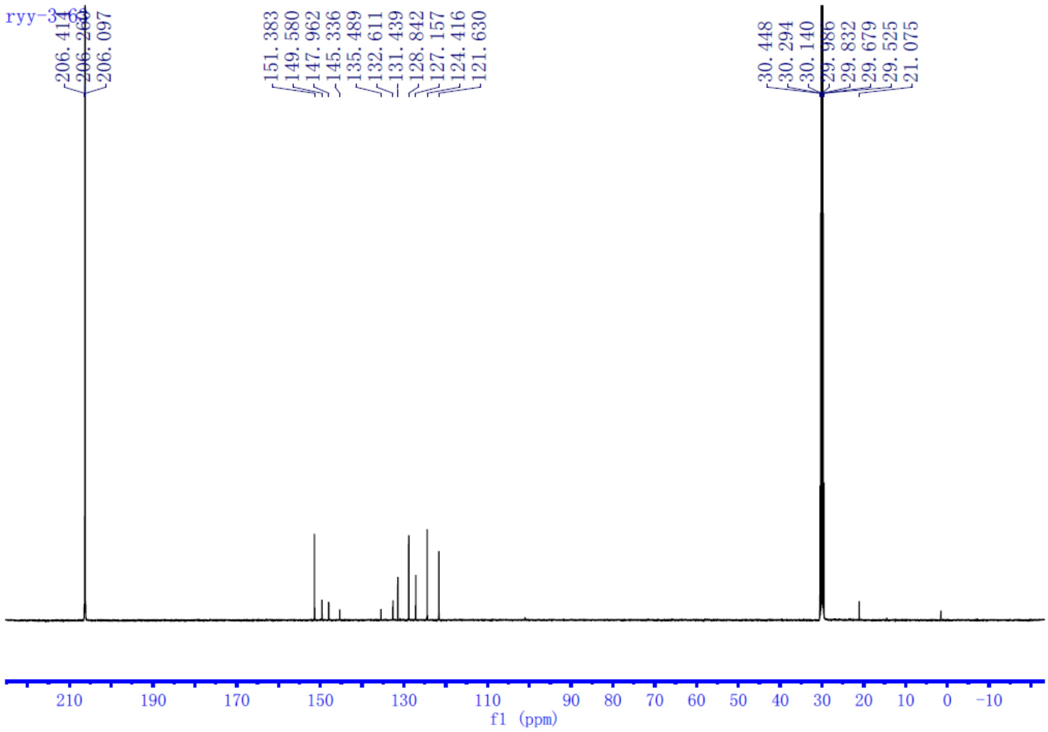

Supplementary Figure 17. ^{13}C NMR spectrum of **L6** (126 MHz, d_6-acetone).

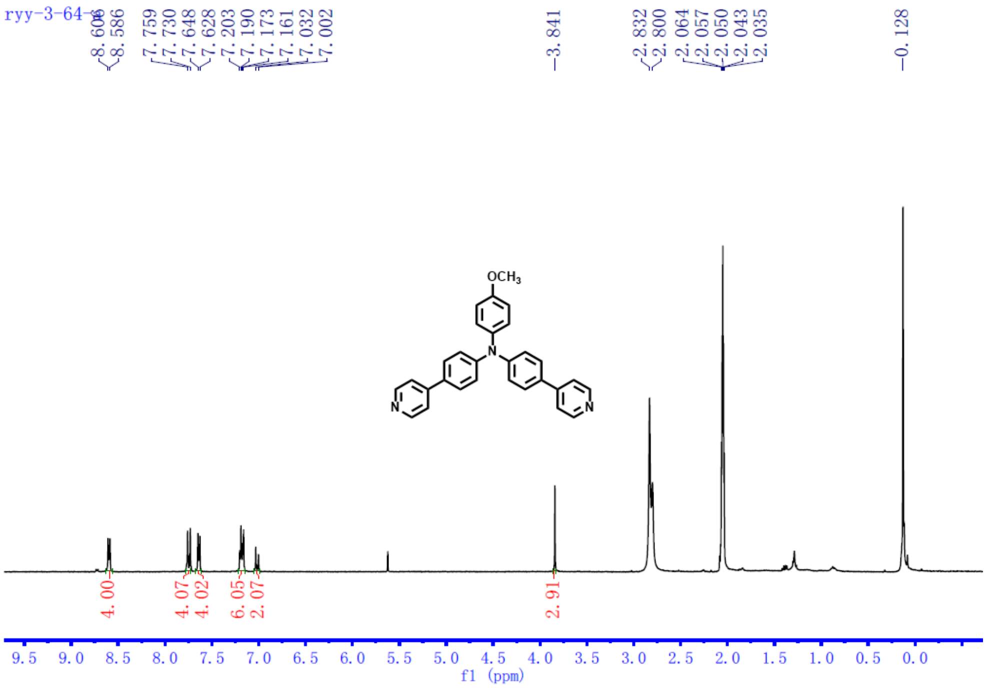

Supplementary Figure 18. ^{1}H NMR spectrum of **L7** (400 MHz, d_6-acetone).

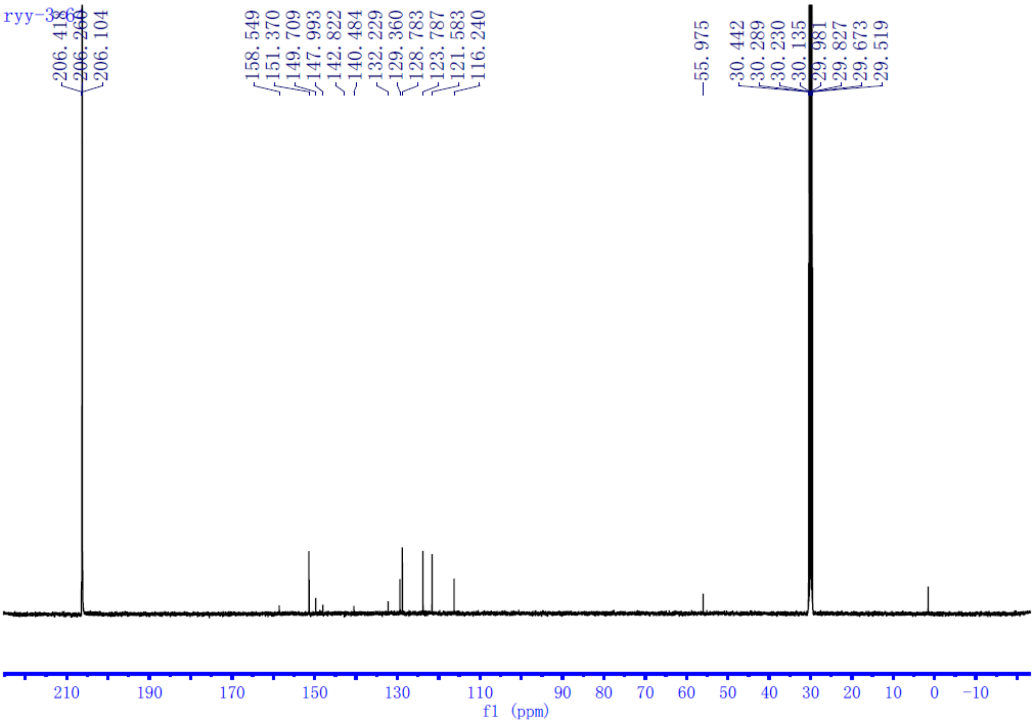

Supplementary Figure 19. ^{13}C NMR spectrum of **L7** (126 MHz, d_6-acetone).

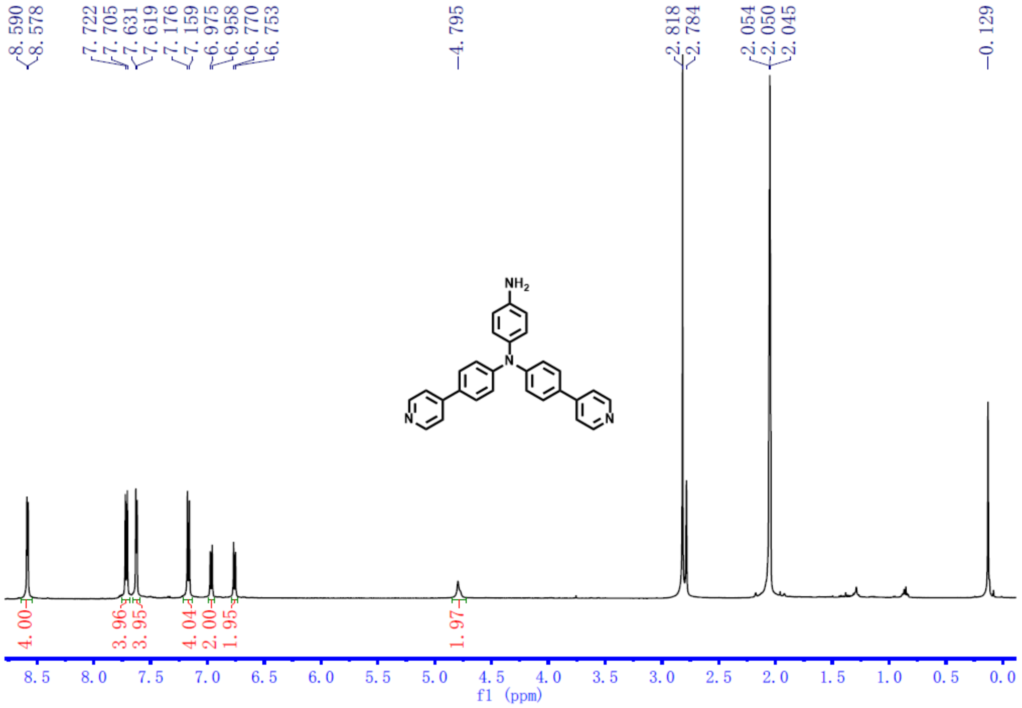

Supplementary Figure 20. ^{1}H NMR spectrum of **L8** (500 MHz, d_6-acetone).

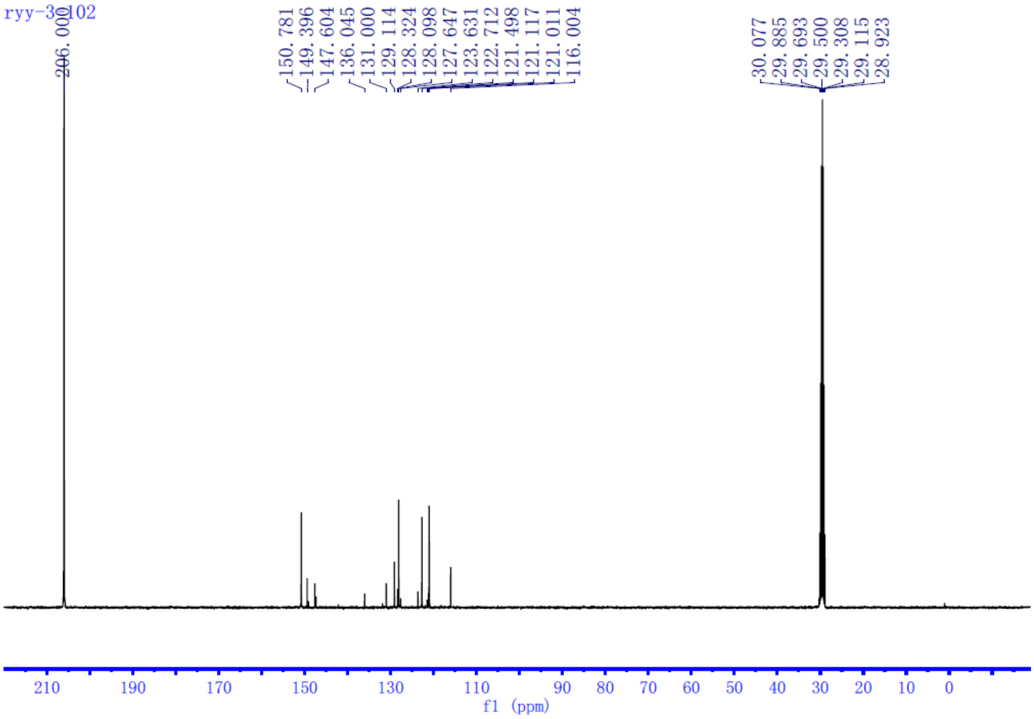

Supplementary Figure 21. ^{13}C NMR spectrum of **L8** (126 MHz, d_6-acetone).

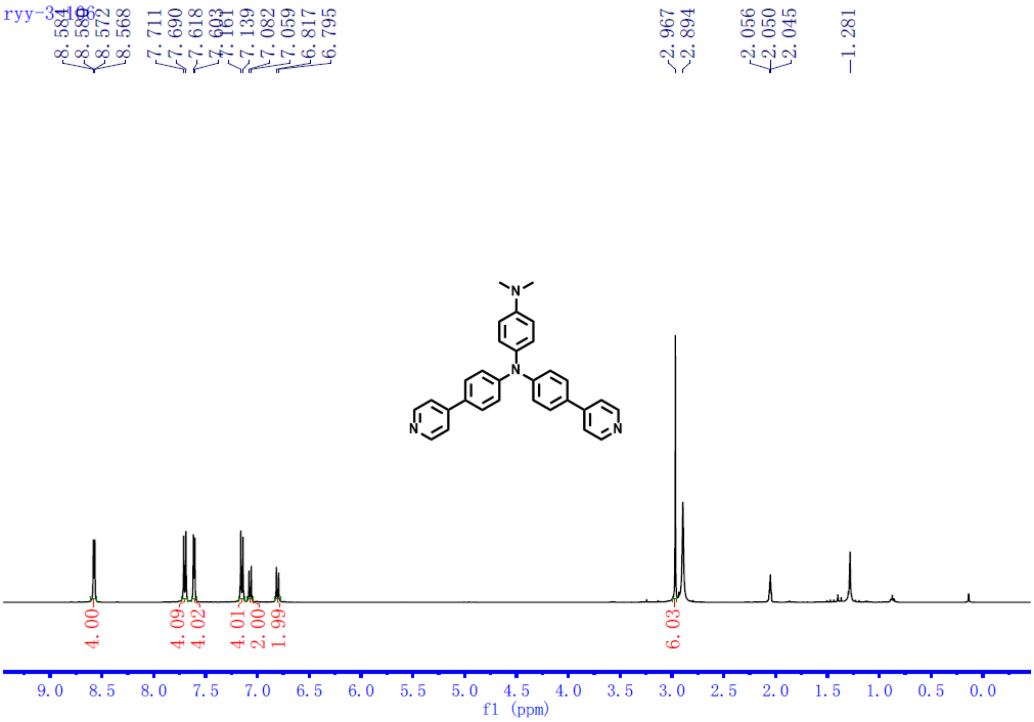

Supplementary Figure 22. ^{1}H NMR spectrum of **L9** (400 MHz, d_6-acetone).

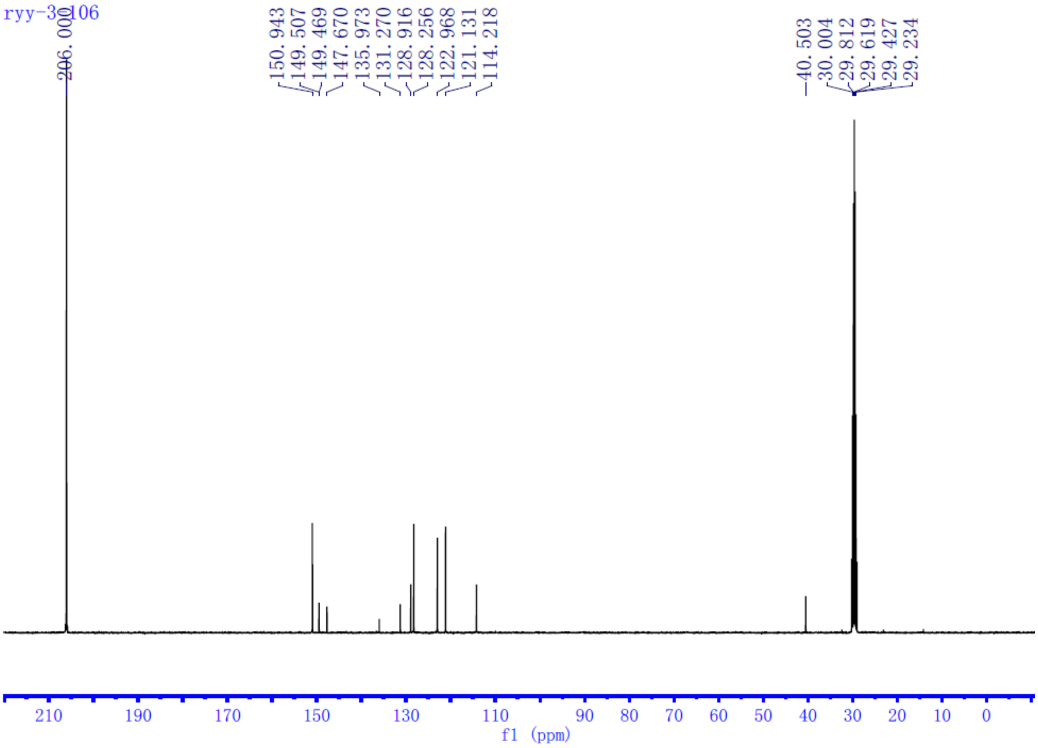

Supplementary Figure 23. ^{13}C NMR spectrum of **L9** (126 MHz, d_6-acetone).

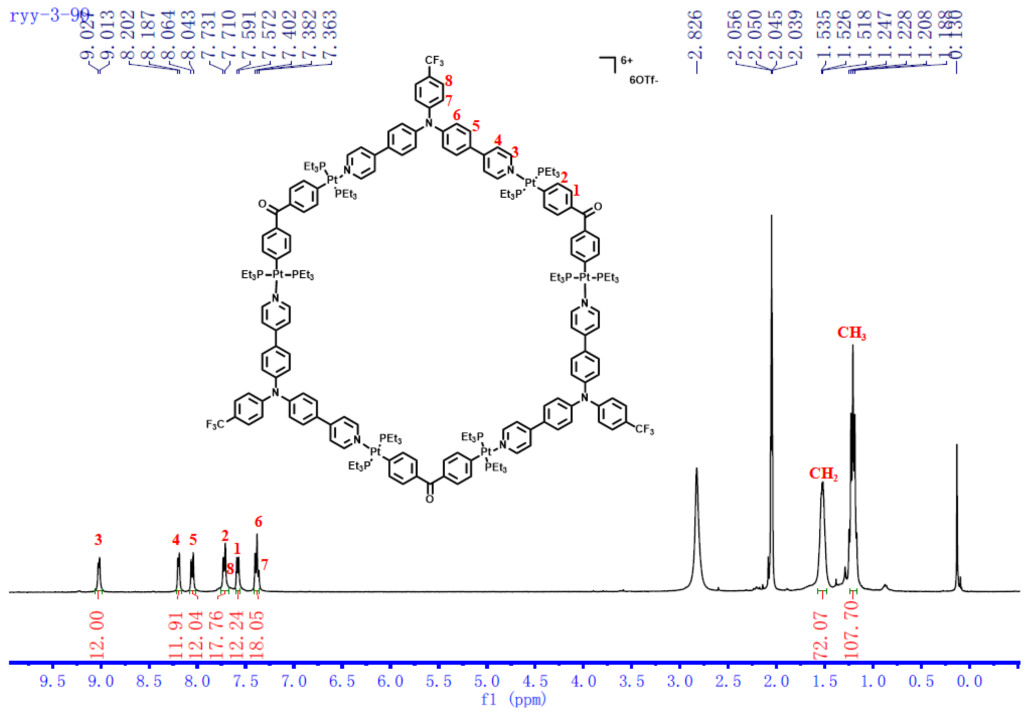

Supplementary Figure 24. ^{1}H NMR spectrum of **H1** (400 MHz, d_6-acetone).

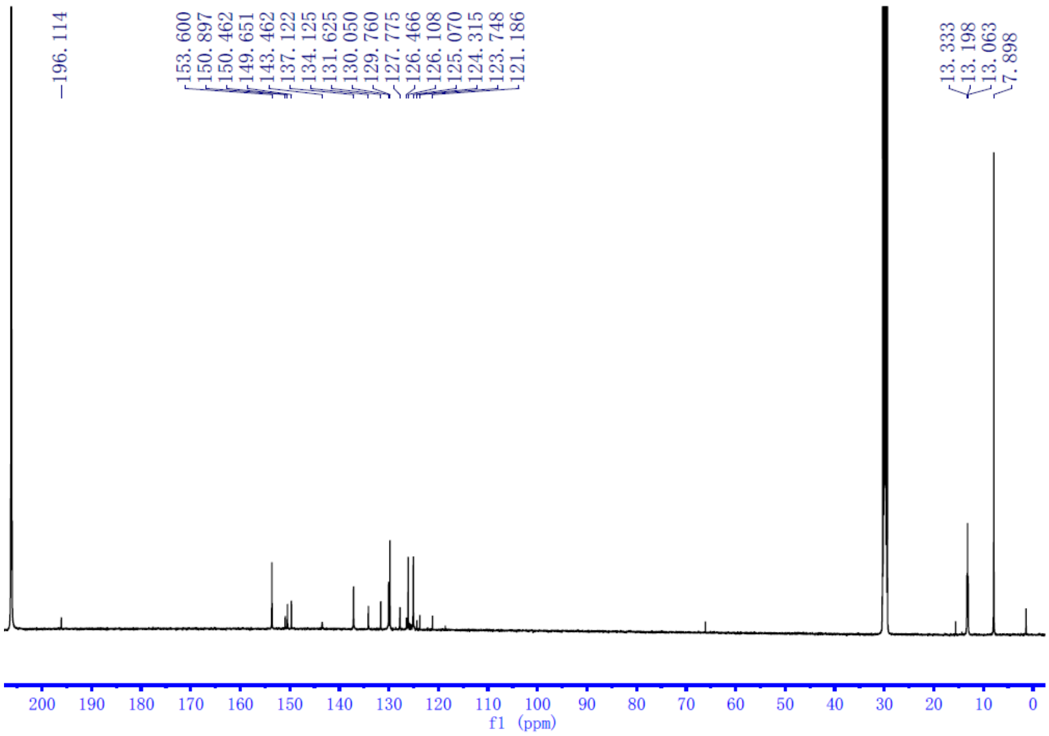

Supplementary Figure 25. ^{13}C NMR spectrum of **H1** (126 MHz, d_6-acetone).

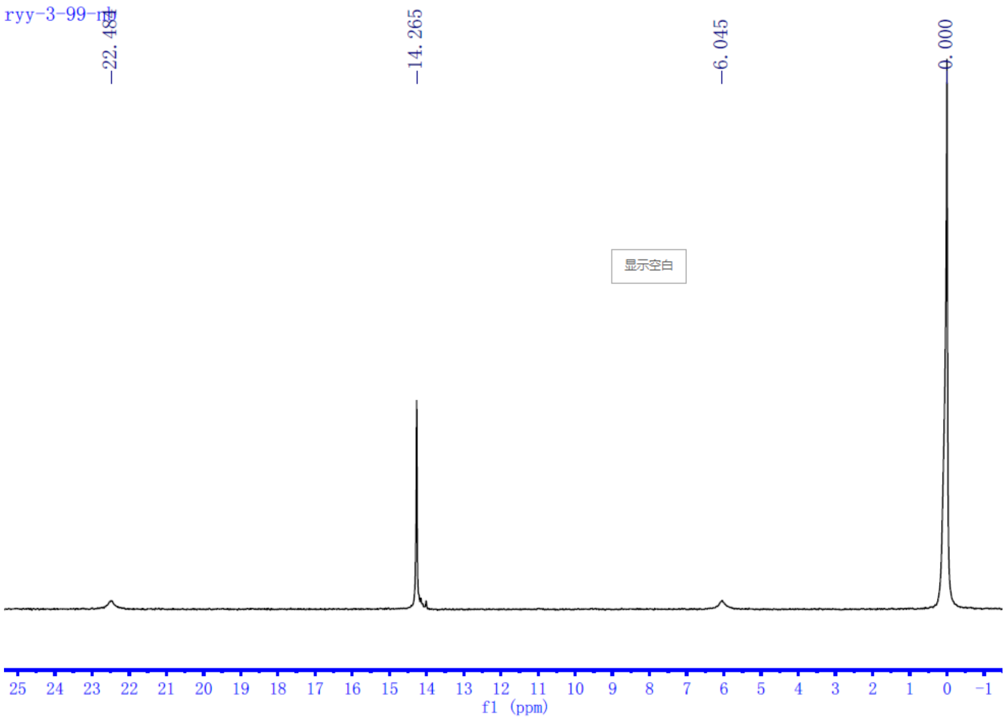

Supplementary Figure 26. ^{31}P NMR spectrum of **H1** (161.9 MHz, d_6-acetone).

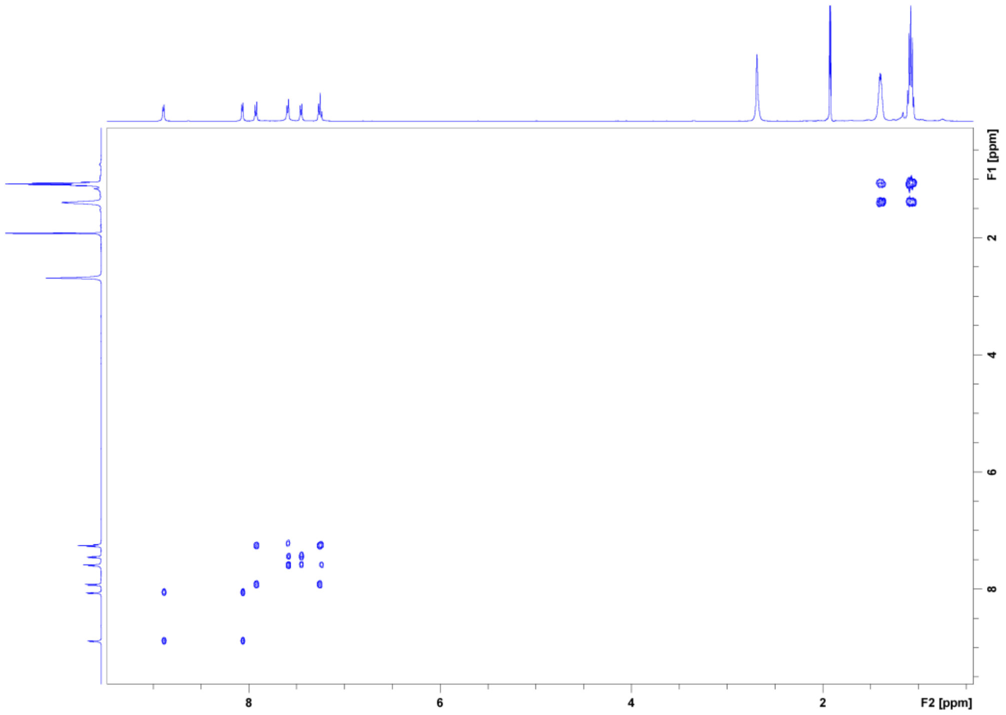

Supplementary Figure 27. ^{1}H-^{1}H COSY spectrum of **H1** (500 MHz, d_6-acetone).

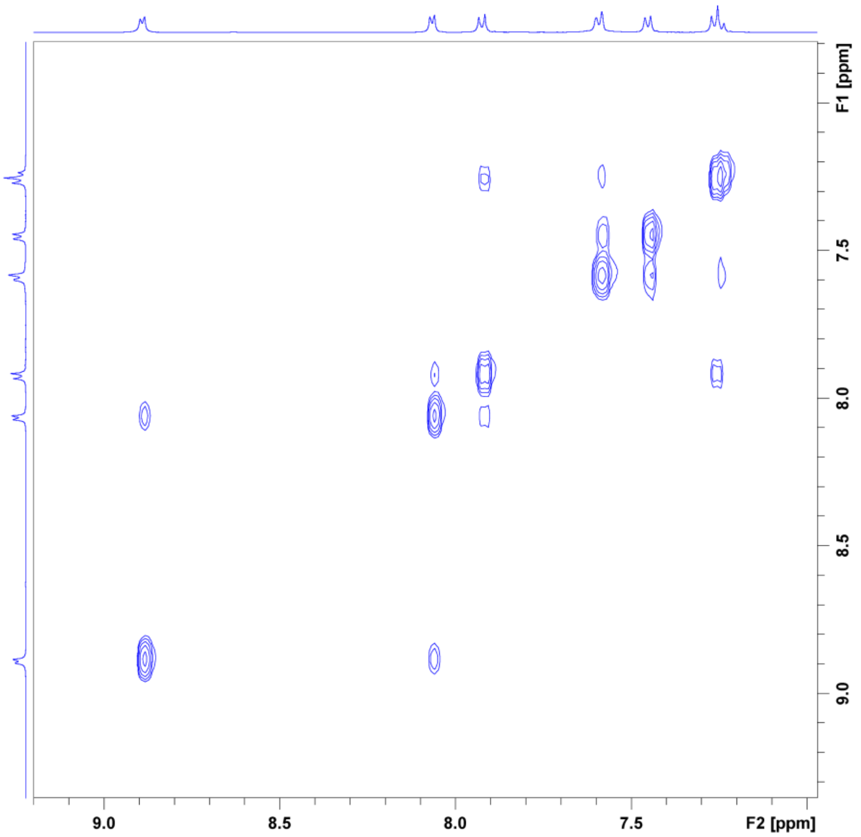

Supplementary Figure 28. Aromatic region of the ^{1}H-^{1}H NOESY spectrum of **H1** (500 MHz, d_6-acetone).

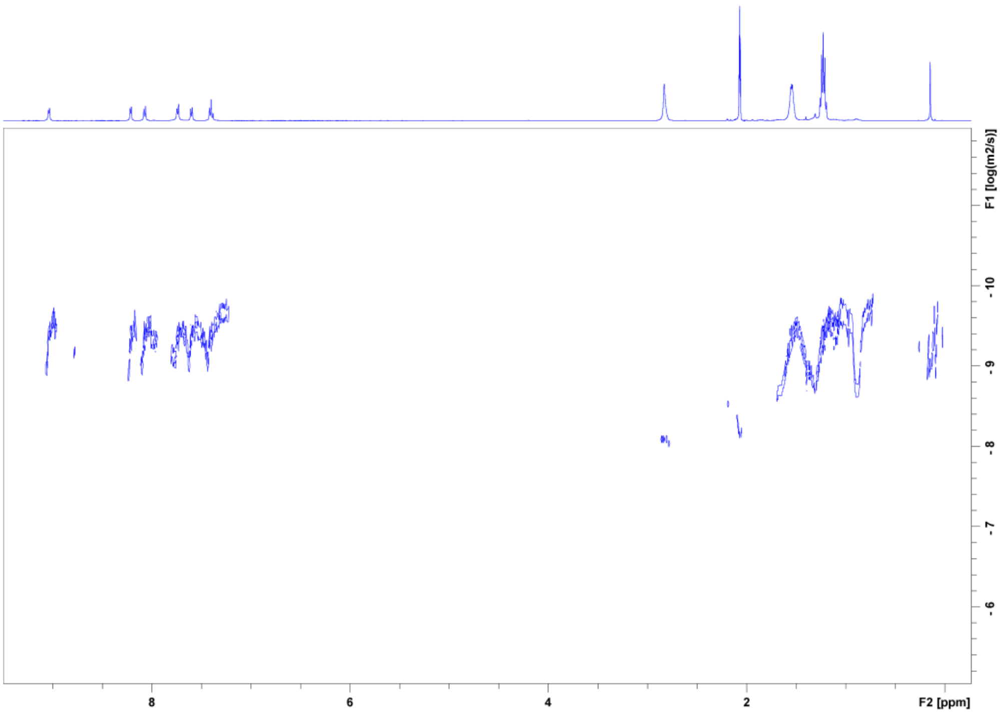

Supplementary Figure 29. ^{1}H DOSY spectrum of **H1** (500 MHz, d_6-acetone).

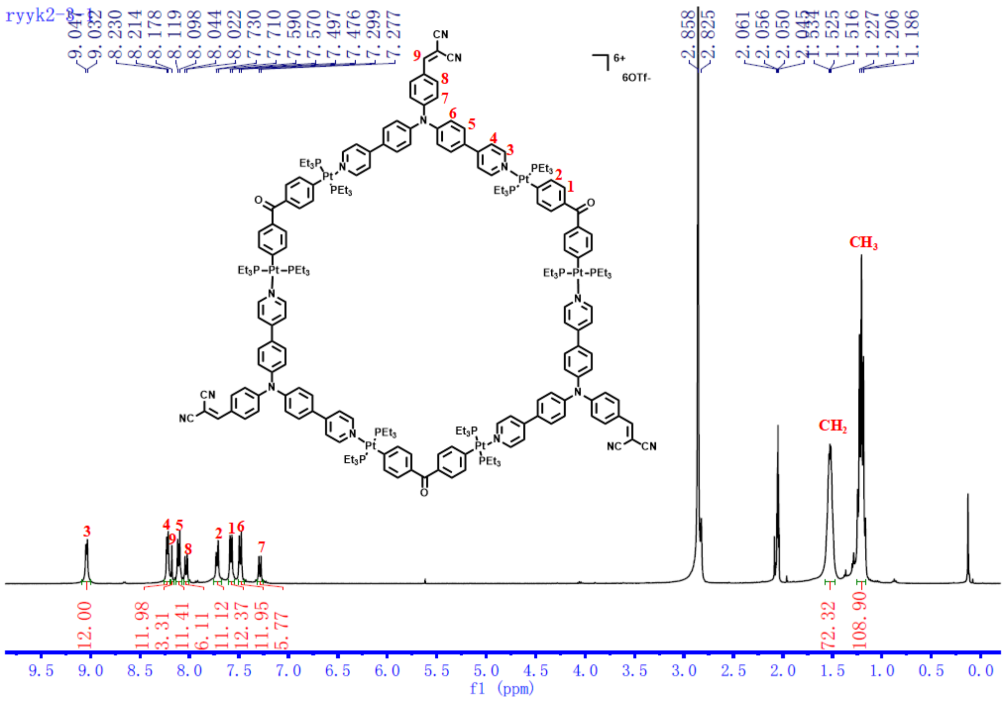

Supplementary Figure 30. ^{1}H NMR spectrum of **H2** (400 MHz, d_6-acetone).

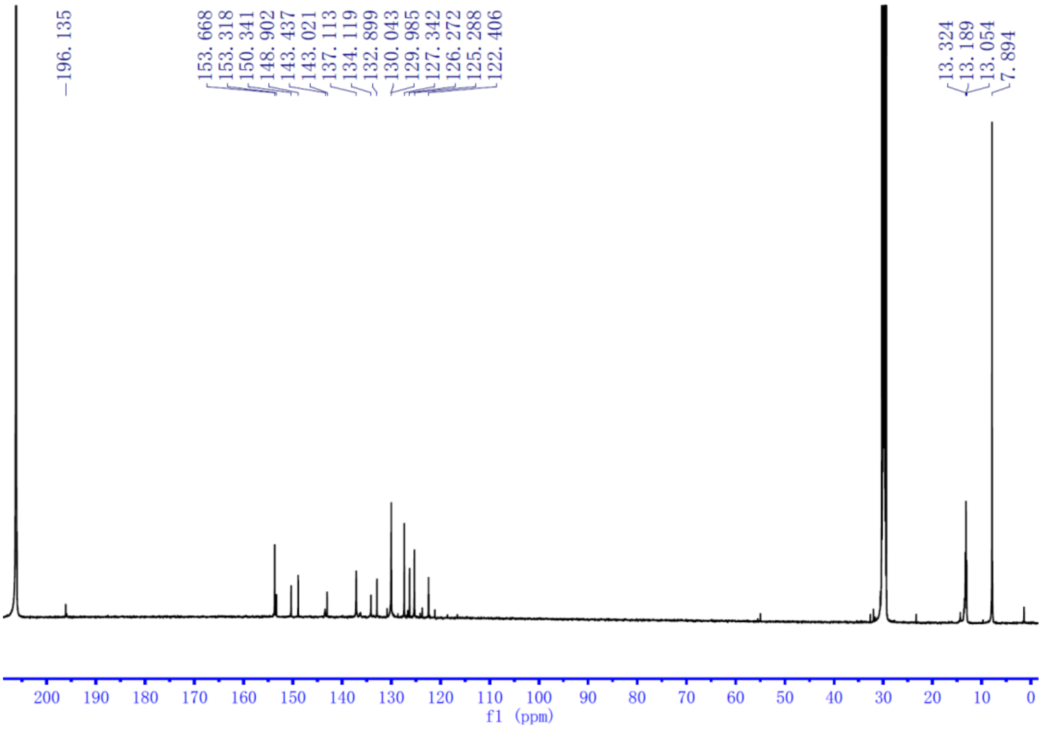

Supplementary Figure 31. ^{13}C NMR spectrum of **H2** (126 MHz, d_6-acetone).

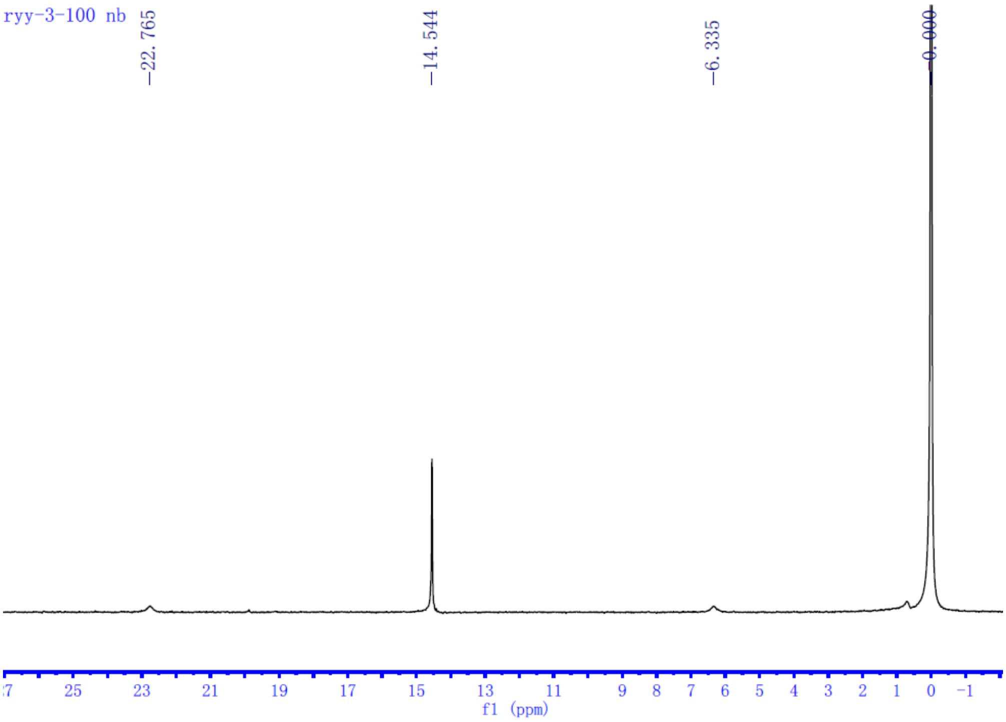

Supplementary Figure 32. ^{31}P NMR spectrum of **H2** (161.9 MHz, d_6-acetone).

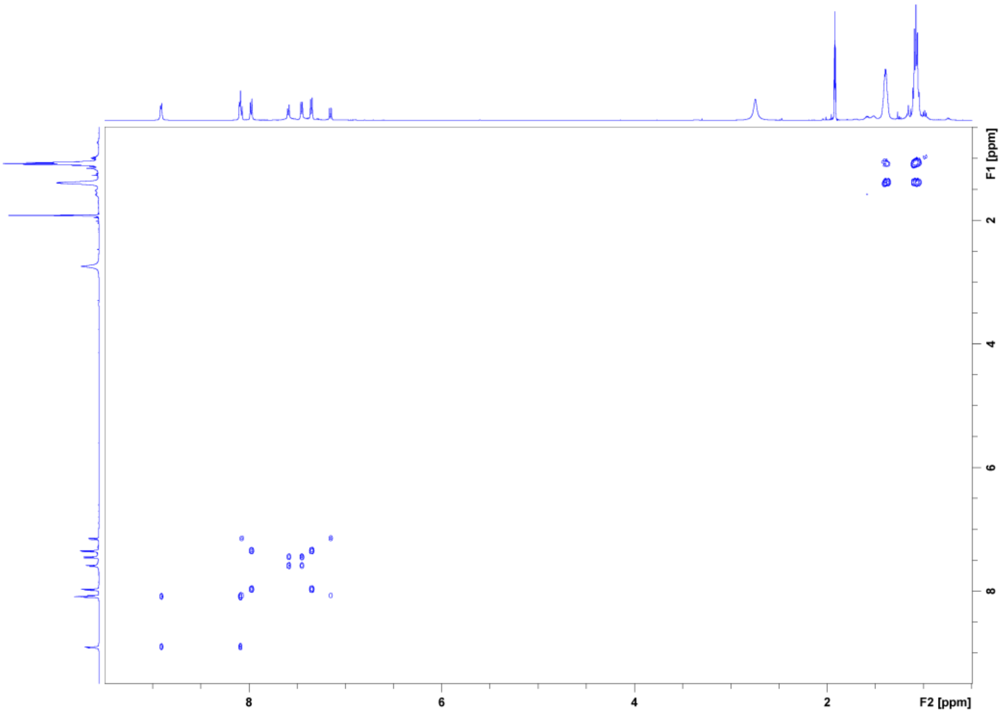

Supplementary Figure 33. ^{1}H-^{1}H COSY spectrum of **H2** (500 MHz, d_6-acetone).

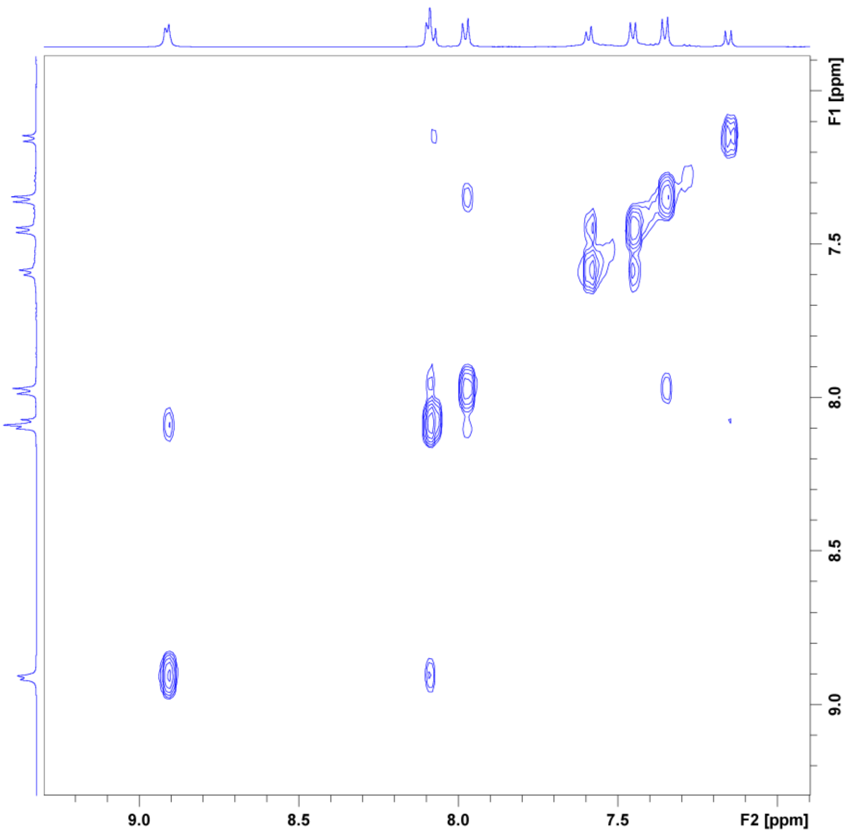

Supplementary Figure 34. Aromatic region of the ^{1}H-^{1}H NOESY spectrum of **H2** (500 MHz, d_6-acetone).

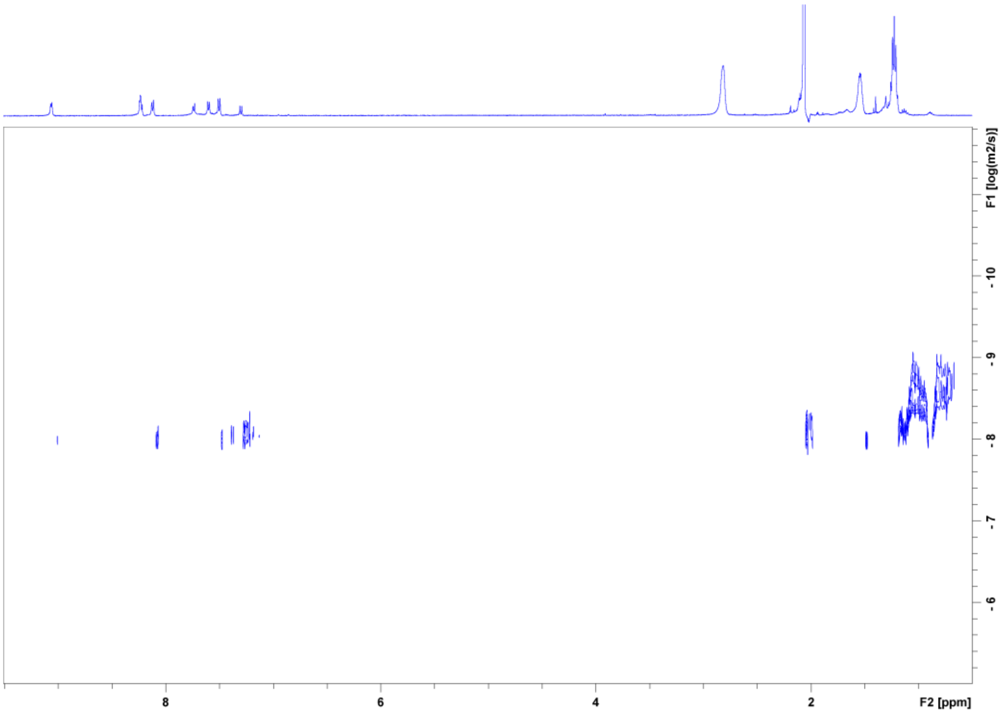

Supplementary Figure 35. ^{1}H DOSY spectrum of **H2** (500 MHz, d_6-acetone).

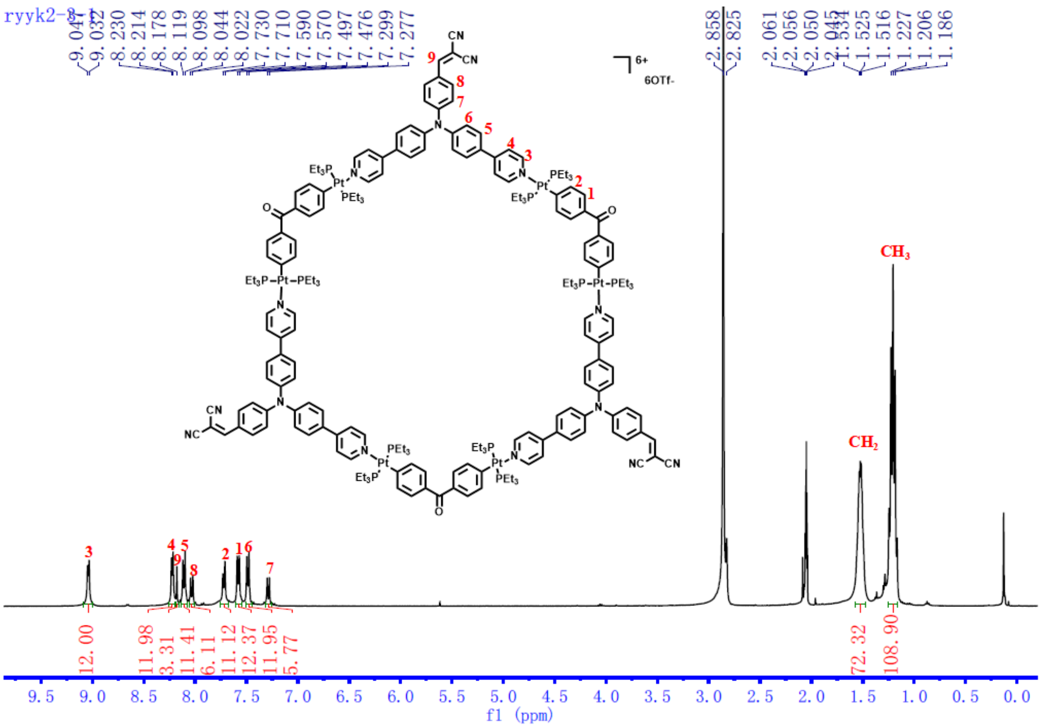

Supplementary Figure 36. ^{1}H NMR spectrum of **H3** (400 MHz, d_6-acetone).

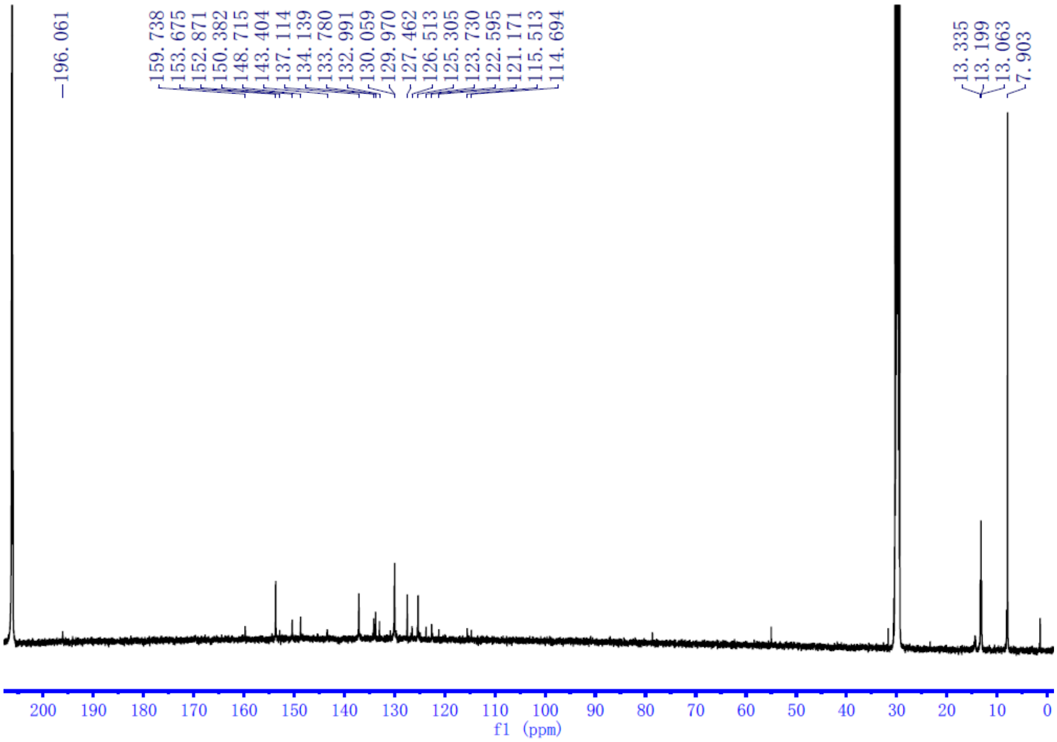

Supplementary Figure 37. ^{13}C NMR spectrum of **H3** (126 MHz, d_6-acetone).

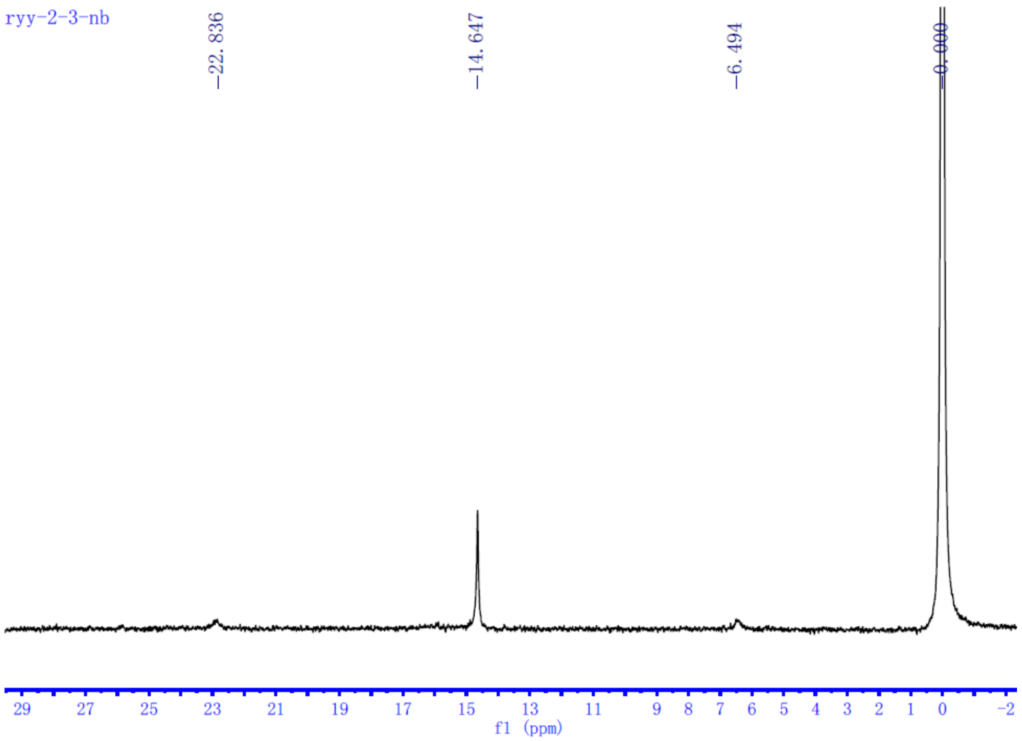

Supplementary Figure 38. ^{31}P NMR spectrum of **H3** (161.9 MHz, d_6-acetone).

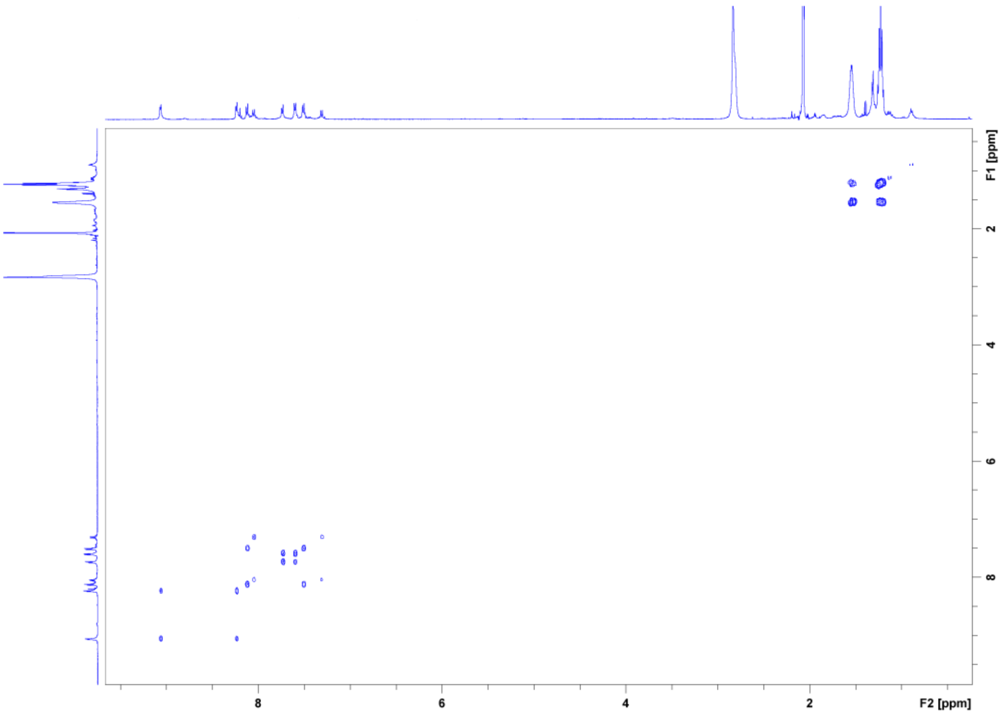

Supplementary Figure 39. ^{1}H-^{1}H COSY spectrum of **H3** (500 MHz, d_6-acetone).

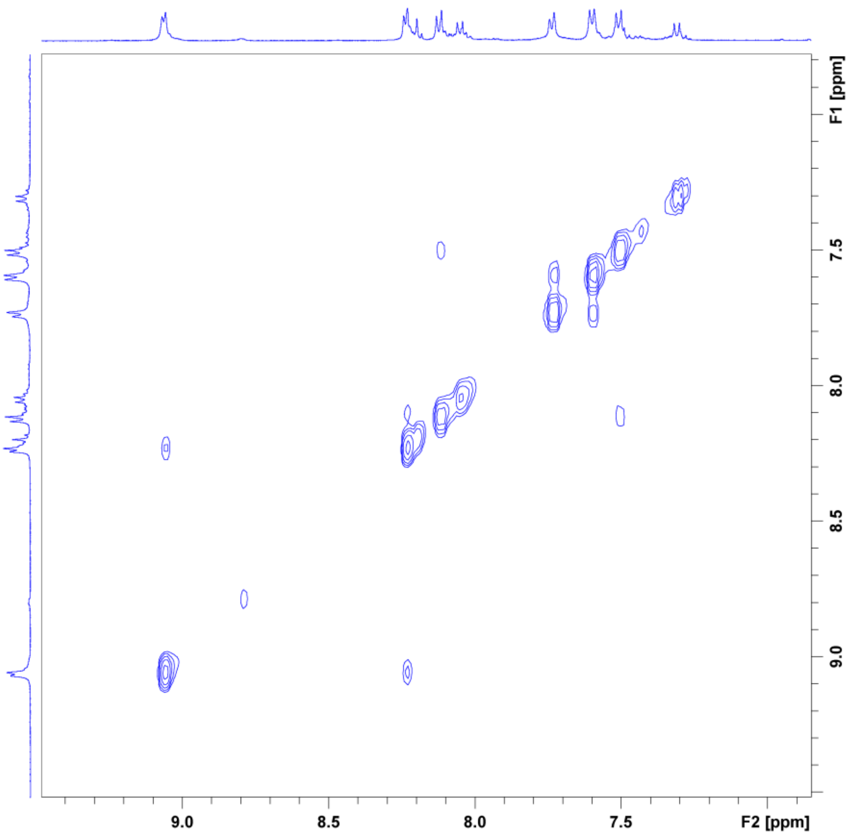

Supplementary Figure 40. Aromatic region of the ^{1}H-^{1}H NOESY spectrum of **H3** (500 MHz, d_6-acetone).

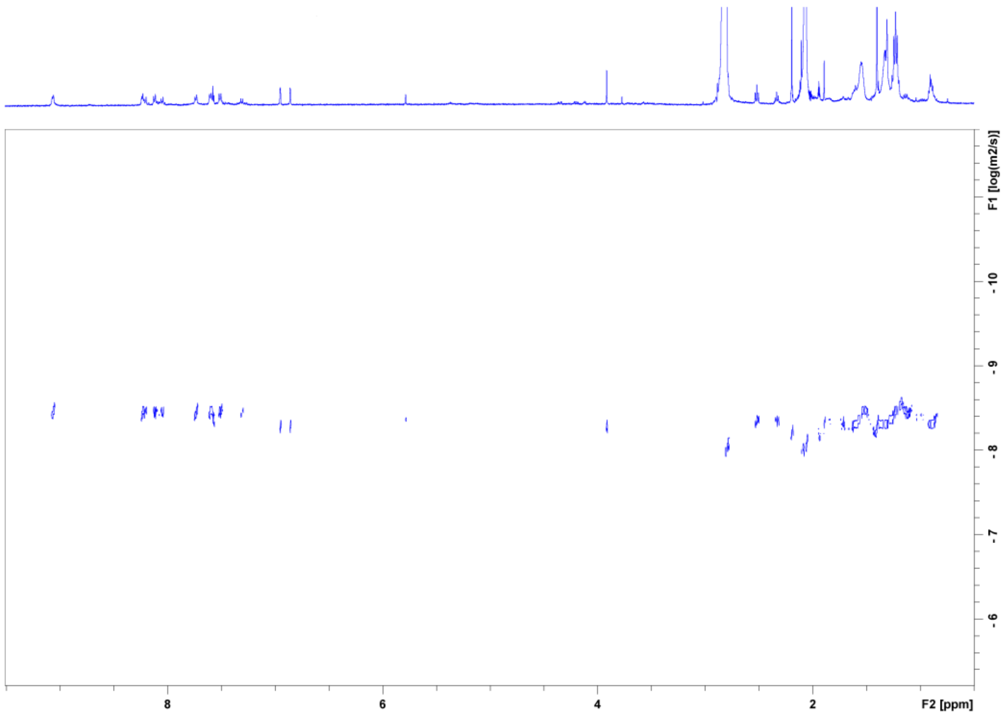

Supplementary Figure 41. ^{1}H DOSY spectrum of **H3** (500 MHz, d_6-acetone).

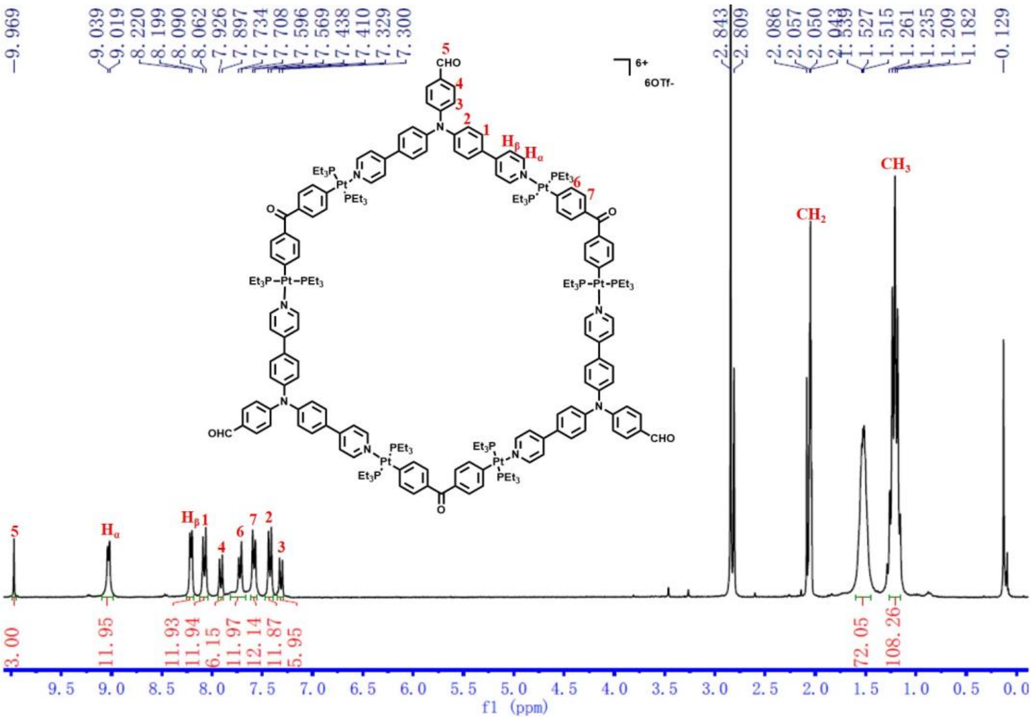

Supplementary Figure 42. ^{1}H NMR spectrum of **H4** (400 MHz, d_6-acetone).

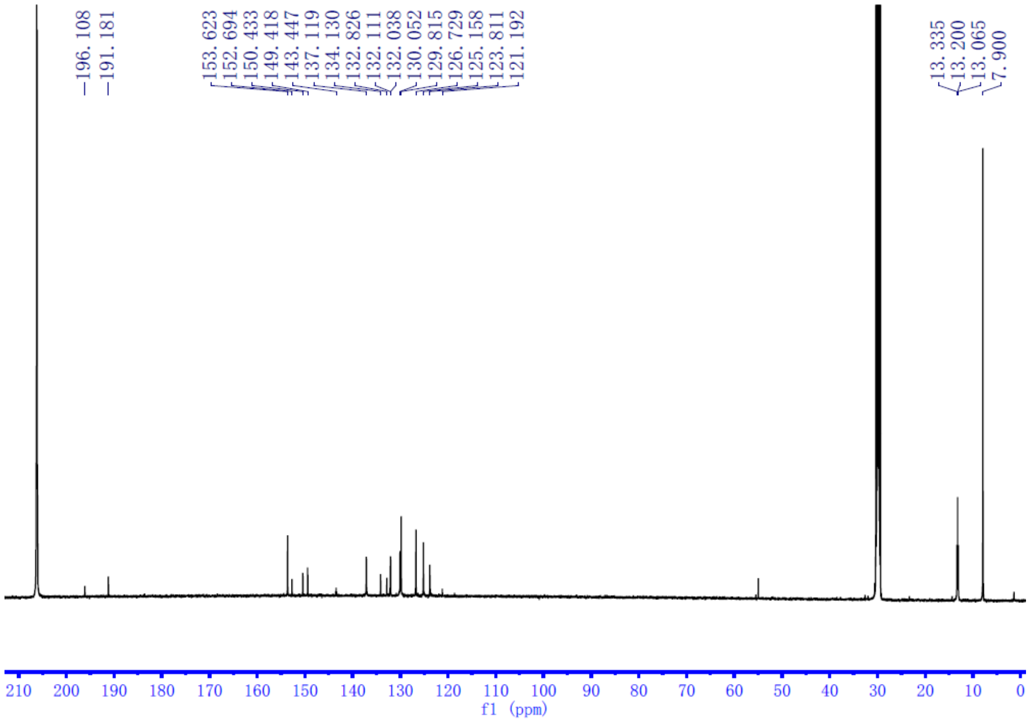

Supplementary Figure 43. ^{13}C NMR spectrum of **H4** (126 MHz, d_6-acetone).

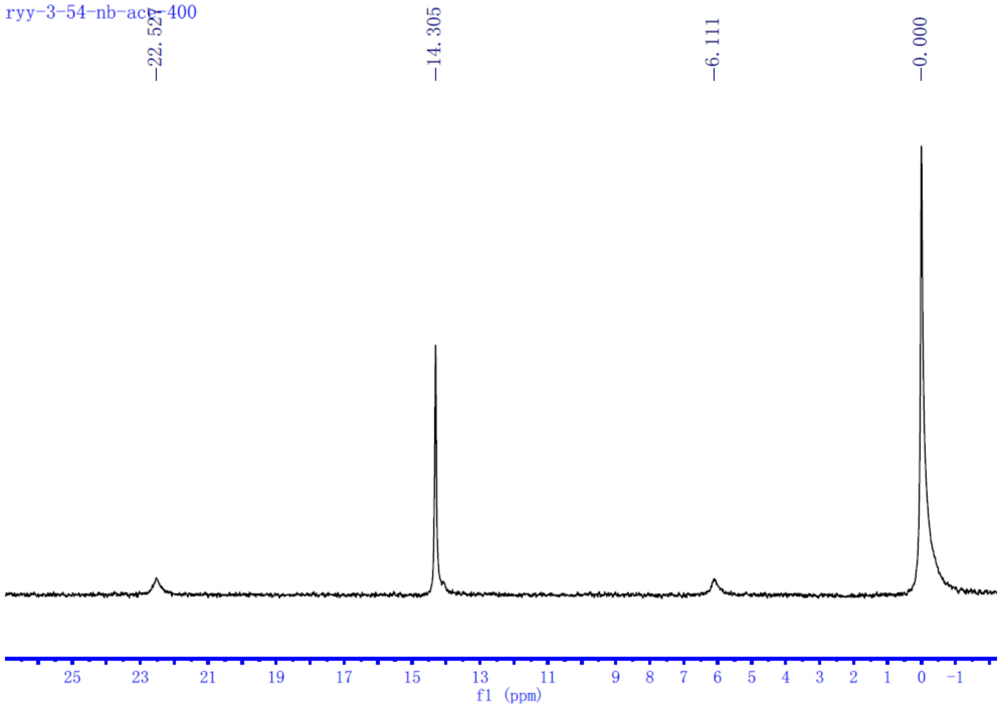

Supplementary Figure 44. ^{31}P NMR spectrum of **H4** (161.9 MHz, d_6-acetone).

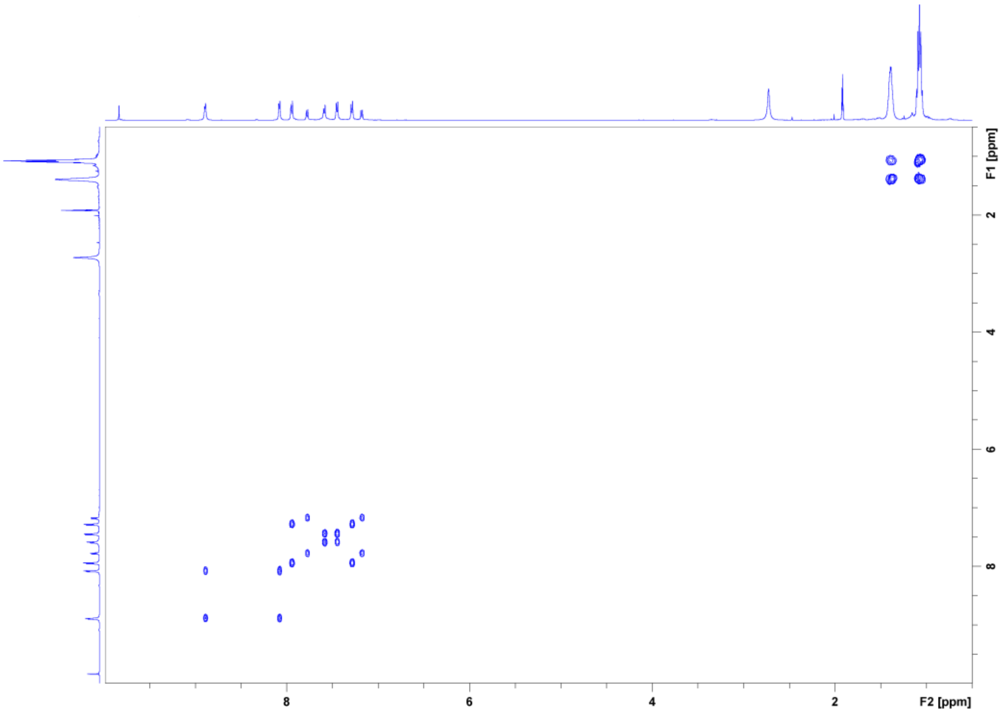

Supplementary Figure 45. ^{1}H-^{1}H COSY spectrum of **H4** (500 MHz, d_6-acetone).

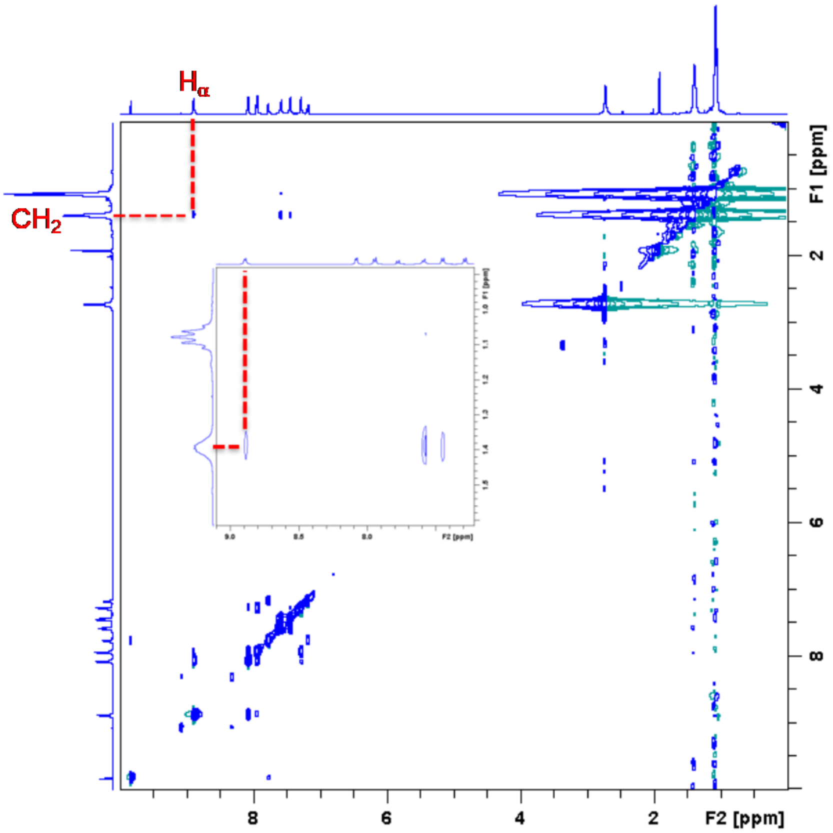

Supplementary Figure 46. ^{1}H-^{1}H NOESY spectrum of **H4** (500 MHz, d_6-acetone).

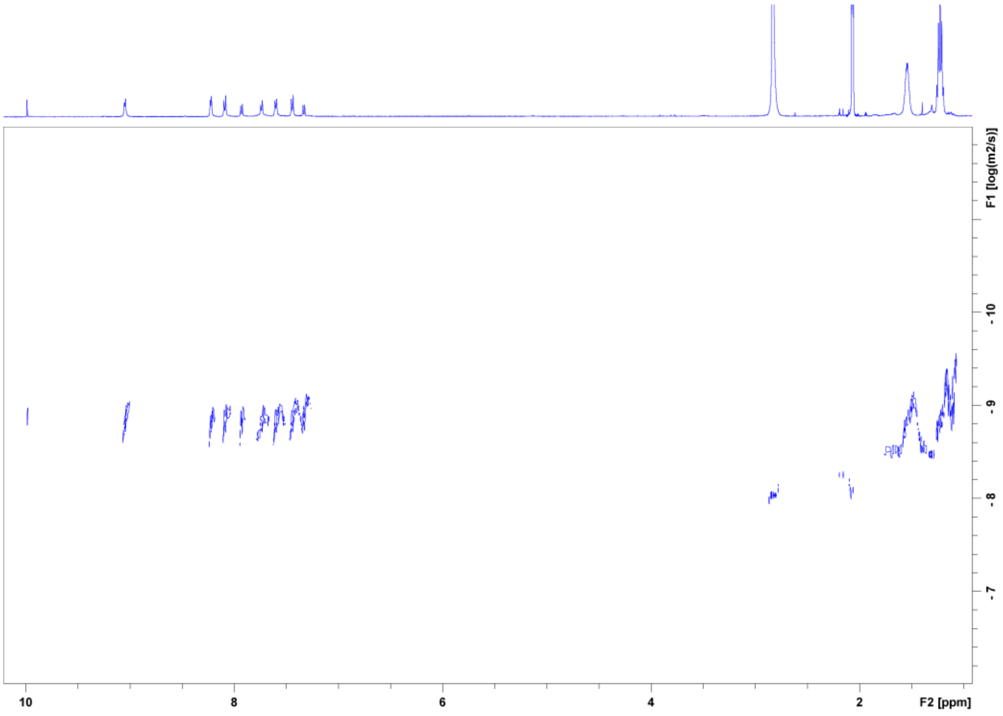

Supplementary Figure 47. ^{1}H DOSY spectrum of **H4** (500 MHz, d_6-acetone).

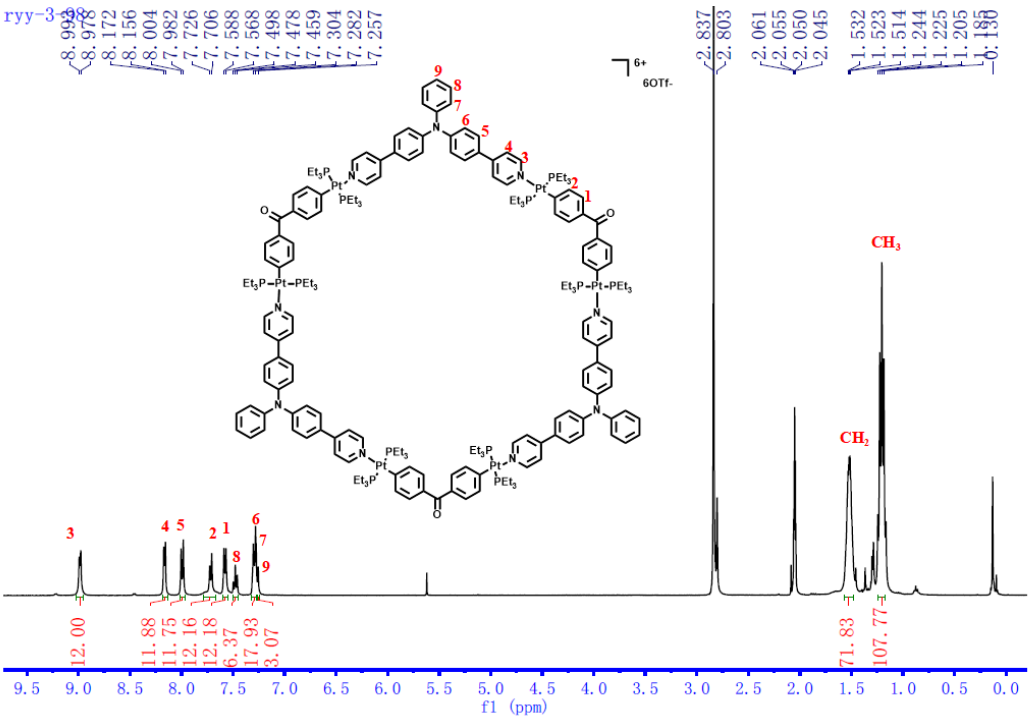

Supplementary Figure 48. ^{1}H NMR spectrum of **H5** (400 MHz, d_6-acetone).

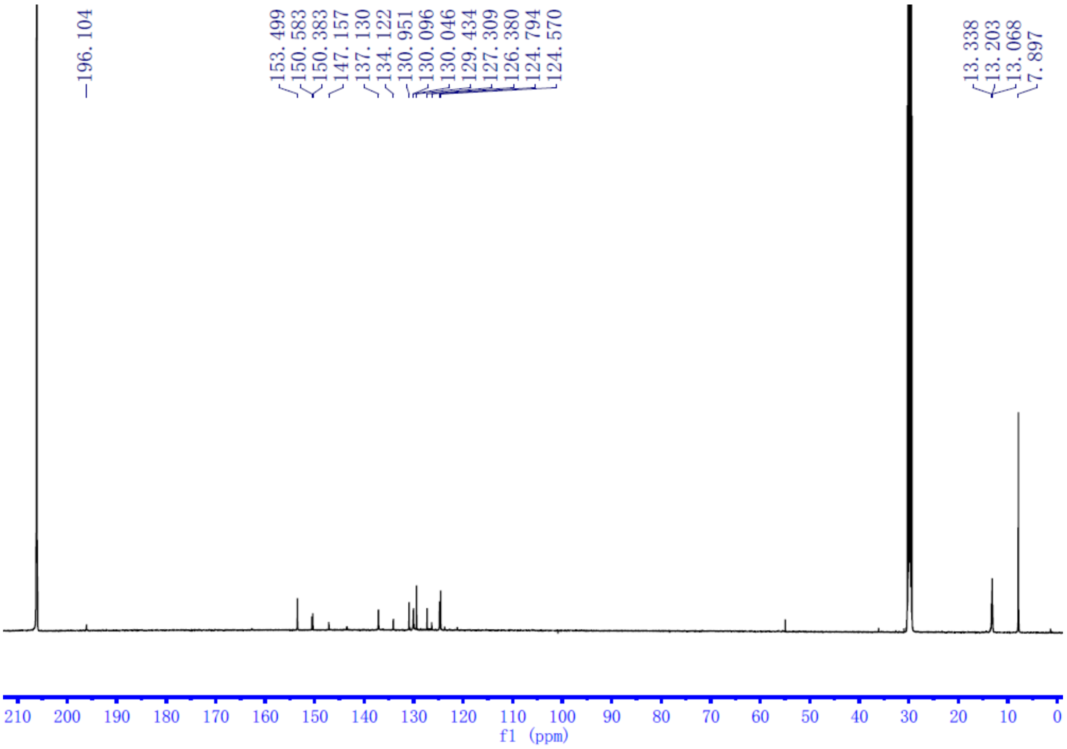

Supplementary Figure 49. ^{13}C NMR spectrum of **H5** (126 MHz, d_6-acetone).

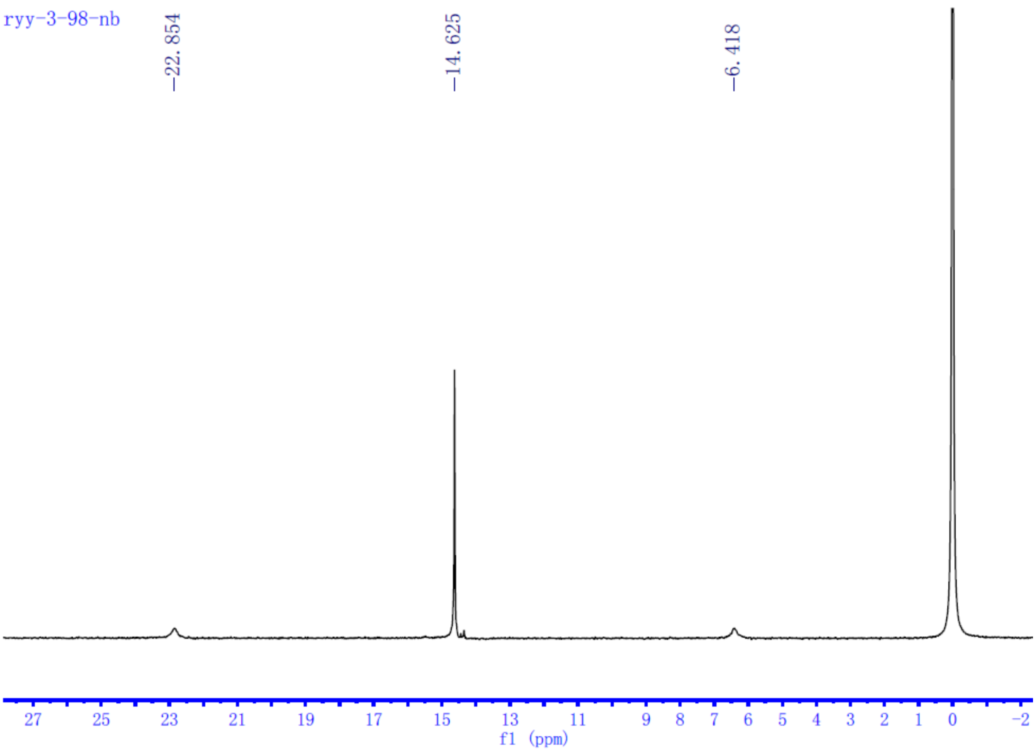

Supplementary Figure 50. ^{31}P NMR spectrum of **H5** (161.9 MHz, d_6-acetone).

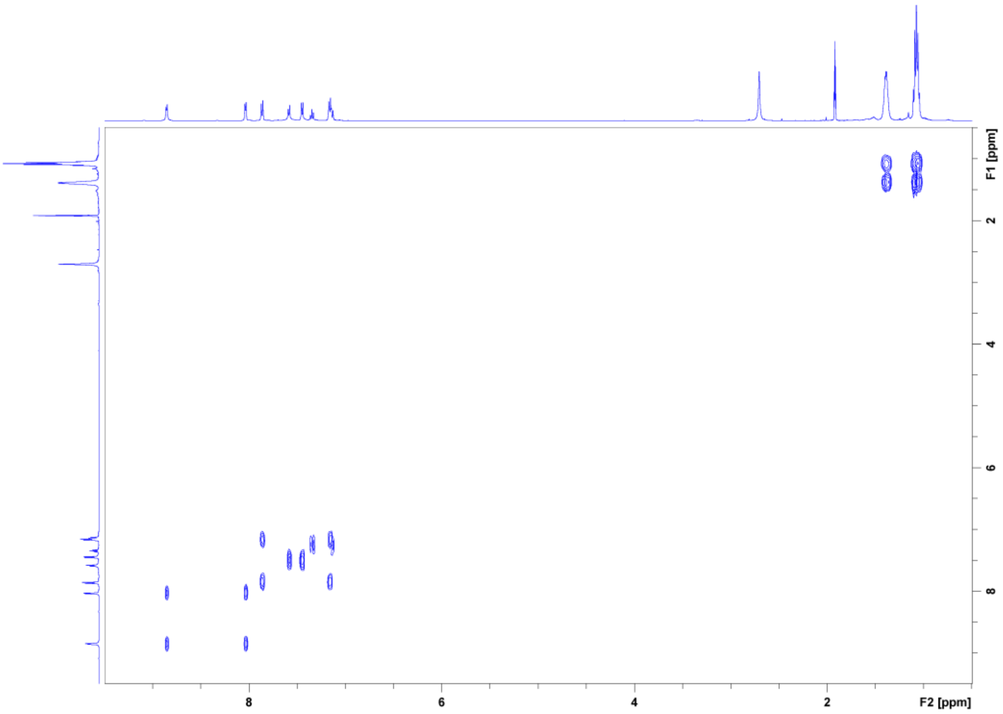

Supplementary Figure 51. ^{1}H-^{1}H COSY spectrum of **H5** (500 MHz, d_6-acetone).

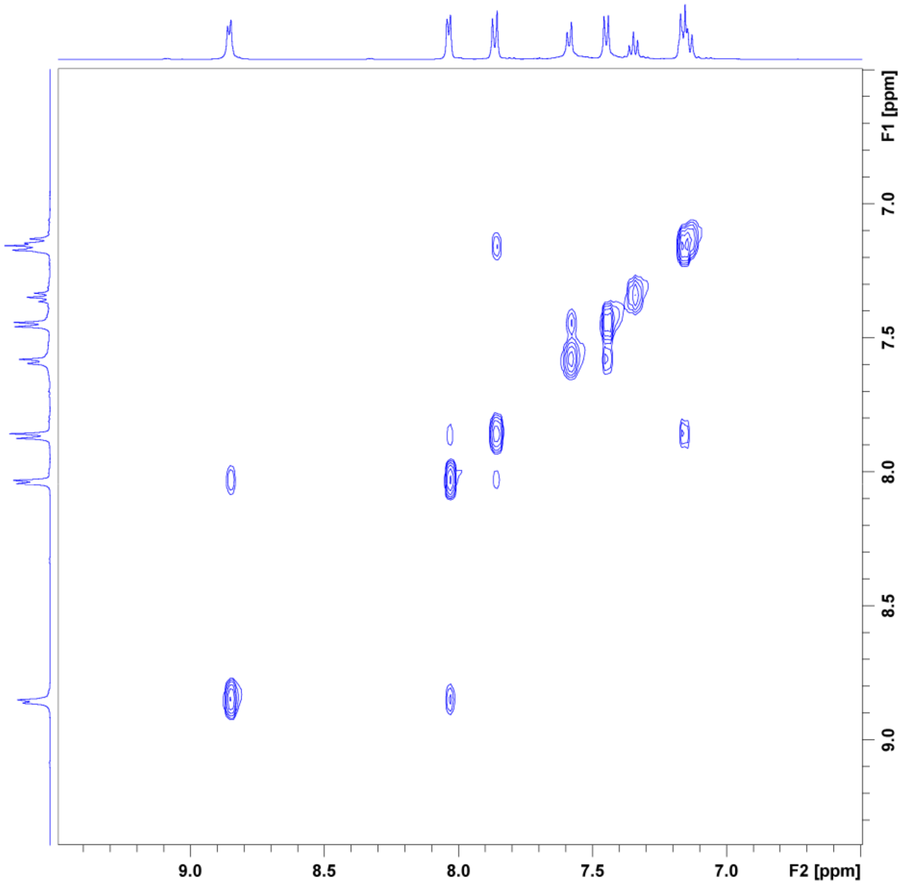

Supplementary Figure 52. Aromatic region of the ^{1}H-^{1}H NOESY spectrum of **H5** (500 MHz, d_6-acetone).

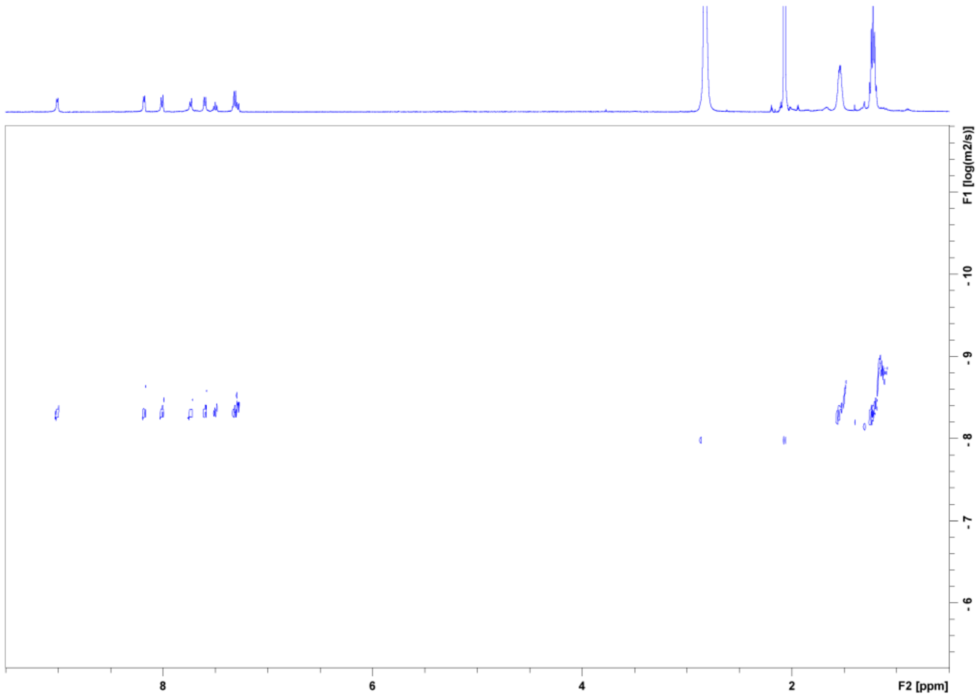

Supplementary Figure 53. ^{1}H DOSY spectrum of **H5** (500 MHz, d_6-acetone).

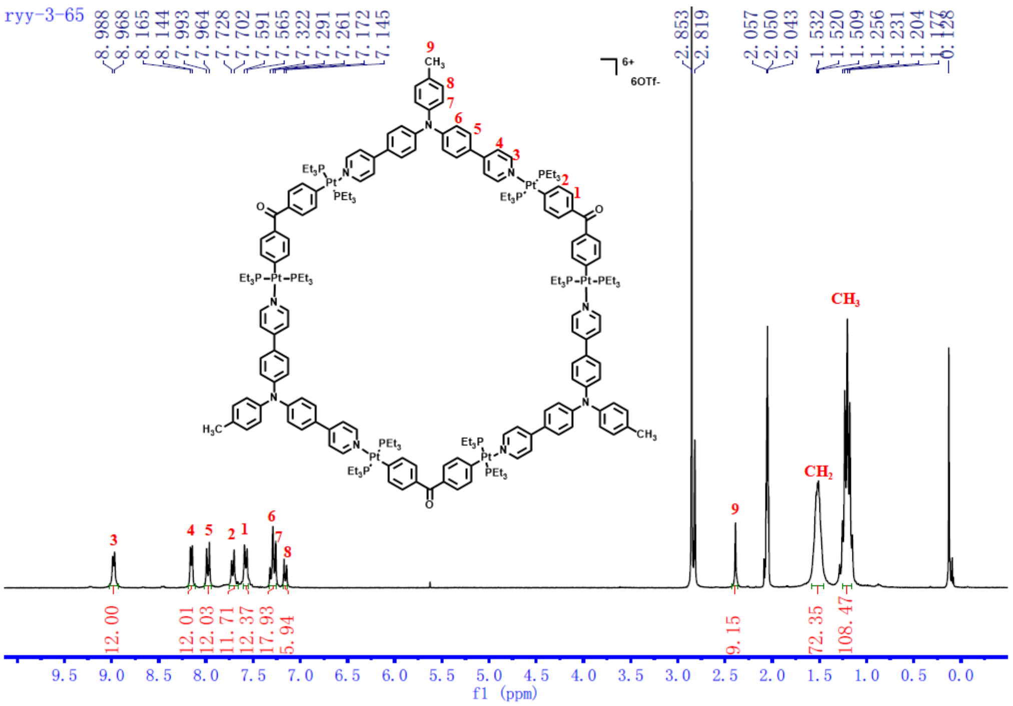

Supplementary Figure 54. ^{1}H NMR spectrum of **H6** (400 MHz, d_6-acetone).

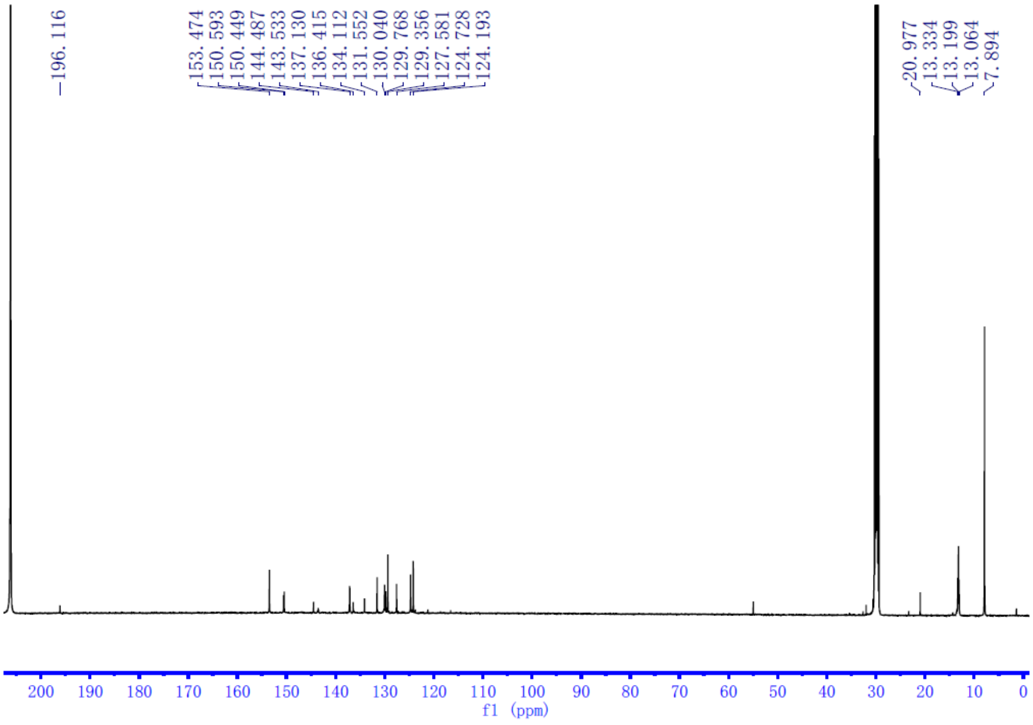

Supplementary Figure 55. ^{13}C NMR spectrum of **H6** (126 MHz, d_6-acetone).

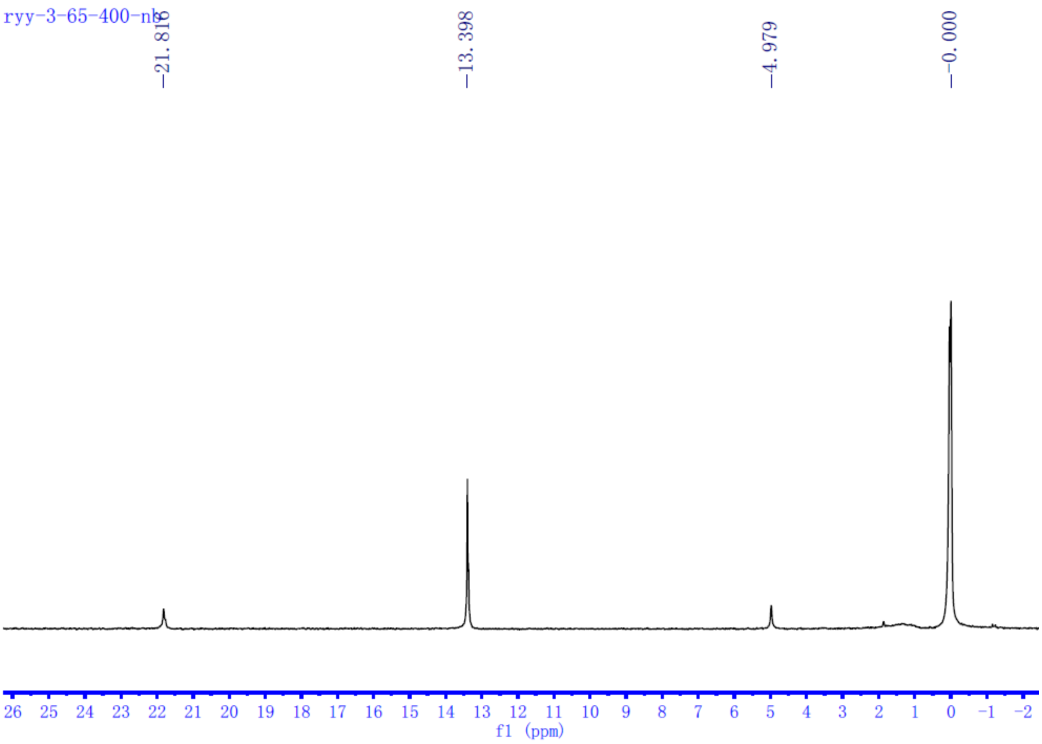

Supplementary Figure 56. ^{31}P NMR spectrum of **H6** (161.9 MHz, d_6-acetone).

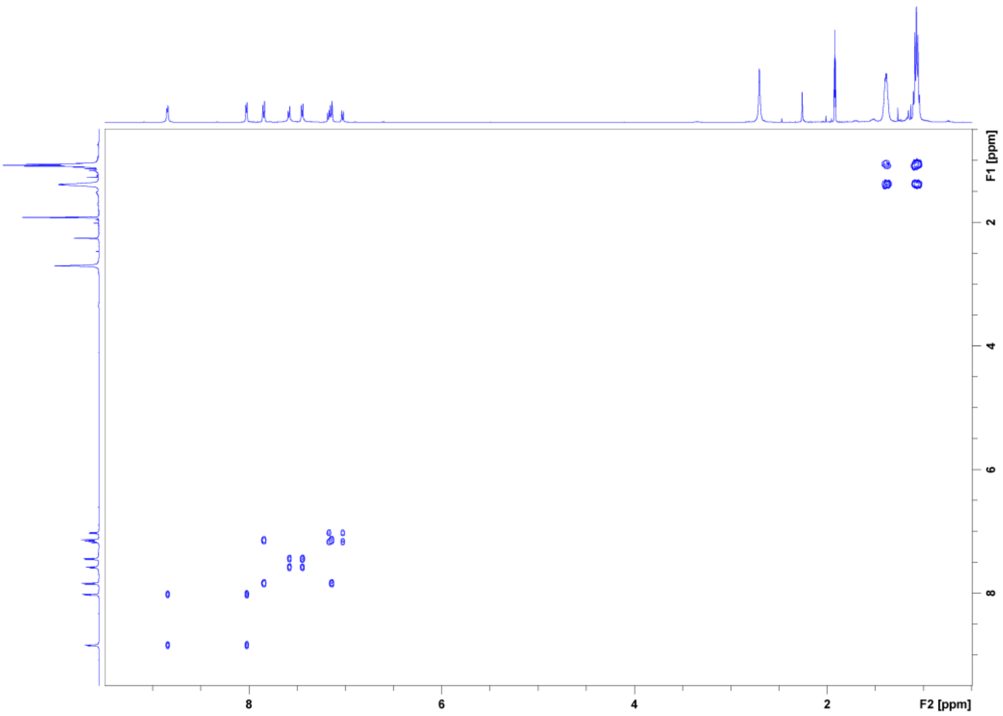

Supplementary Figure 57. ^{1}H-^{1}H COSY spectrum of **H6** (500 MHz, d_6-acetone).

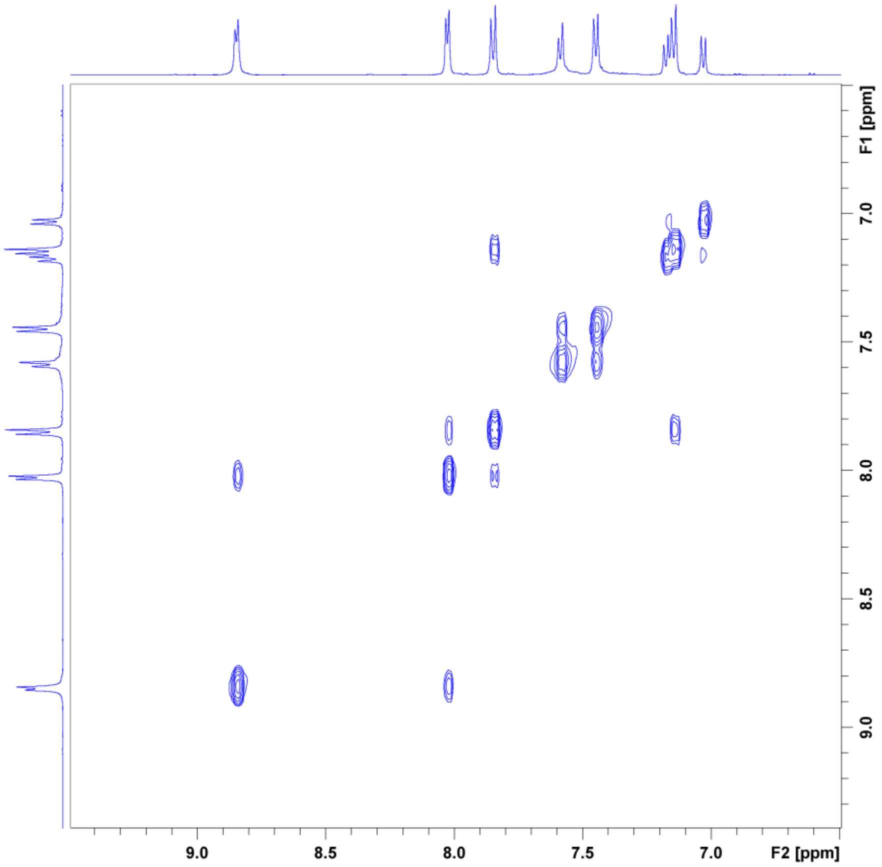

Supplementary Figure 58. Aromatic region of the ¹H-¹H NOESY spectrum of **H6** (500 MHz, *d*₆-acetone).

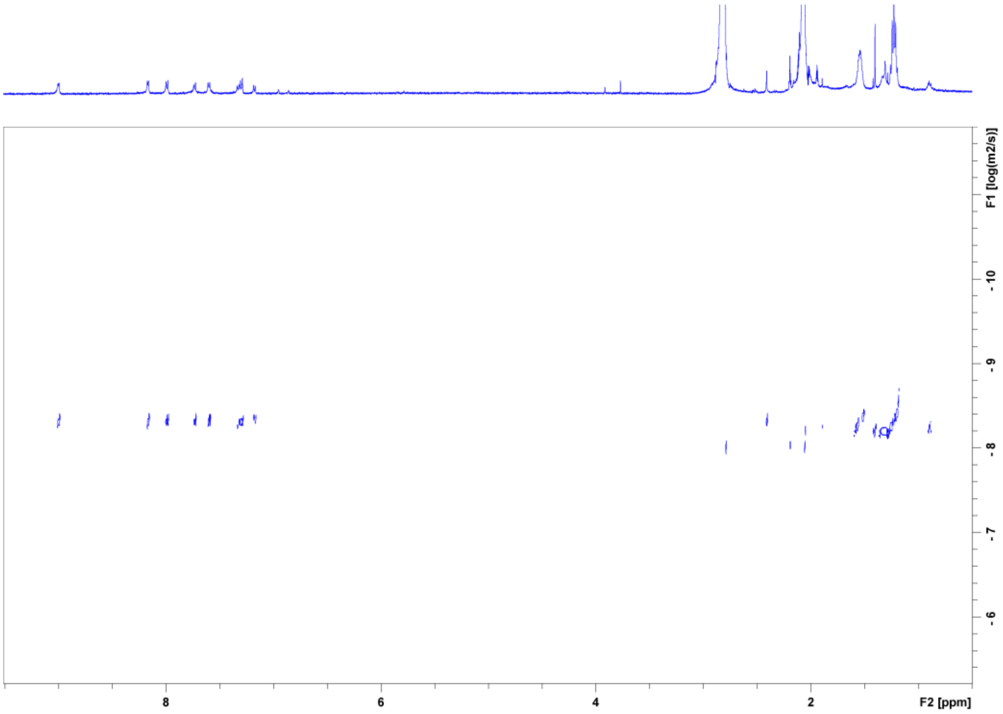

Supplementary Figure 59. ¹H DOSY spectrum of **H6** (500 MHz, *d*₆-acetone).

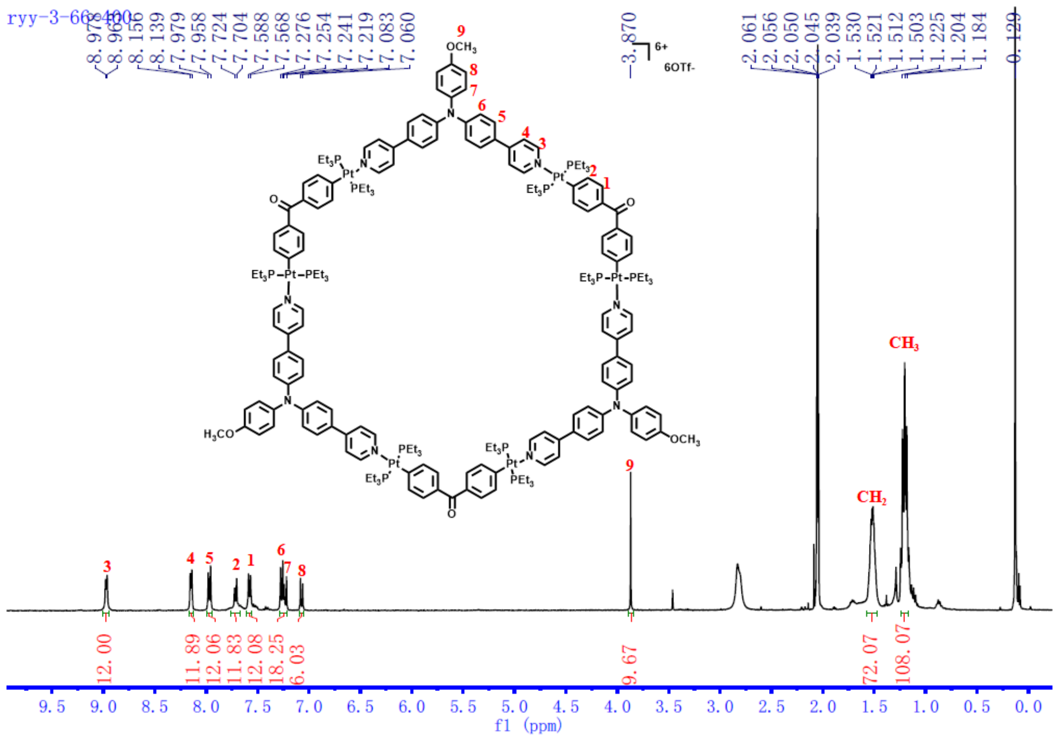

Supplementary Figure 60. ^{1}H NMR spectrum of **H7** (400 MHz, d_6-acetone).

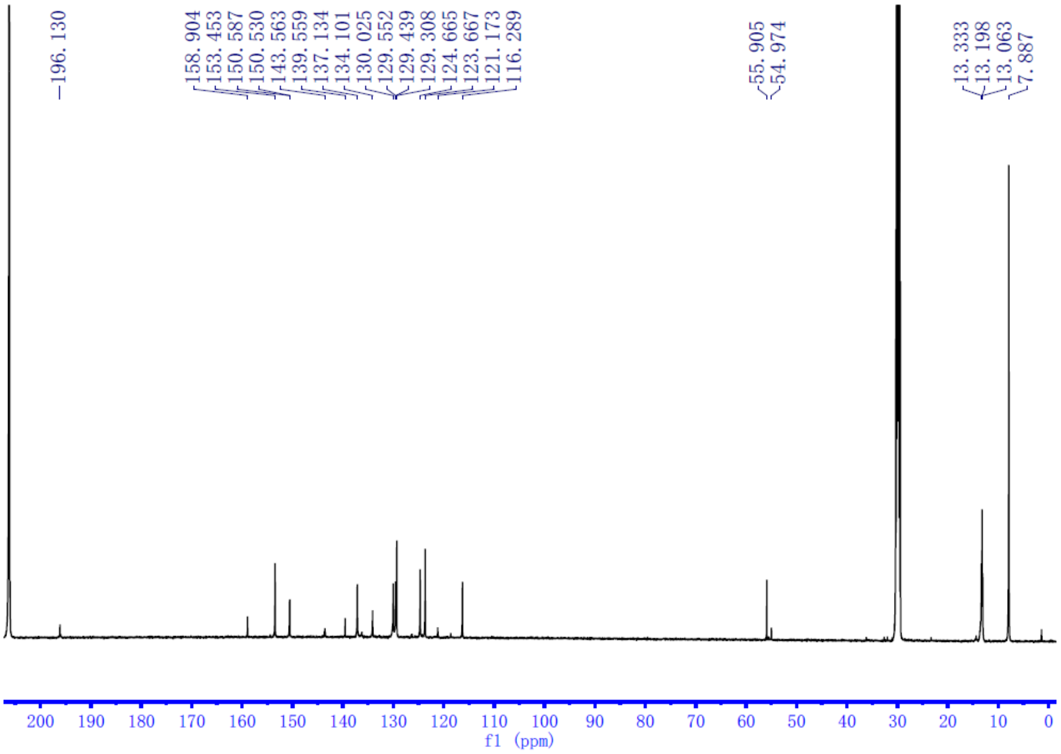

Supplementary Figure 61. ^{13}C NMR spectrum of **H7** (126 MHz, d_6-acetone).

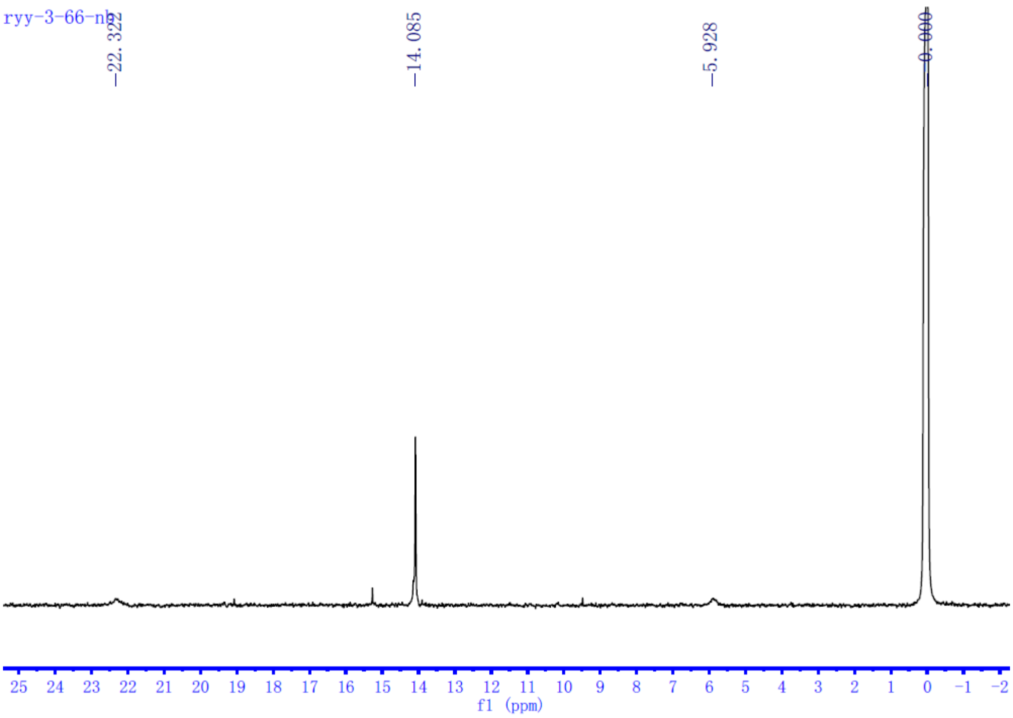

Supplementary Figure 62. ^{31}P NMR spectrum of **H7** (161.9 MHz, d_6-acetone).

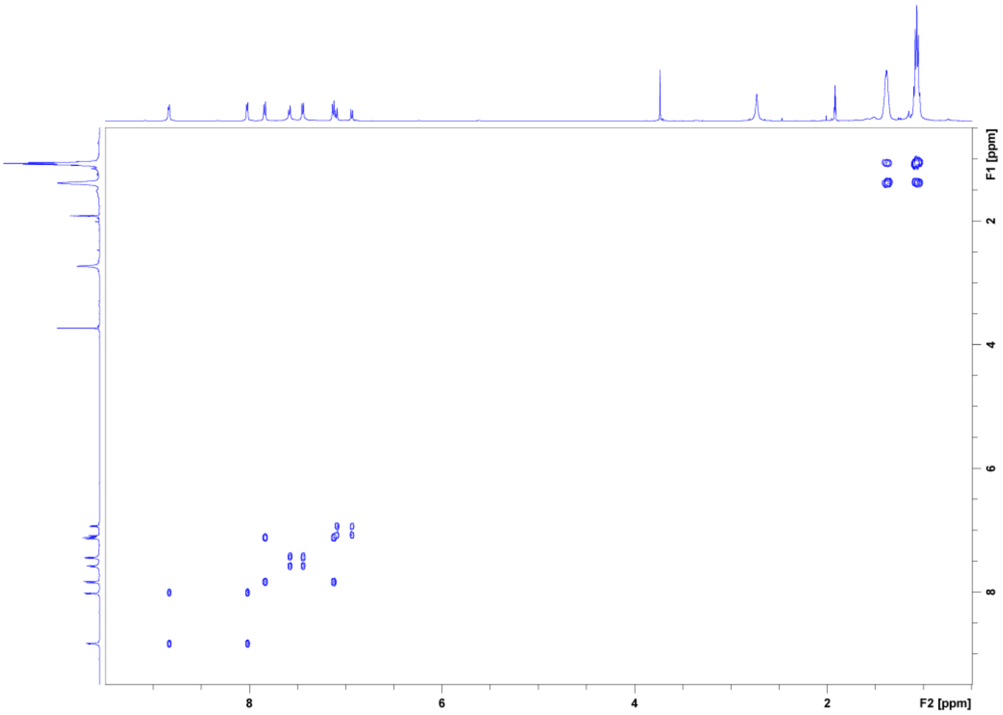

Supplementary Figure 63. ^{1}H-^{1}H COSY spectrum of **H7** (500 MHz, d_6-acetone).

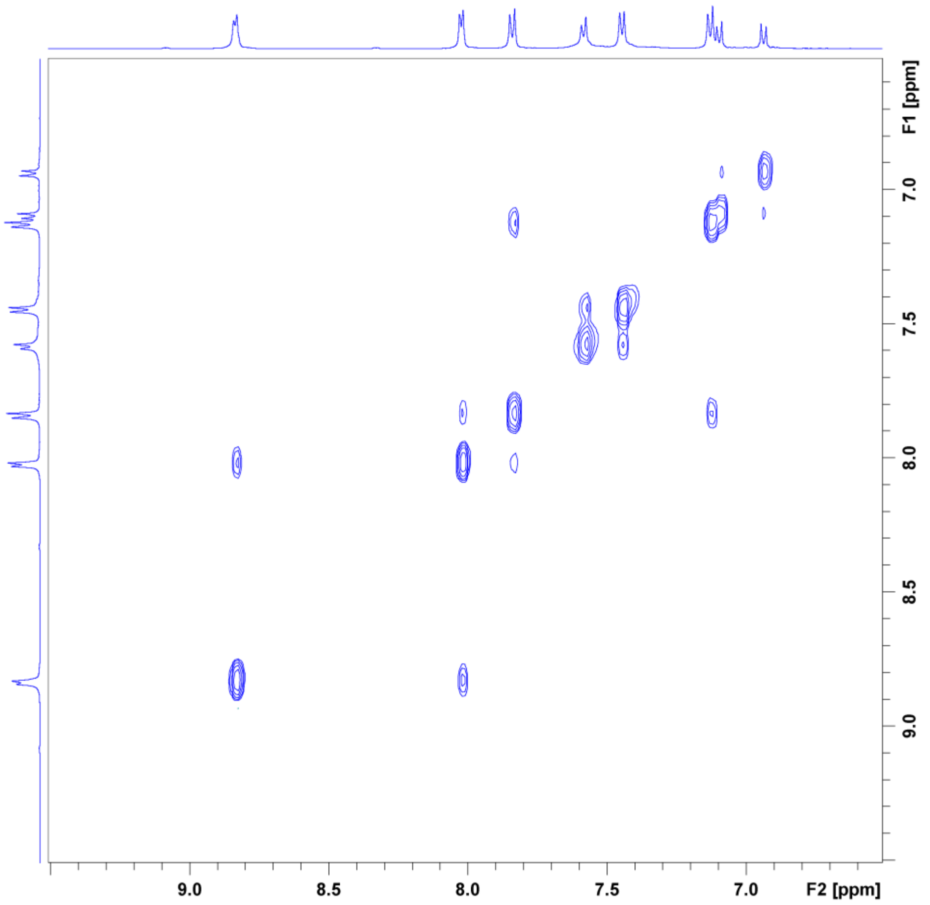

Supplementary Figure 64. Aromatic region of the ^{1}H-^{1}H NOESY spectrum of **H7** (500 MHz, d_6-acetone).

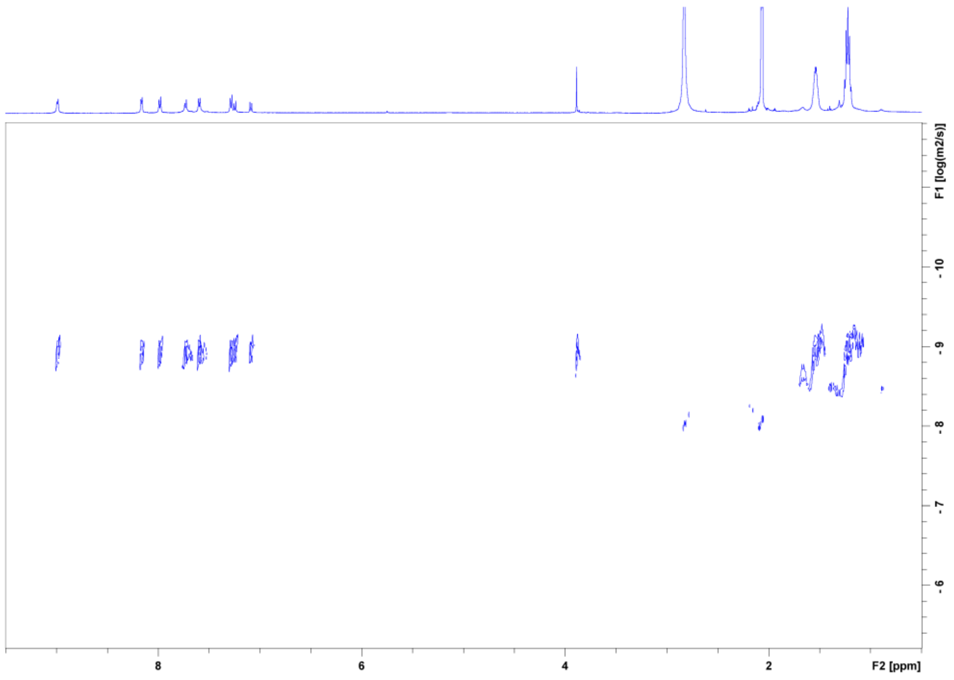

Supplementary Figure 65. ^{1}H DOSY spectrum of **H7** (500 MHz, d_6-acetone).

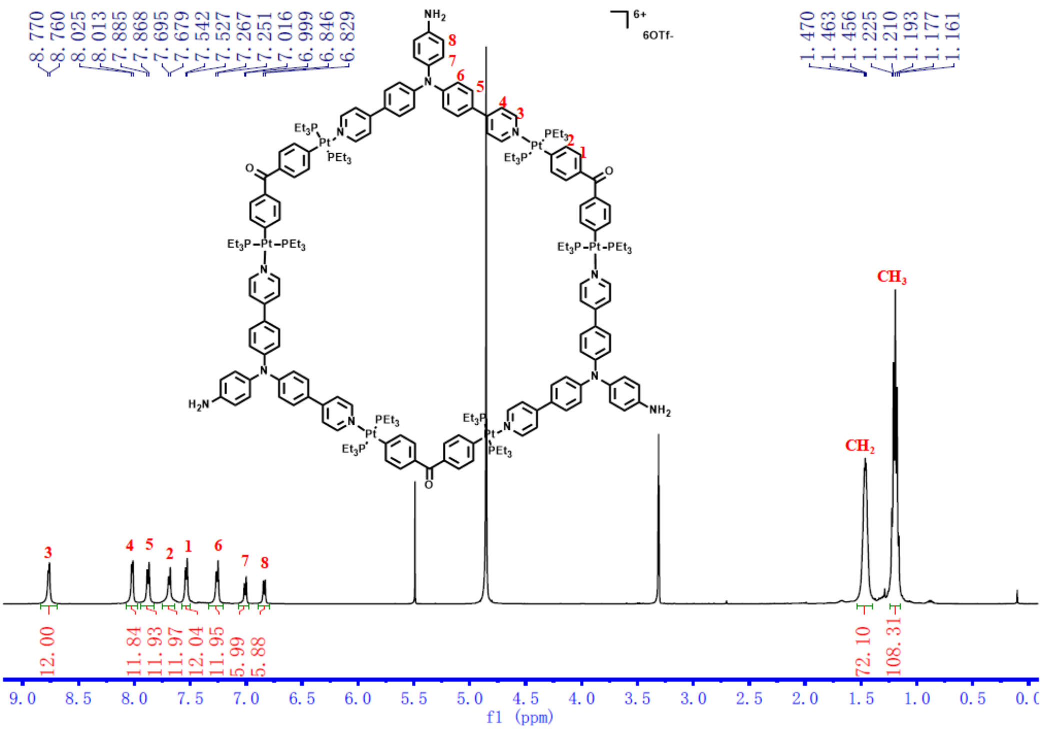

Supplementary Figure 66. ^{1}H NMR spectrum of **H8** (500 MHz, MeOD).

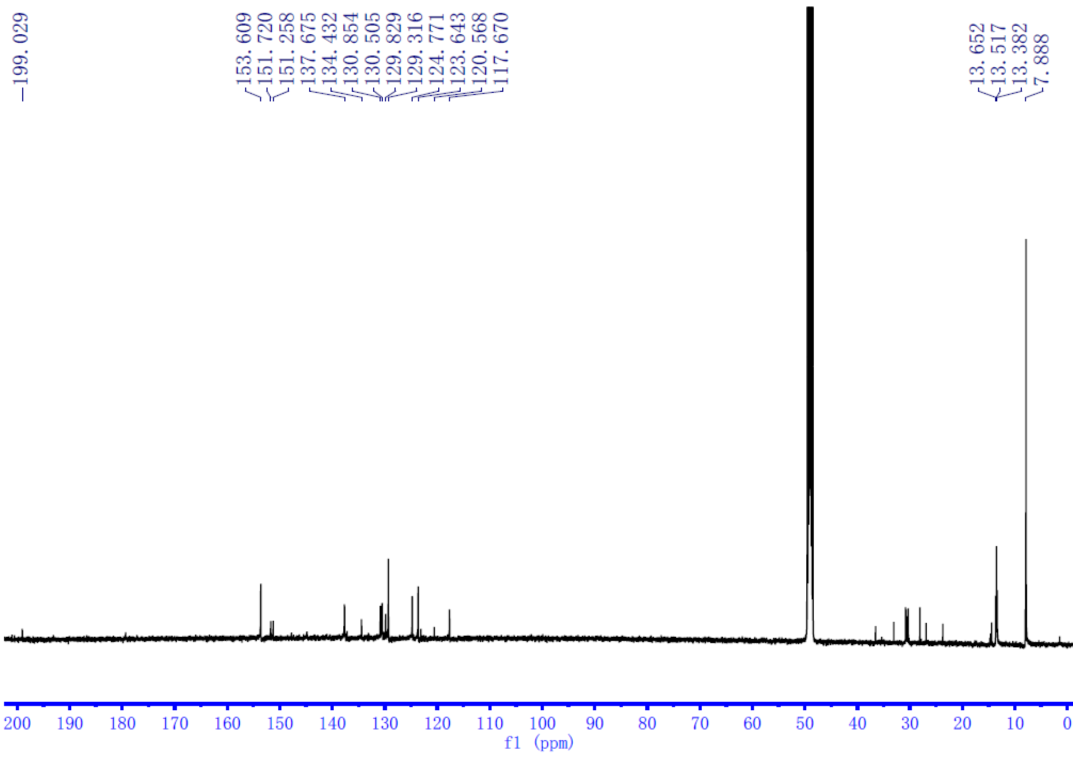

Supplementary Figure 67. ^{13}C NMR spectrum of **H8** (126 MHz, MeOD).

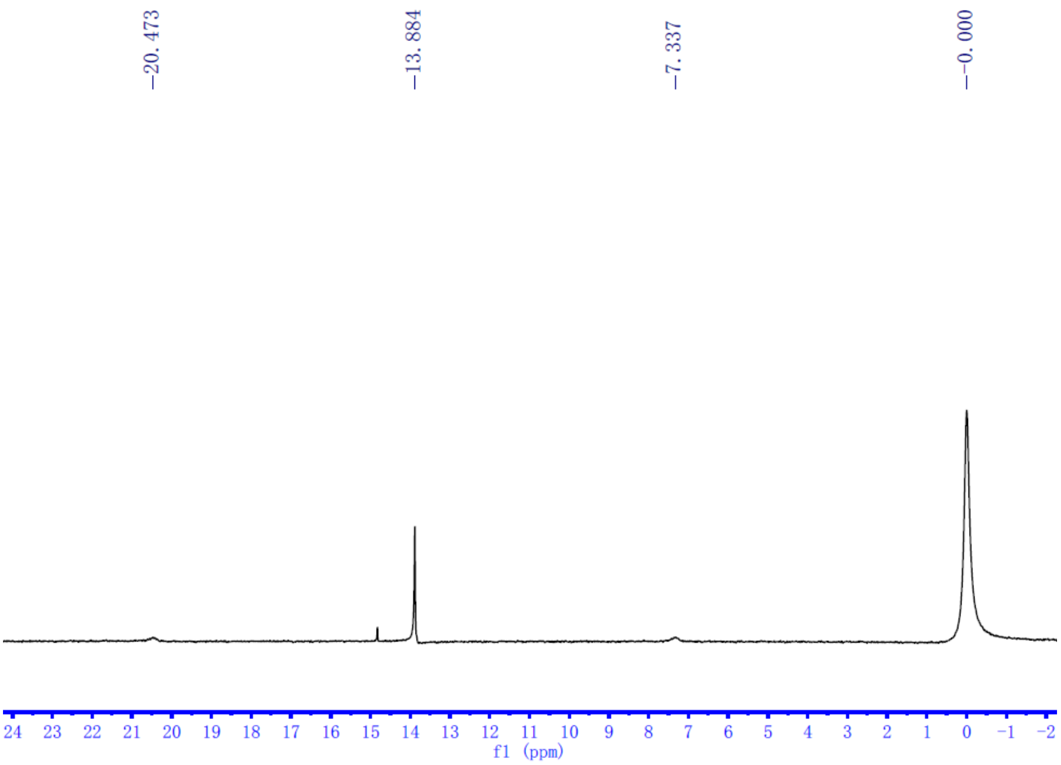

Supplementary Figure 68. ^{31}P NMR spectrum of **H8** (161.9 MHz, MeOD).

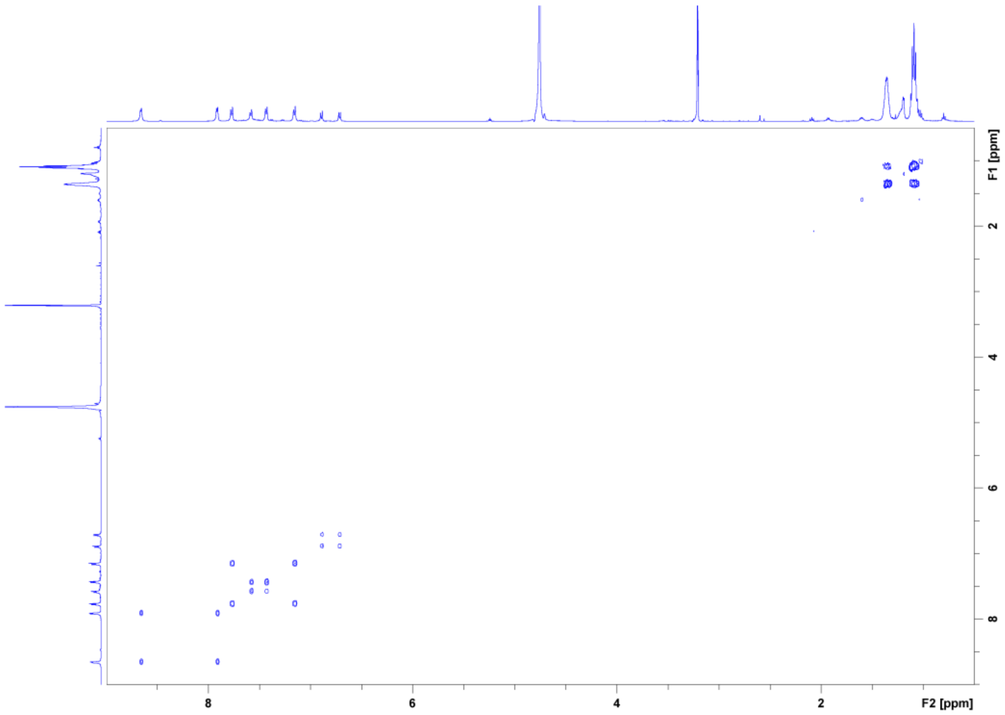

Supplementary Figure 69. ^{1}H-^{1}H COSY spectrum of **H8** (500 MHz, MeOD).

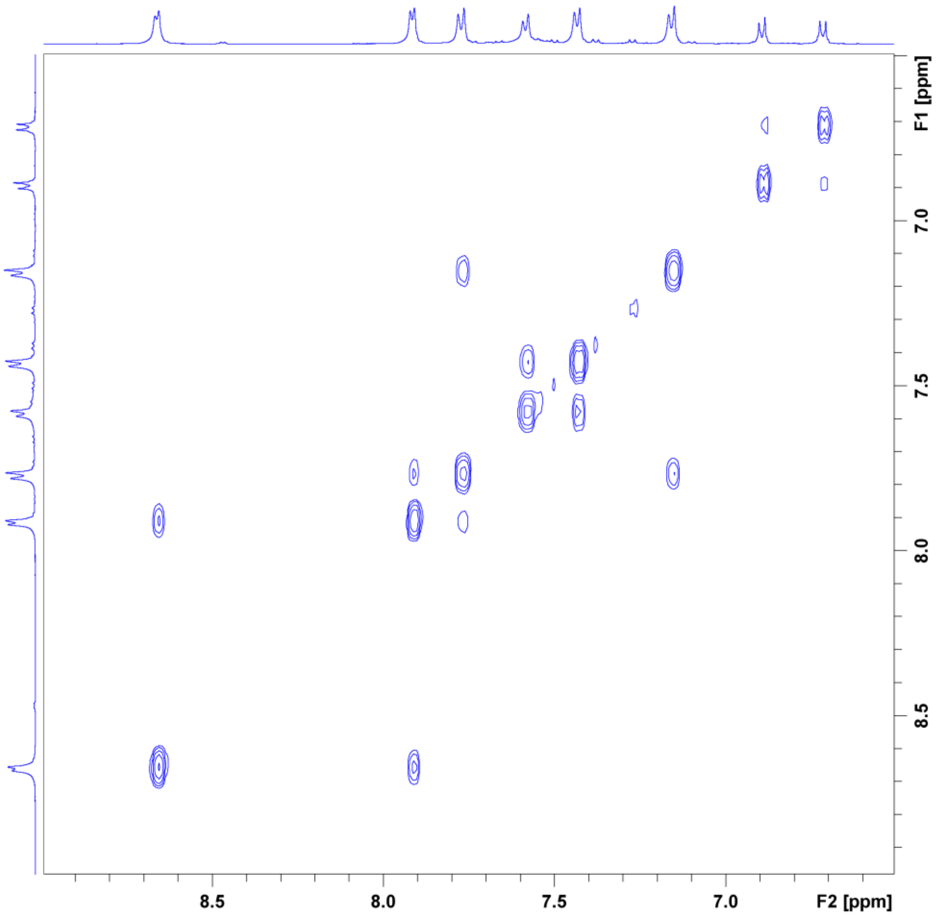

Supplementary Figure 70. Aromatic region of the ^{1}H-^{1}H NOESY spectrum of **H8** (500 MHz, MeOD).

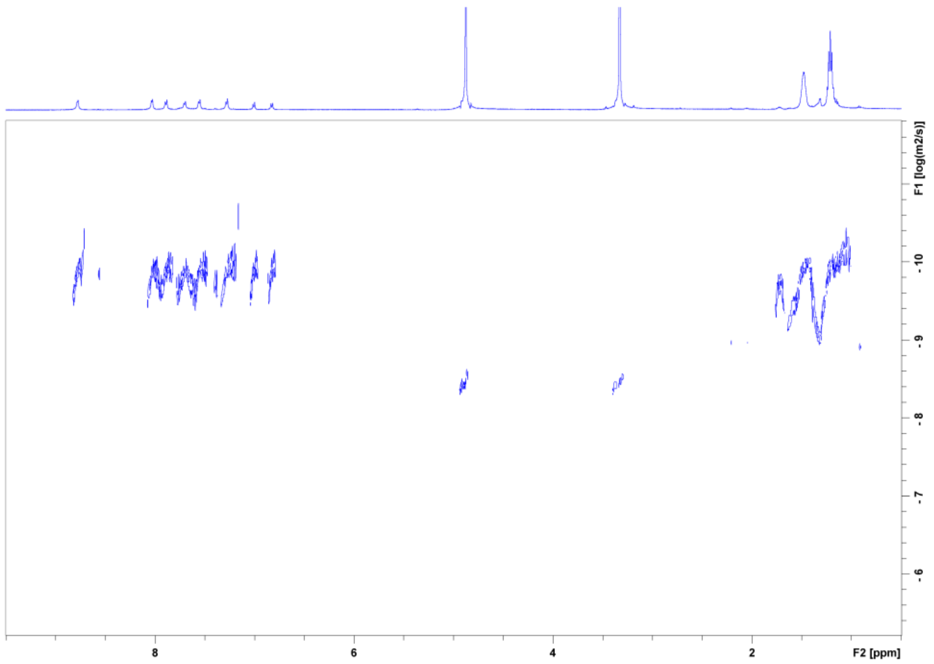

Supplementary Figure 71. ^{1}H DOSY spectrum of **H8** (500 MHz, MeOD).

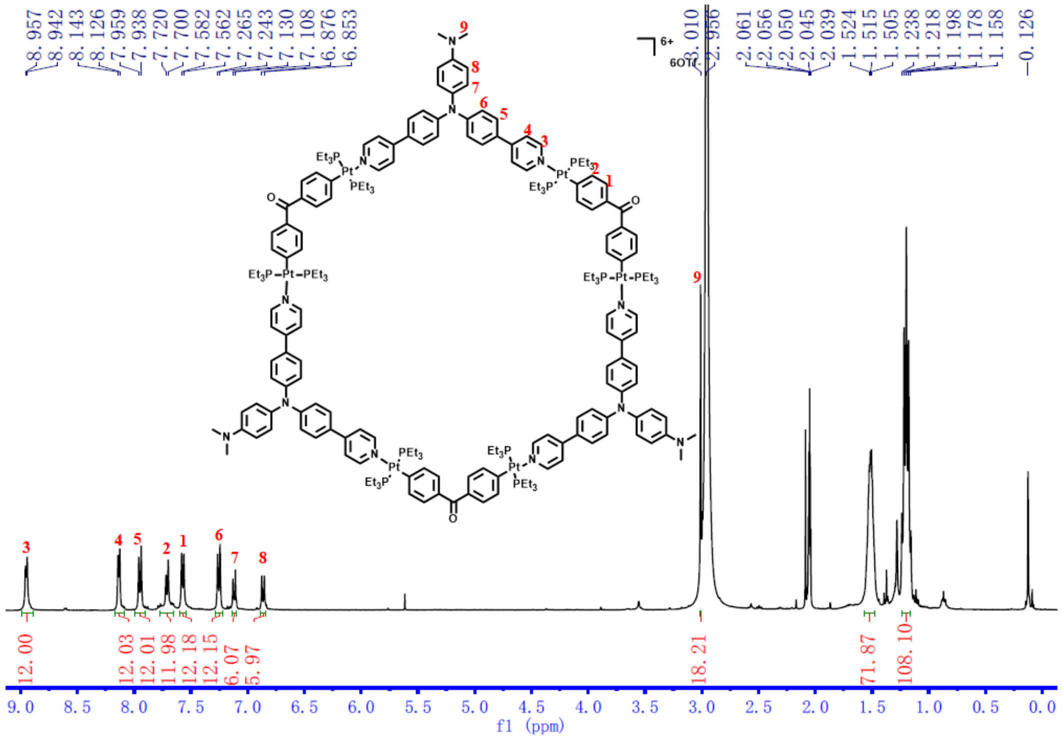

Supplementary Figure 72. ^{1}H NMR spectrum of **H9** (400 MHz, d_6-acetone).

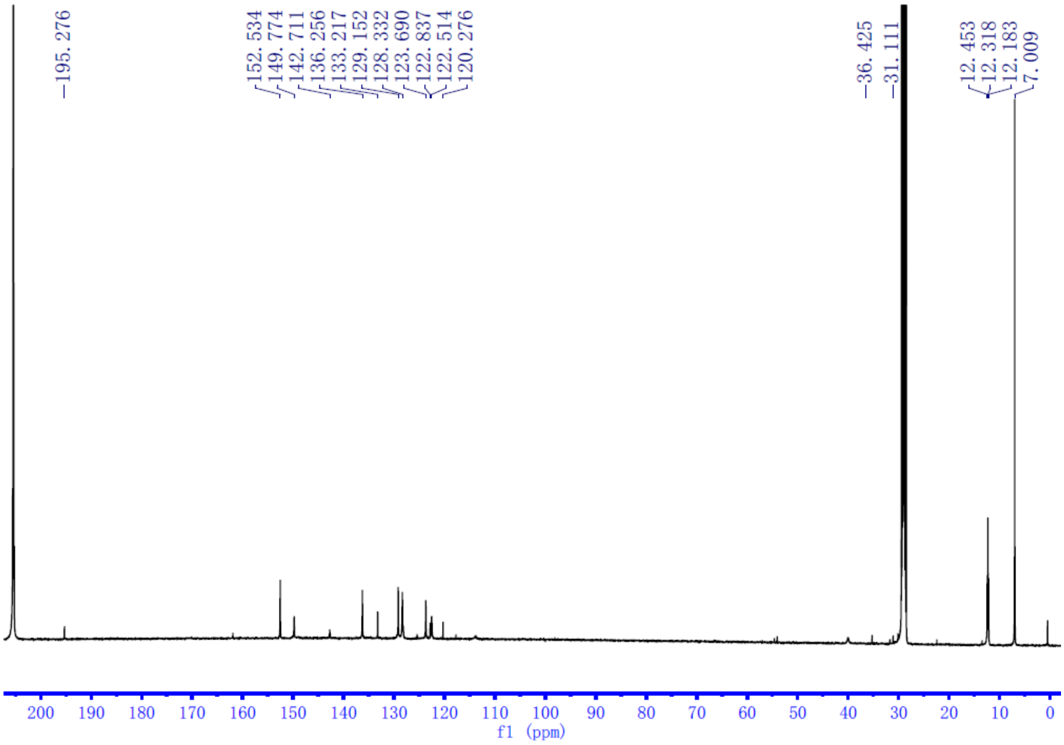

Supplementary Figure 73. ^{13}C NMR spectrum of **H9** (126 MHz, d_6-acetone).

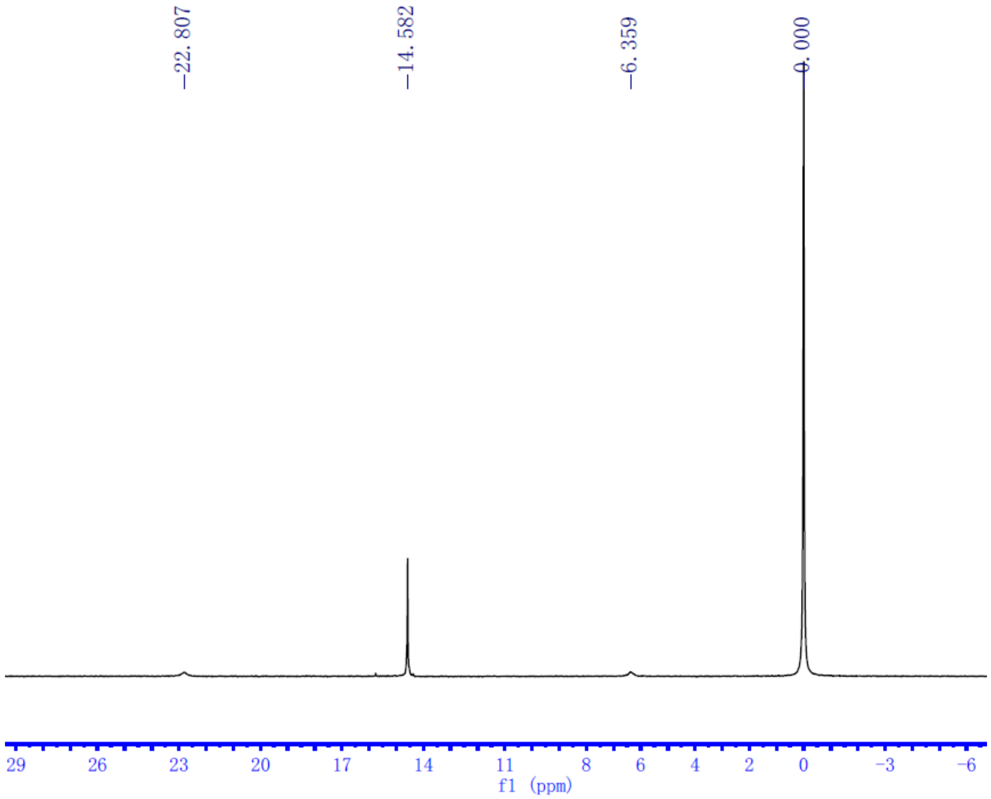

Supplementary Figure 74. ^{31}P NMR spectrum of **H9** (161.9 MHz, d_6-acetone).

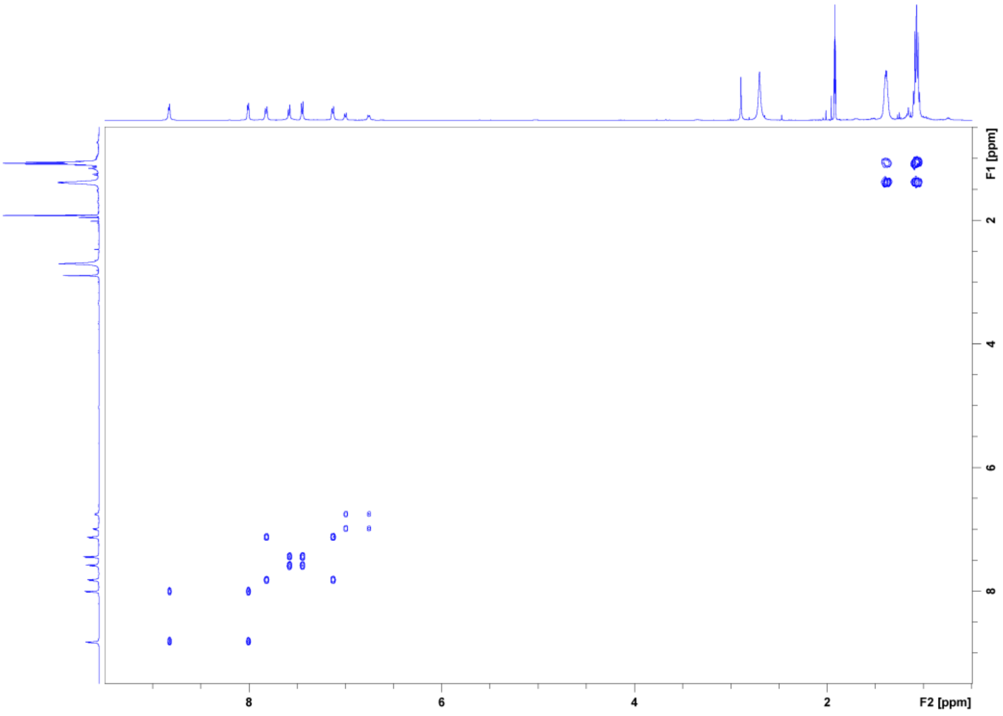

Supplementary Figure 75. ^{1}H-^{1}H COSY spectrum of **H9** (500 MHz, d_6-acetone).

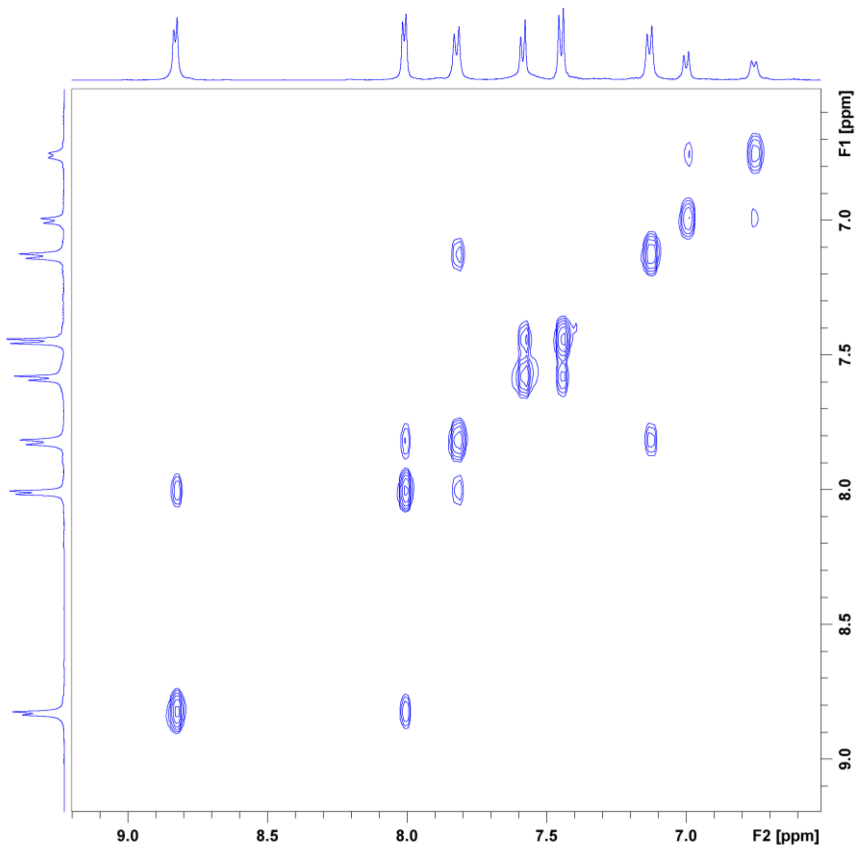

Supplementary Figure 76. Aromatic region of the ^{1}H-^{1}H NOESY spectrum of **H9** (500 MHz, d_6-acetone).

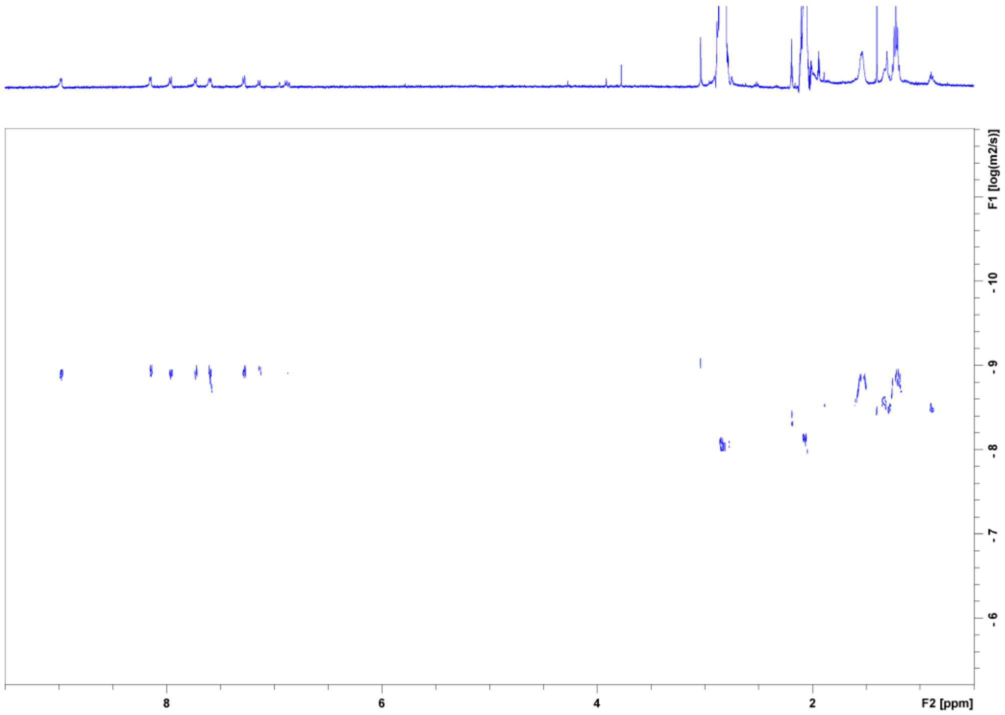

Supplementary Figure 77. ^{1}H DOSY spectrum of **H9** (500 MHz, d_6-acetone).

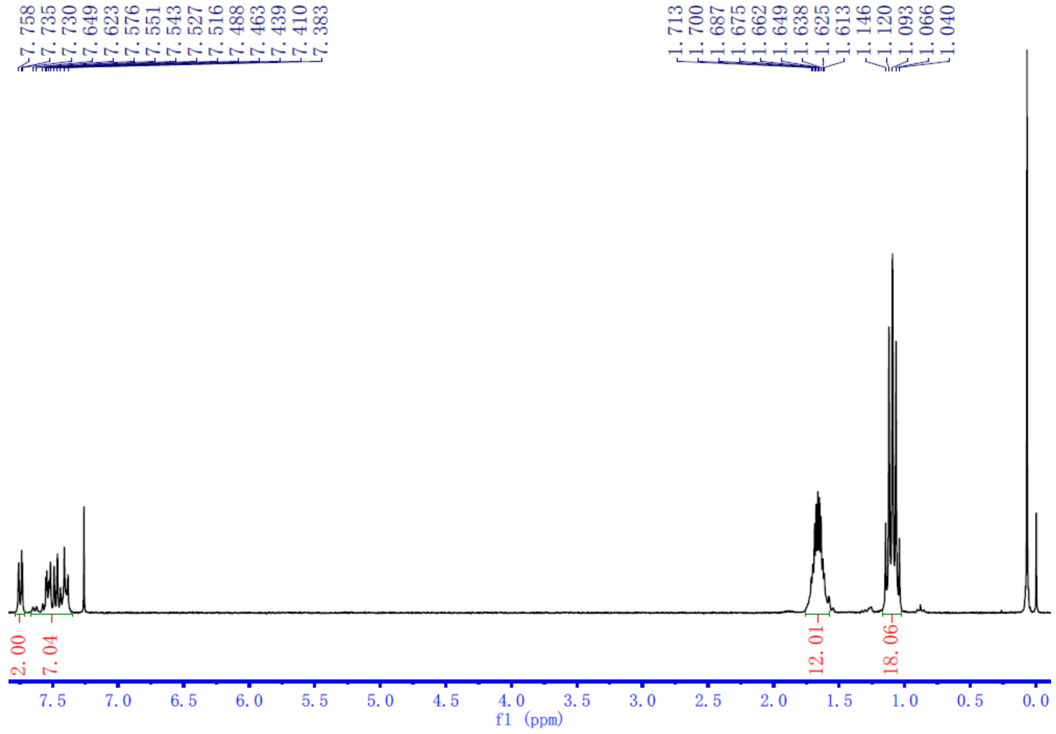

Supplementary Figure 78. ^{1}H NMR spectrum of **9** (300 MHz, $CDCl_3$).

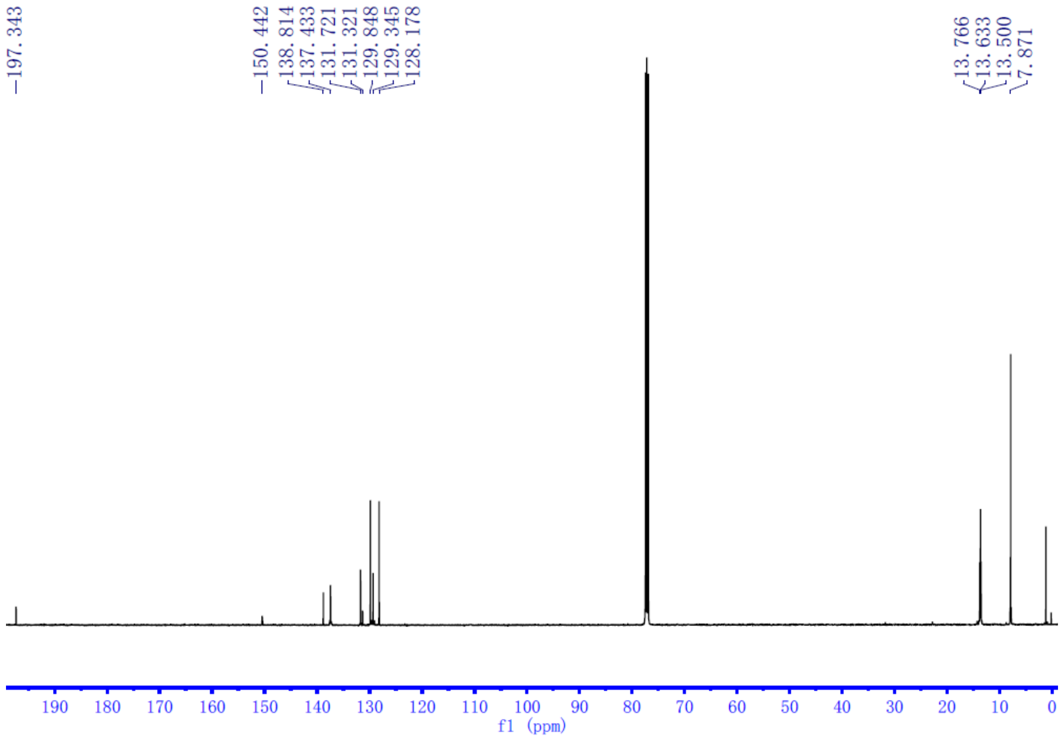

Supplementary Figure 79. ^{13}C NMR spectrum of **9** (126 MHz, $CDCl_3$).

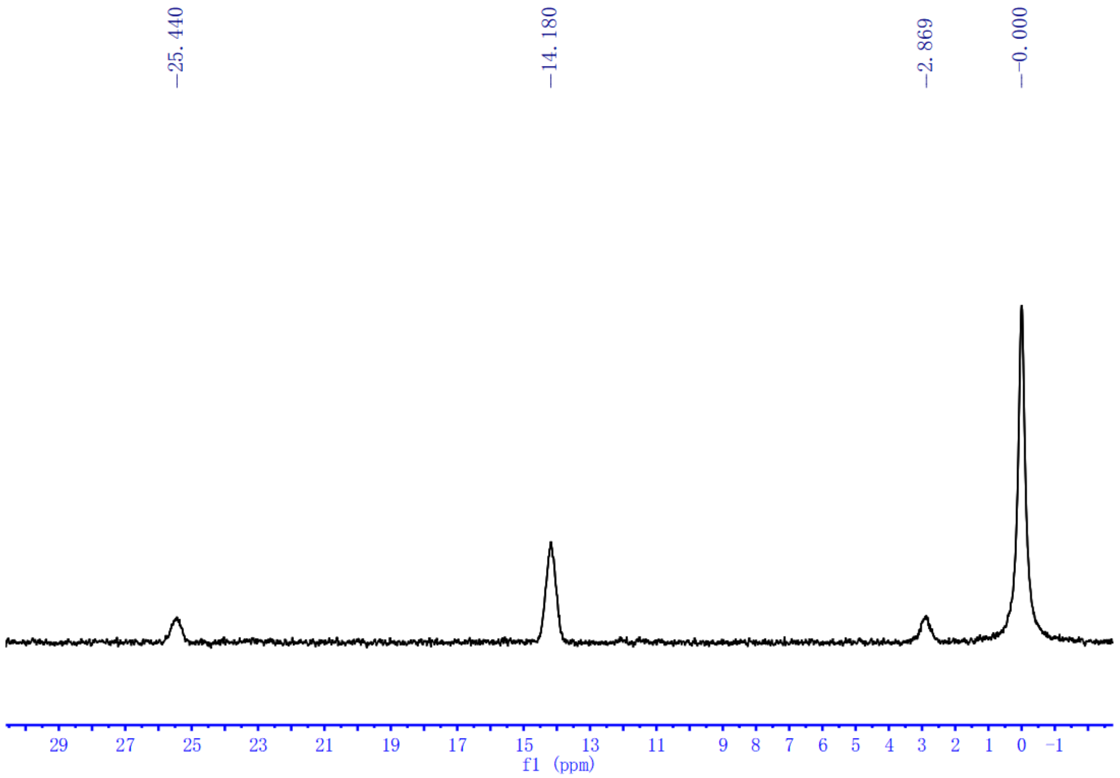

Supplementary Figure 80. ^{31}P NMR spectrum of **9** (122 MHz, $CDCl_3$).

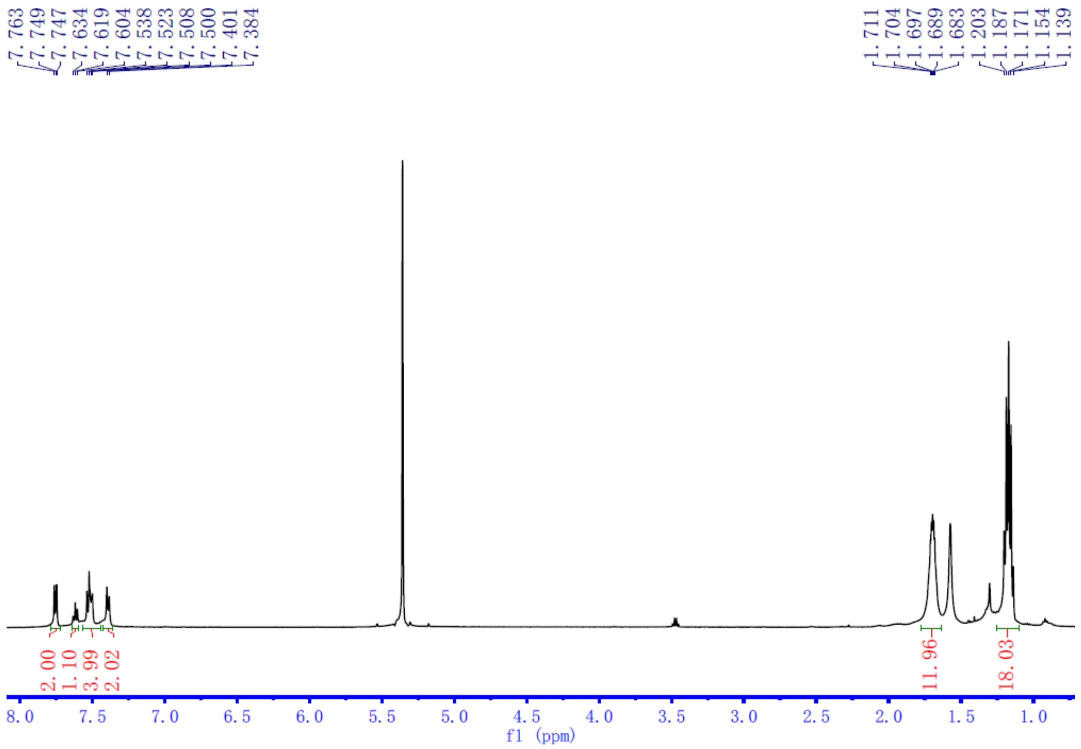

Supplementary Figure 81. ^{1}H NMR spectrum of **10** (500 MHz, CD_2Cl_2).

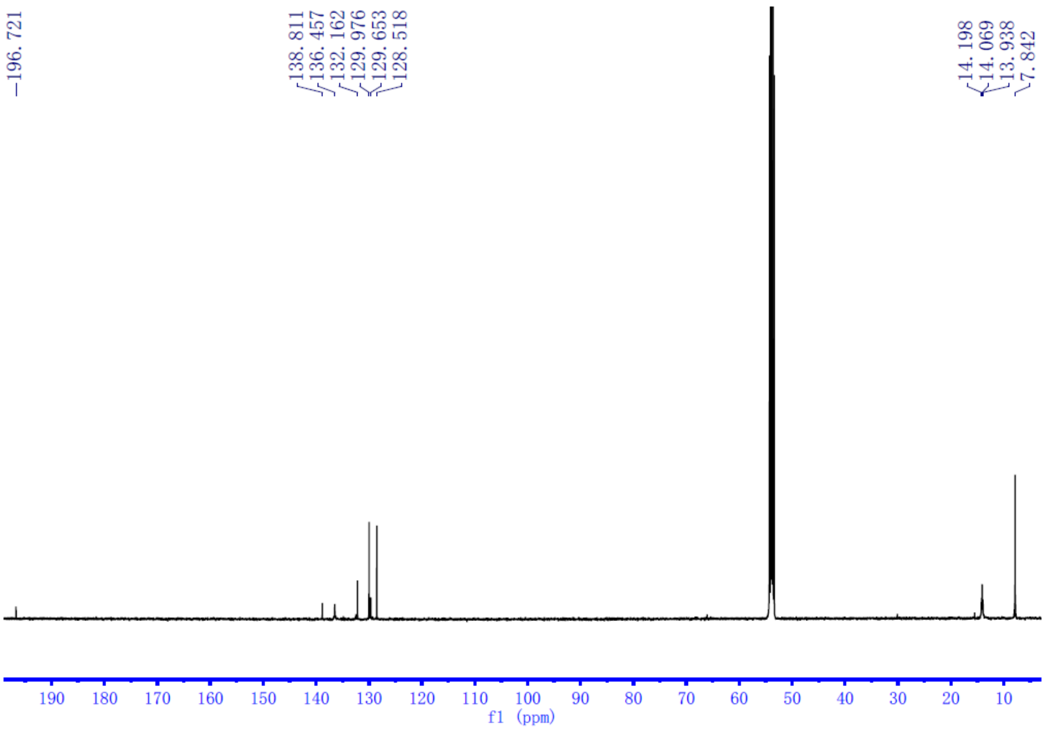

Supplementary Figure 82. ^{13}C NMR spectrum of **10** (126 MHz, CD_2Cl_2).

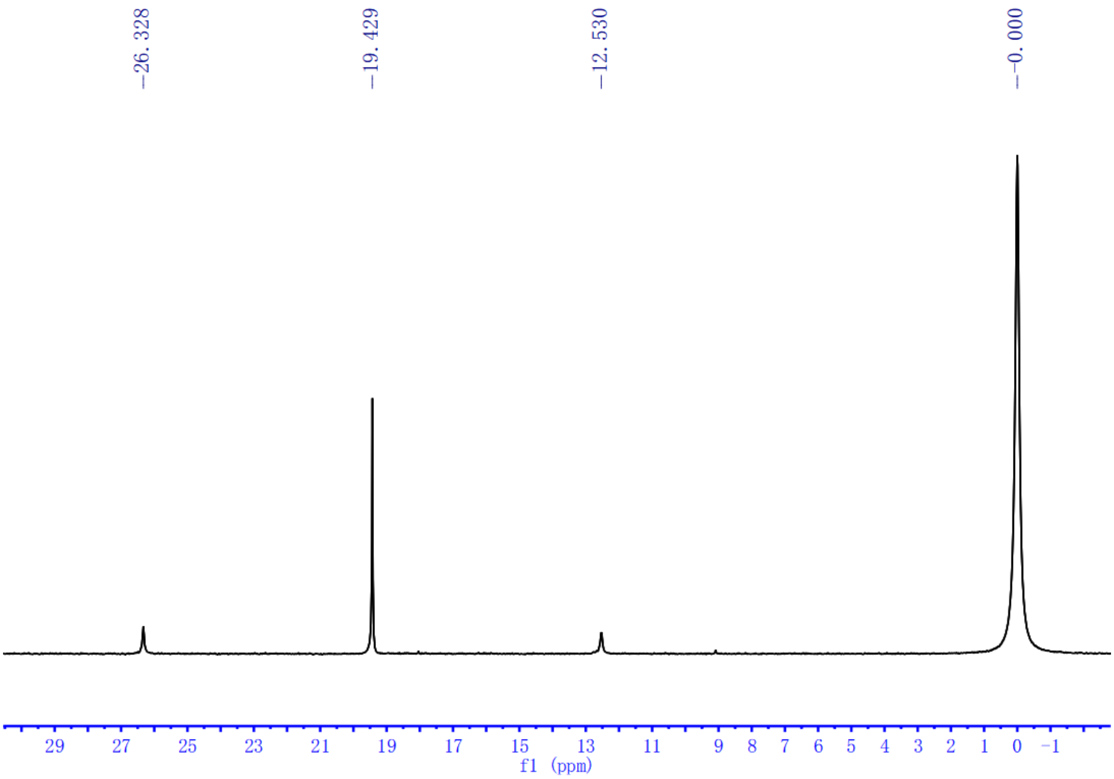

Supplementary Figure 83. ^{31}P NMR spectrum of **10** (202 MHz, CD_2Cl_2).

Elemental Composition Report **Page 1**

Single Mass Analysis
Tolerance = 5.0 mDa / DBE: min = -1.5, max = 50.0
Element prediction: Off

Monoisotopic Mass, Odd and Even Electron Ions
32 formula(e) evaluated with 1 results within limits (up to 50 closest results for each mass)
Elements Used:
C: 0-20 H: 0-15 N: 0-2 F: 0-3 I: 0-2

RYY3-75
20150504006 138 (2.300) TOF MS EI+
8.61e+001

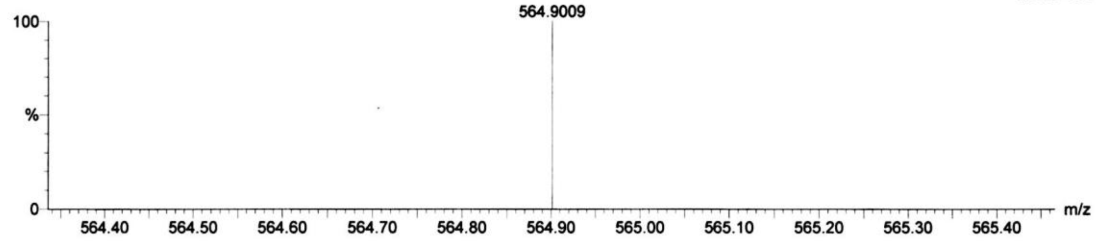

Minimum:				-1.5		
Maximum:		5.0	10.0	50.0		
Mass	Calc. Mass	mDa	PPM	DBE	i-FIT	Formula
564.9009	564.9011	-0.2	-0.4	12.0	5546056.0	C19 H12 N F3 I2

Supplementary Figure 84. High-resolution EI-mass spectrometry analysis of **2**.

Elemental Composition Report **Page 1**

Single Mass Analysis
Tolerance = 5.0 mDa / DBE: min = -1.5, max = 50.0
Element prediction: Off

Monoisotopic Mass, Odd and Even Electron Ions
8 formula(e) evaluated with 1 results within limits (up to 50 closest results for each mass)
Elements Used:
C: 0-30 H: 0-22 N: 0-3 F: 0-3

RYY3-78
20150504008 723 (12.051) TOF MS EI+
9.82e+002

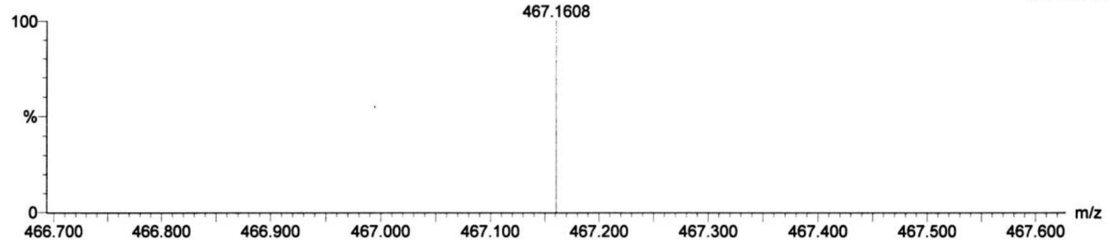

Minimum:				-1.5		
Maximum:		5.0	10.0	50.0		
Mass	Calc. Mass	mDa	PPM	DBE	i-FIT	Formula
467.1608	467.1609	-0.1	-0.2	20.0	5546508.5	C29 H20 N3 F3

Supplementary Figure 85. High-resolution EI-mass spectrometry analysis of ligand **L1**.

Elemental Composition Report

Single Mass Analysis
Tolerance = 5.0 mDa / DBE: min = -1.5, max = 50.0
Element prediction: Off

Monoisotopic Mass, Odd and Even Electron Ions
11 formula(e) evaluated with 1 results within limits (up to 50 best isotopic matches for each mass)
Elements Used:
C: 0-30 H: 0-20 N: 0-4 O: 0-3
RYY-3-92
20151217001 979 (16.318)

TOF MS EI+
1.73e+002

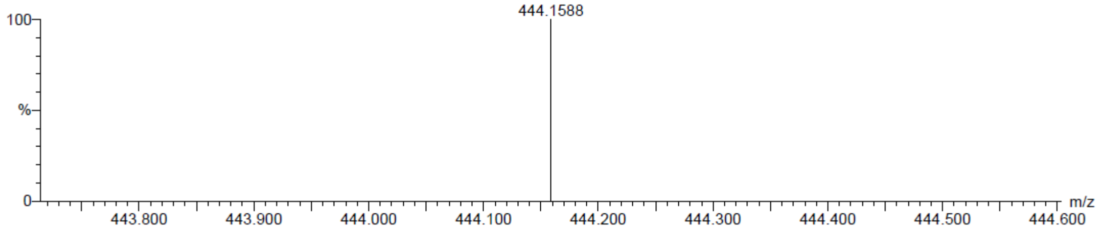

Minimum:				-1.5		
Maximum:		5.0	10.0	50.0		
Mass	Calc. Mass	mDa	PPM	DBE	i-FIT	Formula
444.1588	444.1586	0.2	0.5	21.0	5546104.5	C28 H20 N4 O2

Supplementary Figure 86. High-resolution EI-mass spectrometry analysis of ligand **L2**.

Elemental Composition Report

Single Mass Analysis
Tolerance = 5.0 mDa / DBE: min = -1.5, max = 50.0
Element prediction: Off

Monoisotopic Mass, Odd and Even Electron Ions
7 formula(e) evaluated with 1 results within limits (up to 50 best isotopic matches for each mass)
Elements Used:
C: 0-35 H: 0-25 N: 0-5
ZJL-P3
20180620002 86 (2.850)

TOF MS EI+
6.68e+001

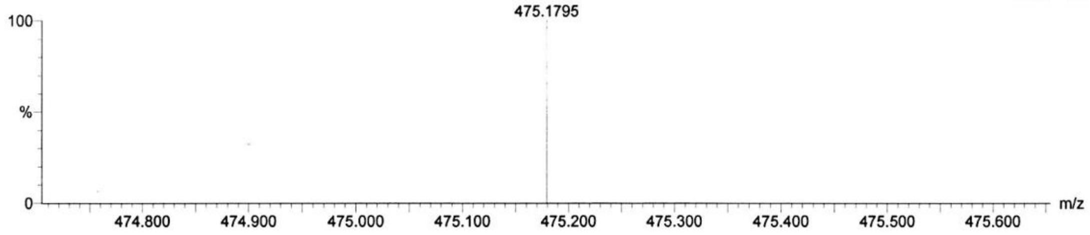

Minimum:				-1.5		
Maximum:		5.0	10.0	50.0		
Mass	Calc. Mass	mDa	PPM	DBE	i-FIT	Formula
475.1795	475.1797	-0.2	-0.4	25.0	5546053.0	C32 H21 N5

Supplementary Figure 87. High-resolution EI-mass spectrometry analysis of ligand **L3**.

Elemental Composition Report **Page 1**

Single Mass Analysis
Tolerance = 5.0 mDa / DBE: min = -1.5, max = 50.0
Element prediction: Off

Monoisotopic Mass, Odd and Even Electron Ions
22 formula(e) evaluated with 1 results within limits (up to 50 best isotopic matches for each mass)
Elements Used:
C: 0-30 H: 0-25 N: 0-5 O: 0-2
ZJL-P4
20180620003 604 (20.118) Cm (576:606) TOF MS EI+ 2.33e+001

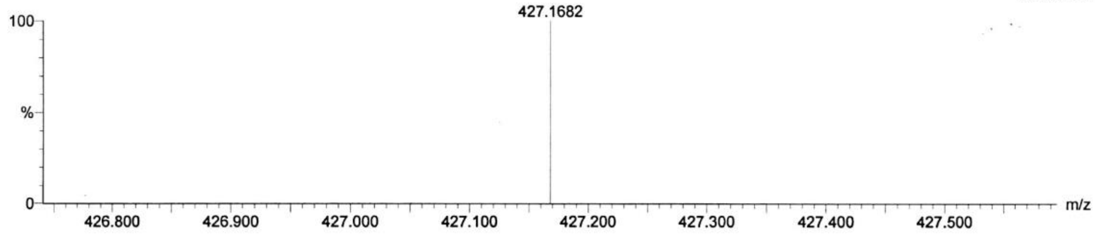

Minimum:				-1.5		
Maximum:		5.0	10.0	50.0		
Mass	Calc. Mass	mDa	PPM	DBE	i-FIT	Formula
427.1682	427.1685	-0.3	-0.7	21.0	5546031.5	C29 H21 N3 O

Supplementary Figure 88. High-resolution EI-mass spectrometry analysis of ligand **L4**.

Elemental Composition Report **Page 1**

Single Mass Analysis
Tolerance = 5.0 mDa / DBE: min = -1.5, max = 50.0
Element prediction: Off

Monoisotopic Mass, Odd and Even Electron Ions
9 formula(e) evaluated with 1 results within limits (up to 50 best isotopic matches for each mass)
Elements Used:
C: 0-30 H: 0-25 N: 0-5
ZJL-P5
20180620004 261 (8.684) Cm (257:263) TOF MS EI+ 1.01e+001

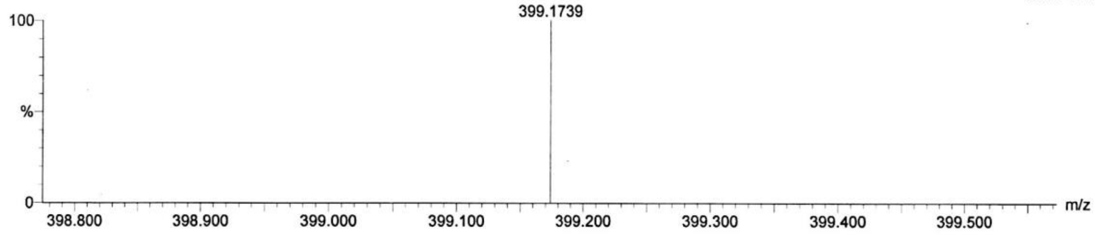

Minimum:				-1.5		
Maximum:		5.0	10.0	50.0		
Mass	Calc. Mass	mDa	PPM	DBE	i-FIT	Formula
399.1739	399.1735	0.4	1.0	20.0	5546026.5	C28 H21 N3

Supplementary Figure 89. High-resolution EI-mass spectrometry analysis of ligand **L5**.

Elemental Composition Report **Page 1**

Single Mass Analysis
Tolerance = 5.0 mDa / DBE: min = -1.5, max = 50.0
Element prediction: Off

Monoisotopic Mass, Odd and Even Electron Ions
8 formula(e) evaluated with 1 results within limits (up to 50 closest results for each mass)
Elements Used:
C: 0-30 H: 0-25 N: 0-3 O: 0-1
ryy3-63
20150424006 485 (8.084) TOF MS EI+ 1.32e+001

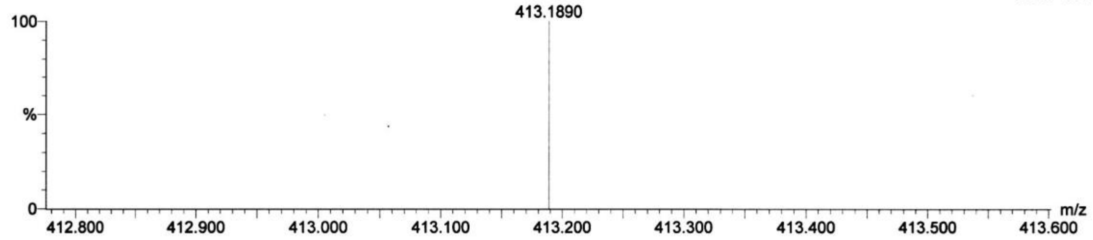

Minimum:				-1.5		
Maximum:		5.0	10.0	50.0		
Mass	Calc. Mass	mDa	PPM	DBE	i-FIT	Formula
413.1890	413.1892	-0.2	-0.5	20.0	5546027.5	C29 H23 N3

Supplementary Figure 90. High-resolution EI-mass spectrometry analysis of ligand **L6**.

Elemental Composition Report **Page 1**

Single Mass Analysis
Tolerance = 5.0 mDa / DBE: min = -1.5, max = 50.0
Element prediction: Off

Monoisotopic Mass, Odd and Even Electron Ions
20 formula(e) evaluated with 1 results within limits (up to 50 closest results for each mass)
Elements Used:
C: 0-30 H: 0-25 N: 0-3 O: 0-1 79Br: 0-1

ryy3-64
20150424005 1070 (17.835) TOF MS EI+ 7.70e+001

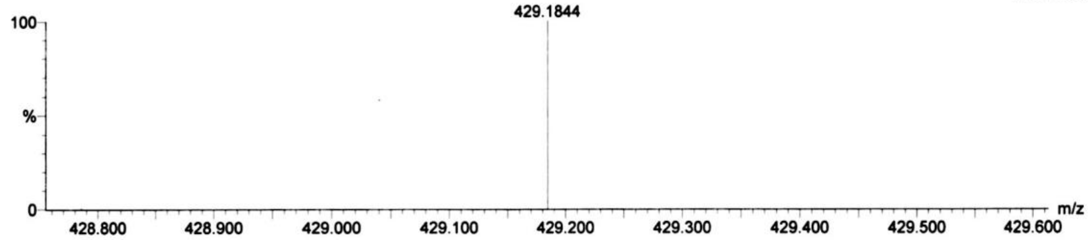

Minimum:				-1.5		
Maximum:		5.0	10.0	50.0		
Mass	Calc. Mass	mDa	PPM	DBE	i-FIT	Formula
429.1844	429.1841	0.3	0.7	20.0	5546057.0	C29 H23 N3 O

Supplementary Figure 91. High-resolution EI-mass spectrometry analysis of ligand **L7**.

Elemental Composition Report

Page 1

Single Mass Analysis

Tolerance = 5.0 mDa / DBE: min = -1.5, max = 50.0
Element prediction: Off

Monoisotopic Mass, Odd and Even Electron Ions
4 formula(e) evaluated with 1 results within limits (up to 50 best isotopic matches for each mass)
Elements Used:
C: 0-30 H: 0-25 N: 0-4
RYY 3-102
20150615004 1243 (20.718)

TOF MS EI+
4.81e+002

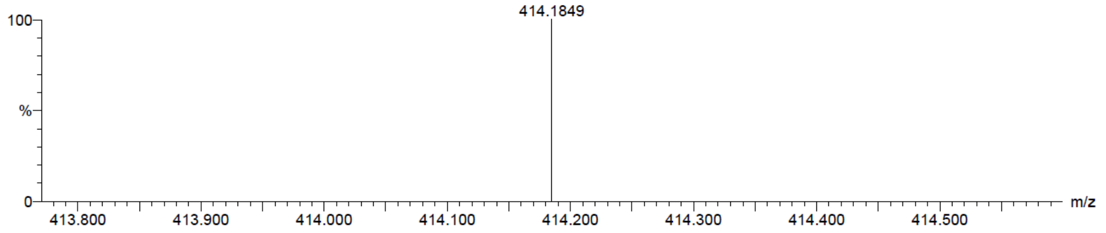

Minimum:				-1.5		
Maximum:		5.0	10.0	50.0		
Mass	Calc. Mass	mDa	PPM	DBE	i-FIT	Formula
414.1849	414.1844	0.5	1.2	20.0	5546258.0	C28 H22 N4

Supplementary Figure 92. High-resolution EI-mass spectrometry analysis of ligand **L8**.

Elemental Composition Report

Page 1

Single Mass Analysis

Tolerance = 5.0 mDa / DBE: min = -1.5, max = 50.0
Element prediction: Off

Monoisotopic Mass, Odd and Even Electron Ions
2 formula(e) evaluated with 1 results within limits (up to 50 best isotopic matches for each mass)
Elements Used:
C: 0-30 H: 0-28 N: 0-4
RYY 3-106
20150615005 906 (15.101)

TOF MS EI+
6.99e+001

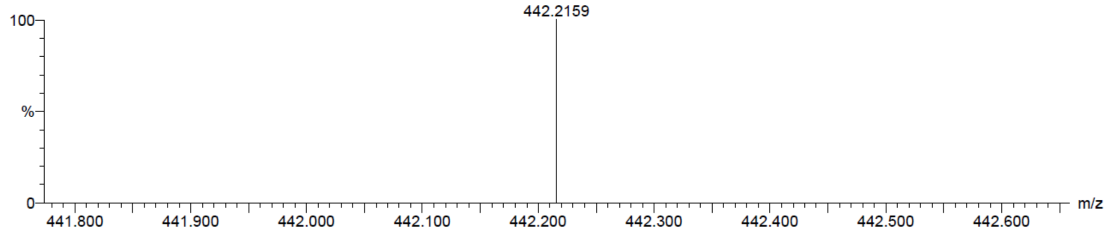

Minimum:				-1.5		
Maximum:		5.0	10.0	50.0		
Mass	Calc. Mass	mDa	PPM	DBE	i-FIT	Formula
442.2159	442.2157	0.2	0.5	20.0	5546054.0	C30 H26 N4

Supplementary Figure 93. High-resolution EI-mass spectrometry analysis of ligand **L9**.

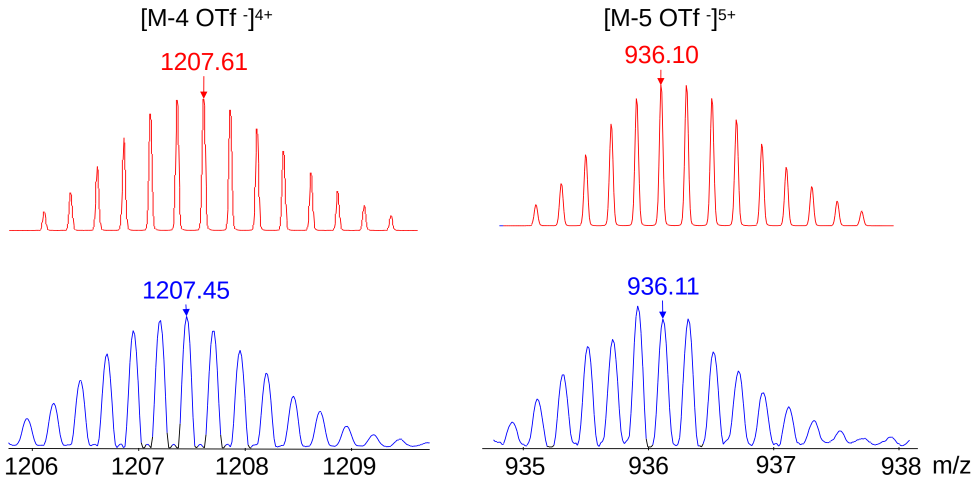

Supplementary Figure 94. Theoretical (top) and experimental (bottom) ESI-TOF-MS spectra of metallacycle **H1**.

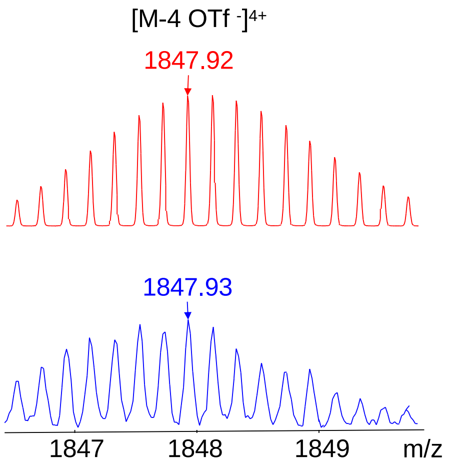

Supplementary Figure 95. Theoretical (top) and experimental (bottom) ESI-TOF-MS spectra of metallacycle **H2**.

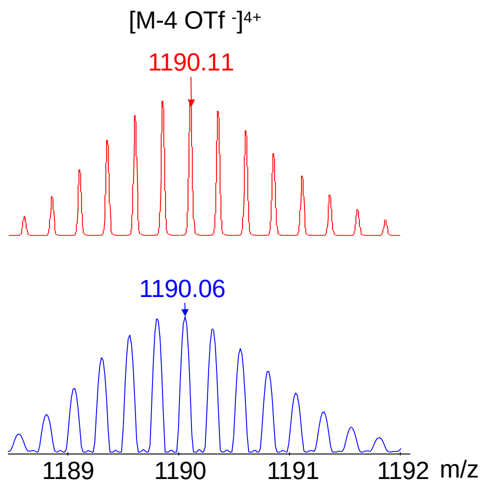

Supplementary Figure 96. Theoretical (top) and experimental (bottom) ESI-TOF-MS spectra of metallacycle **H3**.

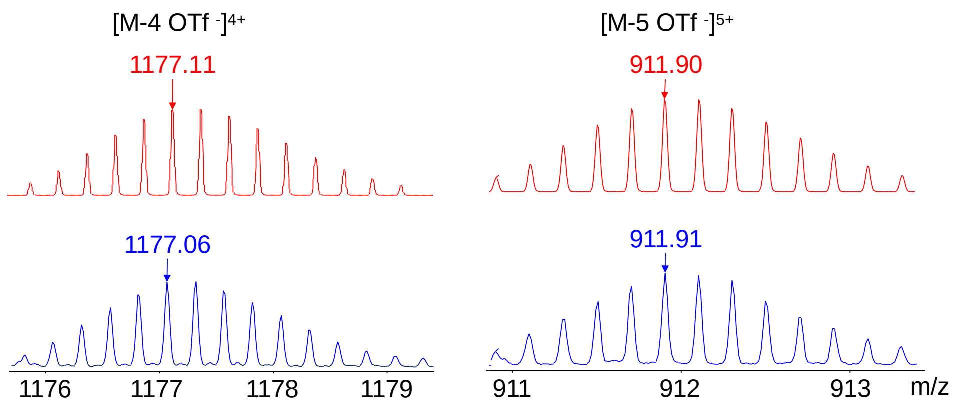

Supplementary Figure 97. Theoretical (top) and experimental (bottom) ESI-TOF-MS spectra of metallacycle **H4**.

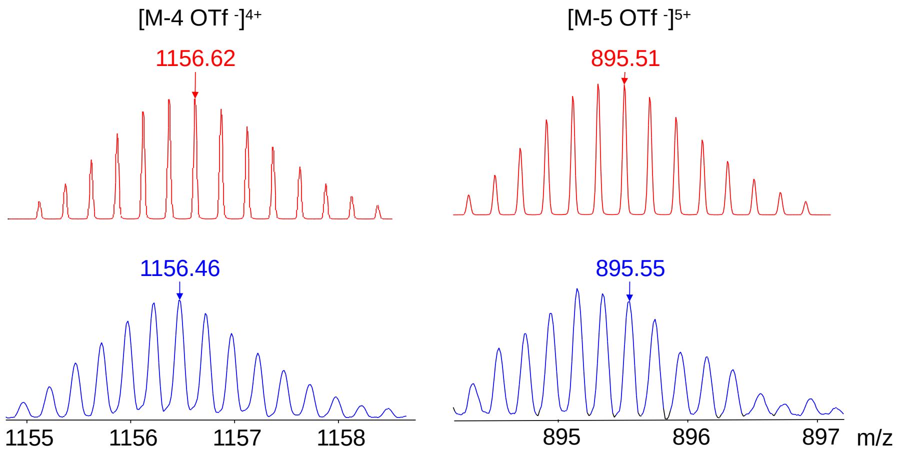

Supplementary Figure 98. Theoretical (top) and experimental (bottom) ESI-TOF-MS spectra of metallacycle **H5**.

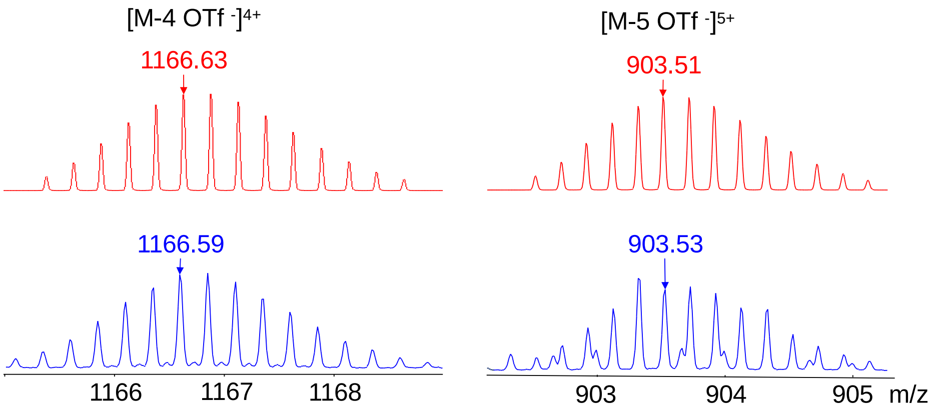

Supplementary Figure 99. Theoretical (top) and experimental (bottom) ESI-TOF-MS spectra of metallacycle **H6**.

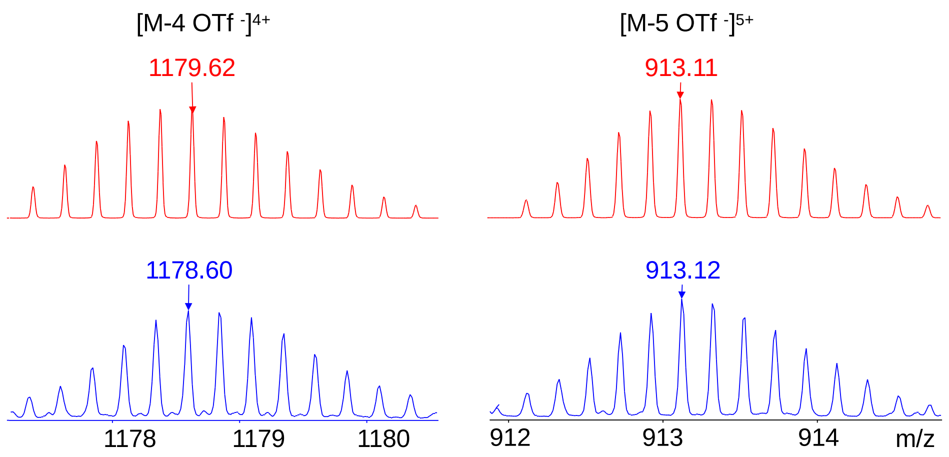

Supplementary Figure 100. Theoretical (top) and experimental (bottom) ESI-TOF-MS spectra of metallacycle **H7**.

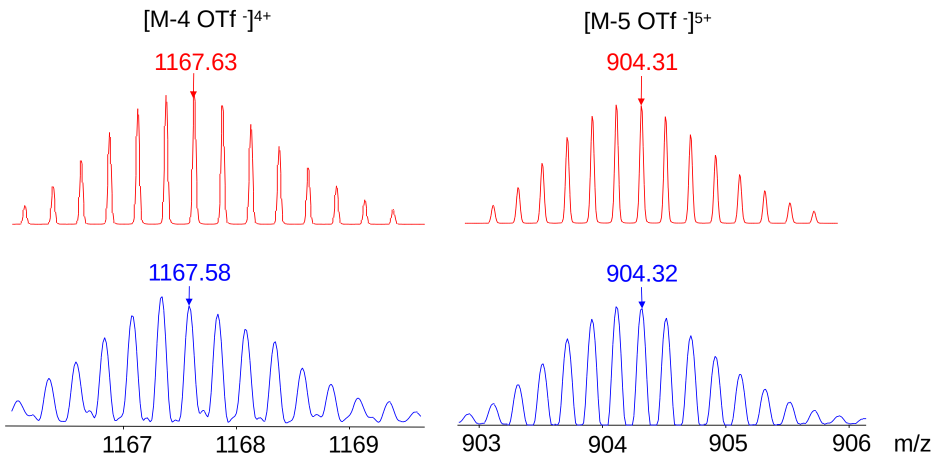

Supplementary Figure 101. Theoretical (top) and experimental (bottom) ESI-TOF-MS spectra of metallacycle **H8**.

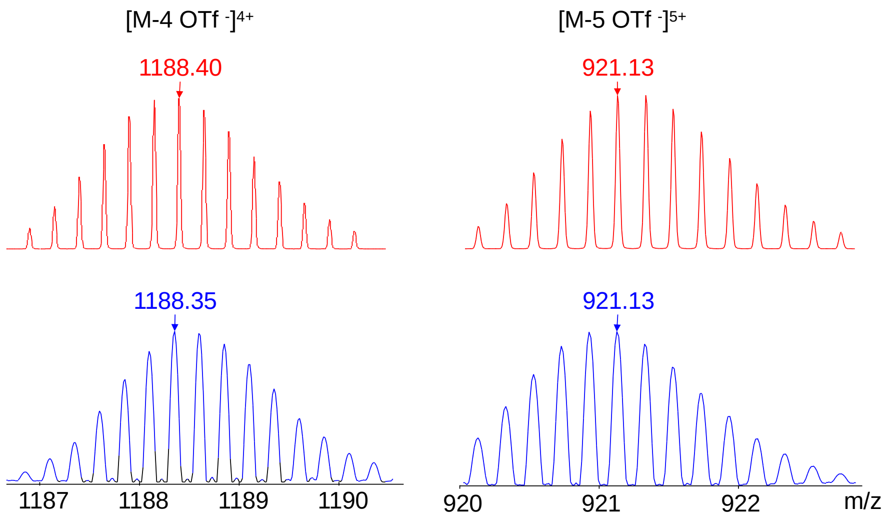

Supplementary Figure 102. Theoretical (top) and experimental (bottom) ESI-TOF-MS spectra of metallacycle **H9**.

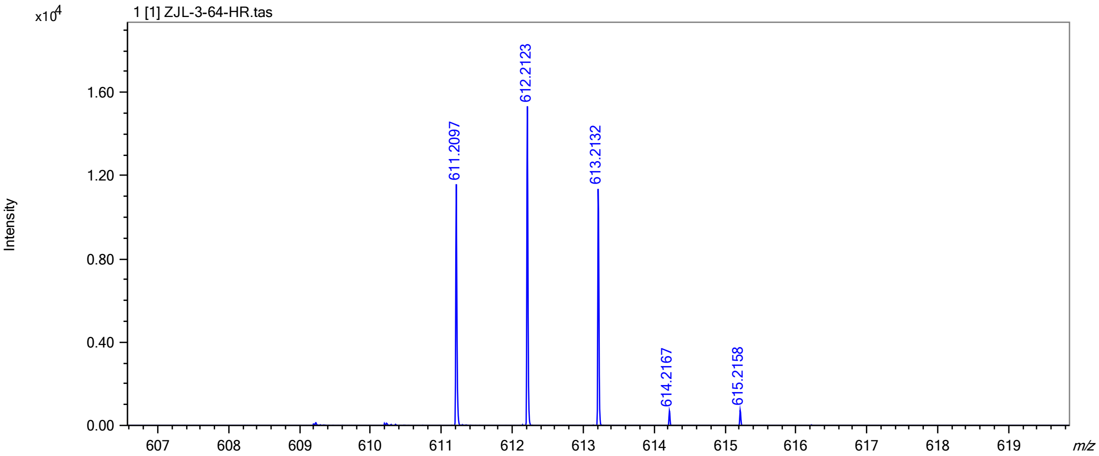

Supplementary Figure 103. High-resolution MALDI-TOF-mass spectrometry analysis of **10** $(M-OTf)^+$.

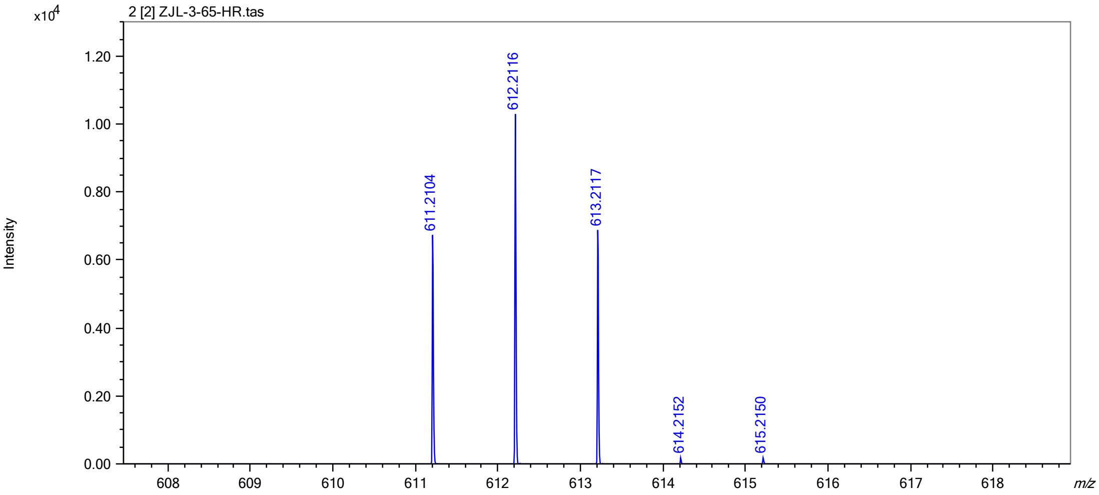

Supplementary Figure 104. High-resolution MALDI-TOF-mass spectrometry analysis of **10** $(M-OTf)^+$.

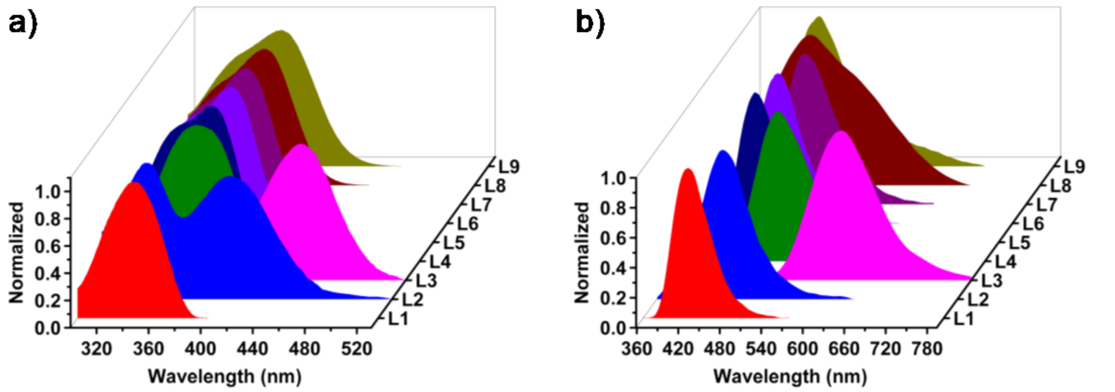

Supplementary Figure 105. Normalized absorption (a) and fluorescence emission (b) spectra of ligands **L1-L9** in DCM.

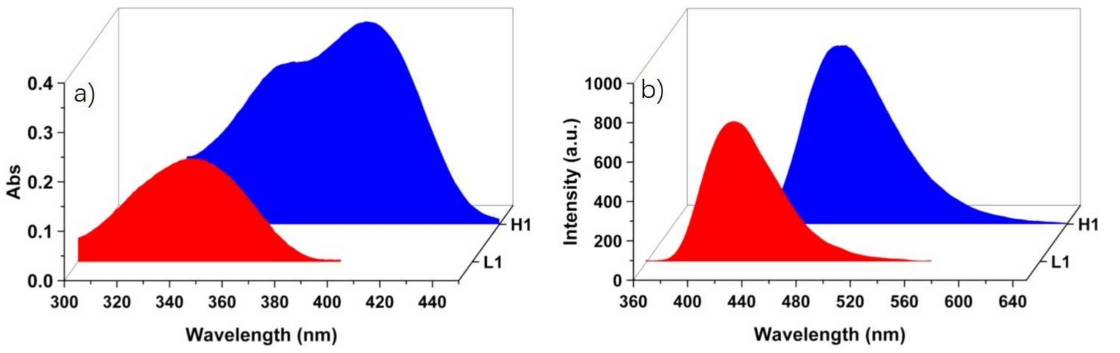

Supplementary Figure 106. Stacked a) absorption and b) emission spectra of **L1** (6 μM) and **H1** (2 μM) in DCM.

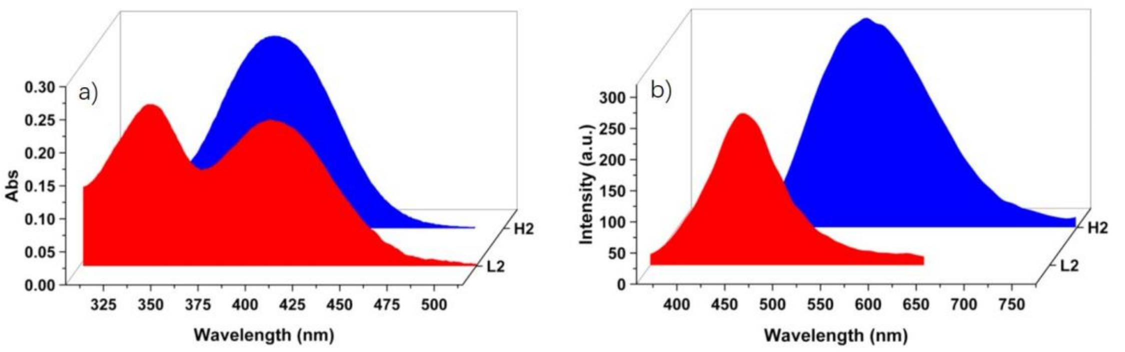

Supplementary Figure 107. Stacked a) absorption and b) emission spectra of **L2** (6 μM) and **H2** (2 μM) in DCM.

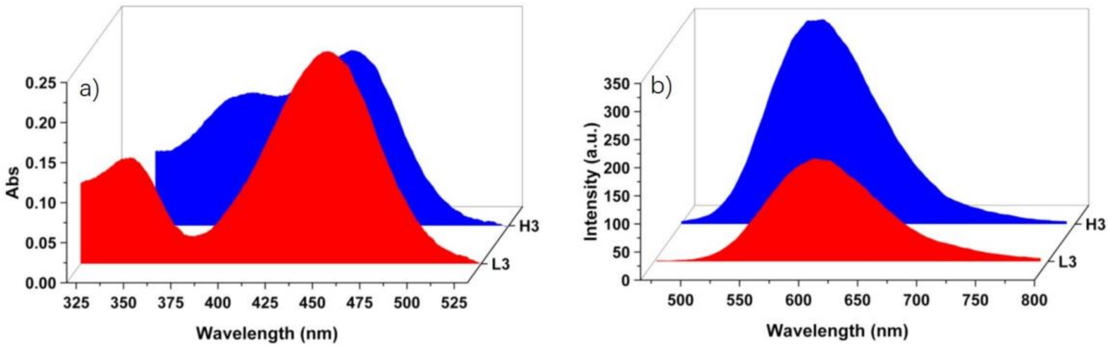

Supplementary Figure 108. Stacked a) absorption and b) emission spectra of **L3** (6 μM) and **H3** (2 μM) in DCM.

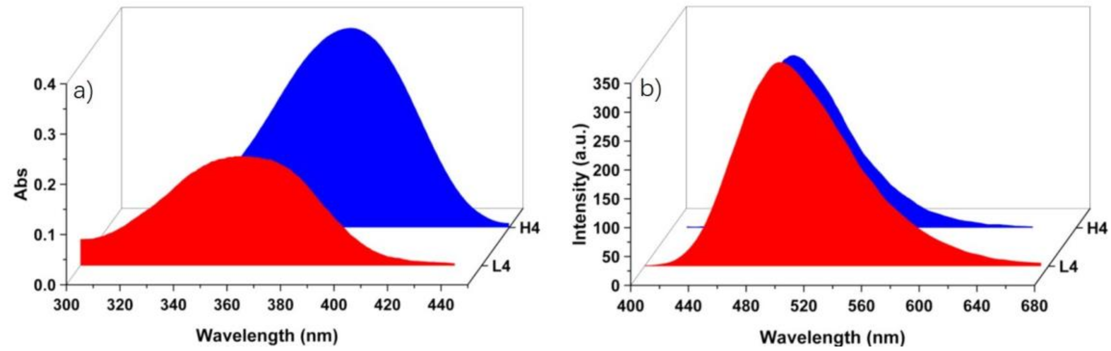

Supplementary Figure 109. Stacked a) absorption and b) emission spectra of **L4** (6 μM) and **H4** (2 μM) in DCM.

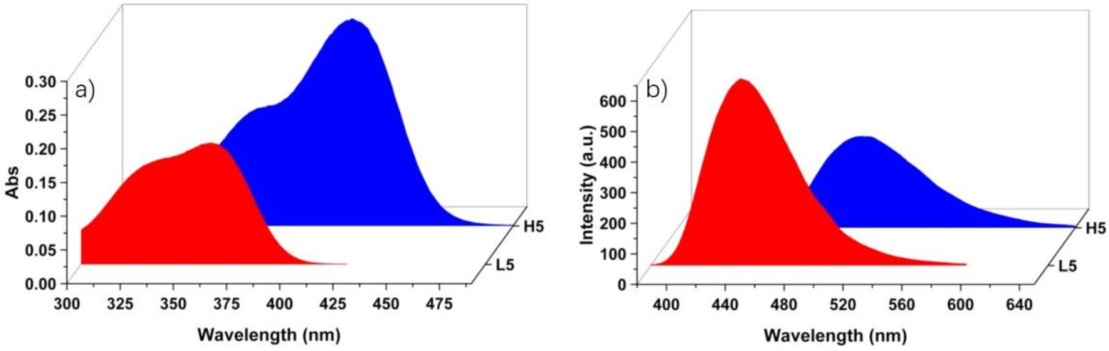

Supplementary Figure 110. Stacked a) absorption and b) emission spectra of **L5** (6 μM) and **H5** (2 μM) in DCM.

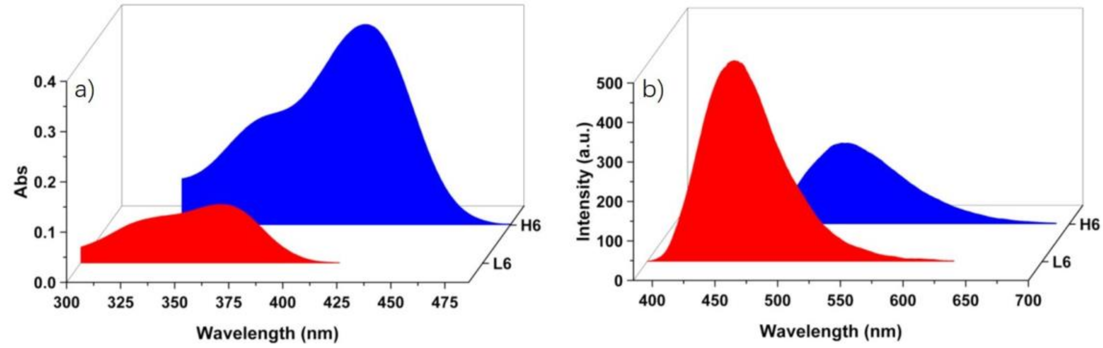

Supplementary Figure 111. Stacked a) absorption and b) emission spectra of **L6** (6 μM) and **H6** (2 μM) in DCM.

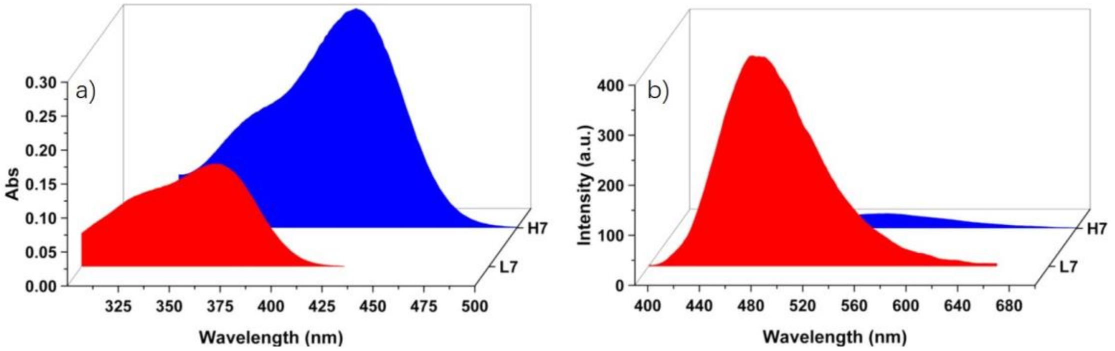

Supplementary Figure 112. Stacked a) absorption and b) emission spectra of **L7** (6 μM) and **H7** (2 μM) in DCM.

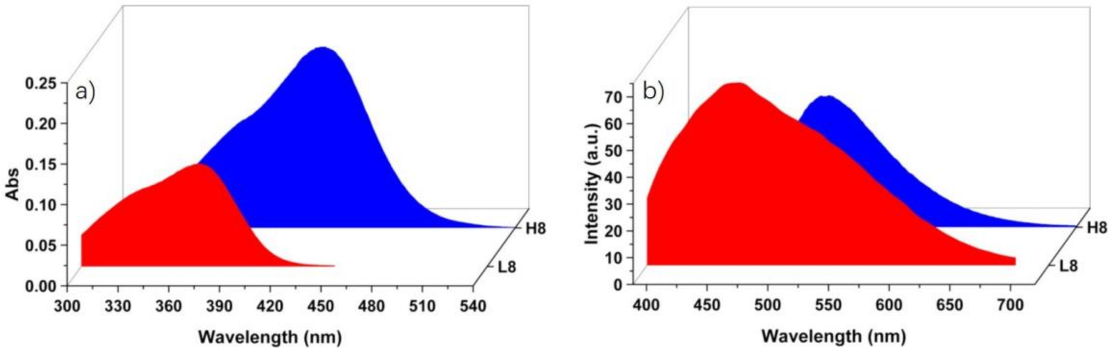

Supplementary Figure 113. Stacked a) absorption and b) emission spectra of **L8** (6 μM) and **H8** (2 μM) in DCM.

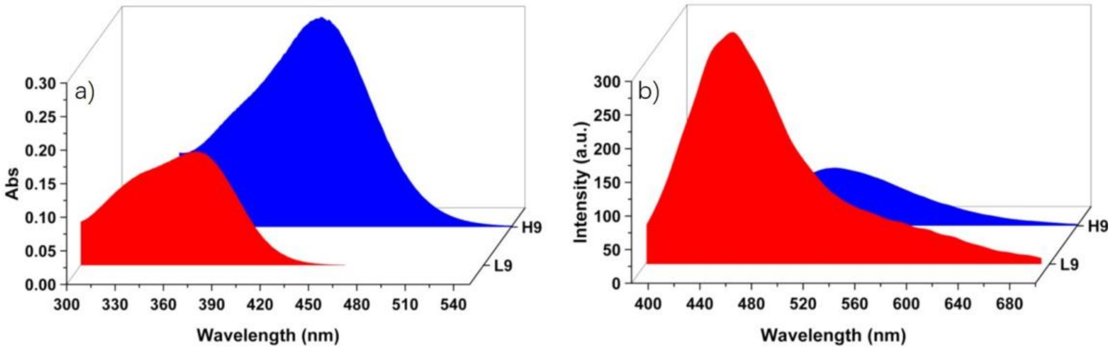

Supplementary Figure 114. Stacked a) absorption and b) emission spectra of **L9** (6 μM) and **H9** (2 μM) in DCM.

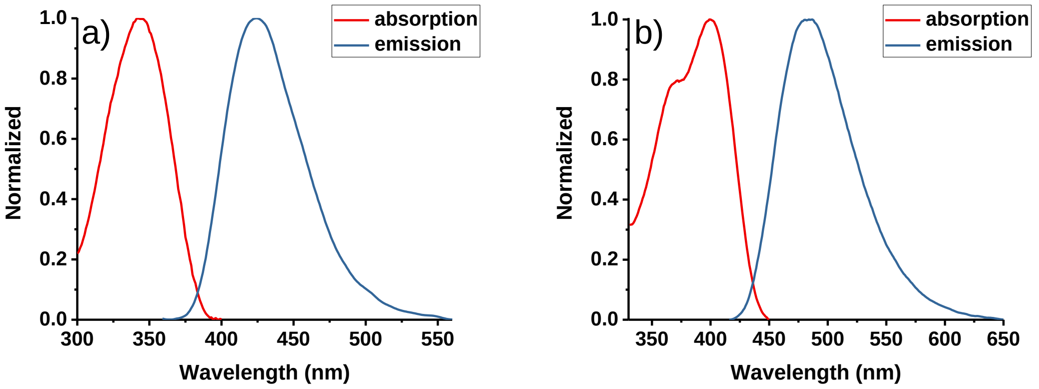

Supplementary Figure 115. Normalized absorption and emission spectra of a) **L1** (6 μM) and b) **H1** (6 μM) in DCM.

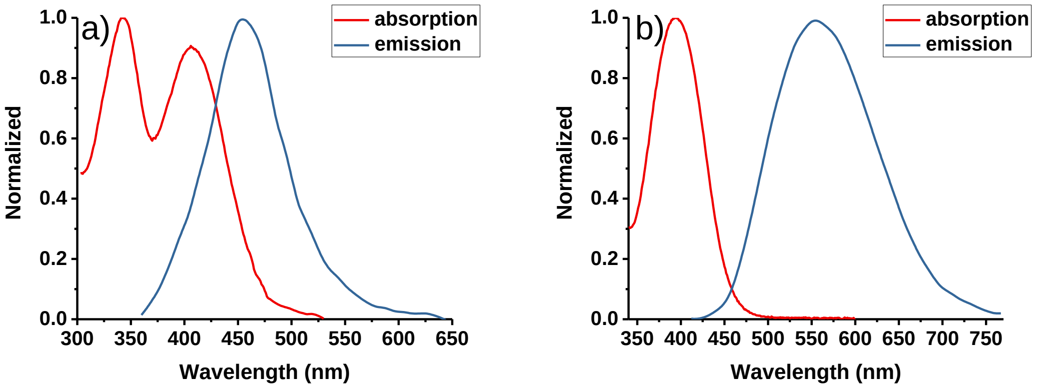

Supplementary Figure 116. Normalized absorption and emission spectra of a) **L2** (6 μM) and b) **H2** (6 μM) in DCM.

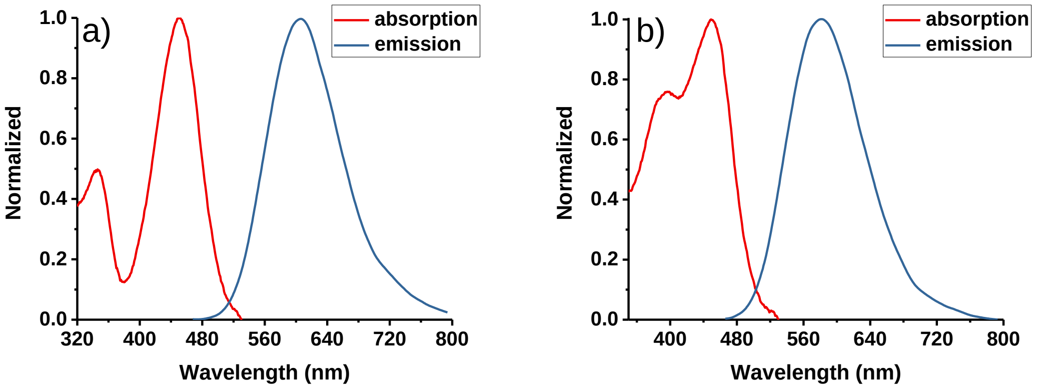

Supplementary Figure 117. Normalized absorption and emission spectra of a) **L3** (6 μM) and b) **H3** (6 μM) in DCM.

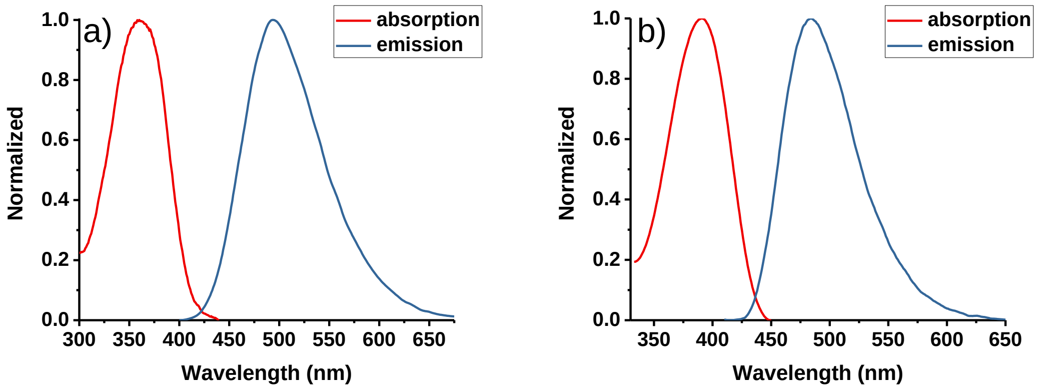

Supplementary Figure 118. Normalized absorption and emission spectra of a) **L4** (6 μM) and b) **H4** (6 μM) in DCM.

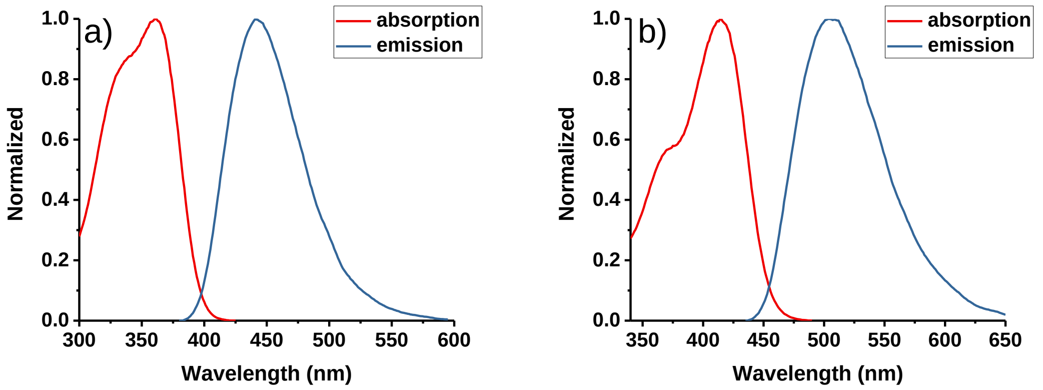

Supplementary Figure 119. Normalized absorption and emission spectra of a) **L5** (6 μM) and b) **H5** (6 μM) in DCM.

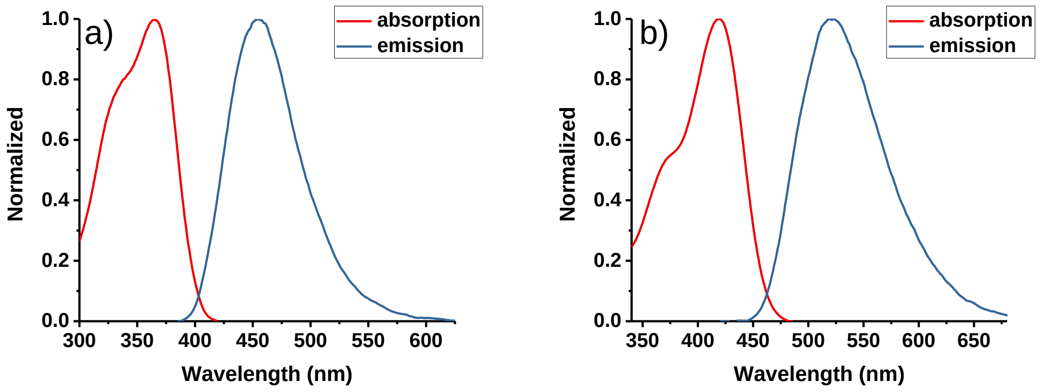

Supplementary Figure 120. Normalized absorption and emission spectra of a) **L6** (6 μM) and b) **H6** (6 μM) in DCM.

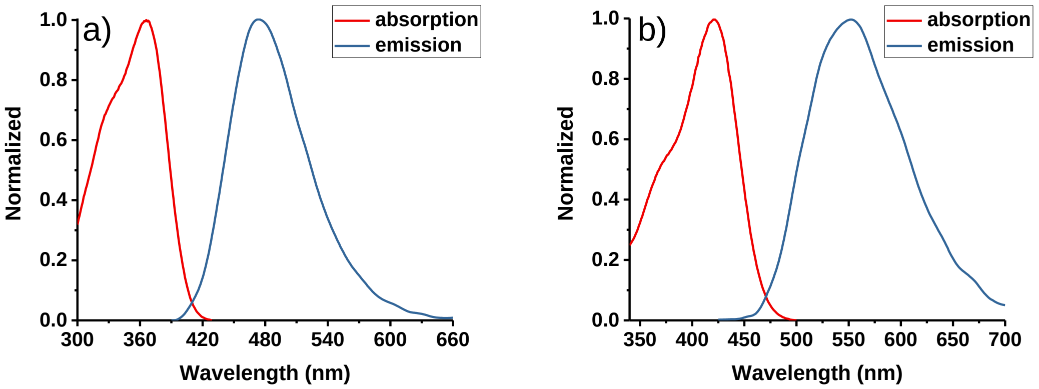

Supplementary Figure 121. Normalized absorption and emission spectra of a) **L7** (6 μM) and b) **H7** (6 μM) in DCM.

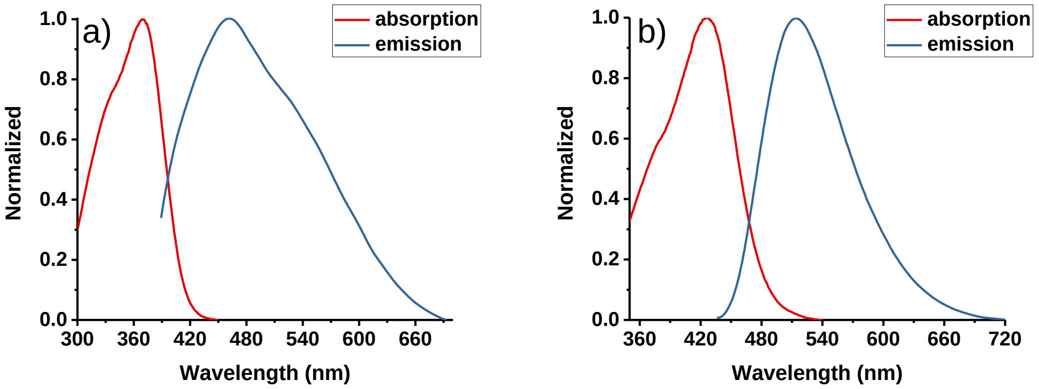

Supplementary Figure 122. Normalized absorption and emission spectra of a) **L8** (6 μM) and b) **H8** (6 μM) in DCM.

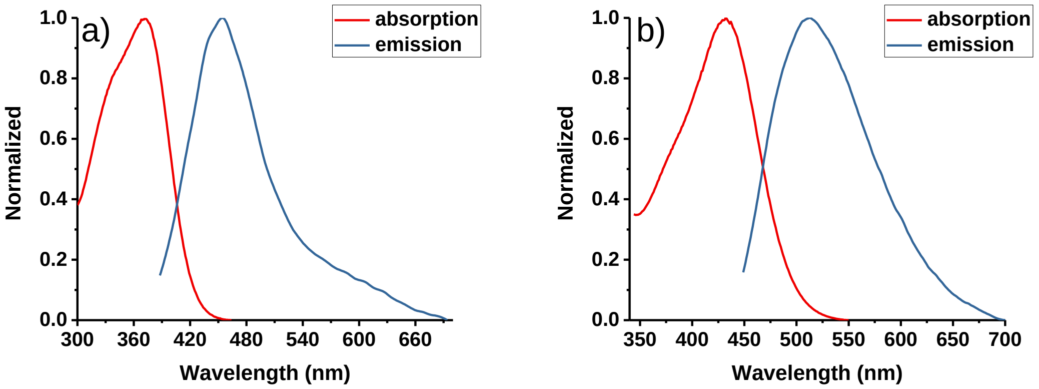

Supplementary Figure 123. Normalized absorption and emission spectra of a) **L9** (6 μM) and b) **H9** (6 μM) in DCM.

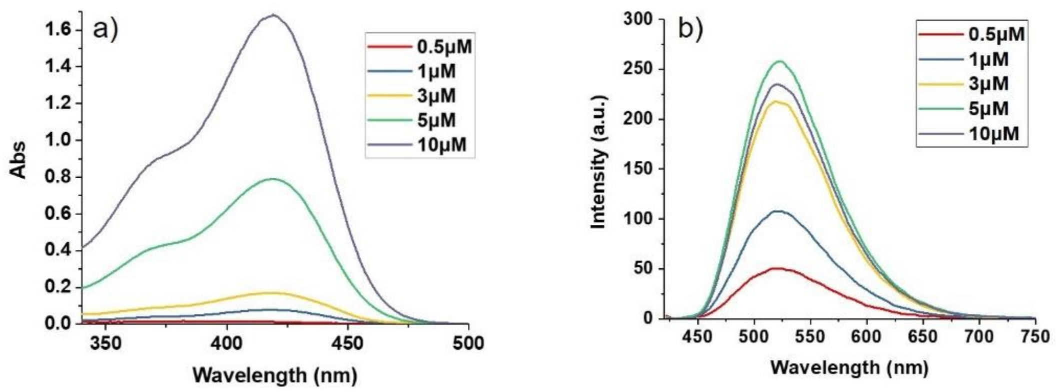

Supplementary Figure 124. Absorption (a) and fluorescent emission (b) spectra of metallacycle **H6** in CH_2Cl_2 at different concentrations.

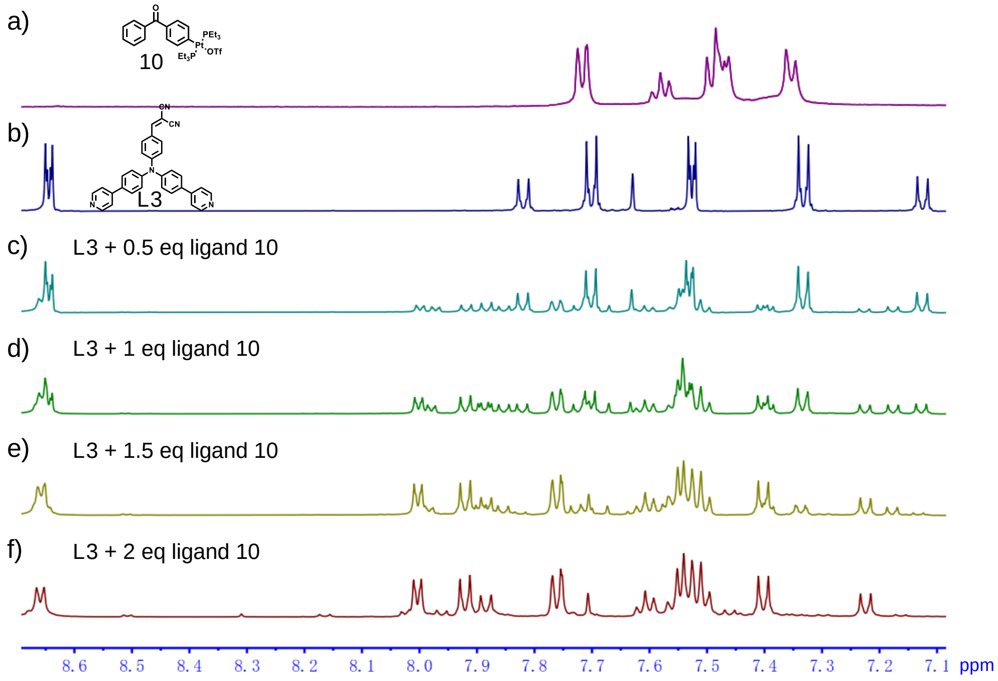

Supplementary Figure 125. Partial ^{1}H NMR spectra of mono-Pt(II) ligand **10** (a), ligand **L3** (b) and ligand **L3** upon addition of 0.5 eq. ligand **10** (c), 1.0 eq. ligand **10** (d), 1.5 eq. ligand **10** (e), 2.0 eq. ligand **10** (f) in CD_2Cl_2.

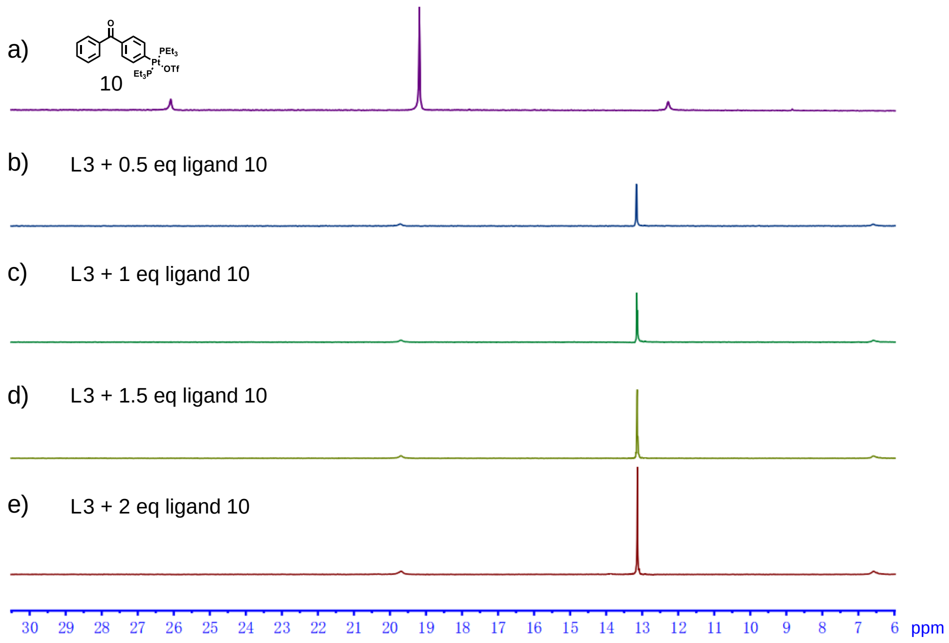

Supplementary Figure 126. Partial ^{31}P NMR spectra of mono-Pt(II) ligand **10** (a), and ligand **L3** upon addition of 0.5 eq. ligand **10** (b), 1.0 eq. ligand **10** (c), 1.5 eq. ligand **10** (d), 2.0 eq. ligand **10** (e) in CD_2Cl_2.

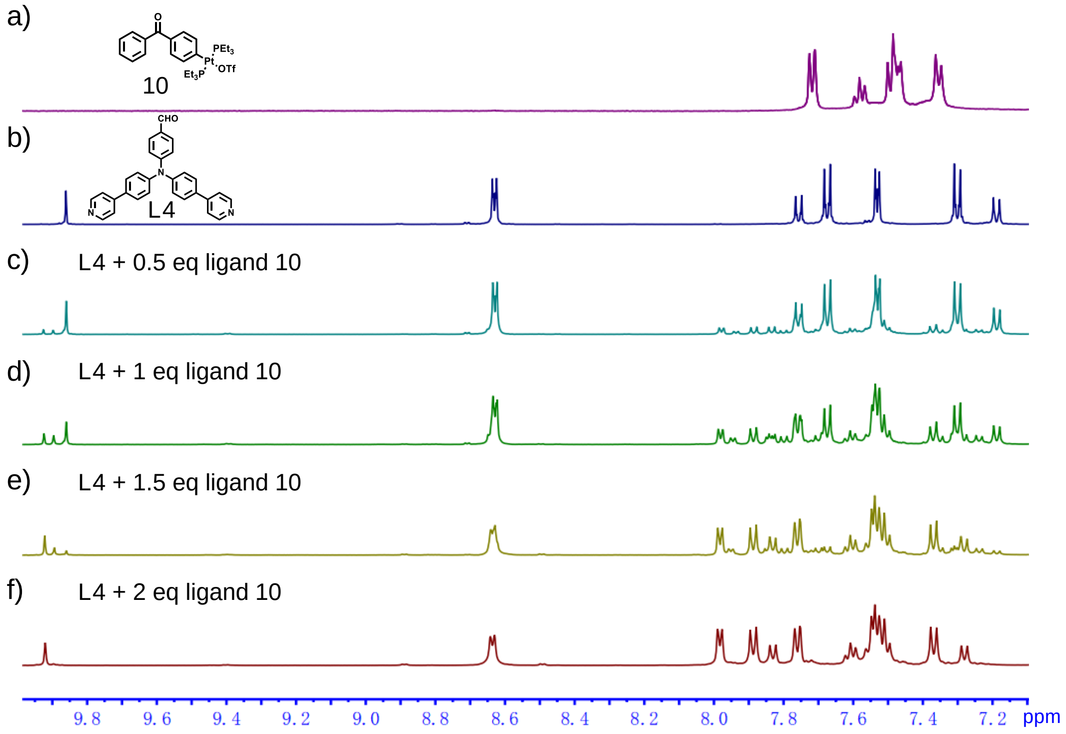

Supplementary Figure 127. Partial ^{1}H NMR spectra of mono-Pt(II) ligand **10** (a), ligand **L4** (b) and ligand **L4** upon addition of 0.5 eq. ligand **10** (c), 1.0 eq. ligand **10** (d), 1.5 eq. ligand **10** (e), 2.0 eq. ligand **10** (f) in CD_2Cl_2.

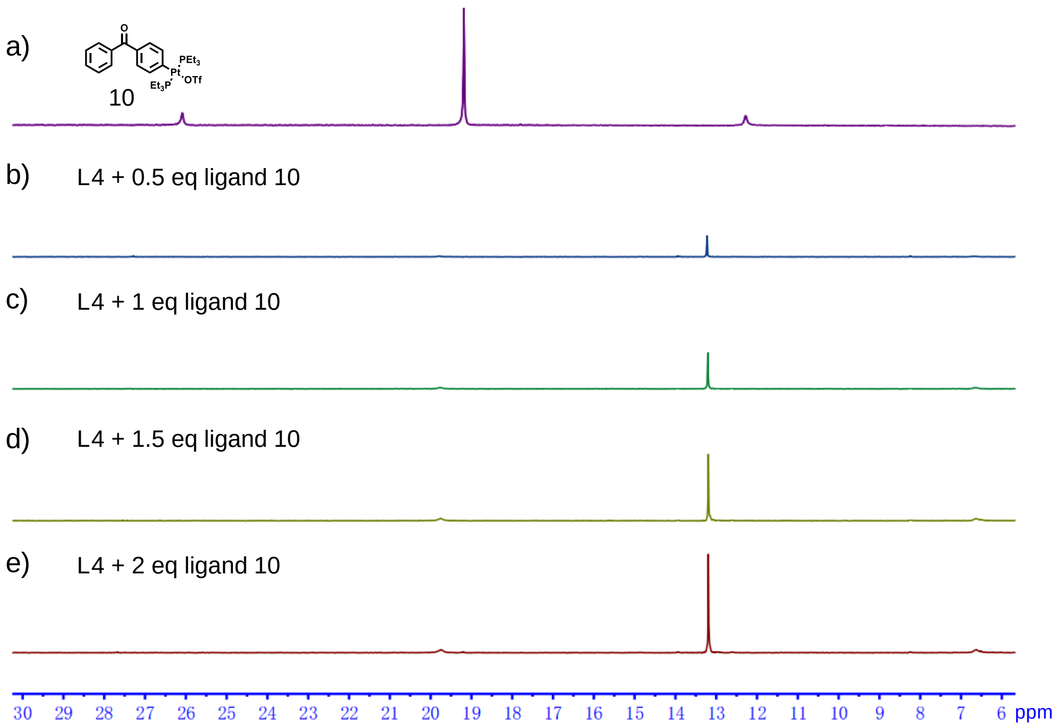

Supplementary Figure 128. Partial ^{31}P NMR spectra of mono-Pt(II) ligand **10** (a), and ligand **L4** upon addition of 0.5 eq. ligand **10** (b), 1.0 eq. ligand **10** (c), 1.5 eq. ligand **10** (d), 2.0 eq. ligand **10** (e) in CD_2Cl_2.

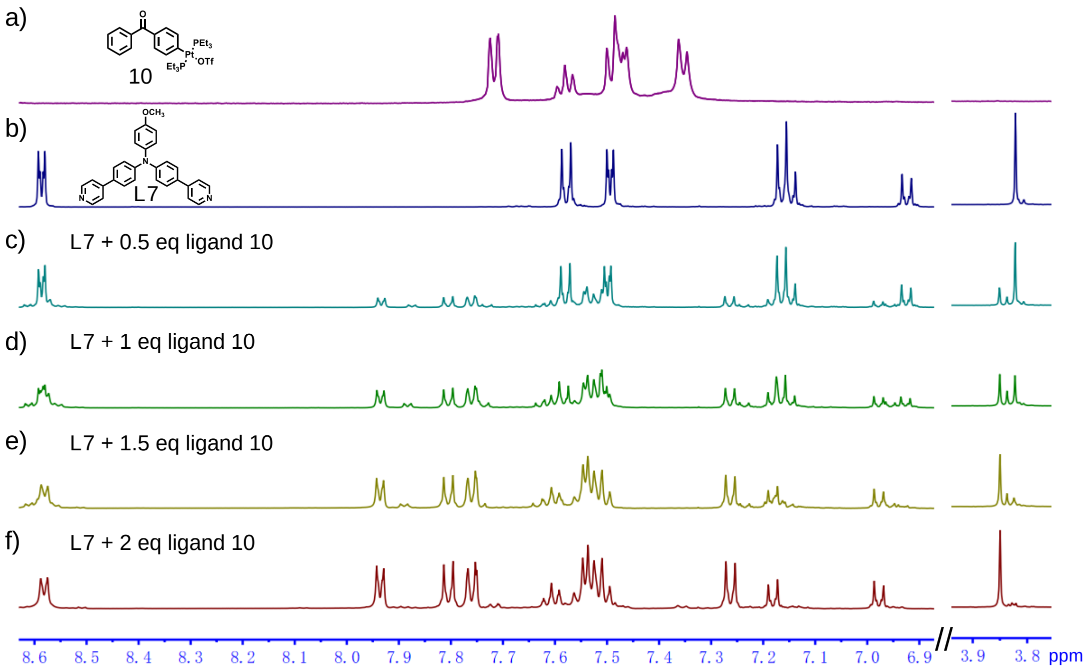

Supplementary Figure 129. Partial ^{1}H NMR spectra of mono-Pt(II) ligand **10** (a), ligand **L7** (b) and ligand **L7** upon addition of 0.5 eq. ligand **10** (c), 1.0 eq. ligand **10** (d), 1.5 eq. ligand **10** (e), 2.0 eq. ligand **10** (f) in CD_2Cl_2.

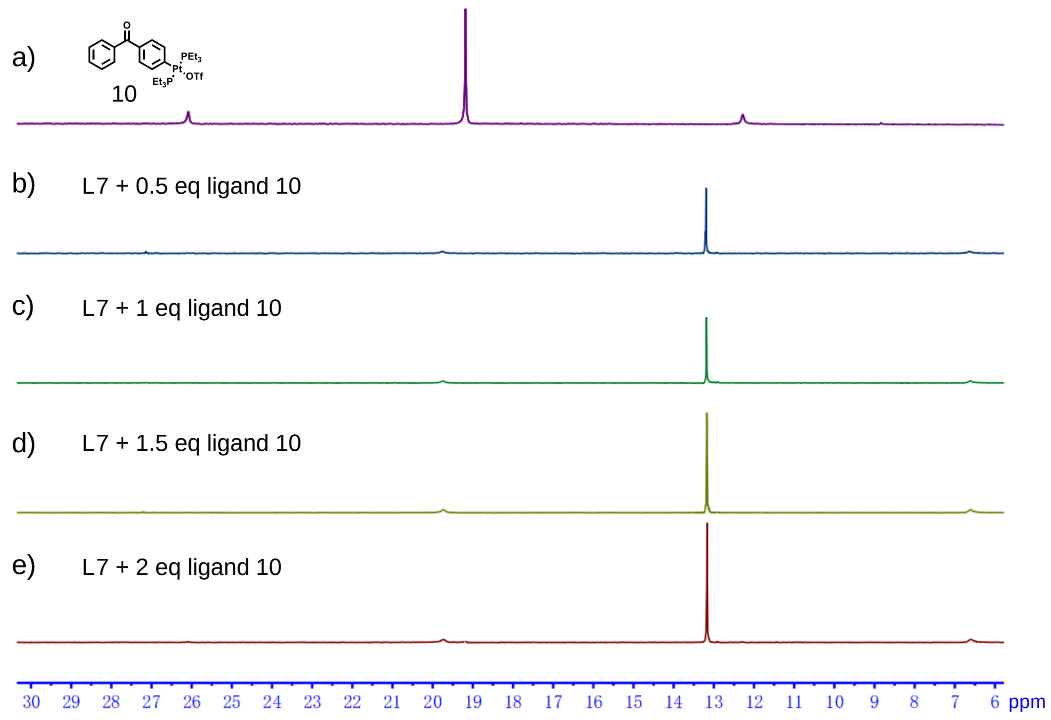

Supplementary Figure 130. Partial ^{31}P NMR spectra of mono-Pt(II) ligand **10** (a), and ligand **L7** upon addition of 0.5 eq. ligand **10** (b), 1.0 eq. ligand **10** (c), 1.5 eq. ligand **10** (d), 2.0 eq. ligand **10** (e) in CD_2Cl_2.

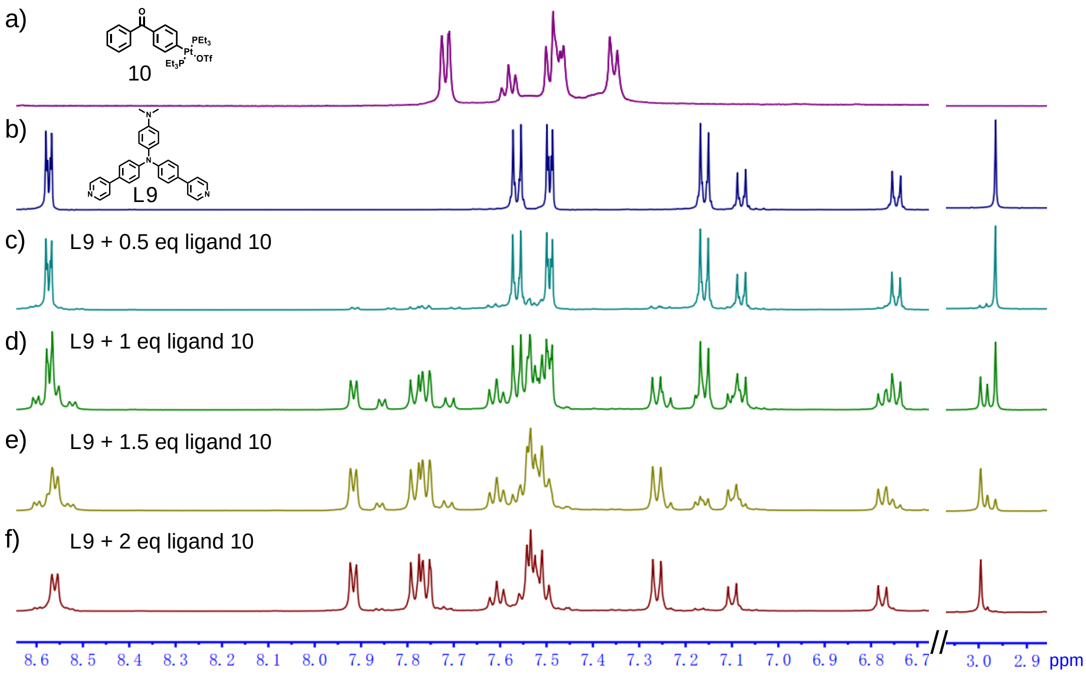

Supplementary Figure 131. Partial ^{1}H NMR spectra of mono-Pt(II) ligand **10** (a), ligand **L9** (b) and ligand **L9** upon addition of 0.5 eq. ligand **10** (c), 1.0 eq. ligand **10** (d), 1.5 eq. ligand **10** (e), 2.0 eq. ligand **10** (f) in CD_2Cl_2.

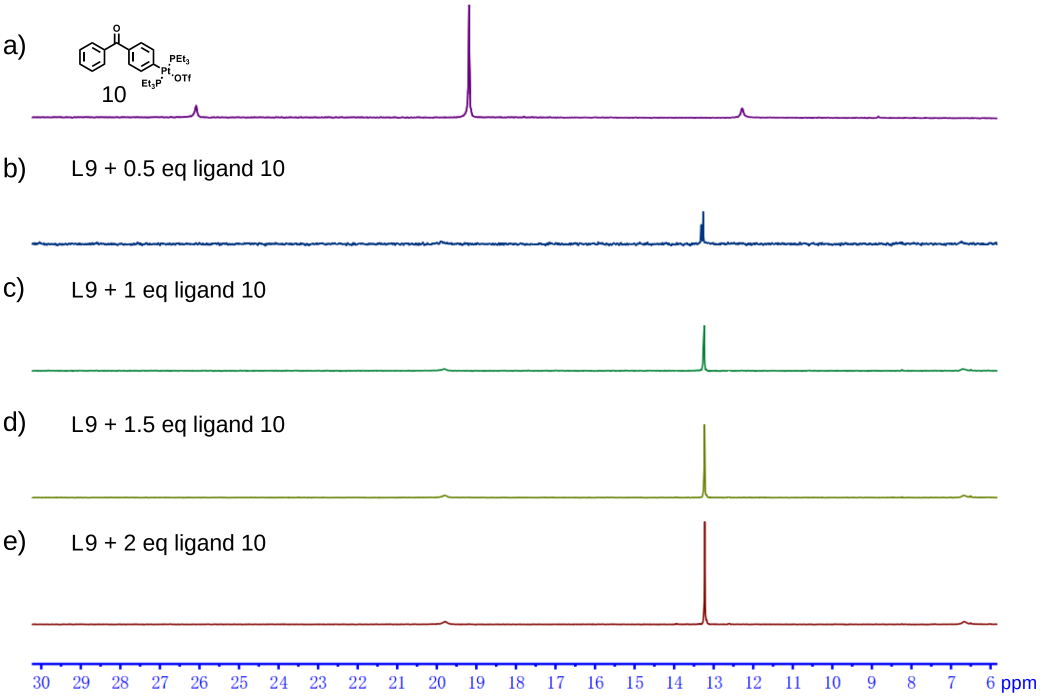

Supplementary Figure 132. Partial ^{31}P NMR spectra of mono-Pt(II) ligand **10** (a), and ligand **L9** upon addition of 0.5 eq. ligand **10** (b), 1.0 eq. ligand **10** (c), 1.5 eq. ligand **10** (d), 2.0 eq. ligand **10** (e) in CD_2Cl_2.

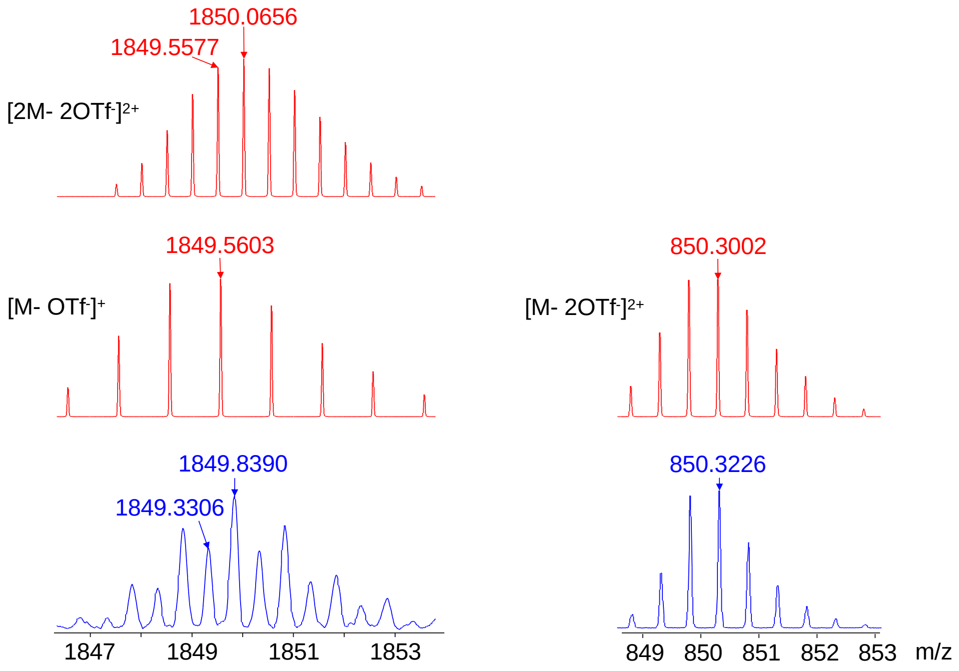

Supplementary Figure 133. Theoretical (red) and experimental (blue) ESI-TOF-MS spectra of ligand **L3** upon the addition of 2eq. ligand **10**.

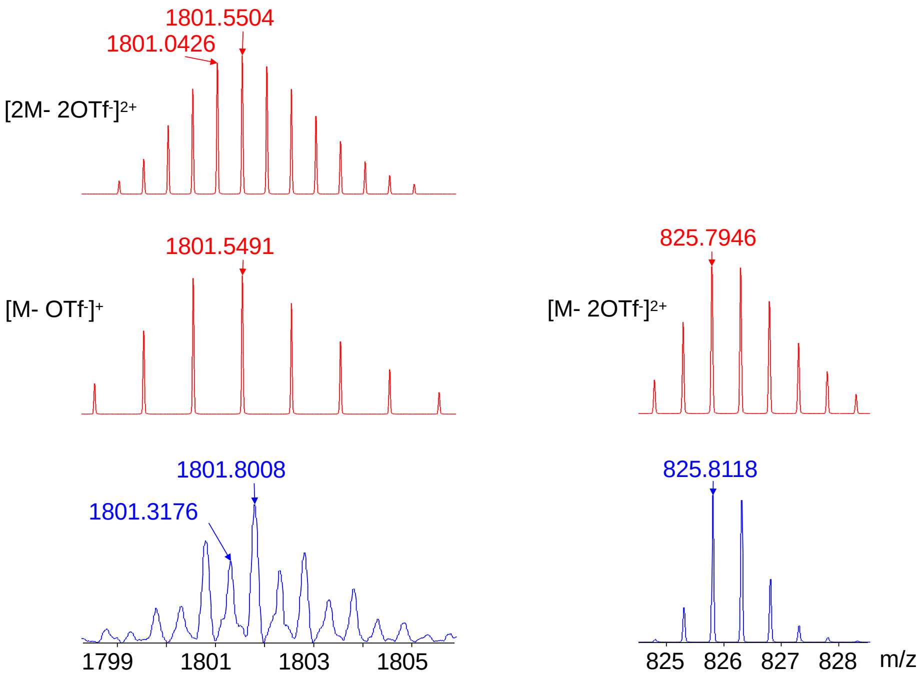

Supplementary Figure 134. Theoretical (red) and experimental (blue) ESI-TOF-MS spectra of ligand **L4** upon the addition of 2eq. ligand **10**.

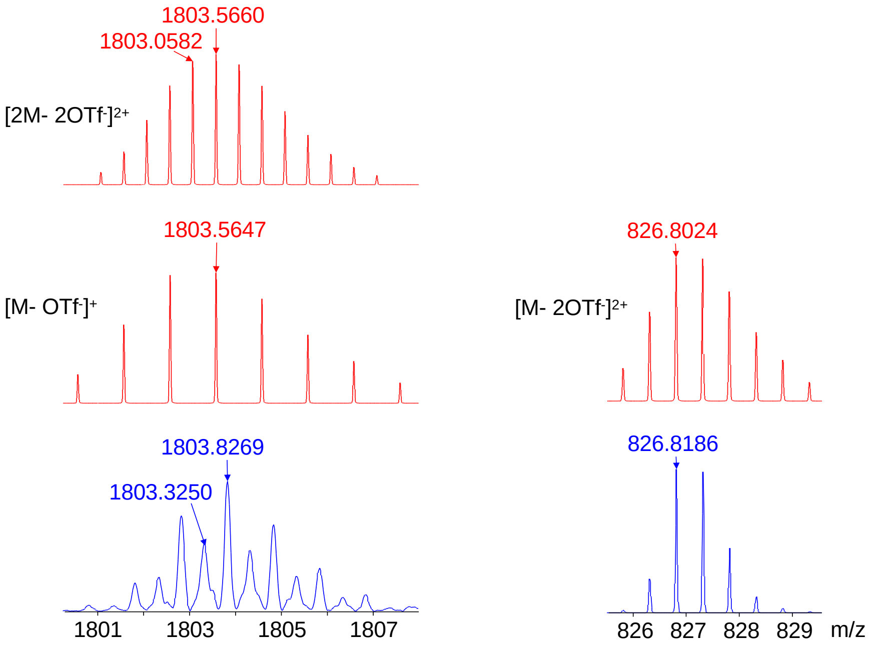

Supplementary Figure 135. Theoretical (red) and experimental (blue) ESI-TOF-MS spectra of ligand **L7** upon the addition of 2eq. ligand **10**.

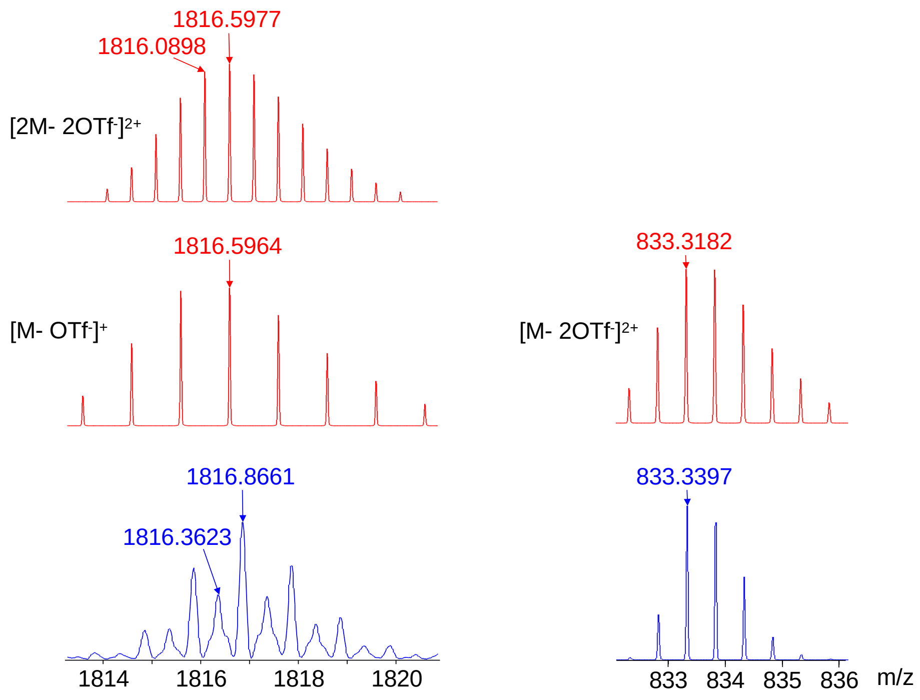

Supplementary Figure 136. Theoretical (red) and experimental (blue) ESI-TOF-MS spectra of ligand **L9** upon the addition of 2eq. ligand **10**.

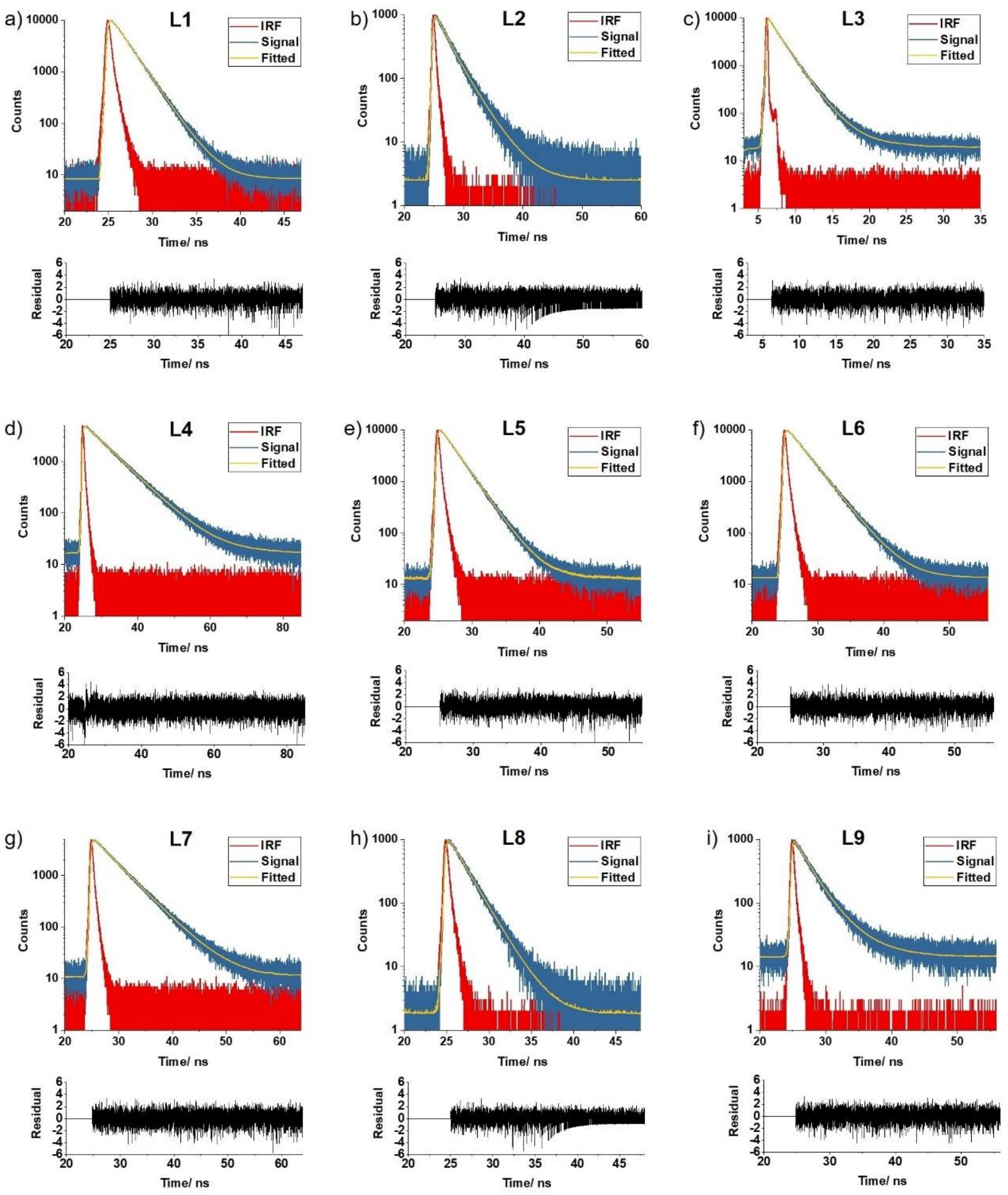

Supplementary Figure 137. Time-resolved fluorescence decay curves of ligands **L1** (a, at 420 nm), **L2** (b, at 450 nm), **L3** (c, at 560 nm), **L4** (d, at 485 nm), **L5** (e, at 440 nm), **L6** (f, at 450 nm), **L7** (g, at 475 nm), **L8** (h, at 410 nm), and **L9** (i, at 445 nm) in DCM (5 μM) in air. Fitted by convoluting the IRF from the scattering of SiO_2 nanoparticles.

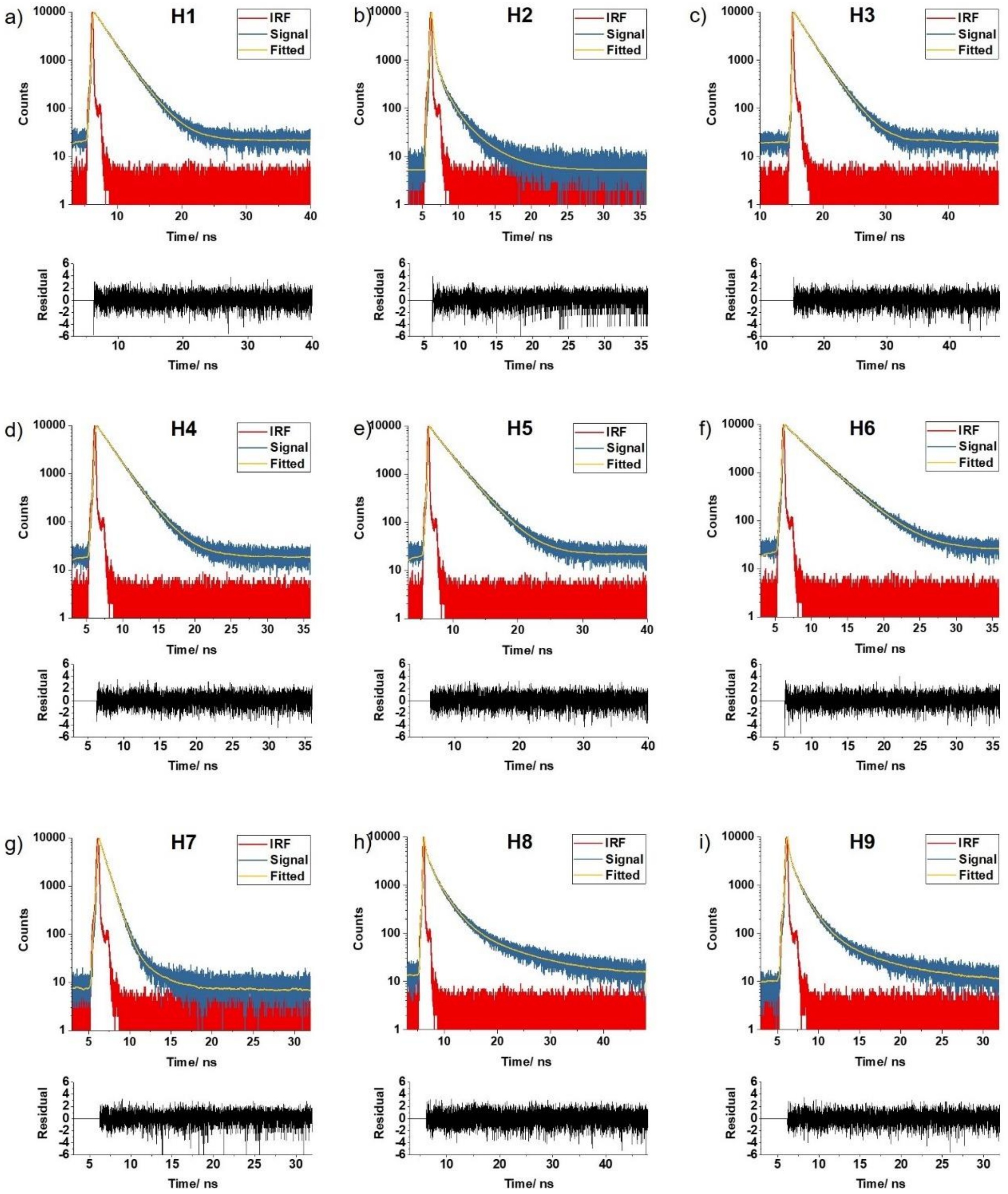

Supplementary Figure 138. Time-resolved fluorescence decay curves of metallacycles **H1** (a, at 520 nm), **H2** (b, at 580 nm), **H3** (c, at 590 nm), **H4** (d, at 520 nm), **H5** (e, at 520 nm), **H6** (f, at 540 nm), **H7** (g, at 520 nm), **H8** (h, at 500 nm), and **H9** (i, at 520 nm) in DCM (5 μM) in air. Fitted by convoluting the IRF from the scattering of SiO_2 nanoparticles.

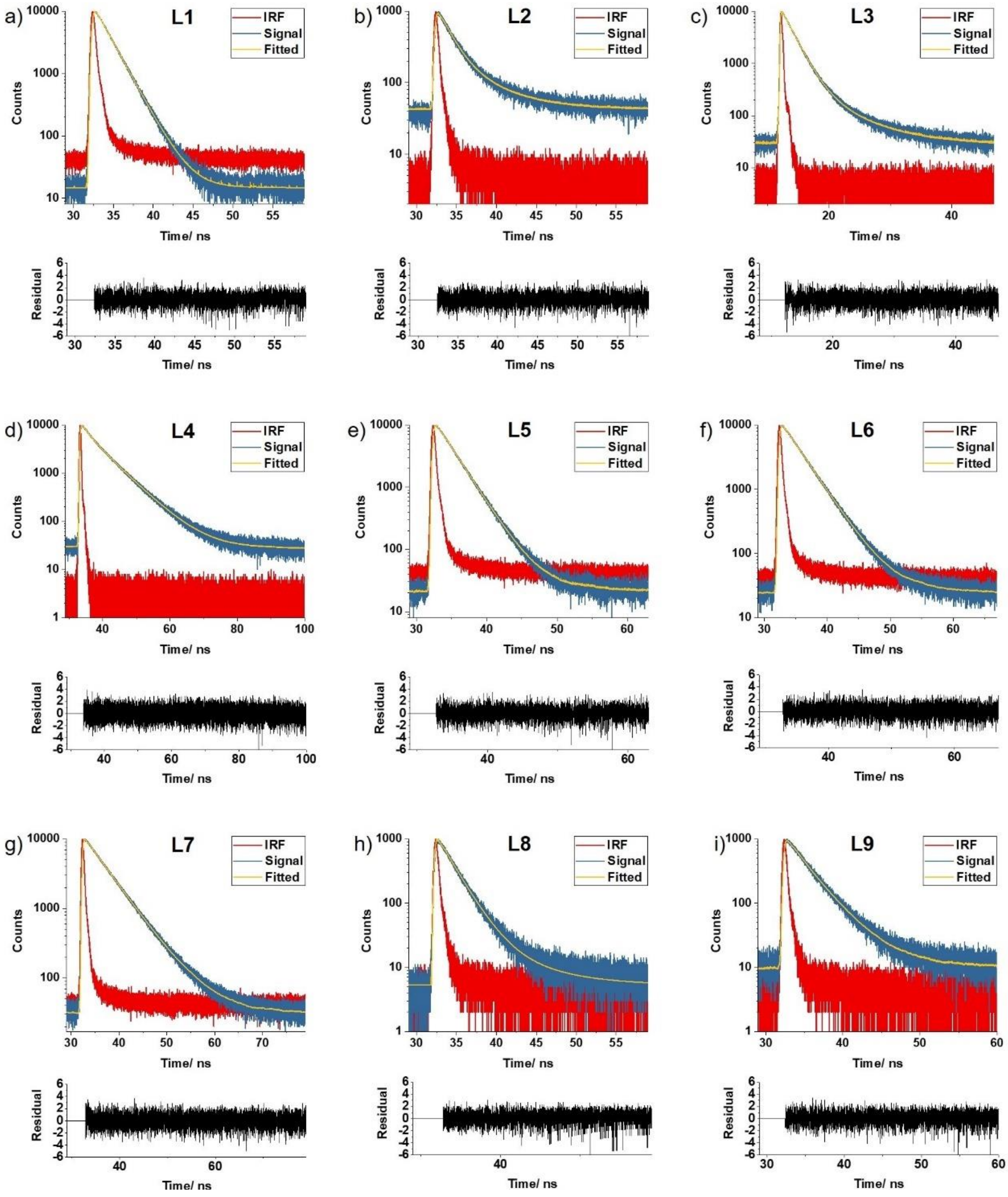

Supplementary Figure 139. Time-resolved fluorescence decay curves of ligands **L1** (a, at 420 nm), **L2** (b, at 450 nm), **L3** (c, at 560 nm), **L4** (d, at 485 nm), **L5** (e, at 440 nm), **L6** (f, at 450 nm), **L7** (g, at 475 nm), **L8** (h, at 410 nm), and **L9** (i, at 445 nm) in DCM (5 μM) under N_2 atmosphere. Fitted by convoluting the IRF from the scattering of SiO_2 nanoparticles.

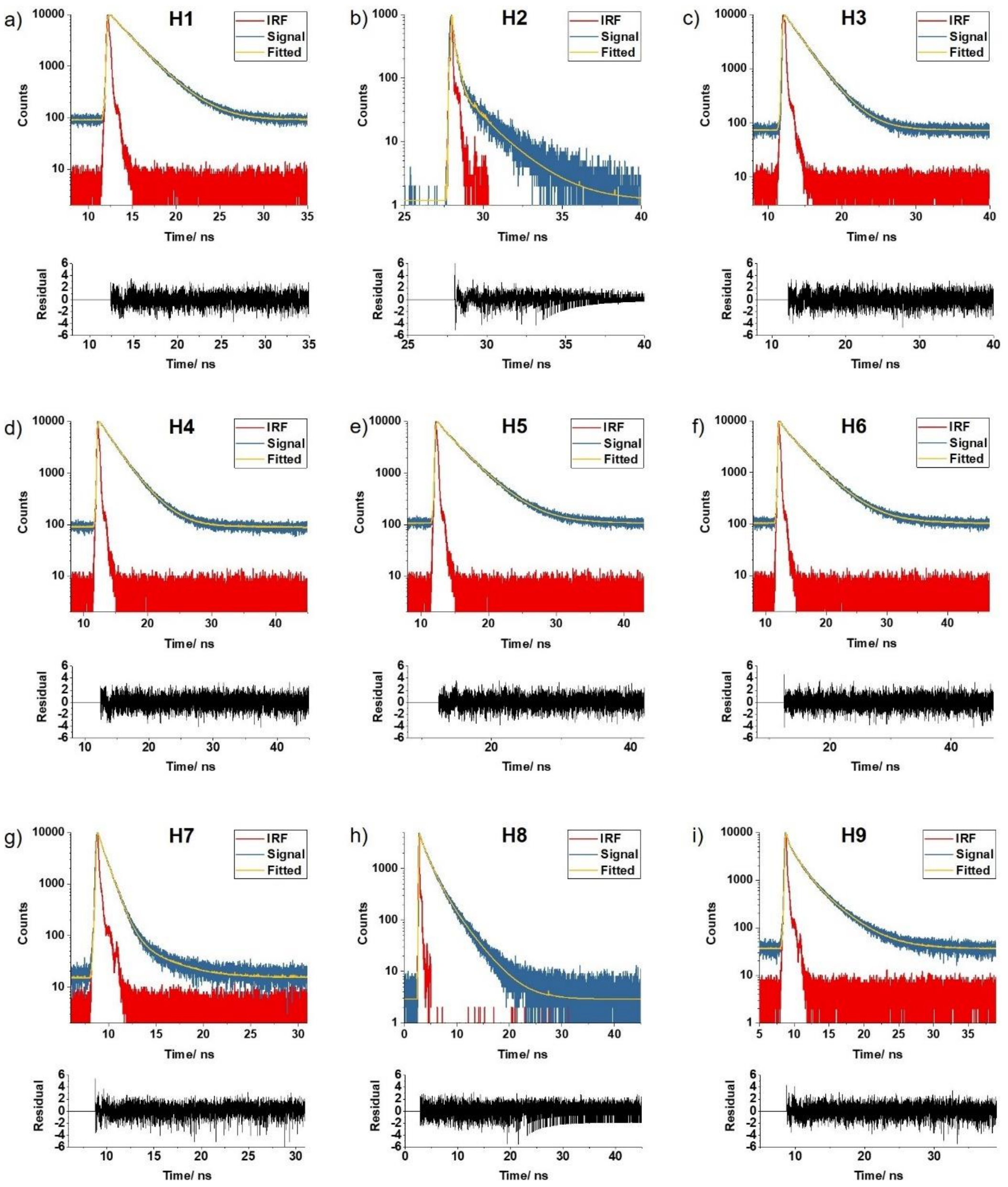

Supplementary Figure 140. Time-resolved fluorescence decay curves of metallacycles **H1** (a, at 520 nm), **H2** (b, at 580 nm), **H3** (c, at 590 nm), **H4** (d, at 520 nm), **H5** (e, at 520 nm), **H6** (f, at 540 nm), **H7** (g, at 520 nm), **H8** (h, at 500 nm), and **H9** (i, at 520 nm) in DCM (5 μM) under N_2 atmosphere. Fitted by convoluting the IRF from the scattering of SiO_2 nanoparticles.

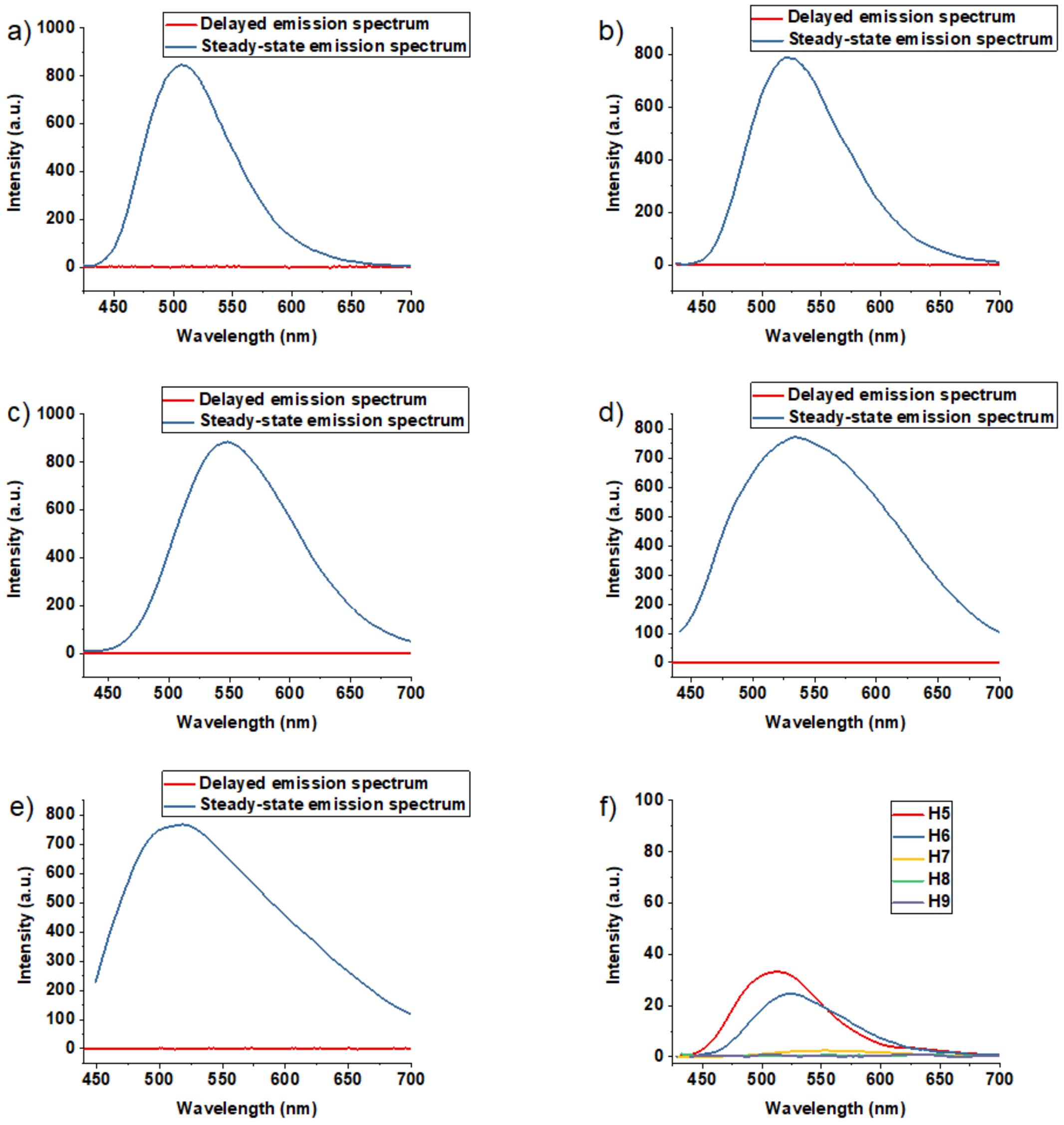

Supplementary Figure 141. The emission spectra of metallacycles (a) **H5**, (b) **H6**, (c) **H7**, (d) **H8**, and (e) **H9** (2 μM in DCM, excitation at the absorbance maxima). Blue line: Steady-state emission spectra (delay time, 0 s). Red line: delayed emission spectra. (f) Delayed emission spectra of metallacycles **H5-H9** (2 μM in DCM, excitation at the absorbance maxima) in air. Delayed emission spectra were monitored in phosphorescence mode (total decay time, 20 ms; delay time, 0.1 ms; gate time, 2.0 ms).

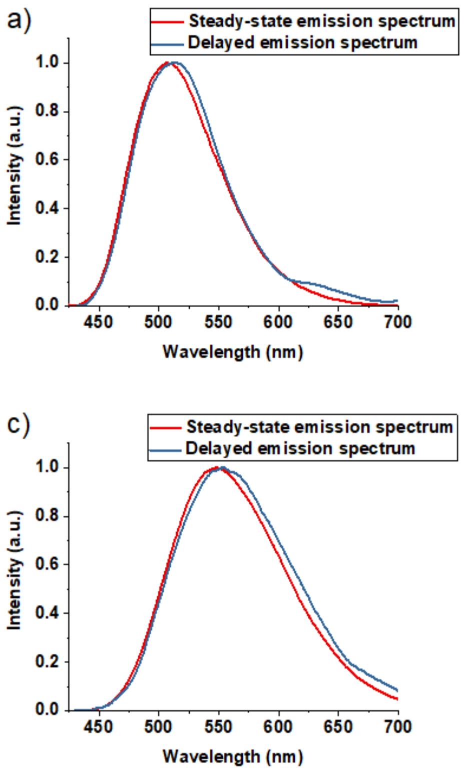

Supplementary Figure 142. Normalized emission spectra of metallacycles (a) **H5**, (b) **H6**, (c) **H7** (2 μM in DCM, excitation at the absorbance maxima). Red line: steady-state emission spectra (delay time, 0 s). Blue line: delayed emission spectra. Delayed emission spectra were monitored in phosphorescence mode (total decay time, 20 ms; delay time, 0.1 ms; gate time, 2.0 ms).

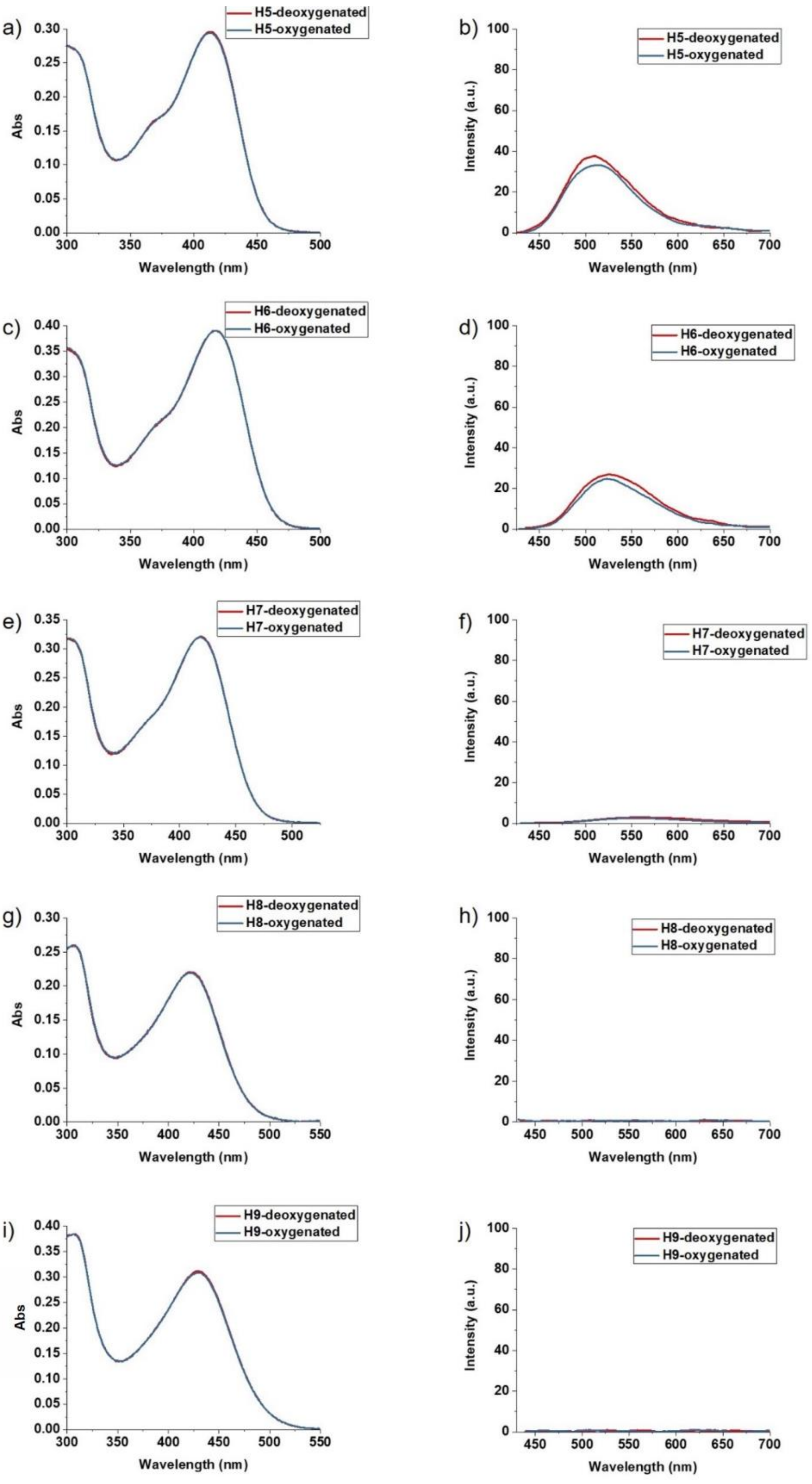

Supplementary Figure 143. Absorption spectra (a, c, e, g, i) and delayed emission spectra (b, d, f, h, j) of metallacycles **H5-H9** (2 μM in DCM, excitation at the absorbance maxima) in air (blue line), and under N_2 atmosphere (red line). All detections were carried out in phosphorescence mode (total decay time, 20 ms; delay time, 0.1 ms; gate time, 2.0 ms).

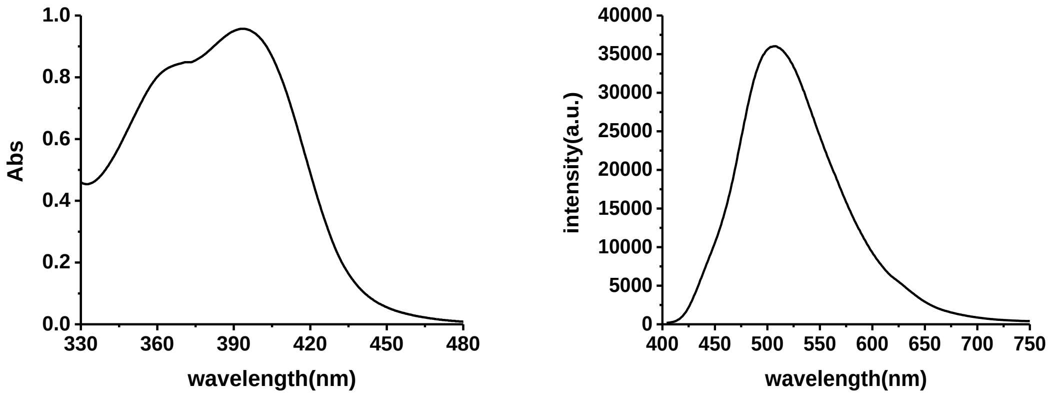

Supplementary Figure 144. Absorption (left), emission (right, λ_{exc} = 393 nm) spectra of thin film doped with 0.1 wt% of metallacycle **H1**.

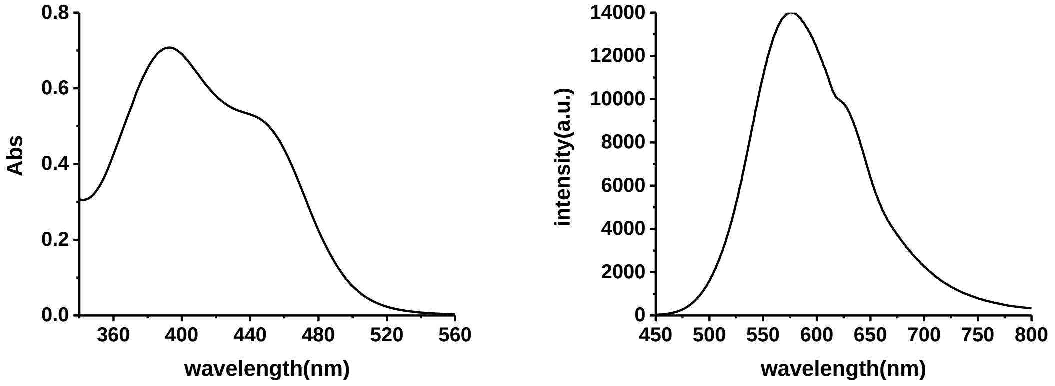

Supplementary Figure 145. Absorption (left), emission (right, λ_{exc} = 442 nm) spectra of thin film doped with 0.1 wt% of metallacycle **H3**.

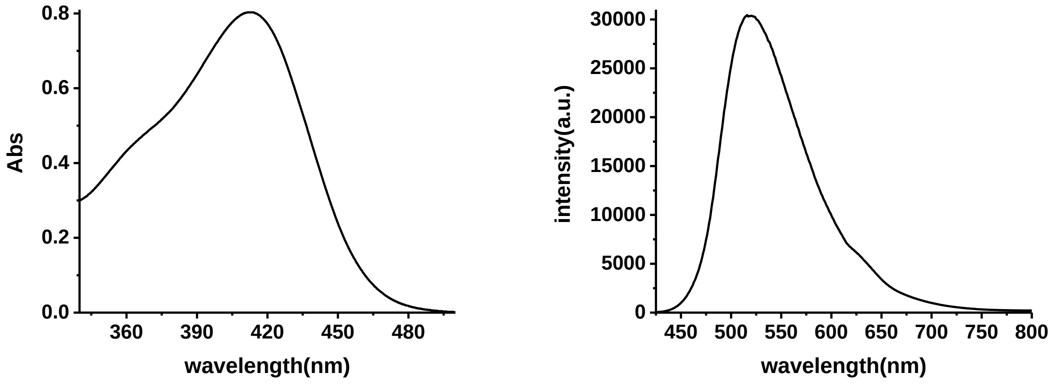

Supplementary Figure 146. Absorption (left), emission (right, λ_{exc} =413 nm) spectra of thin film doped with 0.1 wt% of metallacycle **H7**.

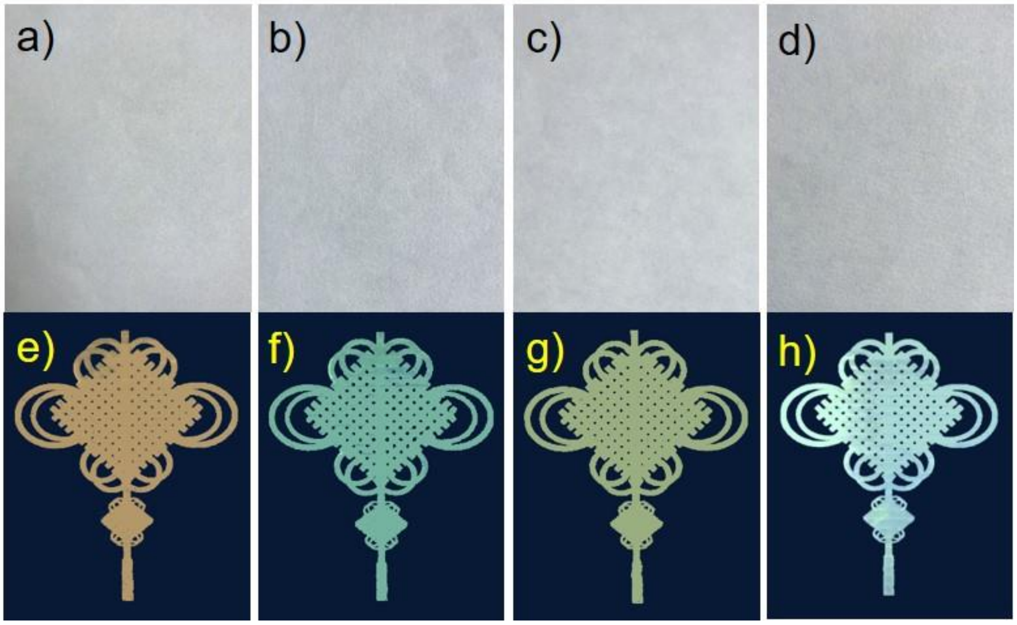

Supplementary Figure 147. Photographs of the fluorescent patterns by inkjet printing from complex **C3** in visible light (a) and under 365 nm light excitation (e), complex **C4** in visible light (b) and under 365 nm light excitation (f), ligand **L5** in visible light (c) and under 365 nm light excitation (g), and ligand **L6** in visible light (d) and under 365 nm light excitation (h).

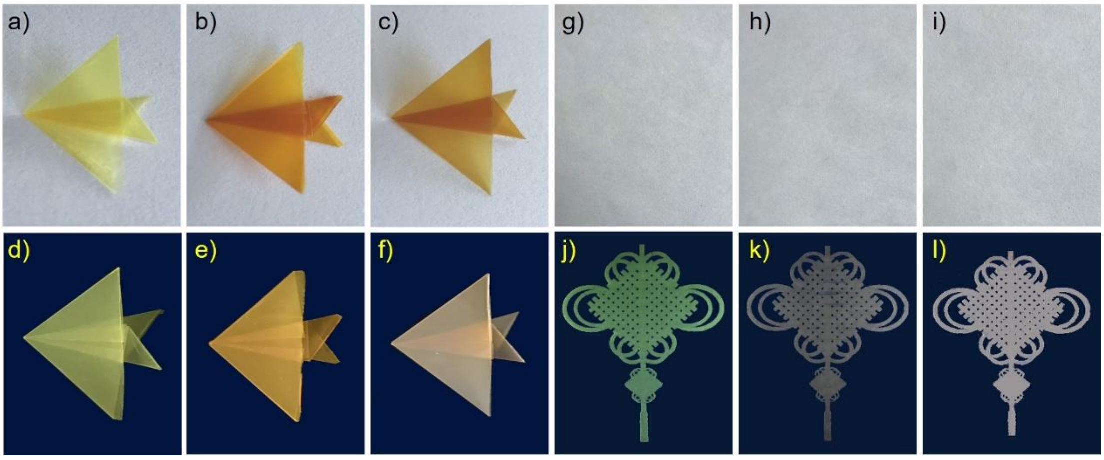

Supplementary Figure 148. Photographs of the flexible fluorescent films (a-f) and the fluorescent patterns (g-l) by inkjet printing from ligand **L1** in visible light (a, g) and under 365 nm light excitation (d, j), ligand **L3** in visible light (b, h) and under 365 nm light excitation (e, k), ligand **L7** in visible light (c, i) and under 365 nm light excitation (f, l).

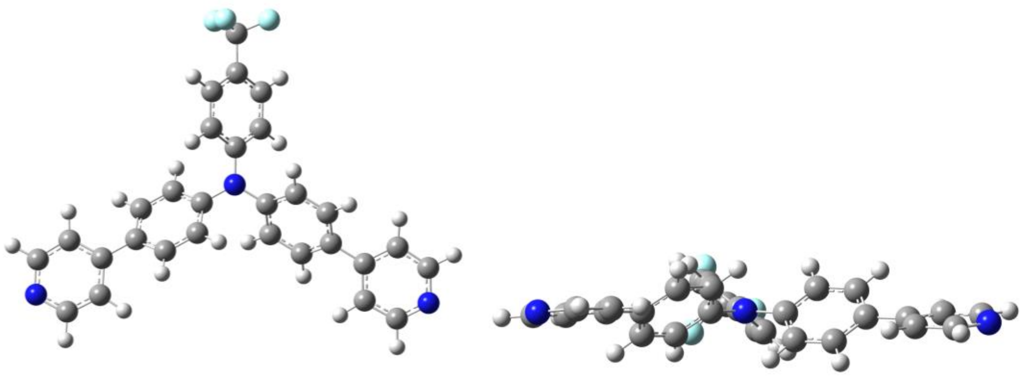

Supplementary Figure 149. Geometric structure of ligand **L1** optimized by the density functional theoretical (DFT) method.

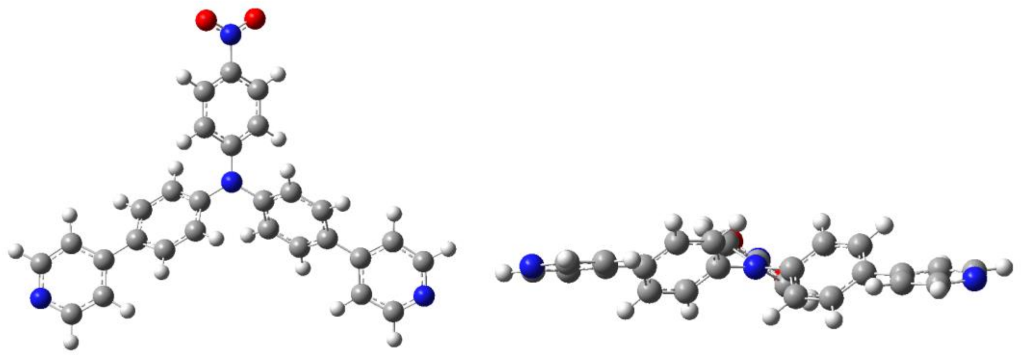

Supplementary Figure 150. Geometric structure of ligand **L2** optimized by the density functional theoretical (DFT) method.

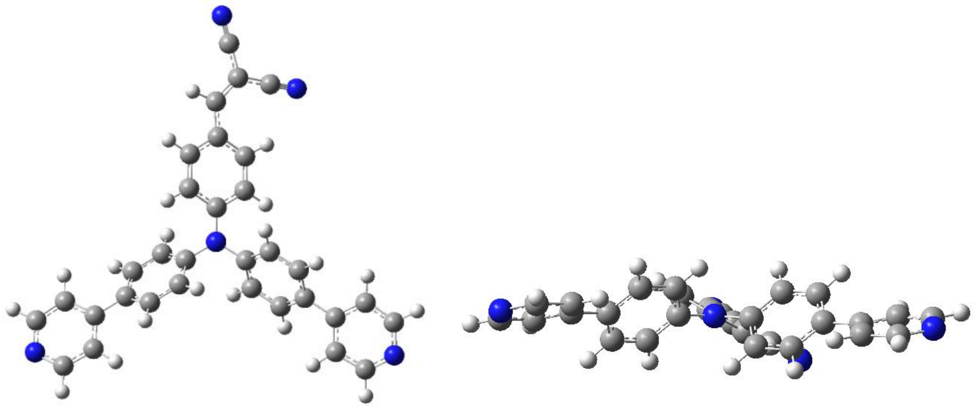

Supplementary Figure 151. Geometric structure of ligand **L3** optimized by the density functional theoretical (DFT) method.

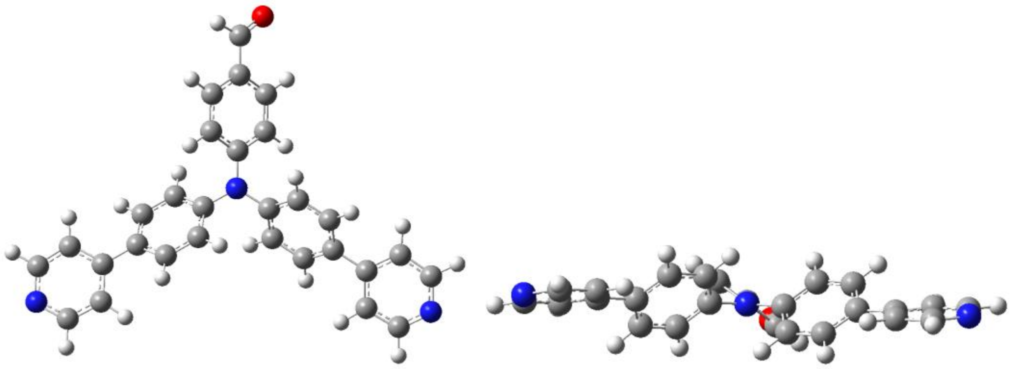

Supplementary Figure 152. Geometric structure of ligand **L4** optimized by the density functional theoretical (DFT) method.

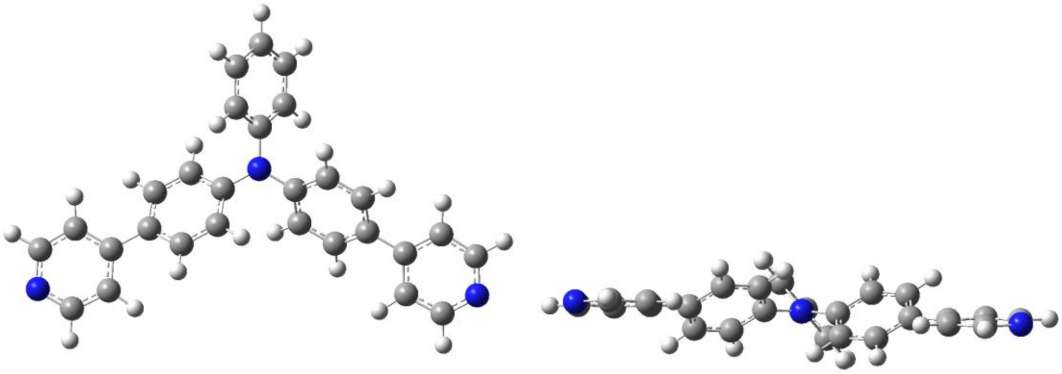

Supplementary Figure 153. Geometric structure of ligand **L5** optimized by the density functional theoretical (DFT) method.

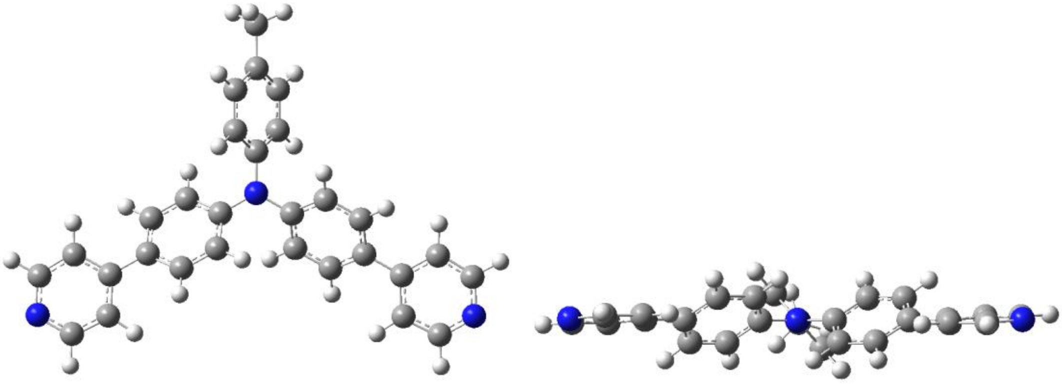

Supplementary Figure 154. Geometric structure of ligand **L6** optimized by the density functional theoretical (DFT) method.

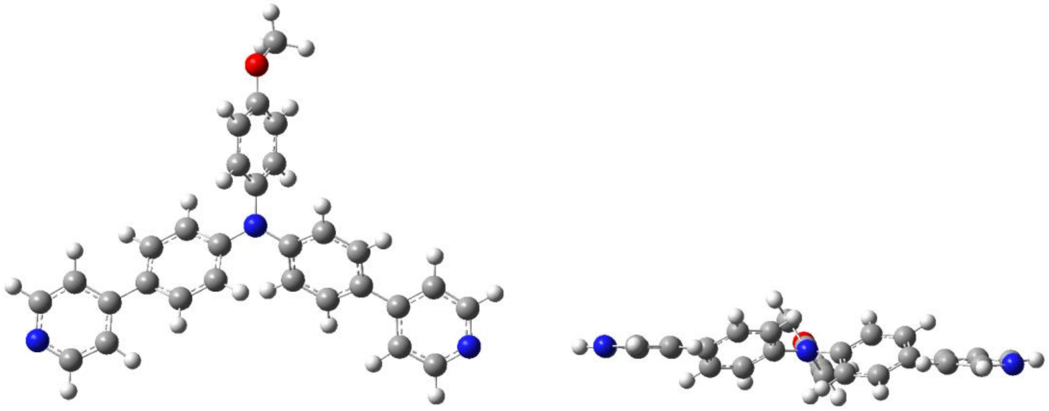

Supplementary Figure 155. Geometric structure of ligand **L7** optimized by the density functional theoretical (DFT) method.

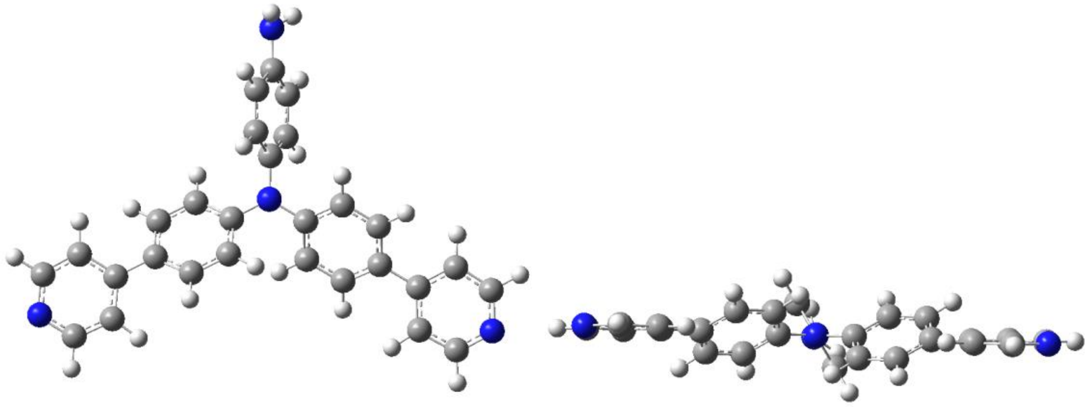

Supplementary Figure 156. Geometric structure of ligand **L8** optimized by the density functional theoretical (DFT) method.

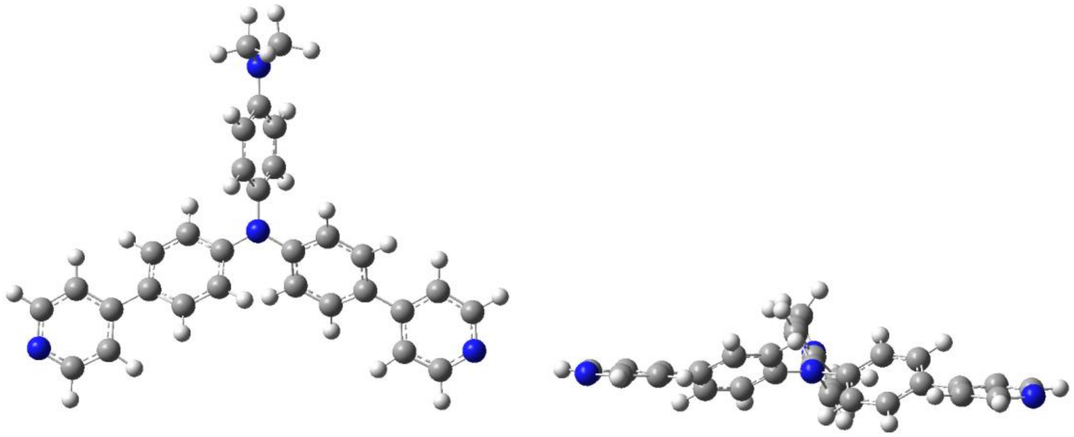

Supplementary Figure 157. Geometric structure of ligand **L9** optimized by the density functional theoretical (DFT) method.

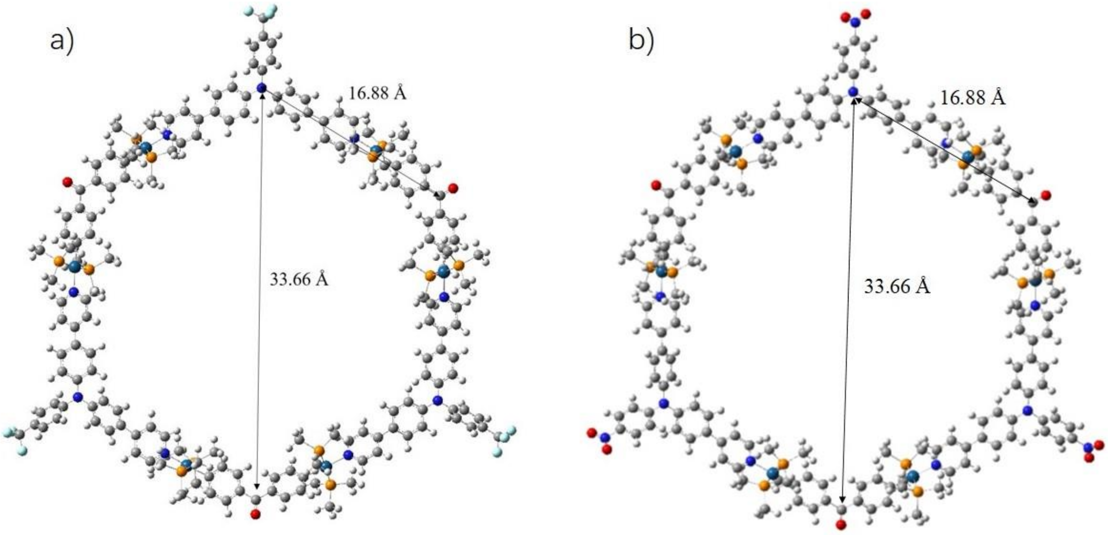

Supplementary Figure 158. Geometric structure of metallacycles a) **H1** and b) **H2** optimized by the PM6 semiempirical molecular orbital method.

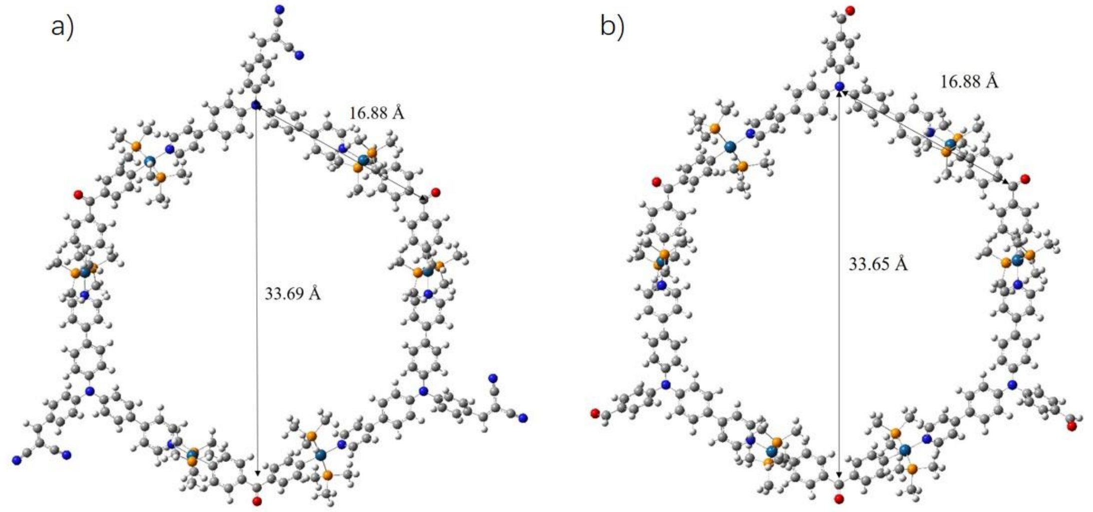

Supplementary Figure 159. Geometric structure of metallacycles a) **H3** and b) **H4** optimized by the PM6 semiempirical molecular orbital method.

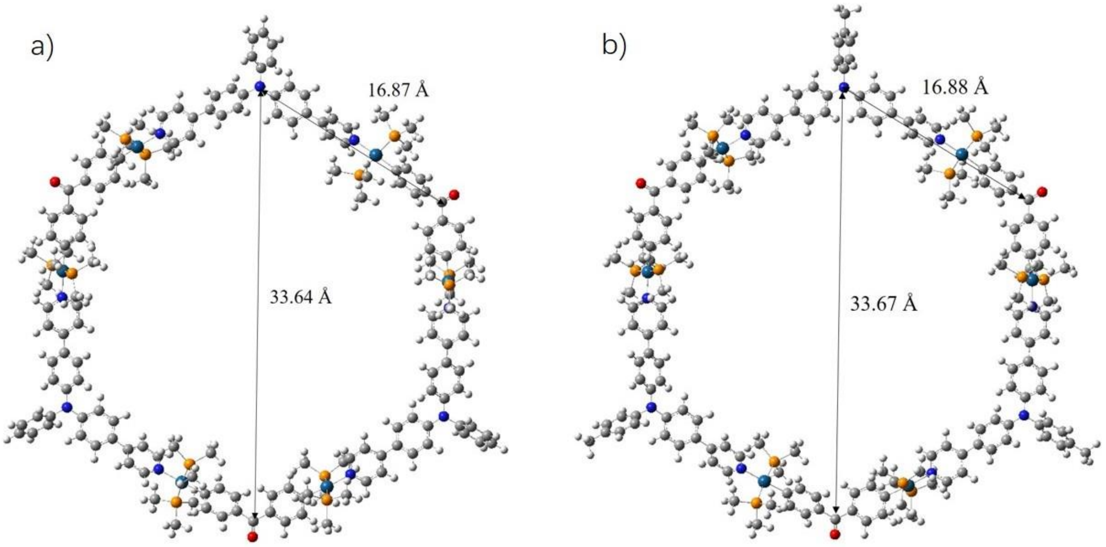

Supplementary Figure 160. Geometric structure of metallacycles a) **H5** and b) **H6** optimized by the PM6 semiempirical molecular orbital method.

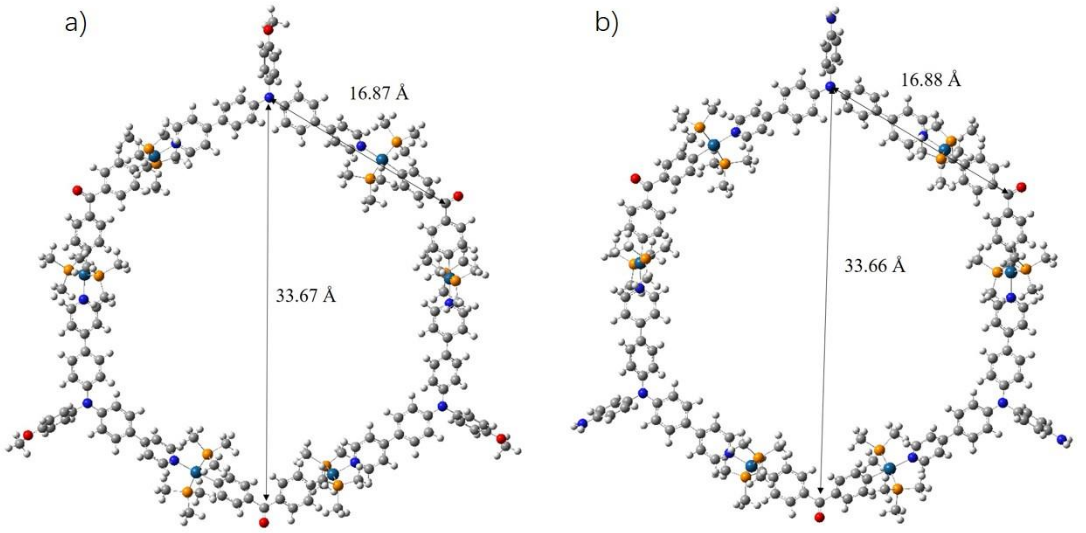

Supplementary Figure 161. Geometric structure of metallacycles a) **H7** and b) **H8** optimized by the PM6 semiempirical molecular orbital method.

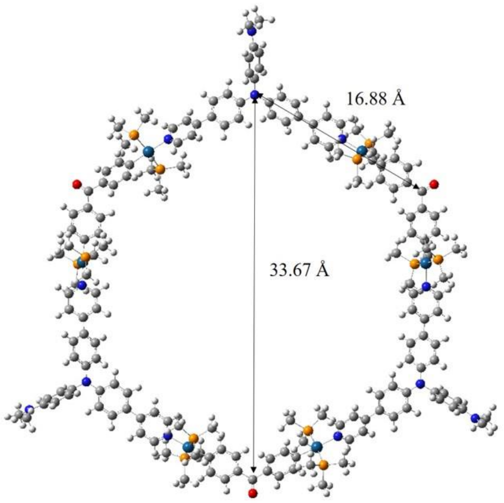

Supplementary Figure 162. Geometric structure of metallacycle **H9** optimized by the PM6 semiempirical molecular orbital method.

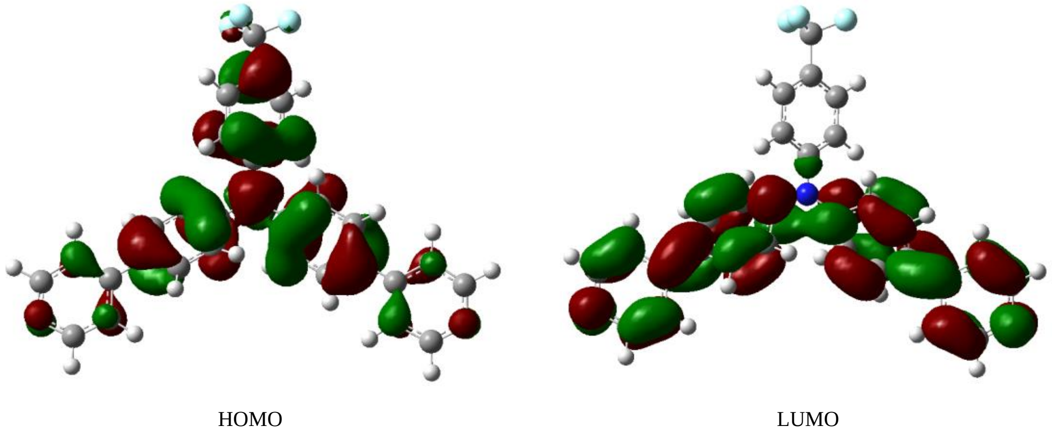

Supplementary Figure 163. Frontier orbital of ligand **L1**.

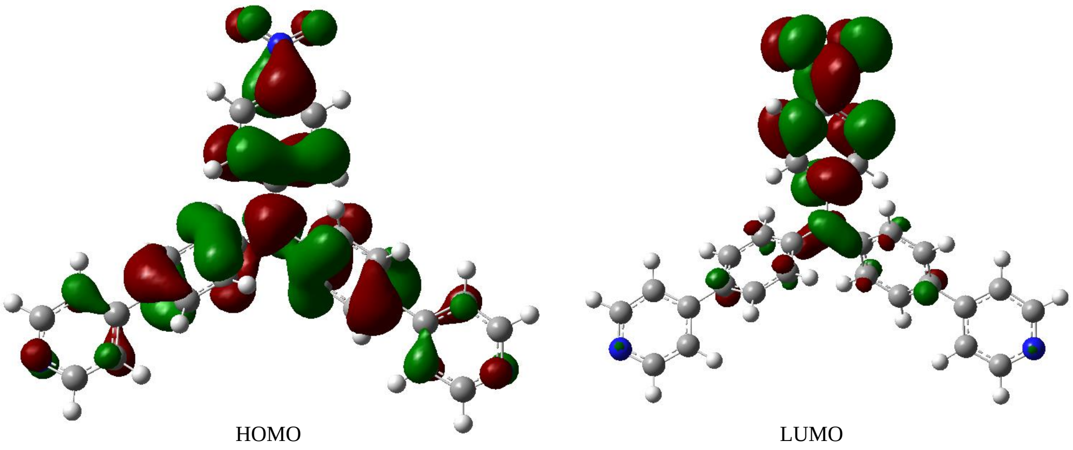

Supplementary Figure 164. Frontier orbital of ligand **L2**.

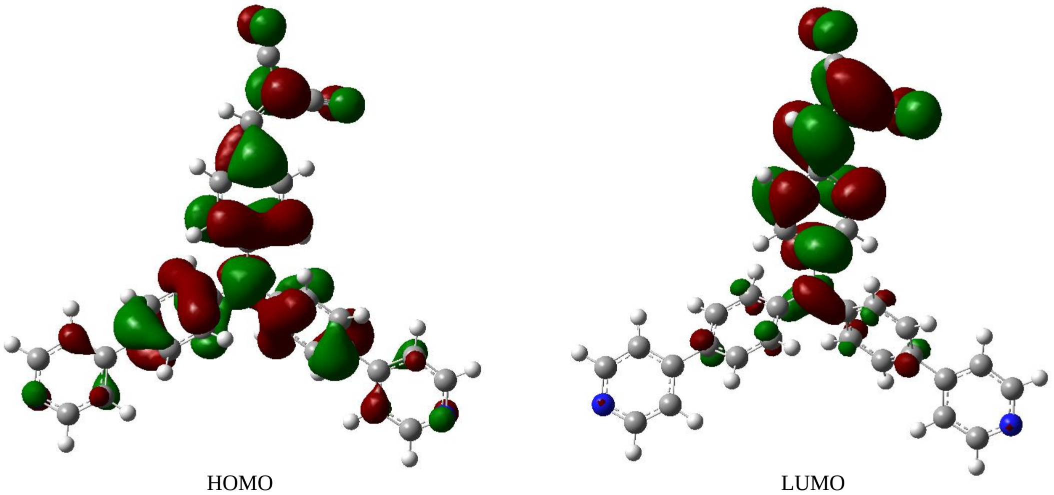

HOMO LUMO

Supplementary Figure 165. Frontier orbital of ligand **L3**.

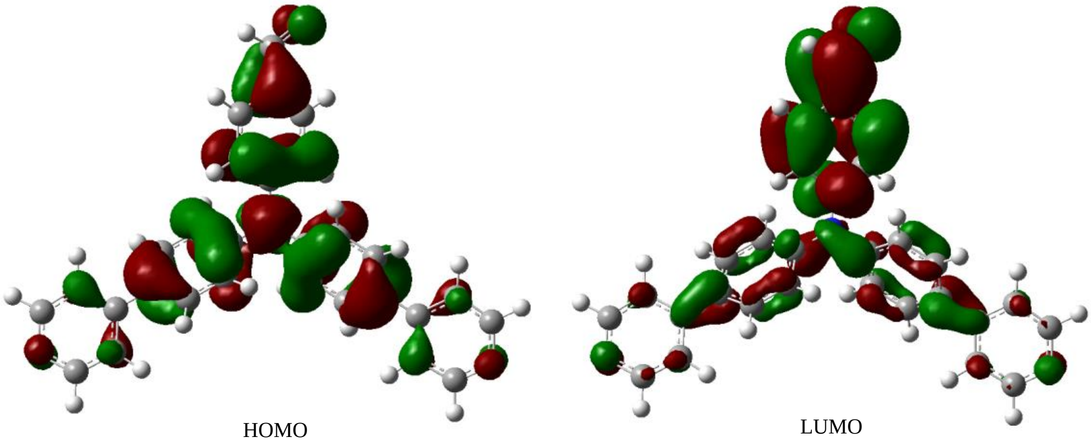

HOMO LUMO

Supplementary Figure 166. Frontier orbital of ligand **L4**.

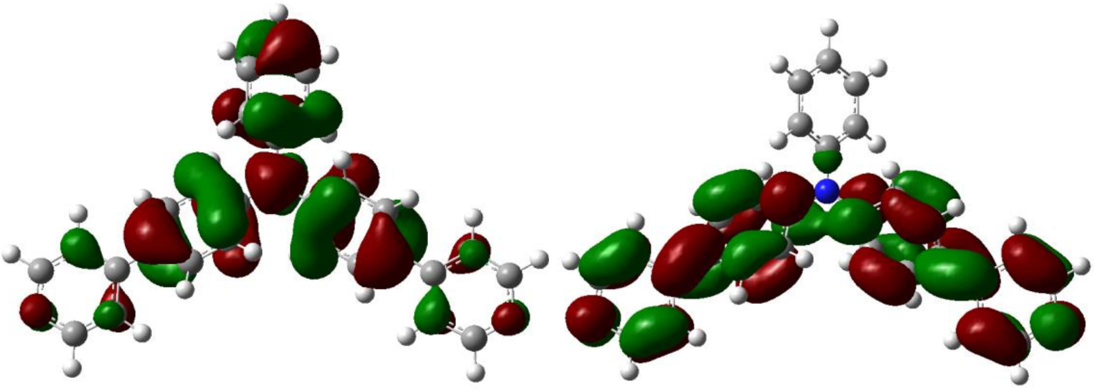

HOMO LUMO

Supplementary Figure 167. Frontier orbital of ligand **L5**.

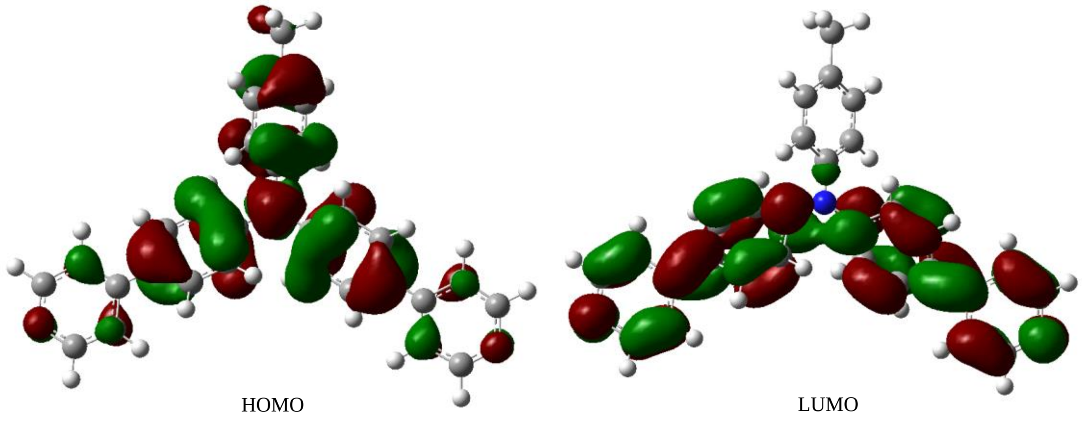

Supplementary Figure 168. Frontier orbital of ligand **L6**.

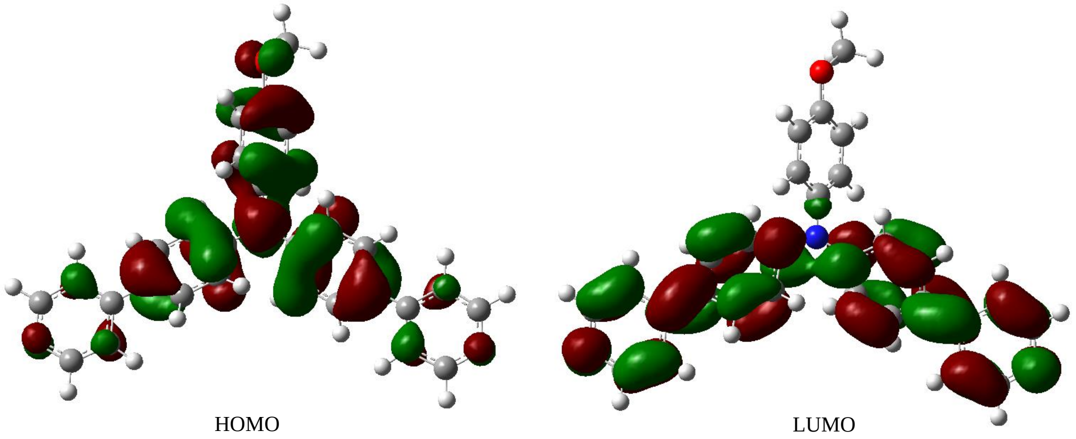

Supplementary Figure 169. Frontier orbital of ligand **L7**.

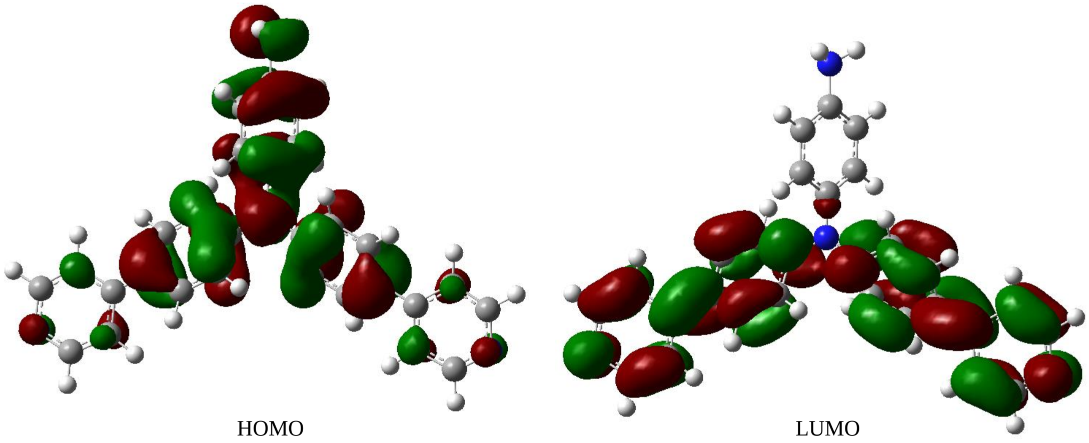

Supplementary Figure 170. Frontier orbital of ligand **L8**.

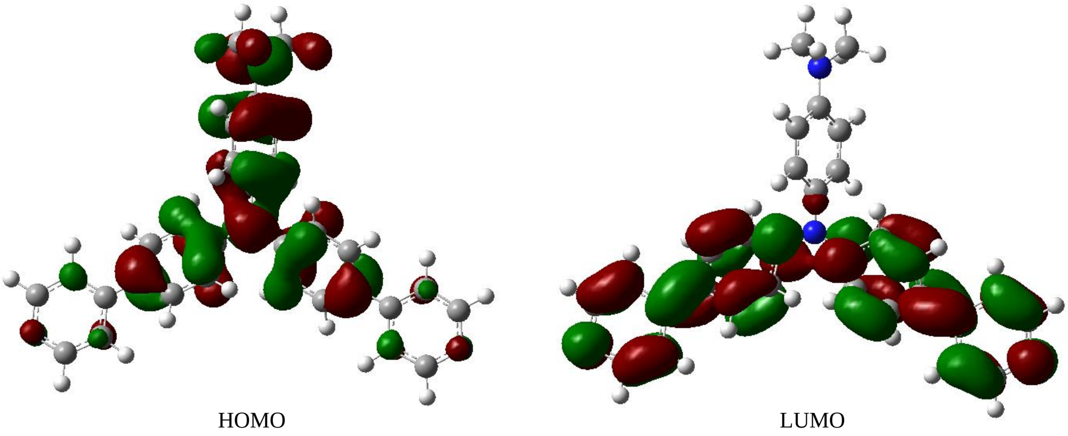

Supplementary Figure 171. Frontier orbital of ligand **L9**.

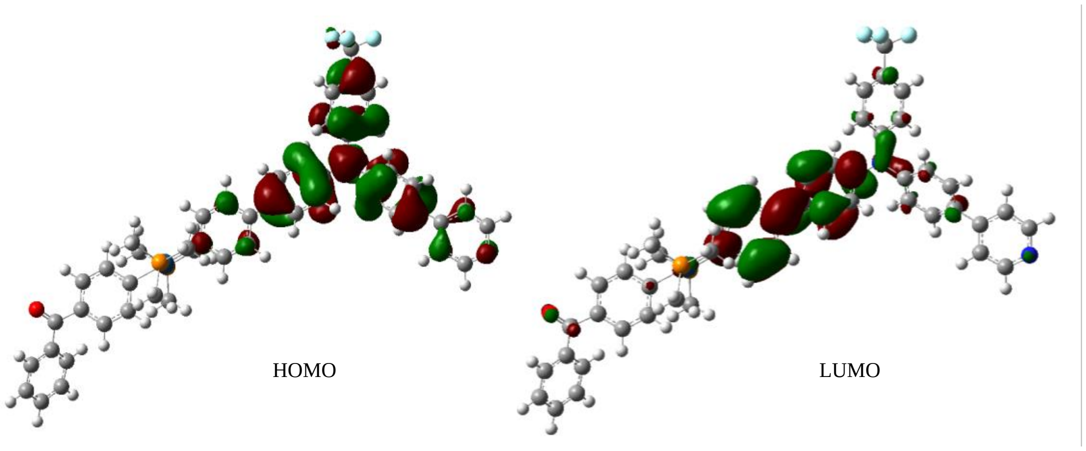

Supplementary Figure 172. Frontier orbital of metallacycle **H1** (1/3).

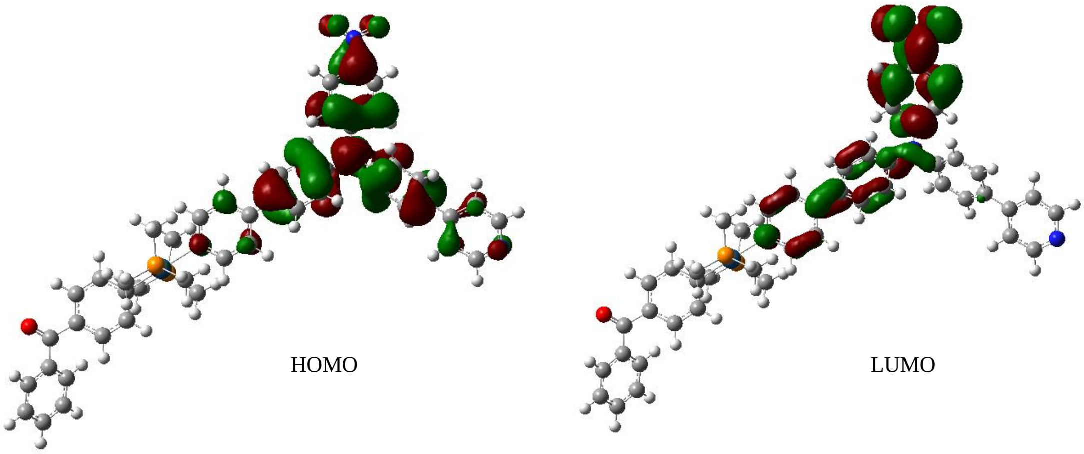

Supplementary Figure 173. Frontier orbital of metallacycle **H2** (1/3).

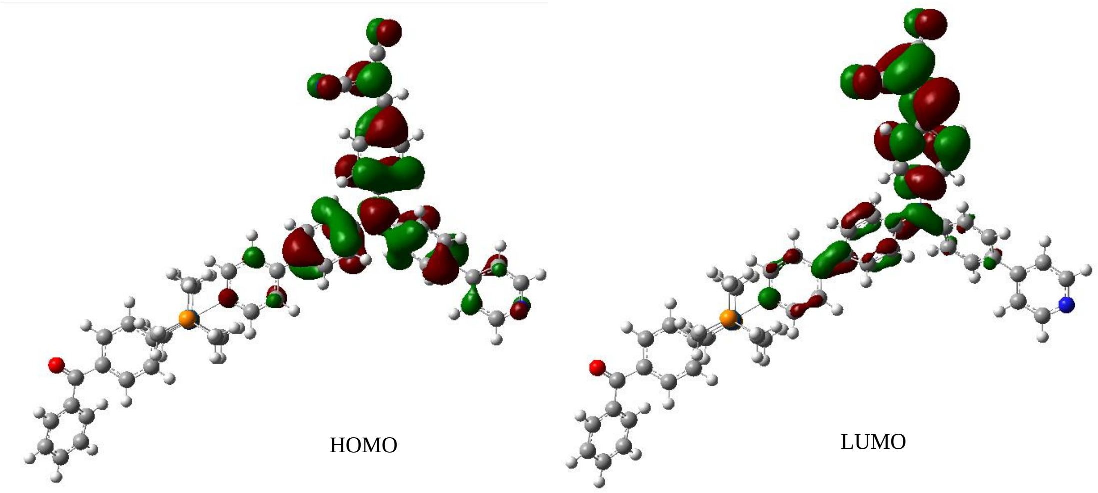

Supplementary Figure 174. Frontier orbital of metallacycle **H3** (1/3).

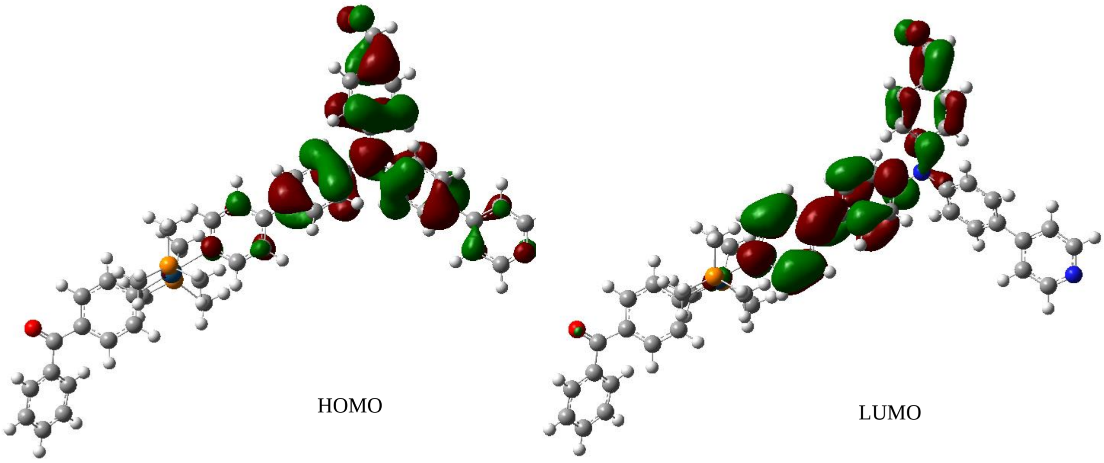

Supplementary Figure 175. Frontier orbital of metallacycle **H4** (1/3).

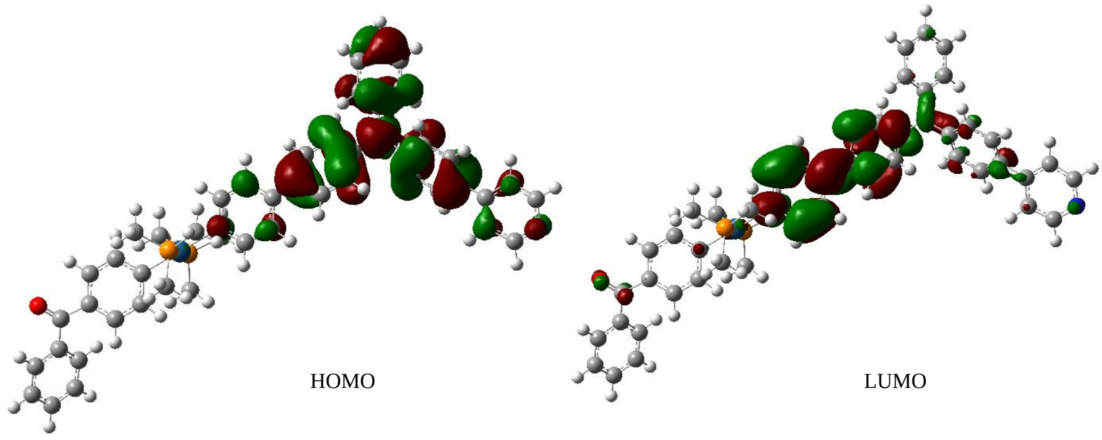

Supplementary Figure 176. Frontier orbital of metallacycle **H5** (1/3).

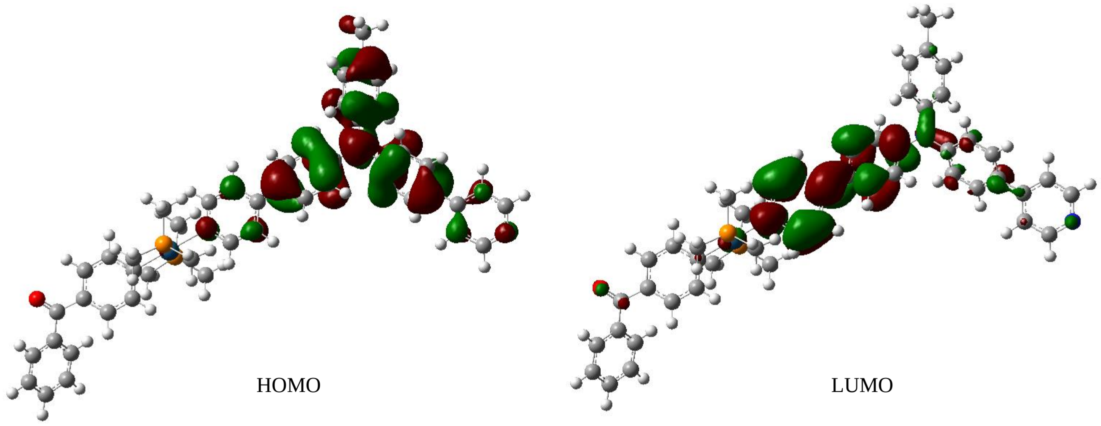

Supplementary Figure 177. Frontier orbital of metallacycle **H6** (1/3).

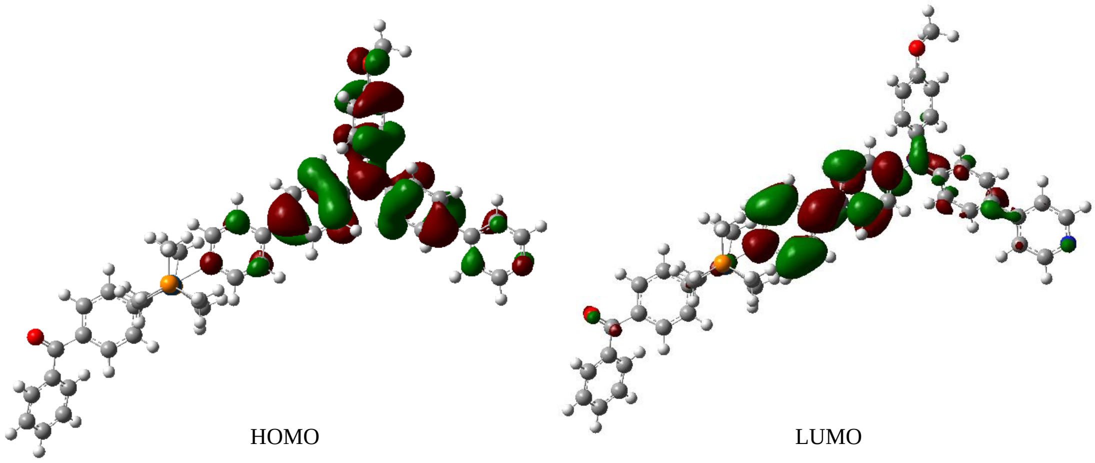

Supplementary Figure 178. Frontier orbital of metallacycle **H7** (1/3).

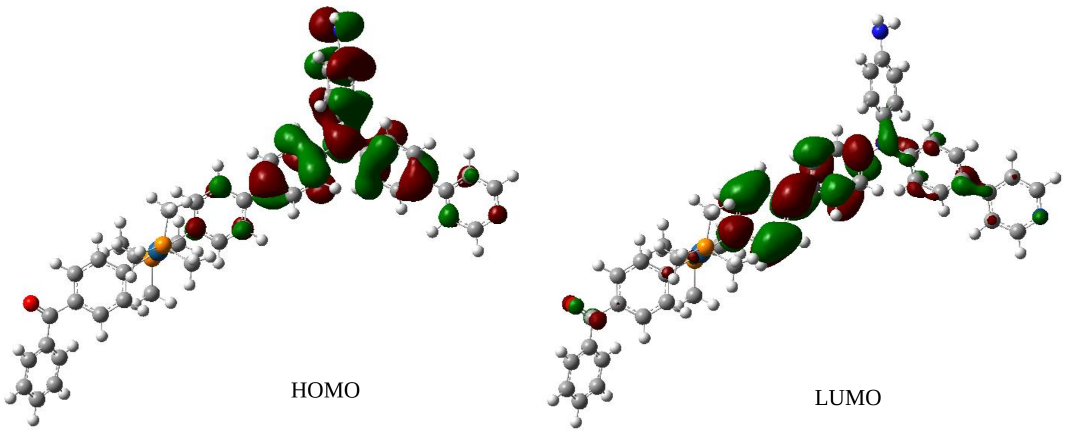

Supplementary Figure 179. Frontier orbital of metallacycle **H8** (1/3).

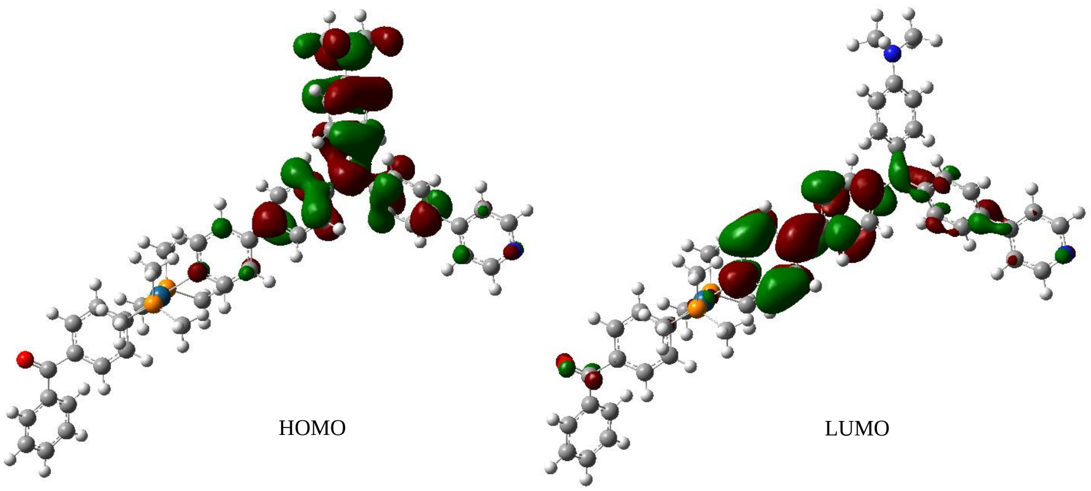

Supplementary Figure 180. Frontier orbital of metallacycle **H9** (1/3).

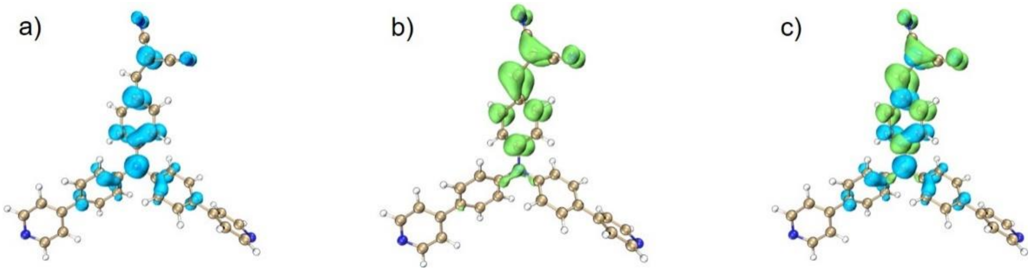

Supplementary Figure 181. Electron-hole distribution of the S1 state for ligand **L3**. (a) The blue colors represent holes; (b) The green colors represent electrons; (c) Overlap of electron-hole for the S1 state of ligand **L3**.

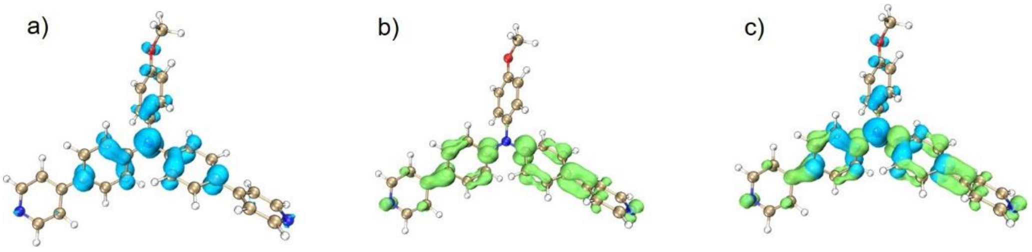

Supplementary Figure 182. Electron-hole distribution of the S1 state for ligand **L7**. (a) The blue colors represent holes; (b) The green colors represent electrons; (c) Overlap of electron-hole for the S1 state of ligand **L7**.

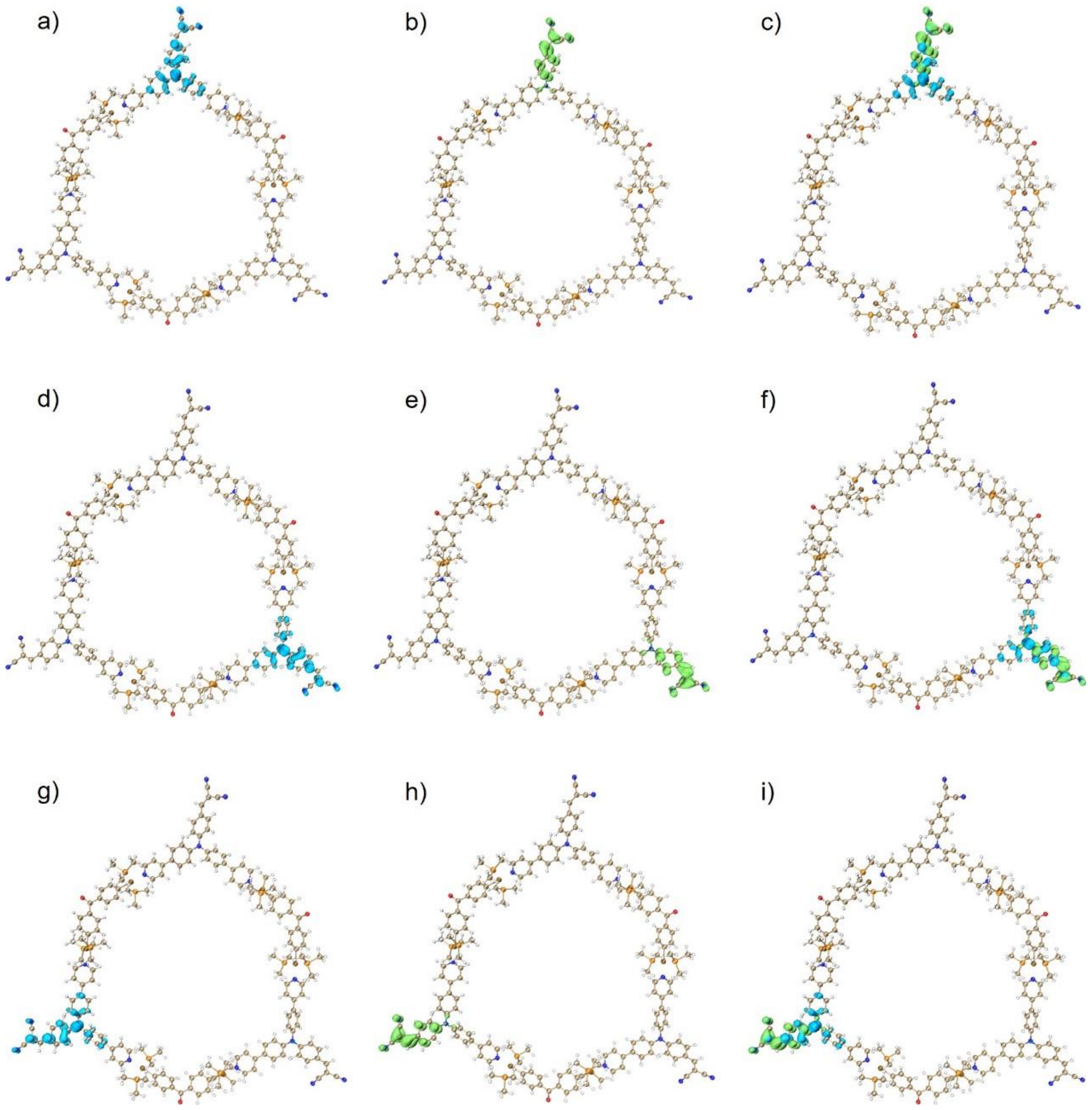

Supplementary Figure 183. Electron-hole distribution of the (a-c) S1, (d-f) S2, and (g-i) S3 states for metallacycle **H3**. The blue colors represent holes, the green colors represent electrons. Overlap of electron-hole for the (c) S1, (f) S2, and (i) S3 states of metallacycle **H3**.

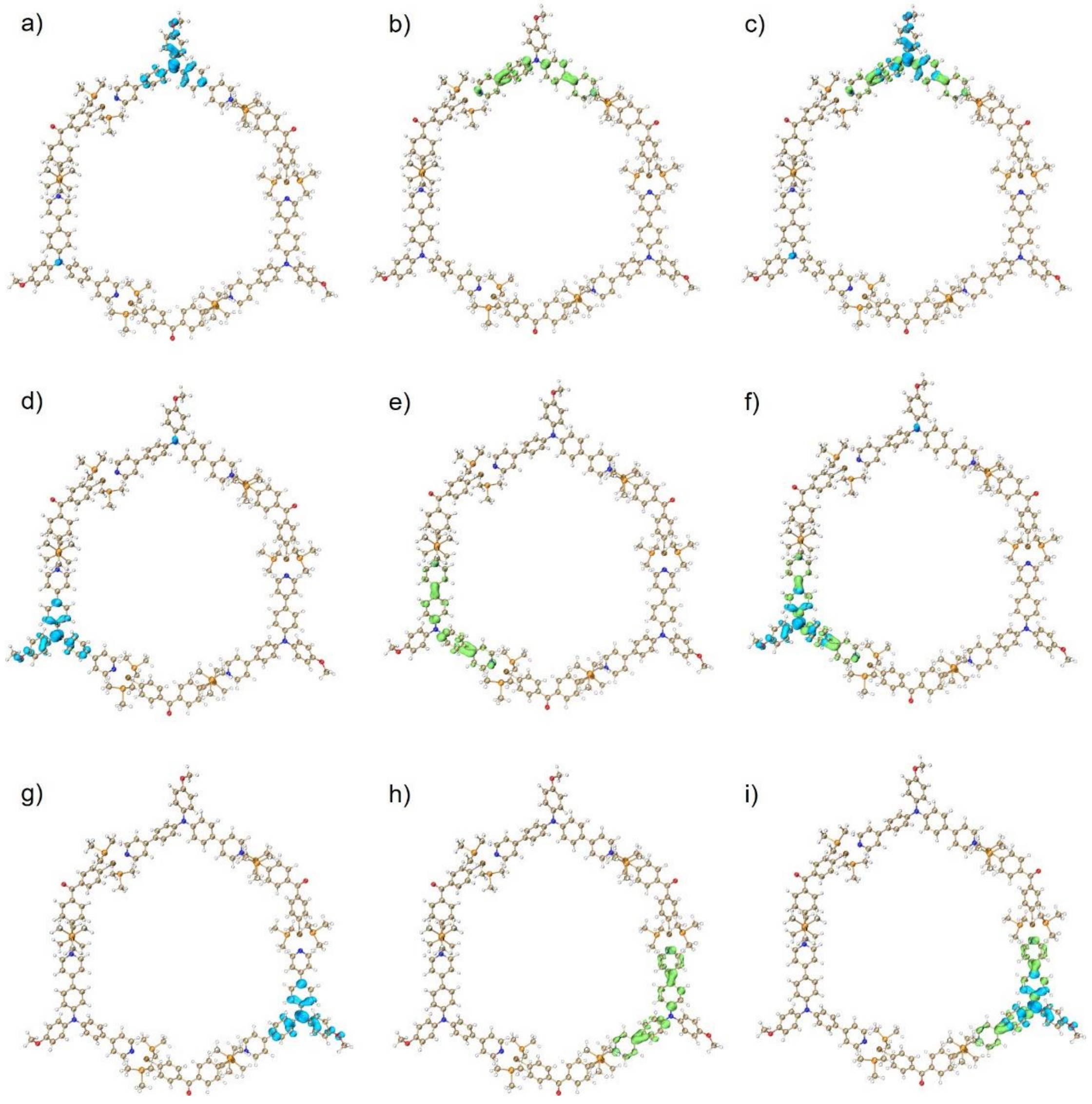

Supplementary Figure 184. Electron-hole distribution of the (a-c) S1, (d-f) S2, and (g-i) S3 states for metallacycle **H7**. The blue colors represent holes, the green colors represent electrons. Overlap of electron-hole for the (c) S1, (f) S2, and (i) S3 states of metallacycle **H7**.

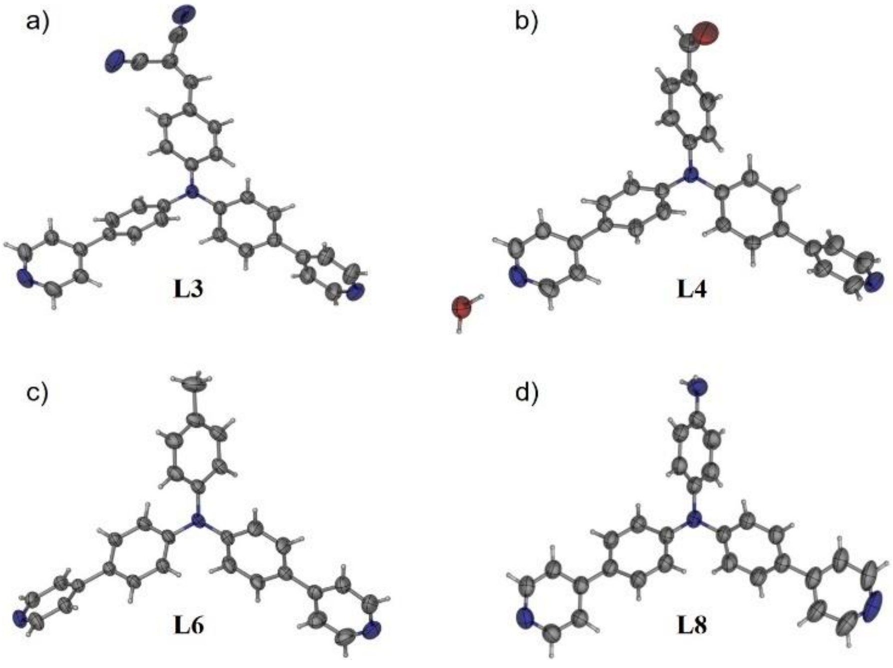

Supplementary Figure 185. Crystal structures of ligands a) **L3**, b) **L4**, c) **L6**, and d) **L8**.

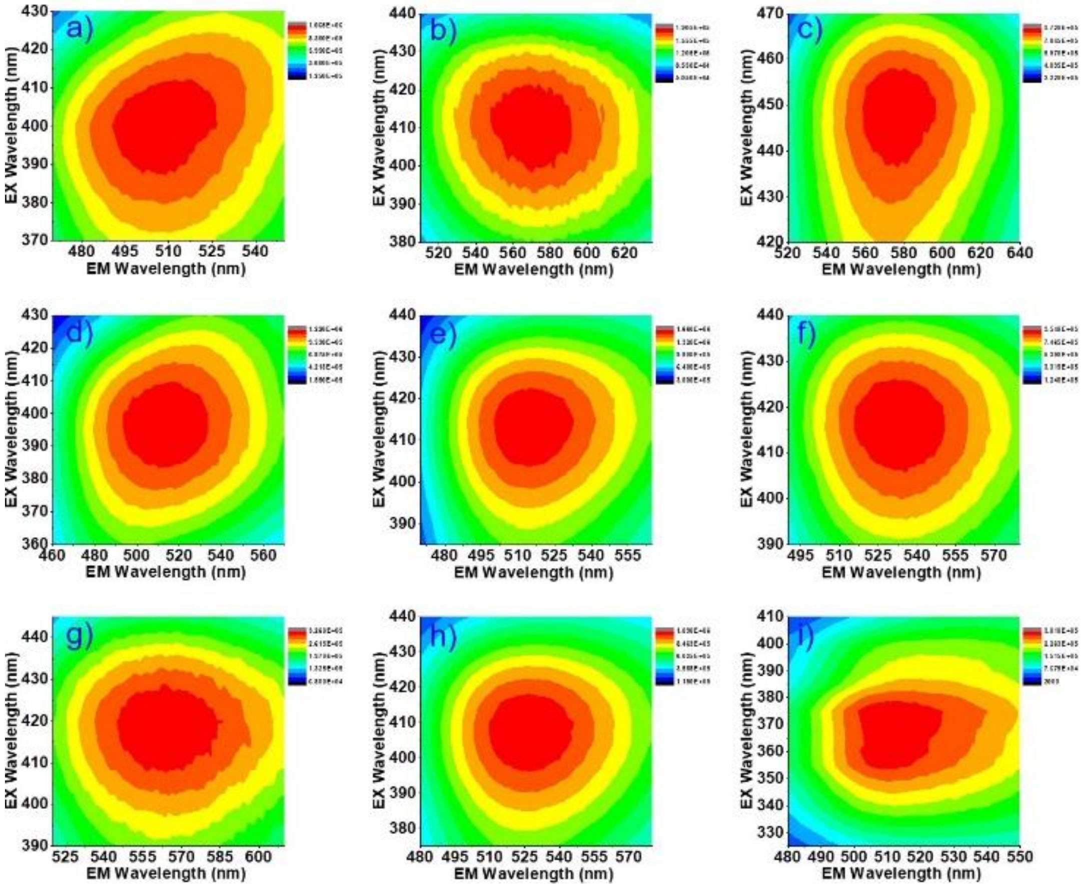

Supplementary Figure 186. 3-D excitation−emission matrix spectra of metallacycles **H1-H9** in dichloromethane (Figure a to Figure i were sequentially corresponded to metallacycles **H1** to **H9**).

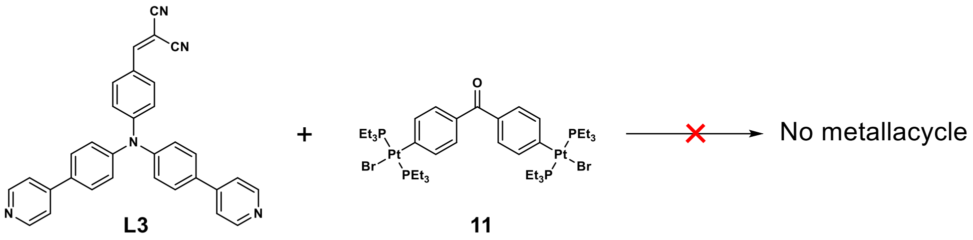

Supplementary Figure 187. The stirring of ligand **L3** and compound **11** in dichloromethane for 6.0 hours cannot form the metallacycle.

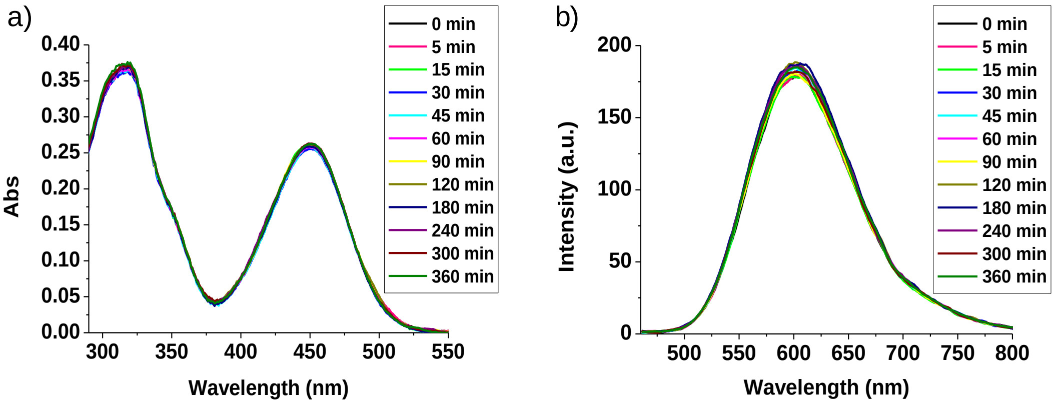

Supplementary Figure 188. Time-dependent changes in the absorption (a) and emission (b) spectra of the mixture of ligand **L3** and compound **11** in dichloromethane.

Supplementary Tables

Supplementary Table 1. Crystal data and structure refinement for ligand **L3**

Identification code	exp_506
Empirical formula	$C_{32}H_{21}N_5$
Formula weight	475.54
Temperature/K	293(2)
Crystal system	triclinic
Space group	*P*-1
a/Å	9.0019(3)
b/Å	12.5212(4)
c/Å	14.9408(4)
α/°	97.090(2)
β/°	106.359(3)
γ/°	107.535(3)
Volume/Å^3	1500.33(9)
Z	2
ρ_{calc}/g cm^{-3}	1.053
μ/mm^{-1}	0.501
F(000)	496.0
Crystal size/mm^3	0.42 × 0.4 × 0.36
Radiation	CuKα (λ = 1.54184)
2Θ range for data collection/°	7.604 to 148.804
Index ranges	$-10 \leq h \leq 11$, $-15 \leq k \leq 15$, $-18 \leq l \leq 18$
Reflections collected	31849
Independent reflections	5991 [R_{int} = 0.0809, R_{sigma} = 0.0493]
Data/restraints/parameters	5991/0/335
Goodness-of-fit on F^2	1.091
Final R indexes [I>=2σ (I)]	R_1 = 0.0560, wR_2 = 0.1668
Final R indexes [all data]	R_1 = 0.0731, wR_2 = 0.1788
Largest diff. peak/hole / e Å^{-3}	0.20/-0.27

Supplementary Table 2. Crystal data and structure refinement for ligand **L4**

dentification code	exp_392
Empirical formula	$C_{29}H_{23}N_3O_2$
Formula weight	445.50
Temperature/K	293(2)
Crystal system	monoclinic
Space group	$P2_1/c$
a/Å	10.6153(3)
b/Å	9.8791(3)
c/Å	22.7897(9)
α/°	90
β/°	96.660(3)
γ/°	90
Volume/Å^3	2373.82(14)
Z	4
ρ_{calc}/g cm^{-3}	1.247
μ/mm^{-1}	0.632
F(000)	936.0
Crystal size/mm^3	0.48 × 0.26 × 0.18
Radiation	CuKα (λ = 1.54184)
2Θ range for data collection/°	7.812 to 134.146
Index ranges	$-11 \leq h \leq 12$, $-11 \leq k \leq 11$, $-26 \leq l \leq 27$
Reflections collected	22834
Independent reflections	4201 [R_{int} = 0.0505, R_{sigma} = 0.0295]
Data/restraints/parameters	4201/1/315
Goodness-of-fit on F^2	1.039
Final R indexes [I>=2σ (I)]	R_1 = 0.0485, wR_2 = 0.1354
Final R indexes [all data]	R_1 = 0.0623, wR_2 = 0.1440
Largest diff. peak/hole / e Å^{-3}	0.51/-0.21

Supplementary Table 3. Crystal data and structure refinement for ligands **L6**

Identification code	exp_504
Empirical formula	$C_{29}H_{23}N_3$
Formula weight	413.50
Temperature/K	293(2)
Crystal system	monoclinic
Space group	$P2_1$/n
a/Å	14.4974(2)
b/Å	8.95760(10)
c/Å	17.2606(2)
α/°	90
β/°	96.9820(10)
γ/°	90
Volume/Å^3	2224.87(5)
Z	4
ρ_{calc}/g cm^{-3}	1.234
μ/mm^{-1}	0.565
F(000)	872.0
Crystal size/mm^3	0.46 × 0.32 × 0.26
Radiation	CuKα (λ = 1.54184)
2Θ range for data collection/°	7.528 to 149.346
Index ranges	$-18 \leq h \leq 18$, $-11 \leq k \leq 11$, $-21 \leq l \leq 21$
Reflections collected	54809
Independent reflections	4489 [R_{int} = 0.0597, R_{sigma} = 0.0230]
Data/restraints/parameters	4489/0/291
Goodness-of-fit on F^2	1.052
Final R indexes [I>=2σ (I)]	R_1 = 0.0401, wR_2 = 0.1088
Final R indexes [all data]	R_1 = 0.0439, wR_2 = 0.1117
Largest diff. peak/hole / e Å^{-3}	0.19/-0.11

Supplementary Table 4. Crystal data and structure refinement for ligands **L8**

Identification code	exp_379
Empirical formula	$C_{84}H_{66}N_{12}$
Formula weight	1243.48
Temperature/K	293(2)
Crystal system	triclinic
Space group	P-1
a/Å	12.3862(4)
b/Å	18.3189(5)
c/Å	18.4009(5)
α/°	113.900(3)
β/°	104.193(3)
γ/°	102.665(3)
Volume/Å^3	3453.3(2)
Z	2
ρ_{calc}/g cm^{-3}	1.196
μ/mm^{-1}	0.560
F(000)	1308.0
Crystal size/mm^3	0.48 × 0.32 × 0.26
Radiation	CuKα (λ = 1.54184)
2Θ range for data collection/°	7.802 to 150.484
Index ranges	$-15 \leq h \leq 15$, $-22 \leq k \leq 22$, $-22 \leq l \leq 22$
Reflections collected	86631
Independent reflections	13820 [R_{int} = 0.1004, R_{sigma} = 0.0633]
Data/restraints/parameters	13820/2/868
Goodness-of-fit on F^2	1.074
Final R indexes [I>=2σ (I)]	R_1 = 0.0583, wR_2 = 0.1758
Final R indexes [all data]	R_1 = 0.0811, wR_2 = 0.1963
Largest diff. peak/hole / e Å^{-3}	0.22/-0.29

Supplementary Table 5. Fluorescence quantum yields of ligands L1-L9 and metallacycles H1-H9 in DCM (5 µM) under N_2 atmosphere

Substituent	Ligand	Fluorescence quantum yield (%)	Metallacycle	Fluorescence quantum yield (%)
$-CF_3$	**L1**	64	**H1**	71
$-NO_2$	**L2**	1.0	**H2**	<1
$-CH{=}C(CN)_2$	**L3**	15	**H3**	23
−CHO	**L4**	48	**H4**	62
−H	**L5**	61	**H5**	60
$-CH_3$	**L6**	64	**H6**	56
$-OCH_3$	**L7**	61	**H7**	10
$-NH_2$	**L8**	2	**H8**	<1.0
$-N(CH_3)_2$	**L9**	<1.0	**H9**	<1.0

Supplementary Table 6. The fluorescence quantum yields of metallacycle H6 in CH_2Cl_2 at different concentrations

Concentration	0.5 µM	1.0 µM	3.0 µM	5.0 µM	10.0 µM
Fluorescence quantum yield (%)	53	50	53	52	51

Supplementary Table 7. The fluorescence lifetimes of ligands L1-L9 in DCM in air

Ligand		1	2	3	chisqr
**L1**	**Lifetime/ns**	0.2	1.7	2.4	1.07
	**Percent/%**	37	62	1	
	**Average/ns**	1.6			
**L2**		**1**	**2**		**chisqr**
	**Lifetime/ns**	1.6	3.0		1.02
	**Percent/%**	46	54		
	**Average/ns**	2.5			
**L3**		**1**	**2**	**3**	**chisqr**
	**Lifetime/ns**	0.7	1.9	4.3	1.03
	**Percent/%**	19	79	2	
	**Average/ns**	1.9			
**L4**		**1**	**2**		**chisqr**
	**Lifetime/ns**	0.2	6.3		1.09
	**Percent/%**	33	67		
	**Average/ns**	6.2			
**L5**		**1**	**2**		**chisqr**
	**Lifetime/ns**	0.4	2.3		1.06
	**Percent/%**	20	80		
	**Average/ns**	2.2			
**L6**		**1**	**2**	**3**	**chisqr**
	**Lifetime/ns**	0.3	2.45	2.85	1.05
	**Percent/%**	25	32	43	
	**Average/ns**	2.6			
**L7**		**1**	**2**	**3**	**chisqr**
	**Lifetime/ns**	0.1	1.1	4.1	1.03
	**Percent/%**	51	3	46	
	**Average/ns**	3.9			
**L8**		**1**	**2**		**chisqr**
	**Lifetime/ns**	0.3	1.9		0.89
	**Percent/%**	27	73		
	**Average/ns**	1.8			
**L9**		**1**	**2**	**3**	**chisqr**
	**Lifetime/ns**	0.2	2.1	4.3	1.04
	**Percent/%**	43	48	9	
	**Average/ns**	2.6			

Supplementary Table 8. The fluorescence lifetimes of metallacycles H1-H9 in DCM in air

Metallacycle		1	2			chisqr
H1	Lifetime/ns	1.6	2.4			1.07
	Percent/%	35	65			
	Average/ns	2.2				
H2		1	2	3		chisqr
	Lifetime/ns	0.2	1.1	3.16		1.18
	Percent/%	95	4	1		
	Average/ns	0.6				
H3		1	2	3		chisqr
	Lifetime/ns	0.1	1.4	2.4		1.05
	Percent/%	22	26	52		
	Average/ns	2.1				
H4		1	2			chisqr
	Lifetime/ns	1.7	2.4			1.03
	Percent/%	63	37			
	Average/ns	2.0				
H5		1	2	3		chisqr
	Lifetime/ns	0.5	2.0	2.8		1.03
	Percent/%	18	19	63		
	Average/ns	2.6				
H6		1	2	3		chisqr
	Lifetime/ns	0.6	2.4	3.2		1.04
	Percent/%	15	12	73		
	Average/ns	3.1				
H7		1	2	3		chisqr
	Lifetime/ns	0.2	0.7	2.3		1.12
	Percent/%	20	79	1		
	Average/ns	0.8				
H8		1	2	3	4	chisqr
	Lifetime/ns	0.1	0.7	2.2	8.6	1.07
	Percent/%	72	15	12	1	
	Average/ns	2.5				
H9		1	2	3	4	chisqr
	Lifetime/ns	0.08	0.4	1.3	5.5	1.07
	Percent/%	68	22	9	1	
	Average/ns	1.2				

Supplementary Table 9. The fluorescence lifetimes of ligands L1-L9 in DCM under N_2 atmosphere

Ligand		1	2	3	chisqr
L1	Lifetime/ns	0.3	1.0	1.8	1.07
	Percent/%	17	7	76	
	Average/ns	1.7			
L2		1	2		chisqr
	Lifetime/ns	1.5	4.8		1.07
	Percent/%	83	17		
	Average/ns	2.8			
L3		1	2		chisqr
	Lifetime/ns	1.8	6.2		1.08
	Percent/%	95	5		
	Average/ns	2.4			
L4		1	2		chisqr
	Lifetime/ns	2.1	6.4		1.02
	Percent/%	31	69		
	Average/ns	5.9			
L5		1	2		chisqr
	Lifetime/ns	0.2	2.4		1.06
	Percent/%	40	60		
	Average/ns	2.2			
L6		1	2		chisqr
	Lifetime/ns	0.3	2.9		1.02
	Percent/%	30	70		
	Average/ns	2.8			
L7		1	2	3	chisqr
	Lifetime/ns	0.1	1.4	4.5	1.04
	Percent/%	48	3	49	
	Average/ns	4.3			
L8		1	2		chisqr
	Lifetime/ns	2.0	5.9		1.11
	Percent/%	96	4		
	Average/ns	2.4			
L9		1	2	3	chisqr
	Lifetime/ns	0.1	1.9	3.3	1.06
	Percent/%	55	22	23	
	Average/ns	2.7			

Supplementary Table 10. The fluorescence lifetimes of metallacycles H1-H9 in DCM under N_2 atmosphere

**Metallacycle**		**1**	**2**		**chisqr**
**H1**	**Lifetime/ns**	1.4	2.6		1.07
	**Percent/%**	22	78		
	**Average/ns**	2.4			
**H2**		**1**	**2**	**3**	**chisqr**
	**Lifetime/ns**	0.1	0.8	1.9	0.96
	**Percent/%**	92	3	5	
	**Average/ns**	0.9			
**H3**		**1**	**2**	**3**	**chisqr**
	**Lifetime/ns**	0.4	2.0	2.8	1.07
	**Percent/%**	15	61	24	
	**Average/ns**	2.2			
**H4**		**1**	**2**		**chisqr**
	**Lifetime/ns**	1.7	2.8		1.06
	**Percent/%**	45	55		
	**Average/ns**	2.4			
**H5**		**1**	**2**	**3**	**chisqr**
	**Lifetime/ns**	0.4	2.3	3.6	1.08
	**Percent/%**	24	32	44	
	**Average/ns**	3.1			
**H6**		**1**	**2**	**3**	**chisqr**
	**Lifetime/ns**	0.5	2.5	3.8	1.05
	**Percent/%**	23	26	51	
	**Average/ns**	3.3			
**H7**		**1**	**2**	**3**	**chisqr**
	**Lifetime/ns**	0.2	0.7	3.3	1.12
	**Percent/%**	32	67	1	
	**Average/ns**	0.9			
**H8**		**1**	**2**	**3**	**chisqr**
	**Lifetime/ns**	0.2	1.4	3.3	1.04
	**Percent/%**	34	50	16	
	**Average/ns**	2.1			
**H9**		**1**	**2**	**3**	**chisqr**
	**Lifetime/ns**	0.3	1.3	3.3	1.07
	**Percent/%**	46	36	18	
	**Average/ns**	2.2			

Supplementary Table 11. Wavelength, molecular orbitals involved, oscillator strength for ligands L3, L7 and metallacycles H3, H7

Ligand/ metallacycle	Wavelen- gth (nm)	Orbital transitions		Oscillator strength (f)	description
**L3**	468.02	124→125	HOMO→LUMO	1.0160	e⁻ transfer to the side of cyanogroup
	358.88	124→126	HOMO→LUMO+1	0.5079	e⁻ transfer to the side of pyridyl
**H3**	465.45	266→267	HOMO→LUMO	1.1381	e⁻ density increases on cyanogroup
	414.85	266→268	HOMO→LUMO+1	0.7618	e⁻ density increases on pyridyl
**L7**	391.50	113→114	HOMO→LUMO	0.8423	e⁻ transfer to the side of pyridyl
	347.45	113→115	HOMO→LUMO+1	0.3324	e⁻ density decreases on methoxyl, e⁻ density increases on pyridyl
**H7**	449.48	255→256	HOMO→LUMO	1.2159	e⁻ density increases on pyridyl
	394.12	255→257	HOMO→LUMO+1	0.3078	

Supplementary Methods

Preparation of ligand L1

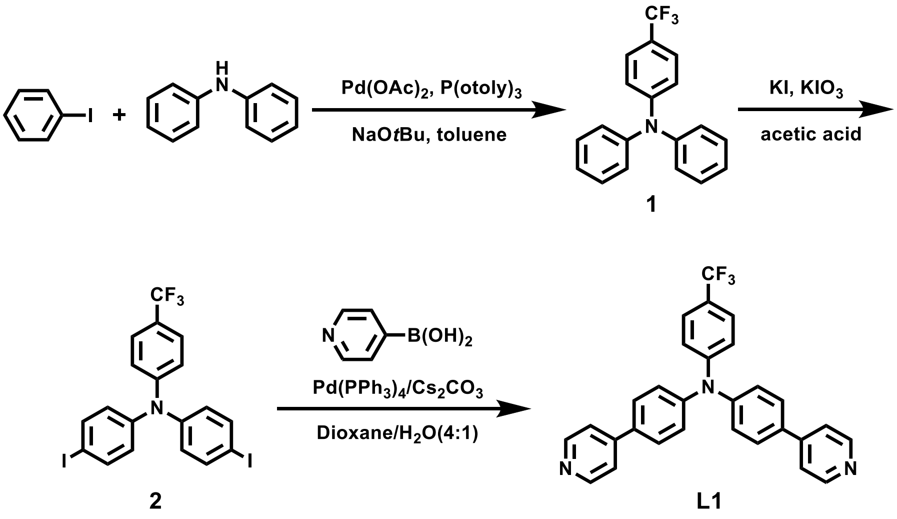

Supplementary Figure 189. Synthesis of ligand **L1**.

Compound 1: Compound **1** was synthesized according to the literature[1].

Compound 2: Compound **1** (802 mg, 1.82 mmol) and potassium iodide (454 mg, 2.73 mmol) were added in acetic acid/water (6.7 mL/0.67 mL). The mixture was stirred at 80 °C for 1 h. Then potassium iodate (585 mg, 2.73 mmol) was added and the reaction was stirred at 80 °C for 4 h. The solution was allowed to cool and the solid was collected, washed with water, and recrystallized from DCM/ethanol giving the product as a white powder (690 mg, 67%). M.p.: 95 °C. ^{1}H NMR (*d*$_6$-acetone, 300 MHz): δ 7.72 (d, 4H, *J* = 8.5 Hz), 7.59 (d, 2H, *J* = 8.4 Hz), 7.17 (d, 2H, *J* = 8.6 Hz), 6.97 (d, 4H, *J* = 8.5 Hz). ^{13}C NMR ($CDCl_3$, 126 MHz): δ 149.97, 146.40, 138.85, 127.02, 126.74, 126.71, 122.39, 87.81. HRMS (EI)**:** *m/z* Calcd for $C_{19}H_{12}F_3NI_2$: 564.9011. Found: 564.9009.

Ligand L1: Compound **2** (500 mg, 0.89 mmol), 4-pyridinylboronic acid (655 mg, 5.34 mmol), Cs_2CO_3 (300 mg, 5.34 mmol) and $Pd(PPh_3)_4$ (102 mg, 0.1 mmol) were added in dioxane/water (10 mL/2.5 mL). The mixture was stirred at 88 °C under Ar for 12 h. Then the mixture was cooled to room temperature and poured into water and the organic layer was separated and the aqueous phase was extracted with dichloromethane (CH_2Cl_2). The combined organic phases were washed with brine and

dried over sodium sulfate and the solvent was removed by rotary evaporation. The crude product was purified on Silica gel column using CH_2Cl_2 and EtOH (70:1, *v*/*v*) as eluent to give yellow solid **L1** (200 mg, 49%). R_f = 0.37(dichloromethane/ EtOH 40/1). M.p.: 162 °C. ^{1}H NMR (d_6-acetone, 400 MHz): δ 8.63(d, 4H, *J* = 4.4 Hz), 7.84 (d, 4H, *J* = 8.4Hz), 7.68 (d, 4H, *J* = 4.4 Hz), 7.65 (d, 2H, *J* = 8.8 Hz), 7.32 (d, 4H, *J* = 8.8 Hz), 7.27 (d, 2H, *J* = 8.8 Hz). ^{13}C NMR (d_6-acetone, 126 MHz): δ151.17, 148.23, 147.42, 134.36, 129.02, 127.39, 127.35, 126.29, 123.36, 121.55. HRMS (EI)**:** *m/z* Calcd for $C_{29}H_{20}F_3N_3$: 467.1609. Found: 467.1608.

Preparation of ligand L2

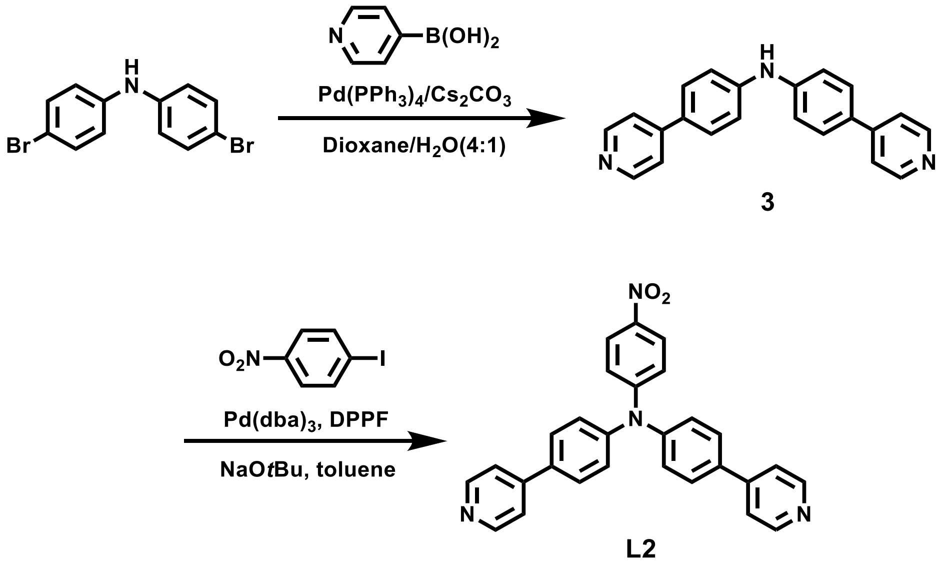

Supplementary Figure 190. Synthesis of ligand **L2**.

Compound 3: Compound **3** was synthesized according to the literature[2].

Ligand L2: Compound **3** (110 mg, 0.25 mmol) were added to a 50 mL Schlenk flask then degassed, and back-filled three times with N_2. Anhydrous and degrassed alcohol (10 mL) were introduced into the reaction flask by syringe. Then Pd/C (3 mg, 0.03 mmol) and hydrazine hydrate (0.7 mL, 14.2 mmol) under N_2. The mixture was stirred at 75 °C under Ar for 10 h. The crude product was purified on Silica gel column using CH_2Cl_2 and EtOH (70:1, *v*/*v*) as eluent to give yellow solid **L2** (500 mg, 54%). R_f = 0.26 (dichloromethane/EtOH 40/1). M.p.: 108 °C. ^{1}H NMR (d_6-acetone, 400 MHz): δ 8.57(d, 4H, *J* = 6 Hz), 7.72 (d, 4H, *J* = 8.8 Hz), 7.65(d, 4H, *J* = 6 Hz), 7.08 (d, 4H, *J* = 8.4 Hz), 6.89 (d, 2H, *J* = 8.4 Hz), 6.63 (d, 2H, *J* = 8.8 Hz). ^{13}C NMR (d_6-acetone, 126

MHz): δ150.78, 149.40, 147.60, 136.05, 131.00, 129.11, 128.32, 128.10, 127.65, 123.63, 122.71, 121.50, 121.12, 121.01, 116.00. HRMS (EI): *m/z* Calcd for $C_{28}H_{22}N_4$: 414.1844. Found: 414.1849.

Preparation of ligand L3

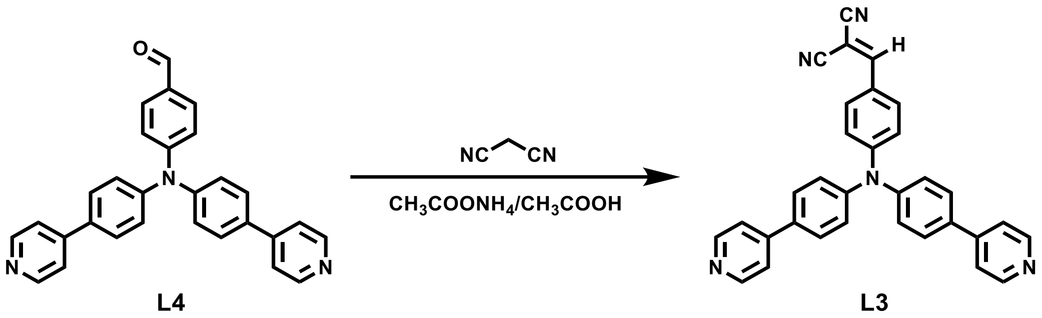

Supplementary Figure 191. Synthesis of **L3**.

Ligand L3: **L4** (306 mg, 0.72 mmol), malonitrile (533 mg, 8.1 mmol) and ammonium acetate (900 mg, 11.7 mmol) were added in 108 mL acetic acid. The mixture was stirred at 70 °C under Ar for 3 h. Then the mixture was cooled to room temperature and poured into brine and was extracted by CH_2Cl_2, and dried under Na_2SO_4. The crude product was purified on Silica gel column using CH_2Cl_2 and EtOH (40:1, *v*/*v*) as eluent to give red solid **L3** (126 mg, 37%). R_f = 0.4 (dichloromethane/ EtOH 20/1). M.p.: 199 °C. ^{1}H NMR (d_6-acetone, 300 MHz): δ 8.65 (d, 4H, *J* = 6.1 Hz), 8.11 (s, 1H), 7.98 (d, 2H, *J* = 9.0 Hz), 7.91 (d, 4H, *J* = 8.6 Hz), 7.70 (d, 4H, *J* = 6.1 Hz), 7.44 (d, 4H, *J* = 8.6 Hz), 7.19 (d, 2H, *J* = 8.8 Hz). ^{13}C NMR (d_6-acetone, 126 MHz): δ 159.64, 153.52, 151.35, 147.45, 147.21, 136.02, 133.85, 129.42, 127.80, 125.32, 121.87, 120.90, 115.75, 114.88. HRMS (EI): *m/z* Calcd for 475.1797. Found: 475.1795.

Preparation of ligand L4

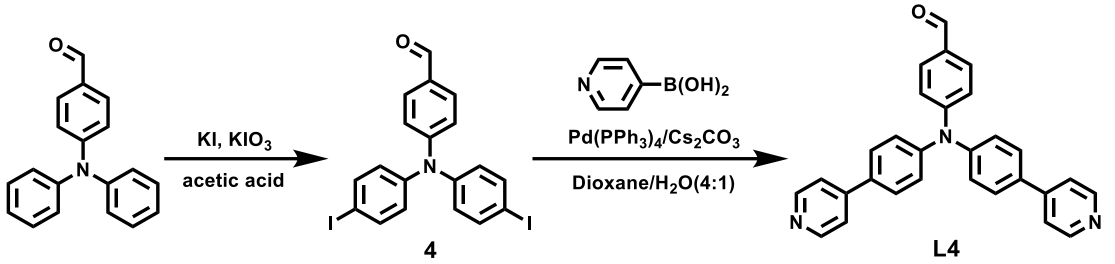

Supplementary Figure 192. Synthesis of ligand **L4**.

Compound 4: Compound **4** was synthesized according to the literature[3].

Ligand L4: Compound **4** (1.20 g, 2.29 mmol), 4-pyridinylboronic acid (1.13 g, 9.19 mmol), Cs_2CO_3 (1.94 g, 5.95 mmol) and $Pd(PPh_3)_4$ (434 mg, 0.38 mmol) were added in dioxane/water (32 mL/8 mL). The mixture was stirred at 88 °C under Ar for 12 h. Then the mixture was cooled to room temperature and poured into water and the organic layer was separated and the aqueous phase was extracted with dichloromethane (CH_2Cl_2). The combined organic phases were washed with brine and dried over sodium sulfate and the solvent was removed by rotary evaporation. The crude product was purified on Silica gel column using CH_2Cl_2 and EtOH (40:1, *v*/*v*) as eluent to give yellow solid **L4** (659 mg, 68%). R_f = 0.35(dichloromethane/ EtOH 20/1). M.p.: 110 °C. ^{1}H NMR (d_6-acetone, 500 MHz): δ 9.91 (s, 1H), 8.64 (dd, 4H, J = 4.6, 1.5 Hz), 7.86 (dd, 6H, J = 10.9, 8.7 Hz), 7.69 (dd, 4H, J = 4.6, 1.5 Hz), 7.37 (d, 4H, J = 8.6 Hz,), 7.21 (d, 2H, J = 8.6 Hz,).^{13}C NMR (d_6-acetone, 126 MHz): δ 191.02, 153.35, 151.37, 148.12, 147.63, 135.11, 132.05, 131.84, 129.33, 127.16, 122.24, 121.85. HRMS (EI)**:** *m/z* Calcd for 427.1685. Found: 427.1682.

Preparation of ligand L5

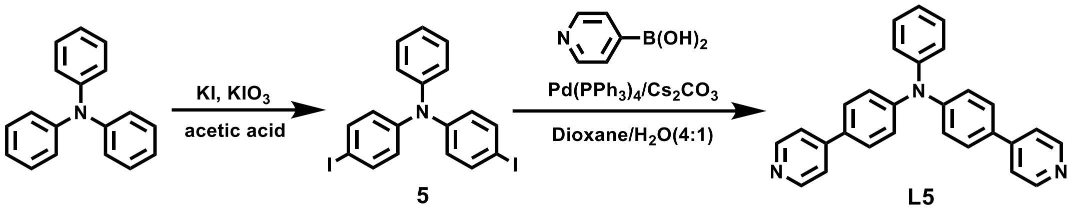

Supplementary Figure 193. Synthesis of ligand **L5**.

Compound 5: Compound **5** was synthesized according to the literature[4].

Ligand L5: Compound **5** (1.00 g, 2 mmol), 4-pyridinylboronic acid (1.40 g, 12 mmol), Cs_2CO_3 (3.70 g, 11.3 mmol) and $Pd(PPh_3)_4$ (230 mg, 0.2 mmol) were added in dioxane/water (20 mL/5 mL). The mixture was stirred at 88 °C under Ar for 12 h. Then the mixture was cooled to room temperature and poured into water and the organic layer was separated and the aqueous phase was extracted with dichloromethane (CH_2Cl_2). The combined organic phases were washed with brine and dried over sodium sulfate and the solvent was removed by rotary evaporation. The crude product was purified on Silica gel column using CH_2Cl_2 and EtOH (70:1, *v*/*v*) as eluent to give yellow solid **L5** (300 mg, 40%). R_f = 0.32 (dichloromethane/EtOH 40/1). M.p.: 133 °C. ^{1}H NMR (d_6-acetone, 400 MHz): δ 8.61 (dd, 4H, *J* = 4.5, 1.7 Hz), 7.79–7.75 (m, 4H), 7.65 (dd, 4H, *J* = 4.5, 1.7 Hz,), 7.43–7.38 (m, 2H), 7.21 (dq, 7H, *J* = 3.7, 2.7 Hz). ^{13}C NMR (d_6-acetone, 126 MHz): δ 151.73, 151.38, 149.42, 147.94, 147.87, 132.94, 130.78, 128.90, 126.62, 125.44, 124.85, 122.26, 121.64. HRMS (EI): *m/z* Calcd for $C_{28}H_{21}N_3$: 399.1735. Found: 399.1739.

Preparation of ligand L6

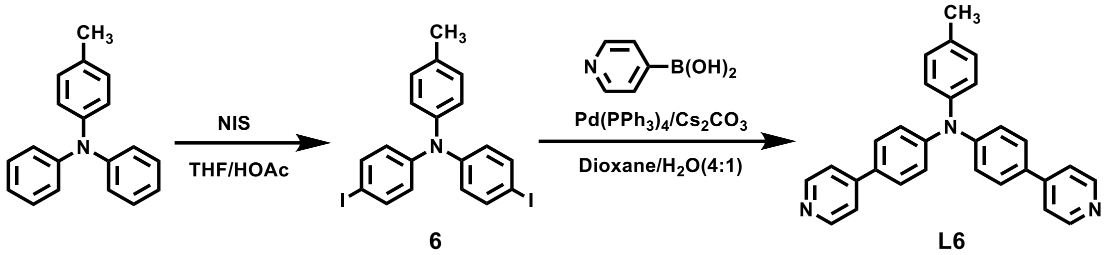

Supplementary Figure 194. Synthesis of ligand **L6**.

Compound 6: Compound **6** was synthesized according to the literature[5].

Ligand L6: Compound **6** (250 mg, 0.48 mmol), 4-pyridinylboronic acid (360 mg, 2.93 mmol), Cs_2CO_3 (956 mg, 2.93 mmol) and $Pd(PPh_3)_4$ (55 mg, 0.05 mmol) were added in dioxane/water(5 mL/1.3 mL). The mixture was stirred at 88 °C under Ar for 12 h. the mixture was cooled to room temperature and poured into water and the organic layer was separated and the aqueous phase was extracted with dichloromethane (CH_2Cl_2). The combined organic phases were washed with brine and dried over sodium sulfate and the solvent was removed by rotary evaporation. The crude product was purified on Silica gel column using CH_2Cl_2 and EtOH (70:1, *v*/*v*) as eluent to give yellow solid **L6** (256 mg, 60%). R_f = 0.35 (dichloromethane/EtOH 40/1). M.p.: 178 °C. ^{1}H NMR (d_6-acetone, 400 MHz): δ 8.60 (d, 4H, J = 4.4 Hz), 7.76 (d, 4H, J = 6.4 Hz), 7.65 (d, 4H, J = 4.4 Hz), 7.24 (d, 2H, J = 8.0 Hz), 7.19 (d, 4H, J = 6.8 Hz), 7.11 (d, 2H, J = 8.4 Hz), 2.35 (s, 3H). ^{13}C NMR (d_6-acetone, 126 MHz): δ 151.38, 149.58, 147.96, 145.34, 135.49, 132.61, 131.44, 128.84, 127.16, 124.42, 121.63, 21.08. HRMS (EI)**:** *m/z* Calcd for $C_{29}H_{23}N_3$: 413.1892. Found: 413.1890.

Preparation of ligand L7

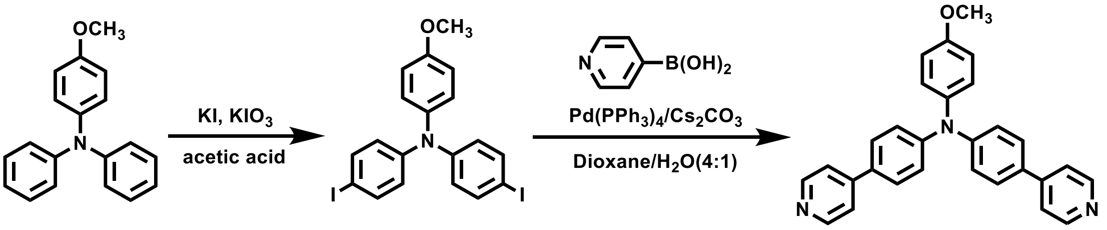

7 **L7**

Supplementary Figure 195. Synthesis of ligand **L7**.

Compound 7: Compound **7** was synthesized according to the literature[6].

Ligand L7: Compound **7** (257 mg, 0.48 mmol), 4-pyridinylboronic acid (360 mg, 2.93 mmol), Cs_2CO_3 (956 mg, 2.93 mmol) and $Pd(PPh_3)_4$ (55 mg, 0.05 mmol) were added in dioxane/water(5 mL/1.3 mL). The mixture was stirred at 88 ^oC under Ar for 12 h. Then the mixture was cooled to room temperature and poured into water and the organic layer was separated and the aqueous phase was extracted with dichloromethane (CH_2Cl_2). The combined organic phases were washed with brine and dried over sodium sulfate and the solvent was removed by rotary evaporation. The crude product was purified on Silica gel column using CH_2Cl_2 and EtOH (70:1, *v*/*v*) as eluent to give yellow solid **L7** (100 mg, 43%). R_f = 0.34 (dichloromethane/EtOH 40/1). M.p.: 70 ^oC. ^1H NMR (d_6-acetone, 300 MHz): δ 8.60 (d, 4H, J = 6 Hz), 7.74 (d, 4H, J = 8.7 Hz), 7.64 (d, 4H, J = 6 Hz), 7.24 (d, 4H, J = 6 Hz), 7.16-7.20 (m, 6H), 7.01 (d, 2H, J = 9 Hz), 3.84 (s, 3H). ^{13}C NMR (d_6-acetone, 126 MHz): δ 158.55, 151.37, 149.71, 147.99, 142.82, 140.48, 132.23, 129.36, 128.78, 123.79, 121.58, 116.24, 55.98. HRMS (EI)**:** *m/z* Calcd for $C_{29}H_{23}N_3O$: 428.1841. Found: 429.1844.

Preparation of ligand L8

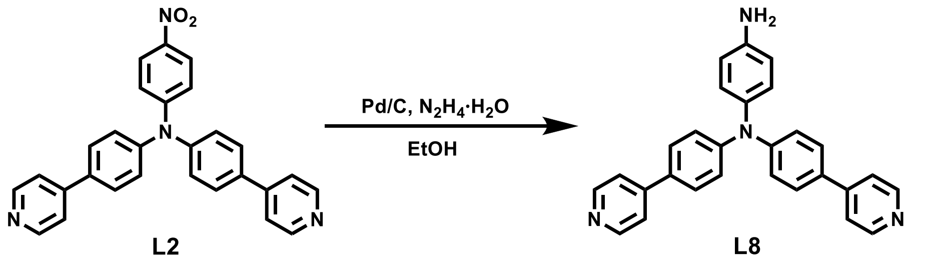

Supplementary Figure 196. Synthesis of ligand **L8**.

Ligand L8: Compound **L2** (110 mg, 0.25 mmol) were added to a 50 mL Schlenk flask then degassed, and back-filled three times with N_2. Anhydrous and degassed alcohol (10 mL) were introduced into the reaction flask by syringe. Then Pd/C (3 mg, 0.03 mmol) and hydrazine hydrate (0.7 mL, 14.2 mmol) under N_2. The mixture was stirred at 75 ^{o}C under Ar for 10 h. The crude product was purified on Silica gel column using CH_2Cl_2 and EtOH (70:1, *v*/*v*) as eluent to give yellow solid **L8** (500 mg, 54%). R_f = 0.26 (dichloromethane/EtOH 40/1). M.p.: 118 ^{o}C. ^{1}H NMR (d_6-acetone, 500 MHz): δ 8.58 (d, 4H, *J* = 5.9 Hz), 7.71 (d, 4H, *J* = 8.6 Hz), 7.62 (d, 4H, *J* = 6.0 Hz), 7.17 (d, 4H, *J* = 8.6 Hz), 6.97 (d, 4H, *J* = 8.5 Hz), 6.76 (d, 4H, *J* = 8.5 Hz), 4.80 (s, 4H). ^{13}C NMR (d_6-acetone, 126 MHz): δ 150.78, 149.40, 147.60, 136.05, 131.00, 129.11, 128.32, 128.10, 127.65, 123.63, 122.71, 121.50, 121.12, 121.01, 116.00. HRMS (EI): *m*/*z* Calcd for $C_{28}H_{22}N_4$: 414.1844. Found: 414.1849.

Preparation of ligand L9

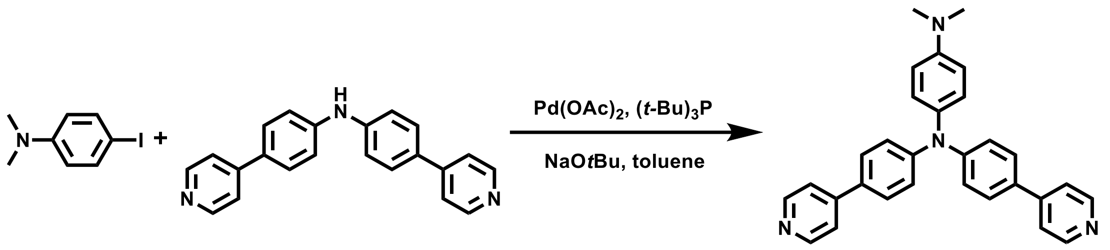

Supplementary Figure 197. Synthesis of ligand **L9**.

Ligand L9: Compound **3** (60 mg, 3.6 mmol)), 4-iodo-*N*, *N*-dimethylaniline (50 mg, 0.2 mmol), Tri-tert-butylphosphine (3 mg, 0.015 mmol), palladium diacetate (1mg, 0.001 mmol) and NaO*t*-Bu(29 mg, 0.3 mmol) were added were added a 50 mL Schlenk flask then degassed, and back-filled three times with N_2. Anhydrous and degassed toluene (1 mL) was introduced into the reaction flask by syringe. The mixture was stirred at 115 °C under Ar for 3.5 h. The crude product was purified on Silica gel column using CH_2Cl_2 and EtOH (70:1, *v*/*v*) as eluent to give yellow solid **L9** (40 mg, 45%). R_f = 0.38 (dichloromethane/EtOH 40/1). M.p.: 95 °C. ^{1}H NMR (d_6-acetone, 400 MHz): δ 8.58 (d, 4H, *J* = 4.8 Hz), 7.7 (d, 4H, *J* = 8.4 Hz), 7.61 (d, 4H, *J* = 4.4 Hz), 7.15 (d, 4H, *J* = 8.8 Hz), 7.07 (d, 2H, *J* = 9.2 Hz), 6.8 (d, 2H, *J* = 8.8 Hz), 2.97(s, 6H). ^{13}C NMR (d_6-acetone, 126 MHz): δ 150.93, 149.51, 149.45, 147.67, 135.93, 131.27, 128.92, 128.26, 122.97, 121.13, 144.22, 40.50. HRMS (EI): *m*/*z* Calcd for $C_{30}H_{26}N_4$: 442.2157. Found: 442.2159.

Preparation of metallacycle H1

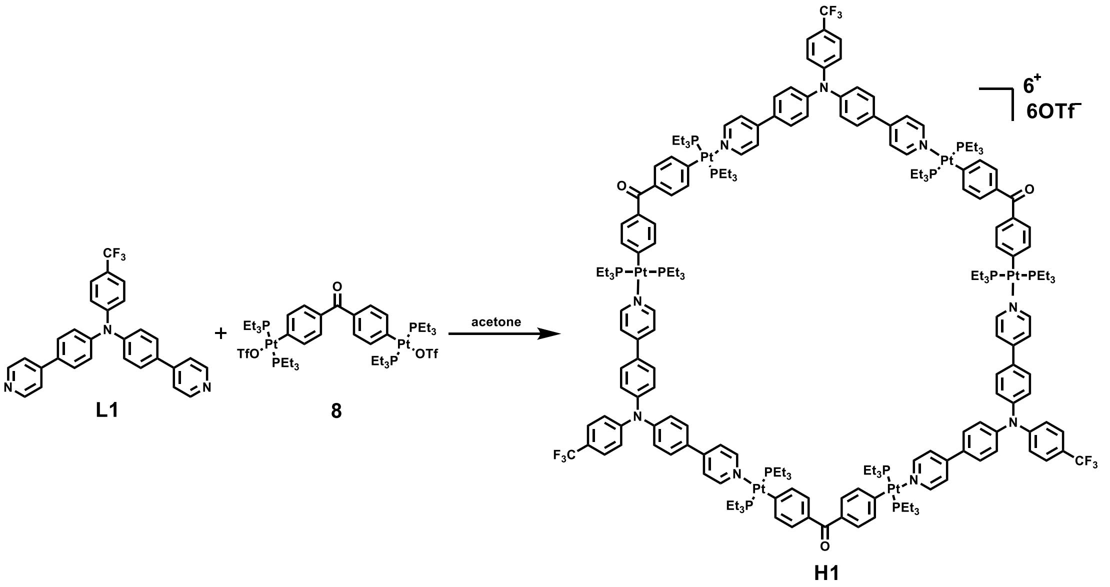

Supplementary Figure 198. Synthesis of metallacycle **H1**.

Metallacycle H1: The dipyridyl donor ligand **L1** (3.09 mg, 6.6 μmol) and the organoplatinum 120° acceptor **8** (8.87 mg, 6.6 μmol) were weighed accurately into a glass vial. To the vial was added 2.0 mL acetone and the reaction solution was then stirred at 40 °C for 4 h to yield a homogeneous light solution. Yellow solid product **H1** was obtained by removing the solvent under vacuum. Yield: 11.96 mg, >99%. M.p.: 261 °C. ^{1}H NMR (d_6-acetone, 400 MHz): δ 9.02 (d, 12H, J = 5.6 Hz), 8.20 (d, 12H, J = 6.0 Hz), 8.05 (d, 12H, J = 8.4 Hz), 7.72 (d, 18H, J = 8Hz), 7.58 (d, 12H, J = 7.6 Hz), 7.36-7.40 (m, 18H), 1.52-1.54 (m,72H), 1.17-1.25 (m, 108H). ^{13}C NMR (d_6-acetone, 126 MHz): δ 196.11, 153.40, 150.75, 150.68, 137.13, 135.50, 134.11, 130.04, 129.62, 129.36, 129.15, 128.82, 124.69, 124.53, 123.97, 123.76, 123.18, 121.20, 116.38, 13.34, 13.20, 13.07, 7.89. ^{31}P NMR (d_6-acetone, 161.9 MHz): δ 14.27 (s, $J_{Pt\text{-}P}$ = 2661.47 Hz); ESI-TOF-MS of **H1**: calcd for [M – 4OTf]$^{4+}$: 1156.3705, found: 1156.4639; calcd for [M – 5OTf]$^{5+}$: 895.3060, found: 895.3412.

Preparation of metallacycle H2

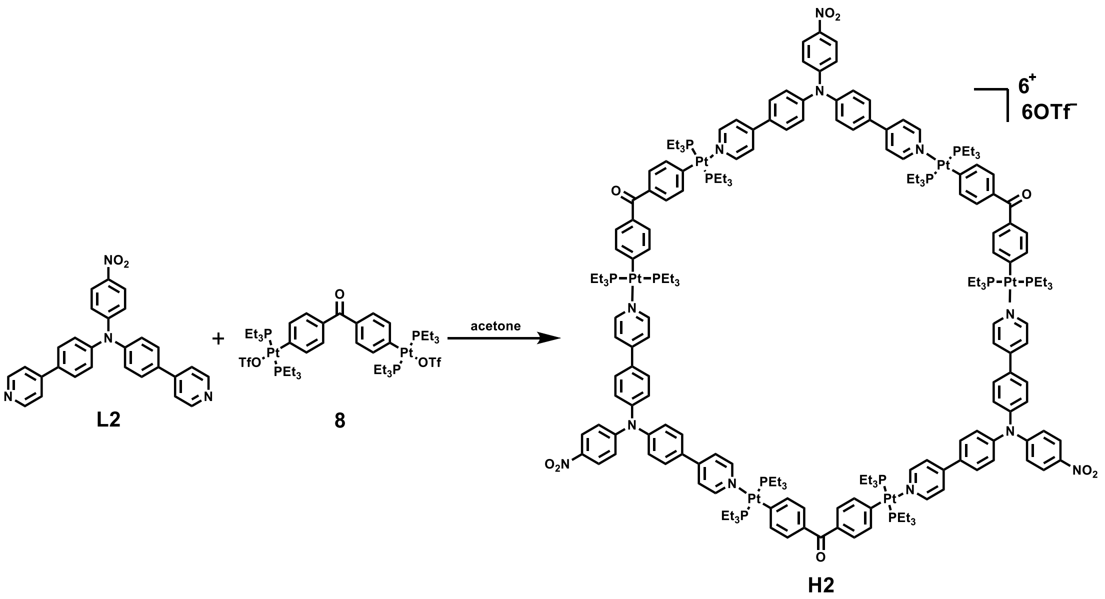

Supplementary Figure 199. Synthesis of metallacycle **H2**.

Metallacycle H2: The dipyridyl donor ligand **L2** (2.6 mg, 5.8 μmol) and the organoplatinum 120° acceptor **8** (7.7 mg, 5.8 μmol) were weighed accurately into a glass vial. To the vial was added 2.0 mL acetone and the reaction solution was then stirred at 40 °C for 4 h to yield a homogeneous light solution. Yellow solid product **H2** was obtained by removing the solvent under vacuum. Yield: 10.3 mg, >99%. M.p.: 278 °C. ^{1}H NMR (d_6-acetone, 400 MHz): δ 9.04 (d, 12H, J = 5.6 HZ), 8.20-8.23 (m, 18H), 8.11 (d, 12H, J = 8.4 Hz), 7.72 (d, 12H, J = 8 Hz), 7.57 (d, 12H, J = 8 Hz), 7.48 (d, 12H, J = 8.4 Hz), 7.28 (d, 6H, J = 9.4 Hz), 1.52-1.53 (m, 72H), 1.17-1.28 (m, 108H). ^{13}C NMR (d_6-acetone, 126 MHz): δ 196.14, 153.67, 153.32, 150.34, 148.90, 143.44, 143.02, 137.11, 134.12, 132.90, 130.04, 129.98, 127.34, 126.27, 125.29, 122.41, 13.32, 13.19, 13.05, 7.89. ^{31}P NMR (d_6-acetone, 161.9 MHz): δ 14.54 (s, $J_{Pt\text{-}P}$ = 2656.76 Hz); ESI-TOF-MS of **H2**: calcd for [M – 4OTf]$^{4+}$: 1189.8573. found: 1189. 8010.

Preparation of metallacycle H3

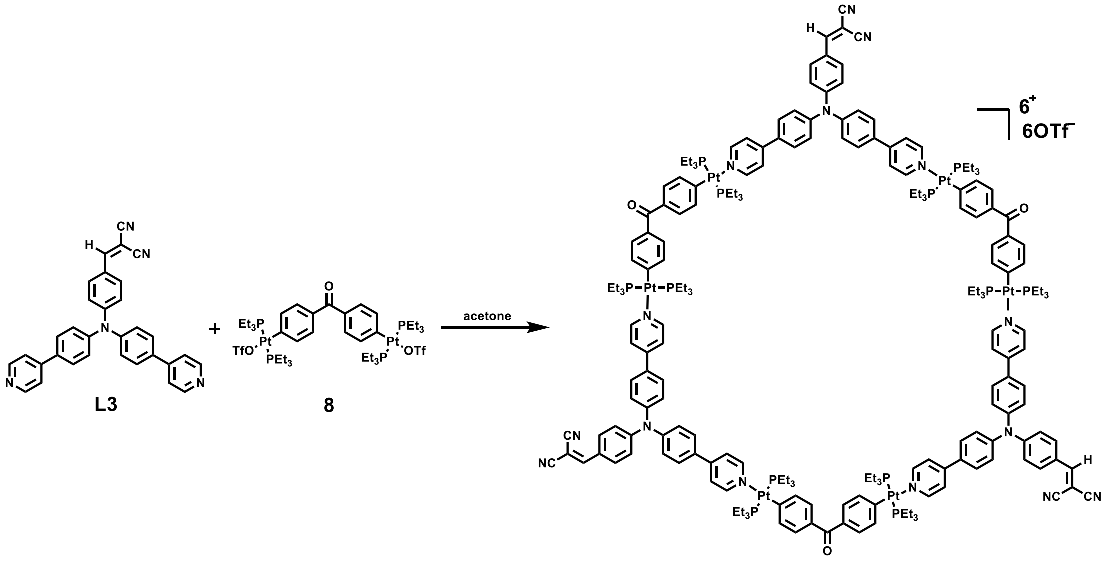

H3

Supplementary Figure 200. Synthesis of metallacycle **H3**.

Metallacycle H3: The dipyridyl donor ligand **L3** (2.07 mg, 4.4 μmol) and the organoplatinum 120° acceptor **8** (5.84 mg, 4.4 μmol) were weighed accurately into a glass vial. To the vial was added 2.0 mL acetone and the reaction solution was then stirred at 40 °C for 4 h to yield a homogeneous light solution. Red solid product **H3** was obtained by removing the solvent under vacuum. Yield: 7.91 mg, >99%. M.p.: 269 °C. ^{1}H NMR (d_6-acetone, 400 MHz): δ 9.04 (d, 12H, J = 6.0 Hz), 8.22 (d, 12H, J = 6.4 Hz), 8.18 (s, 3H), 8.11 (d, 12H, J = 8.6 Hz), 8.03 (d, 6H, J = 9.0 Hz), 7.72 (d, 12H, J = 7.9 Hz), 7.58 (d, 12H, J = 7.9 Hz,), 7.49 (d, 12H, J = 8.6 Hz,), 7.29 (d, 6H, J = 8.9 Hz,), 1.58-1.47 (m, 72H), 1.25-1.17 (m, 108H). ^{13}C NMR (d_6-acetone, 126 MHz): δ 196.06, 159.74, 153.67, 152.87, 150.38, 148.72, 143.40, 137.11, 134.14, 133.78, 132.99, 130.06, 129.97, 127.46, 126.51, 125.31, 123.73, 122.60, 121.17, 115.51, 114.69, 13.33, 13.20, 13.06, 7.90. ^{31}P NMR (d_6-acetone, 161.9 MHz): δ 14.65 (s, J_{Pt-P} = 2580.7 Hz). ESI-TOF-MS of **H3**: calcd for [M – 3OTf]$^{3+}$: 1666.48, found: 1666.39.

Preparation of metallacycle H4

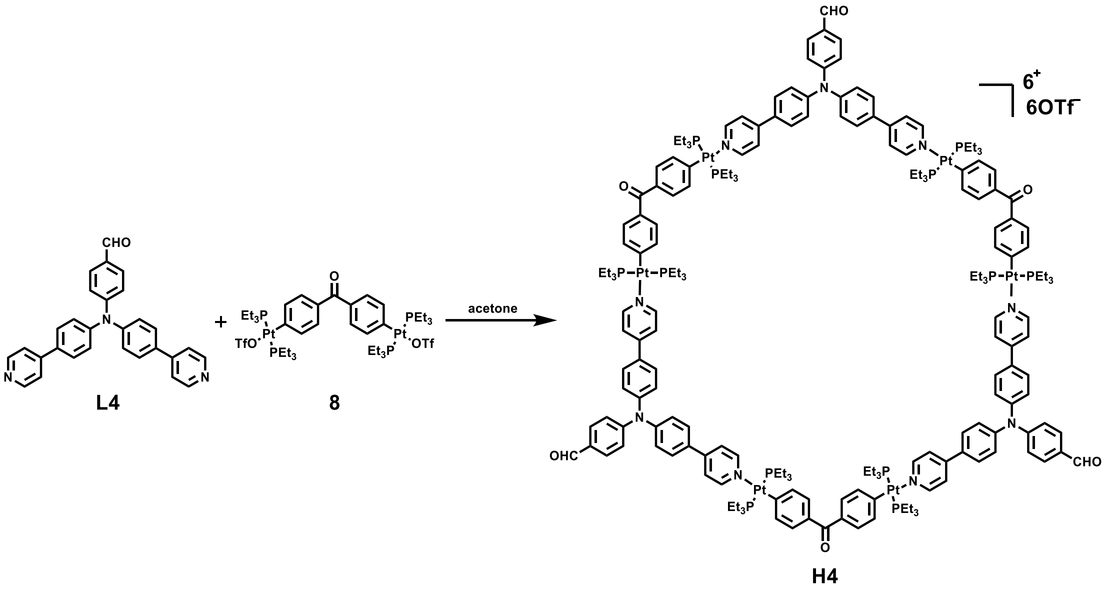

Supplementary Figure 201. Synthesis of metallacycle **H4**.

Metallacycle H4: The dipyridyl donor ligand **L4** (3.25 mg, 7.6 μmol) and the organoplatinum 120° acceptor **8** (11.21 mg, 8.3 μmol) were weighed accurately into a glass vial. To the vial was added 2.0 mL acetone and the reaction solution was then stirred at 40 °C for 4 h to yield a homogeneous light solution. Yellow solid product **H4** was obtained by removing the solvent under vacuum. Yield: 15.9 mg, >99%. M.p.: 298 °C. ^{1}H NMR (d_6-acetone, 300 MHz): δ 9.97 (s, 3H), 9.03 (d, 12H, J = 6 Hz), 8.21 (d, 12H, J = 6.3 Hz), 8.08 (d, 12H, J = 8.4 Hz), 7.91 (d, 6H, J = 8.7 Hz), 7.72 (d, 12H, J = 7.8 Hz), 7.58 (d, 12H, J = 8.1 Hz), 7.42 (d, 12H, J = 8.4 Hz), 7.31 (d, 6H, J = 8.7 Hz), 1.52-1.54 (m, 72H), 1.19-1.26 (m, 108H). ^{13}C NMR (d_6-acetone, 126 MHz): δ 196.11, 191.18, 153.62, 152.69, 150.43, 149.42, 143.45, 137.12, 134.13, 132.83, 132.11, 132.04, 130.05, 129.82, 126.73, 125.16, 123.81, 121.19, 13.33, 13.20, 13.06, 7.90. ^{31}P NMR (d_6-acetone, 161.9 MHz): δ 14.31 (s, J_{Pt-P} = 2653.22 Hz). ESI-TOF-MS of **H4**: calcd for [M – 4OTf]$^{4+}$: 1177.1146, found: 1177.0656; calcd for [M – 5OTf]$^{5+}$: 911.9014, found: 911.9049.

Preparation of metallacycle H5

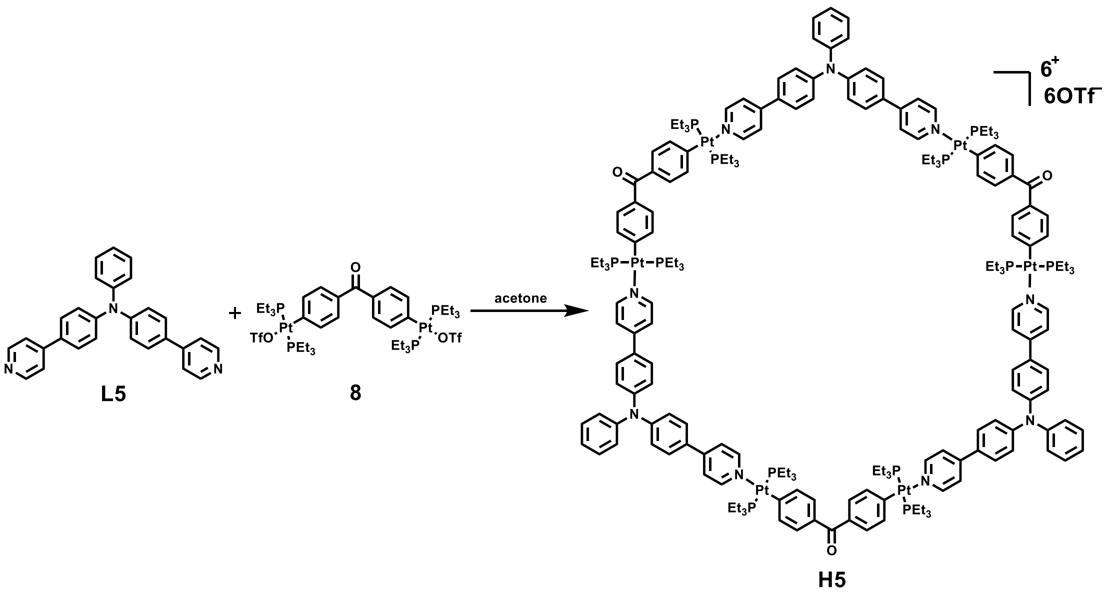

Supplementary Figure 202. Synthesis of metallacycle **H5**.

Metallacycle H5: The dipyridyl donor ligand **L5** (3.92 mg, 9.8 μmol) and the organoplatinum 120° acceptor **8** (13.2 mg, 9.8 μmol) were weighed accurately into a glass vial. To the vial was added 2.0 mL acetone and the reaction solution was then stirred at 40 °C for 4 h to yield a homogeneous light solution. Yellow solid product **H5** was obtained by removing the solvent under vacuum. Yield: 11.18 mg, >99%. M.p.: 286 °C. ^{1}H NMR (d_6-acetone, 400 MHz): δ 8.99 (d, 12H, J = 6 Hz), 8.16 (d, 12H, J = 6.4 Hz), 7.99 (d, 12H, J = 8.8 Hz), 7.72 (d, 12H, J = 8 Hz), 7.58 (d, 12H, J = 8 Hz), 7.46-7.5 (m, 6H), 7.29 (d, 18H, J = 4.4Hz), 7.26(s, 3H), 1.51-1.53 (m, 72H), 1.17-1.24 (m, 108H). ^{13}C NMR (d_6-acetone, 126 MHz): δ 196.10, 153.50, 150.58, 150.38, 147.16, 137.13, 134.12, 130.95, 130.10, 130.05, 129.43, 127.31, 126.38, 124.79, 124.57, 13.34, 13.20, 13.07, 7.90. ^{31}P NMR (d_6-acetone, 161.9 MHz): δ 14.54 (s, $J_{Pt\text{-}P}$ = 2661.96 Hz); ESI-TOF-MS of **H5**: calcd for [M – 4OTf]$^{4+}$: 1207.3610, found: 1207.4419; calcd for [M – 5OTf]$^{5+}$: 936.0985, found: 935.9138.

Preparation of metallacycle H6

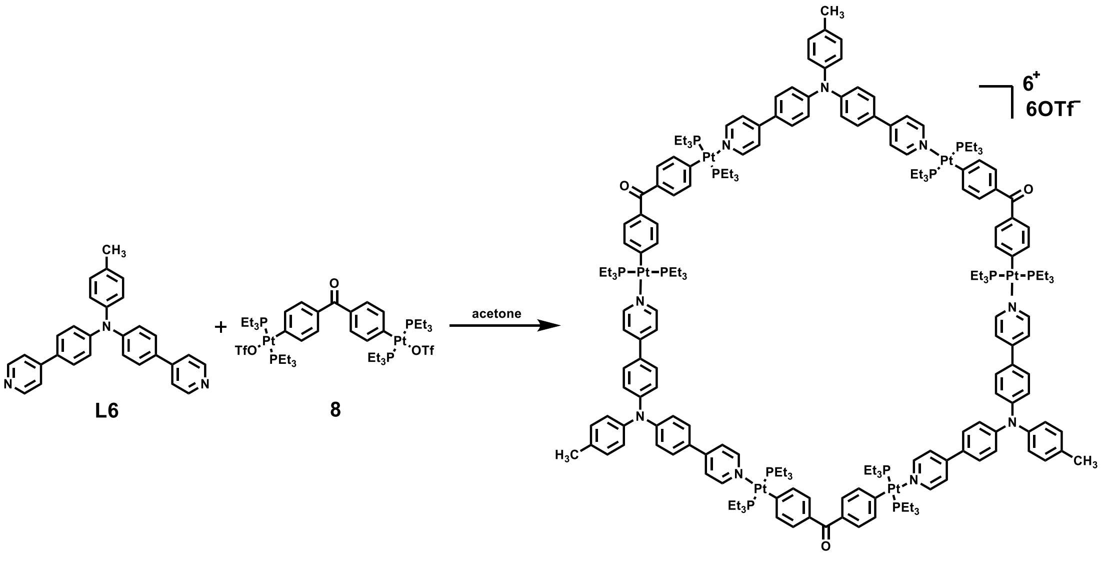

Supplementary Figure 203. Synthesis of metallacycle **H6**.

Metallacycle H6: The dipyridyl donor ligand **L6** (2.63 mg, 6.3 μmol) and the organoplatinum 120° acceptor **8** (8.55 mg, 6.3 μmol) were weighed accurately into a glass vial. To the vial was added 2.0 mL acetone and the reaction solution was then stirred at 40 °C for 4 h to yield a homogeneous light solution. Yellow solid product **H6** was obtained by removing the solvent under vacuum. Yield: 11.18 mg, >99%. M.p.: 268 °C. ^{1}H NMR (d_6-acetone, 300 MHz): δ 8.98 (d, 12H, J = 6 Hz), 8.15 (d, 12H, J = 6.3 Hz), 7.98 (d, 12H, J = 8.7 Hz), 7.72 (d, 12H, J = 7.8 Hz), 7.58 (d, 12H, J = 7.8Hz), 7.26-7.32 (m, 18H), 7.16 (d, 6H, J = 8.1 Hz), 2.40 (s, 9H), 1.51-1.53 (m, 72H), 1.15-1.26 (m, 108H). ^{13}C NMR (d_6-acetone, 126 MHz): δ 196.12, 153.47, 150.59, 150.45, 144.49, 143.53, 137.13, 136.41, 134.11, 131.55, 130.04, 129.77, 129.36, 127.58, 124.73, 124.19, 20.98, 13.33, 13.20, 13.06, 7.89. ^{31}P NMR (d_6-acetone, 161.9 MHz): δ 13.40 (s, $J_{Pt\text{-}P}$ = 2726.08 Hz); ESI-TOF-MS of **H6**: calcd for [M – 4OTf]$^{4+}$: 1166.6302, found: 1166.5944; calcd for [M – 5OTf]$^{5+}$: 903.5138, found: 908.5313.

Preparation of metallacycle H7

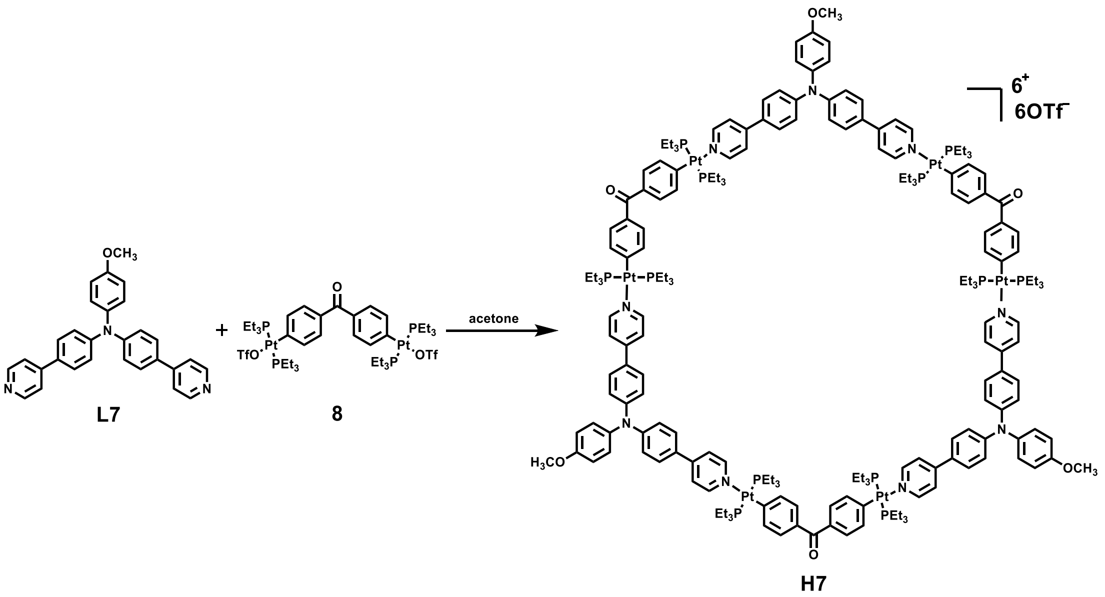

Supplementary Figure 204. Synthesis of metallacycle **H7**.

Metallacycle H7: The dipyridyl donor ligand **L7** (2.6 mg, 6.0 μmol) and the organoplatinum 120° acceptor **8** (8.1 mg, 6.0 μmol) were weighed accurately into a glass vial. To the vial was added 2.0 mL acetone and the reaction solution was then stirred at 40 °C for 4 h to yield a homogeneous light solution. Yellow solid product **H7** was obtained by removing the solvent under vacuum. Yield: 10.7mg, >99%. M.p.: 257 °C. ^{1}H NMR (d_6-acetone, 400 MHz): δ 8.97 (d, 12H, J = 6 Hz), 8.15 (d, 12H, J = 6.8 Hz), 7.97 (d, 12H, J = 8.4 Hz), 7.04 (d, 12H, J = 8 Hz), 7.60 (d, 12H, J = 8 Hz), 7.22-7.28 (m, 18H), 7.07 (d, 6H, J = 9.2Hz), 3.87 (s, 9H), 1.50-1.53 (m, 72H), 1.18-1.23 (m, 108H). ^{13}C NMR (d_6-acetone, 126 MHz): δ 196.13, 158.90, 153.45, 150.59, 150.53, 143.56, 139.56, 137.13, 134.10, 130.03, 129.55, 129.44, 129.31, 124.67, 123.67, 121.17, 116.29, 55.91, 54.97, 13.33, 13.20, 13.06, 7.89. ^{31}P NMR (d_6-acetone, 161.9 MHz): δ 14.09 (s, J_{Pt-P} = 2641.24 Hz). ESI-TOF-MS of **H7**: calcd for [M – 4OTf]$^{4+}$: 1178.3744, found: 1178.3419; calcd for [M – 5OTf]$^{5+}$: 913.1018, found: 913.1212.

Preparation of metallacycle H8

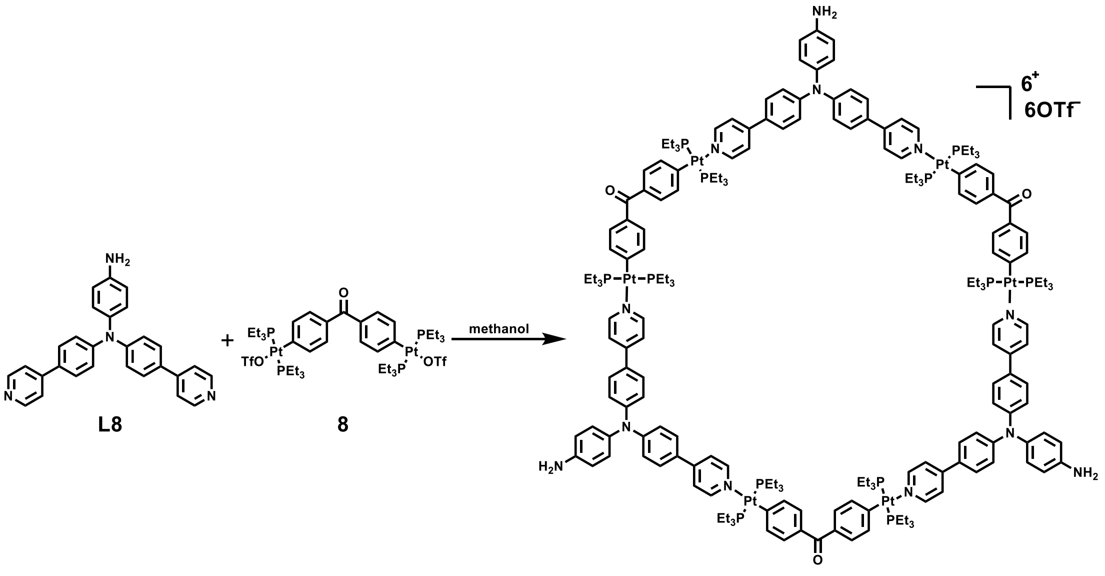

Supplementary Figure 205. Synthesis of metallacycle **H8**.

Metallacycle H8: The dipyridyl donor ligand **L8** (4.36 mg, 10.5 μmol) and the organoplatinum 120° acceptor **8** (14.1 mg, 10.5 μmol) were weighed accurately into a glass vial. To the vial was added 2.0 mL methanol and the reaction solution was then stirred at 60 ^{o}C for 4 h to yield a homogeneous light solution. Yellow solid product **H8** was obtained by removing the solvent under vacuum. Yield: 18.46 mg, >99%. M.p.: >300 ^{o}C. ^{1}H NMR (MeOD, 500 MHz) δ 8.76 (d, 12H, J = 5.3 Hz), 8.02 (d, 12H, J = 6.1 Hz), 7.88 (d, 12H, J = 8.7 Hz), 7.69 (d, 12H, J = 7.8 Hz), 7.53 (d, 12H, J = 7.8 Hz), 7.26 (d, 12H, J = 8.1 Hz), 7.01 (d, 6H, J = 8.6 Hz), 6.84 (d, 6H, J = 8.5 Hz), 1.54-1.40 (m, 72H), 1.24-1.15 (m, 108H). ^{13}C NMR (MeOD, 126 MHz): δ 199.03, 153.61, 151.72, 151.26, 137.68, 134.43, 130.85, 130.51, 129.83, 129.32, 124.77, 123.64, 120.57, 117.67, 13.65, 13.52, 13.38, 7.89. ^{31}P NMR (MeOD, 202 MHz): δ 13.88 (s, J_{Pt-P} = 2664.88 Hz). ESI-TOF-MS of **H8**: calcd for [M – 4OTf]$^{4+}$: 1167.3766, found: 1167.3419; calcd for [M – 5OTf]$^{5+}$: 904.1110, found: 904.1103.

Preparation of metallacycle H9

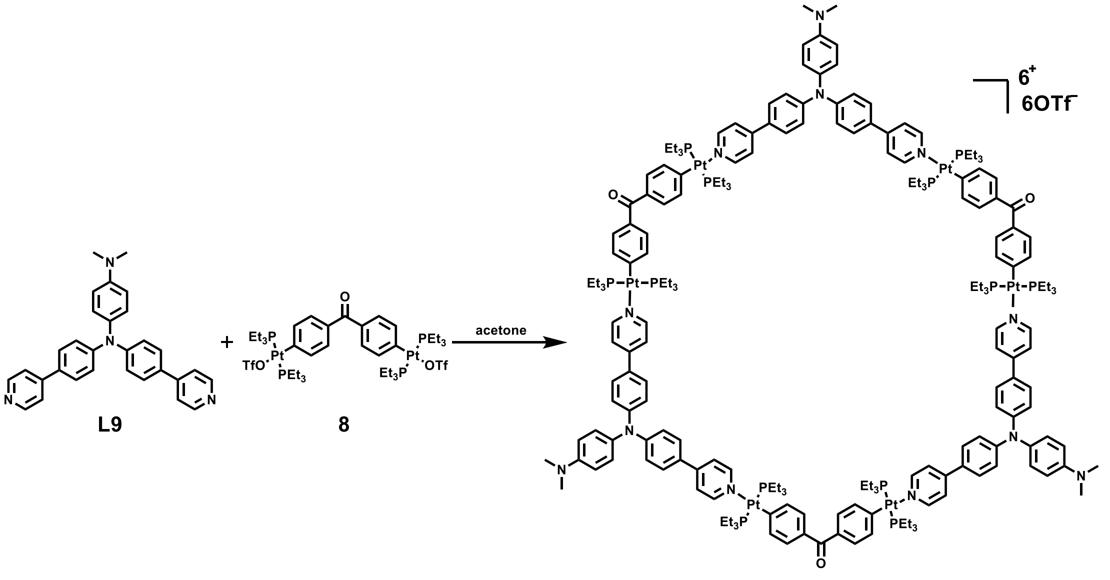

Supplementary Figure 206. Synthesis of metallacycle **H9**.

Metallacycle H9: The dipyridyl donor ligand **L9** (2.43 mg, 5.5 μmol) and the organoplatinum 120° acceptor **8** (7.36 mg, 5.5 μmol) were weighed accurately into a glass vial. To the vial was added 2.0 mL acetone and the reaction solution was then stirred at 40 °C for 4 h to yield a homogeneous light solution. Yellow solid product **H9** was obtained by removing the solvent under vacuum. Yield: 9.79 mg, >99%. M.p.: >300 °C. ^{1}H NMR (d_6-acetone, 400 MHz): δ 8.95 (d, 12H, J = 6 Hz), 8.13 (d, 12H, J = 6.8 Hz), 7.95 (d, 12H, J = 8.4 Hz), 7.71 (d, 12H, J = 8 Hz), 7.57 (d, 12H, J = 8 Hz), 7.25 (d, 12H, J = 8.8 Hz), 7.12 (d, 6H, J = 8.8Hz), 6.86 (d, 6H, J = 9.2 Hz), 3.01 (s, 18H), 1.51-1.52 (m,72H), 1.16-1.24 (m, 108H). ^{13}C NMR (d_6-acetone, 126 MHz): δ 195.28, 152.53, 149.77, 142.71, 136.26, 133.22, 129.15, 128.33, 123.69, 122.84, 122.51, 120.28, 36.42, 31.11, 12.45, 12.32, 12.18, 7.01. ^{31}P NMR (d_6-acetone, 161.9 MHz): δ 14.58 (s, J_{Pt-P} = 2662.60 Hz). ESI-TOF-MS of **H9**: calcd for [M – 4OTf]$^{4+}$: 1188.1482, found: 1188.3524; calcd for [M – 5OTf]$^{5+}$: 921.1313, found: 921.1271.

Preparation of compound 10

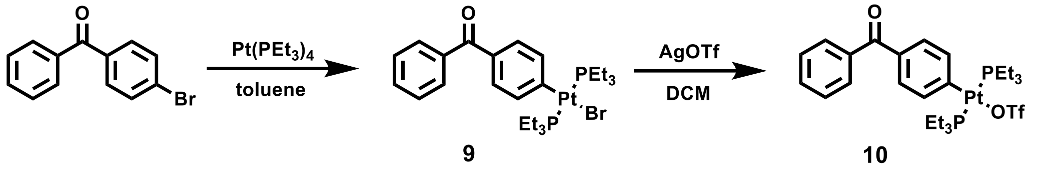

Supplementary Figure 207. Synthesis of **10**.

Compound 9: 4-bromobenzophenone (230 mg, 0.88 mmol) and $Pt(PEt_3)_4$ (1 g, 1.50 mmol) was added in freshly distilled toluene (15 mL) under argon. The resulting solution was then heated for 48 h in an oil bath maintained at 100 °C. The following day the solvent was removed in vacuo, the crude product was purified on silica gel column using CH_2Cl_2 and petroleum ether (1:2, *v*/*v*) as eluent to give white solid **9** (385 mg, 63%). R_f = 0.35(dichloromethane/petroleum ether 1/1). M.p.: 111 °C. ^{1}H NMR ($CDCl_3$, 300 MHz): δ 7.78-7.71 (m, 2H), 7.65-7.38 (m, 7H), 1.72-1.61 (m, 12H), 1.16–1.02 (m, 18H). ^{13}C NMR ($CDCl_3$, 126 MHz): δ 197.34, 150.44, 138.81, 137.43, 131.72, 131.32, 129.85, 129.34, 128.18, 13.77, 13.63, 13.50, 7.87. ^{31}P NMR ($CDCl_3$, 122 MHz): δ 14.18(s, $J_{Pt\text{-}P}$ = 2753.66 Hz). HRMS (MALDI): *m/z* Calcd for: 612.2121. Found: 612.2123 $(M\text{-}OTf)^+$.

Compound 10: Compound **9** (100 mg, 0.14 mmol) and silver trifluoromethanesulfonate (41 mg, 0.16 mmol) were added in dry dichloromethane (10 mL) under argon. The reaction was stirred for 4 h in the dark, after which the reaction mixture was filtered. The filtrate evaporated to dryness under reduced pressure to give yellow solid product **10**. Yield: 103 mg, 94%. M.p.: 90 °C. ^{1}H NMR (CD_2Cl_2, 500 MHz): δ 7.79-7.72 (m, 2H), 7.62 (t, 1H, *J* = 7.4 Hz), 7.52 (dd, 4H, *J* = 13.2, 5.7 Hz), 7.39 (d, 2H, *J* = 8.1 Hz), 1.77–1.63 (m, 12H), 1.26–1.10 (m, 18H). ^{13}C NMR (CD_2Cl_2, 126 MHz): δ 196.72, 138.81, 136.46, 132.16, 129.98, 129.65, 128.52, 14.20, 14.07, 13.94, 7.84. ^{31}P NMR (CD_2Cl_2, 202 MHz): δ 19.42(s, $J_{Pt\text{-}P}$ = 2787.20 Hz). HRMS (MALDI)**:** *m/z* Calcd for: 612.2121. Found: 612.2116 $(M\text{-}OTf)^+$.

Supplementary Discussion

Basic concept of photoinduced electron transfer (PET) and intramolecular charge transfer (ICT)

Photoinduced electron transfer (PET) is an excited state electron transfer process, in which an excited electron is transferred from donor to acceptor. As shown in Supplementary Figure 1, a typical PET molecule often includes three parts: a fluorophore that acts as the electron acceptor, a receptor that serves as an electron donor or a quencher, and a spacer that links the two parts of fluorophore and receptor. Fluorescent molecules based on PET are often structured as fluorophore-spacer-receptor constructs. In the PET system, the photoinduced electron transfer from the receptor to the fluorophore will induce fluorescence quenching. However, this photoinduced electron transfer (PET) process is restricted when the receptor binds upon its electron-withdrawing target (such as metal ions), which thus induces the enhancement of fluorescence emission.

The mechanism of PET-based fluorescent molecule can be explained by the frontier orbital theory. As shown in Supplementary Figure 2a, the electron of the highest occupied molecular orbital (HOMO) is transferred to the lowest unoccupied molecular orbital (LUMO) when the fluorophore is excited by an appropriate light wavelength. Because the HOMO energy level of the receptor is between the LUMO and HOMO levels of the fluorophore, the electron transfers from the HOMO of the receptor to the HOMO of the fluorophore. This electron transfer induces the prevention of the electron in the LUMO of the fluorophore from returning to the HOMO, and thus leads to a fluorescence quenching by means of the PET effect. However, as shown in Supplementary Figure 1b and Supplementary Figure 2b, when the receptor binds upon the electron-withdrawing target, the HOMO energy of the receptor is declined to be lower than the HOMO energy level of the fluorophore. Therefore, the inhibition of PET process induces the enhancement of fluorescence emission.

Intramolecular charge transfer (ICT) is the charge transfer from an electron-rich

donor moiety to an electron-poor acceptor part located in the same molecule. The ICT process generally occurs in the photoexcited state that a molecule reaches due to the absorption of light with an appropriate wavelength. The photoexcitation facilitates the transfer of an electron from one part of a molecule to its other part in the excited state, which leads to the charge distribution in the excited state markedly different from that in the ground state. Fluorescent molecules on the basis of ICT are featured by conjugation of an electron-donating unit to an electron-accepting unit in one molecule to rise a ‘‘push–pull’’ π-electron system in the excited state (Supplementary Figure 3). When the electron-accepting part interacts with an electron-withdrawing guest (such as metal ions), the electron-accepting character of the fluorescent molecule increases, thus generating a red shift in the emission spectrum (Supplementary Figure 3a and Figure 3b). In contrast, an evident blue shift can be observed when the ICT becomes less developed due to the interaction of the electron-donating part with an electron-withdrawing guest (Supplementary Figure 3c and Figure 3d).

Absorption and fluorescent emission spectra of metallacycle H6 at different concentrations

As shown in Supplementary Figure 124, upon increasing the concentration of metallacycle **H6** from 0.5 μM to 10 μM, the significant increases both in absorption spectra and fluorescent emission spectra were observed. However, as the concentration increased, metallacycle **H6** exhibited a small change in the fluorescence quantum yield (Supplementary Table 6). These results indicated that metallacycle **H6** didn't display the obvious AIEE effect.

The fluorescence lifetimes of ligands L1-L9 and metallacycles H1-H9

As shown in Supplementary Table 5, the fluorescence quantum yields of ligands **L1**-**L9** and metallacycles **H1**-**H9** tested in air were slightly lower than each of them measured under N_2 atmosphere, which might be attributed to the collisional quenching effect of oxygen. Moreover, most of fluorescence lifetimes of ligands and

metallacycles in air were slightly shorter than those under N_2 atmosphere (Supplementary Table 7-10), which might be also attributed to the quenching effect of oxygen resulting in an additional rate process that depopulates the excited state.

The steady-state emission and delayed emission spectra of metallacycles

As shown in Supplementary Figure 143, the absorption spectrum of metallacycles **H5-H9** tested under N_2 atmosphere was nearly the same as that tested in air, respectively. Moreover, the luminescence intensity of metallacycles **H5-H7** measured under N_2 atmosphere condition was slightly higher than that measured in air, respectively. In particular, the emission spectra of metallacycles **H8** and **H9** were found no significant changes between N_2 atmosphere and aerated atmosphere. The slight oxygen-sensitive delayed emission of metallacycles **H5-H7** could be considered as thermally activated delayed fluorescence from the first excited singlet state, which was obtained through the reverse intersystem crossing (RISC) process from oxygen-sensitive triplet excited state (T1) state.

Supplementary References

1. Shi, W., Fan, S., Huang, F., Yang, W., Liu, R. & Cao, Y. *J. Mater. Chem.* **16**, 2387–2394 (2006).

2. Wang, Z.-J., Qin, L., Zhang, X., Chen J.-X., & Zheng H.-G. *Cryst. Growth Des.* **15**, 1303−1310 (2015).

3. Jiang, Y., Wang, Y., Wang B., Yang, J., He, N., Qian, S. & Hua J. *Chem. Asian J*. **6**, 157−165 (2011).

4. Grigoras, M., Catargiu, A. M., Ivan, T., Vacareanu, L., Minaev, B. & Stromylo E. *Dyes and Pigments* **113**, 227–238 (2015).

5. Zhan, H., Liu, Q., Dai, F., Ho, C.-L., Fu, Y. Li, L., Zhao, L., Li, H., Xie, Z. & Wong, W.-Y. *Chem. Asian J.* **10**, 1017–1024 (2015).

6. He, M., Twieg, R. J., Gubler U., Wright, D & Moerner W. E. *Chem. Mater.* **15**, 1156–1164 (2003).
